# Supplementary material for: Improving Primary Care After Stroke (IPCAS) randomised controlled trial: protocol for a multidimensional process evaluation
Source: BMJ Open. 2020 Jul 8;10(7):e036879. doi: 10.1136/bmjopen-2020-036879 (PMC7348649; doi:10.1136/bmjopen-2020-036879)
Supplement: Supplementary data [file bmjopen-2020-036879supp001.pdf]

Supplementary file 1

IPCAS trial provider process evaluation/fidelity checklist – training phase								
**Site ID:**		**Duration of training:**			**Training date (Coding date):**			
**Activity**			**Tick as appropriate**			**If *present to some extent*, provide reason(s) for this rating**	**Other comments**	
			**Present**	**Absent**	**Partially done**			
**Structured review**	1. 15-item checklist of needs	a) Discussion of purpose and structure of checklist						
		b) Instructions on how to use checklist						
	2. Physical check	a) Discussion of carrying out optional physical checks at review (in line with QOF)						
	3. Development of action plan(s)	a) Discussion of action plan						
		b) Instructions given: how to log and review agreed actions (using template)						
	4. MLAS	a) Discussion of purpose and structure of MLAS						
		b) Provided instructions for how patients can access MLAS						
	5. Direct point of contact to stroke survivor and carer(s)	a) Given instructions for offering 'direct point of contact' service						
		b) Discussion of how to provide instructions to patients about contacting practice at the end of review						
**Use of direct point of contact**	1. Signposting to services	a) Discussed service mapping: what it is						
		b) Discussed how to use service mapping						

IPCAS fidelity of training checklist v1.1 Last updated: 14/01/2019

IPCAS trial provider process evaluation/fidelity checklist – training phase							
**Site ID:**		**Duration of training:**			**Training date (Coding date):**		
**Activity**			**Tick as appropriate**			**If *present to some extent*, provide reason(s) for this rating**	**Other comments**
			**Present**	**Absent**	**Partially done**		
	2. Provide support and advice	a) Gave information on structure and scope of direct point of contact service: giving advice for stroke-specific issues					
		b) Gave information on structure and scope of direct point of contact service: arranging follow-up appointment(s)					
		c) Gave information on structure and scope of direct point of contact service: brief telephone support/case management					
**Enhanced communication pathways**	1. Discussion on communication pathways	a) Discussed broad structure of enhanced communication pathways					
		b) Initial discussion about dates for face-to-face meeting					
**END OF CHECKLIST**							

Aquino MRJ(Ryc), *et al. BMJ Open* 2020; 10:e036879. doi: 10.1136/bmjopen-2020-036879
