# Supplementary material for: Improving Primary Care After Stroke (IPCAS) randomised controlled trial: protocol for a multidimensional process evaluation
Source: BMJ Open. 2020 Jul 8;10(7):e036879. doi: 10.1136/bmjopen-2020-036879 (PMC7348649; doi:10.1136/bmjopen-2020-036879)
Supplement: Supplementary data [file bmjopen-2020-036879supp002.pdf]

Supplementary file 2

IPCAS trial process evaluation/fidelity checklist
MLAS facilitator training

**Date:**					
**Start time:**					
**End time:**					
**Training day 1**					
	**Please tick as appropriate**			**If *absent, attempted* or *not applicable*, please provide reasons**	**Other comments**
	**Present**	**Absent**	**Attempted**		
**Session 1: Welcome and introductions**					
**Duration of session:**					
**Materials**					
Timetable available	☐	☐	☐		
Headed flipchart paper – working together	☐	☐	☐		
Pre-training 'evaluation' form given (1 per participant)	☐	☐	☐		
**Content**					
Welcome to MLAS	☐	☐	☐		
Introduction of trainers	☐	☐	☐		

V1.2 11/06/2018

**Date:**					
**Start time:**					
**End time:**					
**Training day 1**					
	**Please tick as appropriate**			**If *absent, attempted* or *not applicable*, please provide reasons**	**Other comments**
	**Present**	**Absent**	**Attempted**		
Housekeeping details provided	☐	☐	☐		
Aim of training discussed	☐	☐	☐		
Training overview discussed	☐	☐	☐		
Outline of Day 1 discussed	☐	☐	☐		
Working together discussed	☐	☐	☐		
Provided time for participants to complete pre-training ‘evaluation’ form	☐	☐	☐		

Aquino MRJ(Ryc), *et al. BMJ Open* 2020; 10:e036879. doi: 10.1136/bmjopen-2020-036879

**Session 2: Your story**					
**Duration of session:**					
**Materials**					
Headed flipchart paper (1: Name, role, place of work, 2: Experience, 3: Expectation of training, 4: Your important question)	☐	☐	☐		
**Content**					
Asked: Name, role, place of work	☐	☐	☐		
Asked: Experience (facilitating/observing groups, working with stroke)	☐	☐	☐		
Asked: Expectation(s)	☐	☐	☐		
Asked: Any question in relation to MLAS	☐	☐	☐		
Provided opportunity for each trainee to answer the above questions and record on relevant flipchart	☐	☐	☐		
**Session 3: What is stroke?**					
**Duration of session:**					
**Materials**					
Body outline used	☐	☐	☐		
Images shown: clot, bleed, brain areas	☐	☐	☐		
Images shown: risk factors, neuroplasticity	☐	☐	☐		

Aquino MRJ(Ryc), *et al. BMJ Open* 2020; 10:e036879. doi: 10.1136/bmjopen-2020-036879

Videos shown: Act FAST, neuroplasticity	☐	☐	☐		
Images shown: health and well-being	☐	☐	☐		
Sticky notes used	☐	☐	☐		
**Content**					
Discussed 'what is stroke?'	☐	☐	☐		
Discussed signs and symptoms – ACTFAST	☐	☐	☐		
Discussed impacts of a stroke (including physical, emotional, cognitive, communication, relationships)	☐	☐	☐		
Discussed what neuroplasticity is	☐	☐	☐		
Discussed stroke risk factors	☐	☐	☐		
Discussed managing risk factors	☐	☐	☐		
Participants given an opportunity to contribute to discussion and activities	☐	☐	☐		
**Session 4: Cognitive effects of stroke**					
**Duration of session:**					
**Materials**					
PowerPoint slides used: cognitive effects of stroke	☐	☐	☐		
Video shown: Hidden effects of stroke	☐	☐	☐		

Aquino MRJ(Ryc), *et al. BMJ Open* 2020; 10:e036879. doi: 10.1136/bmjopen-2020-036879

Booklet given: Complete guide to Cognitive problems (1 copy/facilitator)	☐	☐	☐		
**Content**					
Session introduced	☐	☐	☐		
Discussed memory problems after a stroke	☐	☐	☐		
Explored attention difficulties	☐	☐	☐		
Explored changes to perception	☐	☐	☐		
Explored effects on decision-making	☐	☐	☐		
Explored social cognition problems	☐	☐	☐		
Discussed how we can support stroke survivors experiencing these effects/top tips	☐	☐	☐		
Discussed the impact of impaired cognition on the stroke survivor and/or carer/family	☐	☐	☐		
Participants given an opportunity to contribute to discussion and activities	☐	☐	☐		
**Session 5: Emotional changes after stroke**					
**Duration of session:**					
**Materials**					
Video shown: The emotional impact of stroke	☐	☐	☐		
PowerPoint slides: Emotional problems following stroke					

Booklet given: Complete guide to Emotional changes after stroke (1 copy/facilitator)	☐	☐	☐		
**Content**					
Session introduced	☐	☐	☐		
Discussed the emotions stroke survivors highlighted in video observed	☐	☐	☐		
Discussed stages of loss	☐	☐	☐		
Discussed problems stroke survivors might face with emotions	☐	☐	☐		
Discussed difficulty controlling emotions	☐	☐	☐		
Discussed how these emotions can influence people's thoughts and behaviours	☐	☐	☐		
Discussed how facilitators can support stroke survivors experiencing these effects/top tips	☐	☐	☐		
Participants given an opportunity to contribute to discussion and activities	☐	☐	☐		
**Session 6: Relationships**					
**Duration of session:**					
**Materials**					
PowerPoint slides used: Relationships	☐	☐	☐		
**Content**					

Session introduced	☐	☐	☐		
Discussed the effect of a stroke on relationships (partners, friends, family members)	☐	☐	☐		
Discussed problems and solutions concerning physical intimacy after a stroke	☐	☐	☐		
Participants given an opportunity to contribute to discussion and activities	☐	☐	☐		
**Session 7: Communication challenges for survivors of stroke**					
**Duration of session:**					
**Materials**					
PowerPoint slides used: Communication	☐	☐	☐		
Video used: Living with aphasia; aphasia etiquette	☐	☐	☐		
Booklet given: Complete guide to Communication problems after stroke (1 copy/facilitator)	☐	☐	☐		
**Content**					
Session introduced	☐	☐	☐		
Group activity: Identifying tasks the brain needs to complete in order to be able to answer a question	☐	☐	☐		

Aquino MRJ(Ryc), *et al. BMJ Open* 2020; 10:e036879. doi: 10.1136/bmjopen-2020-036879

Discussed communication problems after a stroke	☐	☐	☐		
Discussed how we can support stroke survivors experiencing communication problems (top tips, resources/aids)	☐	☐	☐		
Participants given an opportunity to contribute to discussion and activities	☐	☐	☐		
**Session 8: Summary and evaluation**					
**Duration of session:**					
**Materials**					
Training evaluation form given	☐	☐	☐		
Questions relating to MLAS flipchart	☐	☐	☐		
**Content**					
Summary of Day 1 Training	☐	☐	☐		
Provided time for participants to complete training evaluation form	☐	☐	☐		
Provided details of Day 2 Training	☐	☐	☐		
Participants given an opportunity to check if their questions addressed, and to ask any further questions	☐	☐	☐		
**END OF DAY 1 TRAINING**					

Aquino MRJ(Ryc), *et al. BMJ Open* 2020; 10:e036879. doi: 10.1136/bmjopen-2020-036879

IPCAS trial process evaluation/fidelity checklist
MLAS facilitator training

**Date:**					
**Start time:**					
**End time:**					
**Training day 2**					
	**Please tick as appropriate**			**If *absent, attempted* or *not applicable*, please provide reasons**	**Other comments**
	**Present**	**Absent**	**Attempted**		
**Session 1: Welcome and introductions**					
**Duration of session:**					
**Materials**					
Timetable available	☐	☐	☐		
Headed flipchart paper from day 1 ('Your story' - name, experience, expectation, important question and working together as a group)	☐	☐	☐		
**Content**					
Welcome	☐	☐	☐		
Introduction of trainers	☐	☐	☐		

Aquino MRJ(Ryc), *et al. BMJ Open* 2020; 10:e036879. doi: 10.1136/bmjopen-2020-036879

**Date:**					
**Start time:**					
**End time:**					
**Training day 2**					
	**Please tick as appropriate**			**If *absent, attempted* or *not applicable*, please provide reasons**	**Other comments**
	**Present**	**Absent**	**Attempted**		
Housekeeping details provided	☐	☐	☐		
Overall aim/format of training discussed	☐	☐	☐		
Outline of Day 2 discussed	☐	☐	☐		
Invited any new participants to introduce themselves to group using 'Your story' flipchart from Day 1	☐	☐	☐		
**Session 2: Background to MLAS**					
**Duration of session:**					
**Materials**					
Flipchart paper, pens	☐	☐	☐		
A4 Quadrant image	☐	☐	☐		
**Content**					
Discussed what MLAS is	☐	☐	☐		

**Date:**					
**Start time:**					
**End time:**					
**Training day 2**					
	**Please tick as appropriate**			**If *absent, attempted* or *not applicable*, please provide reasons**	**Other comments**
	**Present**	**Absent**	**Attempted**		
Discussed what a structured education programme is	☐	☐	☐		
Discussed rationale for MLAS	☐	☐	☐		
Discussed aims and format of MLAS course	☐	☐	☐		
Participants given an opportunity to be involved in discussion and ask questions	☐	☐	☐		
**Session 3: Philosophy**					
**Duration of session:**					
**Materials**					
How are you doing? Worksheet (1 per participant)	☐	☐	☐		
PowerPoint slides used: Philosophy	☐	☐	☐		
**Content**					

Aquino MRJ(Ryc), *et al. BMJ Open* 2020; 10:e036879. doi: 10.1136/bmjopen-2020-036879

**Date:**					
**Start time:**					
**End time:**					
**Training day 2**					
	**Please tick as appropriate**			**If *absent, attempted* or *not applicable*, please provide reasons**	**Other comments**
	**Present**	**Absent**	**Attempted**		
Discussed what philosophy is and how it affects our behaviours	☐	☐	☐		
Explored the medical model and empowerment approach	☐	☐	☐		
Explored the philosophy and approach of the MLAS programme	☐	☐	☐		
Facilitated individual activity ('How are you doing' quiz) and gave an opportunity for each trainee to complete their own worksheet	☐	☐	☐		
Discussed the need to have importance, confidence and readiness in order to change behaviour (and not just knowledge)	☐	☐	☐		
Presented overall aim of MLAS and its core philosophy statements	☐	☐	☐		
Discussed MLAS facilitators' role					

**Date:**					
**Start time:**					
**End time:**					
**Training day 2**					
	**Please tick as appropriate**			**If *absent, attempted* or *not applicable*, please provide reasons**	**Other comments**
	**Present**	**Absent**	**Attempted**		
Group activity: Challenges experienced/perceived by facilitators when using the philosophy/approach underpinning MLAS	☐	☐	☐		
Participants given an opportunity to be involved in discussion (e.g. by giving feedback) and ask questions	☐	☐	☐		
**Session 4: Facilitator behaviours**					
**Duration of session:**					
**Materials**					
Rice pudding exercise	☐	☐	☐		
MLAS facilitator behaviours checklist given (1 per participant)	☐	☐	☐		
**Content**					

**Date:**					
**Start time:**					
**End time:**					
**Training day 2**					
	**Please tick as appropriate**			**If *absent, attempted* or *not applicable*, please provide reasons**	**Other comments**
	**Present**	**Absent**	**Attempted**		
Session introduced	☐	☐	☐		
Rice pudding activity (demonstrating different facilitator behaviours – 'good' and 'bad')	☐	☐	☐		
Discussed positive and negative facilitator behaviours that were observed	☐	☐	☐		
Discussed some of the behaviours on the behaviours checklist, and provided examples of what would be observed if such behaviours were being done (e.g. body language, words used etc.)	☐	☐	☐		
Encouraged participants to engage in discussion about any stroke-specific behaviours needed	☐	☐	☐		
Participants given an opportunity to be involved in discussion (e.g. by giving feedback) and ask questions	☐	☐	☐		

Aquino MRJ(Ryc), *et al. BMJ Open* 2020; 10:e036879. doi: 10.1136/bmjopen-2020-036879

**Date:**					
**Start time:**					
**End time:**					
**Training day 2**					
	**Please tick as appropriate**			**If *absent, attempted* or *not applicable*, please provide reasons**	**Other comments**
	**Present**	**Absent**	**Attempted**		
**Session 5: Facilitation skills**					
**Duration of session:**					
**Materials**					
PowerPoint slides used: Facilitation skills	☐	☐	☐		
Flipchart paper and pens					
Open-ended Questions, Affirmations, Reflective listening, Summaries (OARS) sheets given (1 per participant)	☐	☐	☐		
**Content**					
Session introduced	☐	☐	☐		
Facilitated group activity on facilitator role and responsibilities	☐	☐	☐		
OARS introduced to participants	☐	☐	☐		

Aquino MRJ(Ryc), *et al. BMJ Open* 2020; 10:e036879. doi: 10.1136/bmjopen-2020-036879

**Date:**					
**Start time:**					
**End time:**					
**Training day 2**					
	**Please tick as appropriate**			**If *absent, attempted* or *not applicable*, please provide reasons**	**Other comments**
	**Present**	**Absent**	**Attempted**		
Group activity: Challenging scenarios and resolutions	☐	☐	☐		
Encouraged participants to share personal learning	☐	☐	☐		
Participants given an opportunity to be involved in discussion (e.g. by giving feedback) and ask questions	☐	☐	☐		
**Session 6: Theories – Narrative Approach; Self-efficacy**					
**Duration of session:**					
**Materials**					
PowerPoint slides used: Theories	☐	☐	☐		
Sticky notes, flipchart paper, pens	☐	☐	☐		
MLAS Roadmap and accessories	☐	☐	☐		
**Content**					

**Date:**					
**Start time:**					
**End time:**					
**Training day 2**					
	**Please tick as appropriate**			**If *absent, attempted* or *not applicable*, please provide reasons**	**Other comments**
	**Present**	**Absent**	**Attempted**		
Session introduced	☐	☐	☐		
Introduced the Narrative Approach	☐	☐	☐		
Demonstrated how the narrative approach is used in MLAS (journey analogy)	☐	☐	☐		
Introduced Self-efficacy (Mastery, vicarious learning, verbal persuasion and emotions management)	☐	☐	☐		
Group activity: Real-world experiences of self-efficacy	☐	☐	☐		
Examples provided where self-efficacy is used during MLAS	☐	☐	☐		
Introduced cognitive behavioural approach and gave an example of how it is used in MLAS	☐	☐	☐		

Aquino MRJ(Ryc), *et al. BMJ Open* 2020; 10:e036879. doi: 10.1136/bmjopen-2020-036879

**Date:**					
**Start time:**					
**End time:**					
**Training day 2**					
	**Please tick as appropriate**			**If *absent, attempted* or *not applicable*, please provide reasons**	**Other comments**
	**Present**	**Absent**	**Attempted**		
Participants given an opportunity to be involved in discussion (e.g. by giving feedback) and ask questions	☐	☐	☐		
**Session 7: Introduction to Quality Assurance**					
**Duration of session:**					
**Materials**					
Flipchart paper and pens	☐	☐	☐		
**Content**					
Session introduced	☐	☐	☐		
Discussed what quality assurance is and its important	☐	☐	☐		
Facilitated small group activity (recipe and ingredients for making an Apple Pie)	☐	☐	☐		

Aquino MRJ(Ryc), *et al. BMJ Open* 2020; 10:e036879. doi: 10.1136/bmjopen-2020-036879

**Date:**					
**Start time:**					
**End time:**					
**Training day 2**					
	**Please tick as appropriate**			**If *absent, attempted* or *not applicable*, please provide reasons**	**Other comments**
	**Present**	**Absent**	**Attempted**		
Encouraged each group to feedback on group activity.	☐	☐	☐		
Compared similarities and differences of Apple Pie recipes and linked to quality assurance of MLAS (ingredients = curriculum, method = delivery)	☐	☐	☐		
Summarised group discussion on quality assurance processes	☐	☐	☐		
Participants given an opportunity to be involved in discussion (e.g. by giving feedback) and ask questions	☐	☐	☐		
**Session 8: A journey through the MLAS curriculum**					
**Duration of session:**					
**Materials**					

**Date:**					
**Start time:**					
**End time:**					
**Training day 2**					
	**Please tick as appropriate**			**If *absent, attempted* or *not applicable*, please provide reasons**	**Other comments**
	**Present**	**Absent**	**Attempted**		
MLAS curriculum given to each facilitator	☐	☐	☐		
Participant (stroke survivor) handbooks given to each facilitator (4 handbooks in total)	☐	☐	☐		
A journey through the curriculum question sheet given to each facilitator	☐	☐	☐		
**Content**					
Provided an overview of materials given	☐	☐	☐		
Explained the purpose of 'a journey through the curriculum question sheet' and instructions for completion	☐	☐	☐		
**Session 9: Close of the day reflections and evaluation**					
**Duration of session:**					
**Materials**					
Training evaluation form (day 2) given	☐	☐	☐		

**Date:**					
**Start time:**					
**End time:**					
**Training day 2**					
	**Please tick as appropriate**			**If *absent, attempted* or *not applicable*, please provide reasons**	**Other comments**
	**Present**	**Absent**	**Attempted**		
Questions relating to MLAS flipchart	☐	☐	☐		
**Content**					
Summary of Day 2 training by encouraging participants to share learning about what was covered	☐	☐	☐		
Provided time for participants to complete training evaluation form	☐	☐	☐		
Provided details of Day 3 Training	☐	☐	☐		
Participants given an opportunity to check if their question (from flipchart) was addressed, and to ask any further questions	☐	☐	☐		
**END OF DAY 2 TRAINING**					

Aquino MRJ(Ryc), *et al. BMJ Open* 2020; 10:e036879. doi: 10.1136/bmjopen-2020-036879

IPCAS trial process evaluation/fidelity checklist

MLAS facilitator training

**Date:**					
**Start time:**					
**End time:**					
**Training day 3**					
	**Please tick as appropriate**			**If *absent* or *attempted* please provide reasons**	**Other comments**
	**Present**	**Absent**	**Attempted**		
**Session 1: Welcome and introductions**					
**Duration of session:**					
**Materials**					
Timetable available	☐	☐	☐		
Headed flipchart paper from days 1+2 ('Your story' - name, experience, expectation, important question and working together as a group)	☐	☐	☐		
**Content**					
Welcome	☐	☐	☐		

**Date:**					
**Start time:**					
**End time:**					
**Training day 3**					
	**Please tick as appropriate**			**If *absent* or *attempted* please provide reasons**	**Other comments**
	**Present**	**Absent**	**Attempted**		
Re-introduction of trainers	☐	☐	☐		
Housekeeping details provided	☐	☐	☐		
Aim of training discussed	☐	☐	☐		
Outline of Day 3 discussed	☐	☐	☐		
Invited any new participants to introduce themselves to group using 'Your story' flipchart from Day 1+2	☐	☐	☐		
**Session 2: Reflection from 'A journey through the curriculum' activity**					
**Duration of session:**					
**Materials**					
A journey through the curriculum activity sheet	☐	☐	☐		
A journey through the curriculum activity answer sheet given	☐	☐	☐		
MLAS curriculum	☐	☐	☐		
**Content**					

Aquino MRJ(Ryc), *et al. BMJ Open* 2020; 10:e036879. doi: 10.1136/bmjopen-2020-036879

Date:					
**Start time:**					
**End time:**					
**Training day 3**					
	**Please tick as appropriate**			**If *absent* or *attempted* please provide reasons**	**Other comments**
	**Present**	**Absent**	**Attempted**		
Invited participants to share experiences of completing 'a journey through the curriculum' activity	☐	☐	☐		
Discussed format of the programme (1:1 and group-based sessions; interchangeable sessions)	☐	☐	☐		
Discussed format of the curriculum (e.g. key for how to use the curriculum, duration for each section, section overviews)	☐	☐	☐		
Discussed involvement of carers in MLAS and format of this	☐	☐	☐		
Participants given an opportunity to be involved in discussion and ask questions	☐	☐	☐		
**Session 3: A walk through the MLAS curriculum**					
**Duration of session:**					
**Materials**					

**Date:**					
**Start time:**					
**End time:**					
**Training day 3**					
	**Please tick as appropriate**			**If *absent* or *attempted* please provide reasons**	**Other comments**
	**Present**	**Absent**	**Attempted**		
MLAS curriculum	☐	☐	☐		
Participant (stroke survivor) handbooks	☐	☐	☐		
Resources/accessories for programme available	☐	☐	☐		
**Content**					
Discussed the overall aims of the study and the intervention	☐	☐	☐		
Presented each of the MLAS sessions with the resources	☐	☐	☐		
Demonstrated how to use the resources	☐	☐	☐		
Demonstrated links between the sessions and materials to the theories and philosophy applied	☐	☐	☐		
Discussed challenges to delivery and strategies for addressing these	☐	☐	☐		
Discussed tailoring of sessions	☐	☐	☐		

Aquino MRJ(Ryc), *et al. BMJ Open* 2020; 10:e036879. doi: 10.1136/bmjopen-2020-036879

**Date:**					
**Start time:**					
**End time:**					
**Training day 3**					
	**Please tick as appropriate**			**If *absent* or *attempted* please provide reasons**	**Other comments**
	**Present**	**Absent**	**Attempted**		
Discussed some examples of what previous facilitators have observed/dealt with during previous courses	☐	☐	☐		
Participants given an opportunity to be involved in discussion and ask questions	☐	☐	☐		
**Session 4: Preparing for having a go**					
**Duration of session:**					
**Materials**					
Facilitator curricula	☐	☐	☐		
'Have a go' cards (1 per facilitator)	☐	☐	☐		
Roadmap and accessories available	☐	☐	☐		
A4 images: blood clot, bleed available	☐	☐	☐		
A6 images: risk factors, risks I can/cannot change available	☐	☐	☐		

**Date:**					
**Start time:**					
**End time:**					
**Training day 3**					
	**Please tick as appropriate**			**If *absent* or *attempted* please provide reasons**	**Other comments**
	**Present**	**Absent**	**Attempted**		
A4 and A6 images: health and wellbeing available	☐	☐	☐		
A4 images: dashboard available	☐	☐	☐		
Writing instruments available (sticky notes, flipchart paper, pens, Blu Tack)	☐	☐	☐		
**Content**					
Session introduced	☐	☐	☐		
Participants divided into 2 groups	☐	☐	☐		
Explained that each participant has been given details of a section of the MLAS course (to be delivered in the next session of training)	☐	☐	☐		
Given instructions for next steps (i.e. time to prepare for delivery of chosen 10-minute section, how 'have a go' session will run)	☐	☐	☐		

**Date:**					
**Start time:**					
**End time:**					
**Training day 3**					
	**Please tick as appropriate**			**If *absent* or *attempted* please provide reasons**	**Other comments**
	**Present**	**Absent**	**Attempted**		
Offered participants support for preparing for their sessions (e.g. providing relevant resources, explaining content)	☐	☐	☐		
Provided opportunity/time for facilitators to prepare for 10-minute session	☐	☐	☐		
Participants given an opportunity to be involved in discussion and ask questions	☐	☐	☐		
**Session 5: Have a go**					
**Duration of session:**					
**Materials**					
Facilitator curricula	☐	☐	☐		
'Have a go' cards (x2 sets) given out to participants	☐	☐	☐		
Roadmap and accessories displayed	☐	☐	☐		
A4 images: blood clot, bleed displayed	☐	☐	☐		

Aquino MRJ(Ryc), *et al. BMJ Open* 2020; 10:e036879. doi: 10.1136/bmjopen-2020-036879

**Date:**					
**Start time:**					
**End time:**					
**Training day 3**					
	**Please tick as appropriate**			**If *absent* or *attempted* please provide reasons**	**Other comments**
	**Present**	**Absent**	**Attempted**		
A6 images: risk factors, risks I can/cannot change displayed	☐	☐	☐		
A4 and A6 images: health and wellbeing displayed	☐	☐	☐		
A4 images: dashboard displayed	☐	☐	☐		
Writing instruments given (sticky notes, flipchart paper)	☐	☐	☐		
**Content**					
Give instructions on delivery of 10-minute sessions	☐	☐	☐		
Inform participants of aim of practice delivery (i.e. familiarise self with MLAS, increase confidence)	☐	☐	☐		
Give instructions about giving feedback on facilitator behaviours (3 things you did well)	☐	☐	☐		

Aquino MRJ(Ryc), *et al. BMJ Open* 2020; 10:e036879. doi: 10.1136/bmjopen-2020-036879

**Date:**					
**Start time:**					
**End time:**					
**Training day 3**					
	**Please tick as appropriate**			**If *absent* or *attempted* please provide reasons**	**Other comments**
	**Present**	**Absent**	**Attempted**		
Each facilitator given an opportunity to deliver their 10-minute session	☐	☐	☐		
Ensured everyone has written 3 positive facilitator behaviours that they saw on a sticky note and give to facilitator	☐	☐	☐		
Provided 3 positive facilitator behaviours and 1 'even better if…'	☐	☐	☐		
Encourage participants to share summaries and feedback on their experience	☐	☐	☐		
**Session 6: Reviewing confidence**					
**Duration of session:**					
**Materials**					
'How confident do I feel?' worksheet	☐	☐	☐		
**Content**					

**Date:**					
**Start time:**					
**End time:**					
**Training day 3**					
	**Please tick as appropriate**			**If *absent* or *attempted* please provide reasons**	**Other comments**
	**Present**	**Absent**	**Attempted**		
Explores participants' confidence levels to deliver MLAS	☐	☐	☐		
Explore what participants can do to increase their personal confidence to deliver MLAS	☐	☐	☐		
**Session 7: Quality development/Intervention fidelity**					
**Duration of session:**					
**Materials**					
Facilitator behaviours reflection toolkit	☐	☐	☐		
Quality development and intervention fidelity pathway	☐	☐	☐		
**Content**					
Recapped purpose of quality assurance	☐	☐	☐		
Highlighted the importance of self- and peer-reflection	☐	☐	☐		
Discussed facilitator behaviour reflection toolkit	☐	☐	☐		

Aquino MRJ(Ryc), *et al. BMJ Open* 2020; 10:e036879. doi: 10.1136/bmjopen-2020-036879

**Date:**					
**Start time:**					
**End time:**					
**Training day 3**					
	**Please tick as appropriate**			**If *absent* or *attempted* please provide reasons**	**Other comments**
	**Present**	**Absent**	**Attempted**		
Discussed quality assurance pathway	☐	☐	☐		
Explored what intervention fidelity is					
Described the process for intervention fidelity assessment	☐	☐	☐		
Participants given an opportunity to be involved in discussion (e.g. by giving feedback) and ask questions	☐	☐	☐		
**Session 8: Summary, and post reflections, evaluation**					
**Duration of session:**					
**Materials**					
Training evaluation form given	☐	☐	☐		
Questions relating to MLAS flipchart	☐	☐	☐		
**Content**					

Aquino MRJ(Ryc), *et al. BMJ Open* 2020; 10:e036879. doi: 10.1136/bmjopen-2020-036879

**Date:**					
**Start time:**					
**End time:**					
**Training day 3**					
	**Please tick as appropriate**			**If *absent* or *attempted* please provide reasons**	**Other comments**
	**Present**	**Absent**	**Attempted**		
Summary of Day 3 training by encouraging participants to share learning about what was covered today	☐	☐	☐		
Provided time for participants to complete training evaluation (day 3) form	☐	☐	☐		
Participants given an opportunity to check if their questions addressed, and to ask any further questions	☐	☐	☐		
Thanked everyone for attending and contributing	☐	☐	☐		
**END OF DAY 3 TRAINING**					
