# Supplementary material for: Improving Primary Care After Stroke (IPCAS) randomised controlled trial: protocol for a multidimensional process evaluation
Source: BMJ Open. 2020 Jul 8;10(7):e036879. doi: 10.1136/bmjopen-2020-036879 (PMC7348649; doi:10.1136/bmjopen-2020-036879)
Supplement: Supplementary data [file bmjopen-2020-036879supp003.pdf]

Supplementary file 3

Training evaluation form

My Life After Stroke - Day 1 - Stroke

Date:_________________

Thank you for attending the Stroke day (Day 1) of the My Life After Stroke (MLAS) training. We would value your reflection on the training you have received today in the following areas. Please place a tick in the relevant box, indicating how much you agree or disagree with each of the following statements:

	Strongly agree	Agree	Neither agree nor disagree	Disagree	Strongly disagree
I am aware of the cognitive problems that can occur following stroke					
I am aware of some strategies that may help stroke survivors manage the effects of cognitive problems					
I have an understanding of the emotional problems that may follow a stroke					
I am aware of some strategies to support stroke survivors manage the effects of their emotional problems					
I have an appreciation of the effects having a stroke could put on relationships					
I am aware of some strategies that may help stroke survivors manage the effects on their relationships					
I have an awareness of communication problems following a stroke					
I have an awareness of how to communicate effectively with stroke survivors					

Continued overleaf…

Day 1 MLAS – Stroke evaluation continued…

	Strongly agree	Agree	Neither agree nor disagree	Disagree	Strongly disagree
I had the opportunity to ask questions and for these to be addressed					
I learnt something new today					

We would value your opinion on the training you attended today. Please answer the following questions:

Which were the three most helpful aspects of the training to you?<br>1.<br>2.<br>3.
Which were the three least helpful aspects of the training to you?<br>1.<br>2.<br>3.
Which part of the training did you find most enjoyable?
Please identify at least one way that the training could be improved?
Any other comments?

Thank you for completing this evaluation sheet

Training evaluation form

My Life After Stroke - Day 2 – Core training

Date:_________________

Thank you for attending the Core day (Day 2) of the My Life After Stroke (MLAS) training. We would value your reflection on the training you have received today in the following areas. Please place a tick in the relevant box, indicating how much you agree or disagree with each of the following statements:

	Strongly agree	Agree	Neither agree nor disagree	Disagree	Strongly disagree
I am aware of the aims of the MLAS programme					
I am aware of why MLAS has been developed					
My role as an MLAS facilitator is to:					
• Demonstrate a curious, non-judgemental approach towards participants					
• Support participants to explore their personal thoughts and feelings					
• Avoid giving specific advice and instead support participants to develop their own solutions and strategies to the challenges they face					
• Facilitate the groups awareness of sources of support and information					
• Promote the sharing of knowledge and ideas between participants and sign post where appropriate					
I have an understanding of the theories that underpin the MLAS programme					
I have an understanding of how these theories have been integrated practically into the MLAS programme					

Continued overleaf…

Day 2 MLAS Core evaluation continued…

	Strongly agree	Agree	Neither agree nor disagree	Disagree	Strongly disagree
I had the opportunity to ask questions and for these to be addressed					
I have learnt new skills					
I believe I can apply the facilitation skills					
I understand the importance of quality assurance					

I have been given an MLAS curriculum. **Yes / No** (delete as appropriate)

We would value your opinion on the training you attended today. Please answer the following questions:

Which were the three most helpful aspects of the training to you?<br>1.<br><br>2.<br><br>3.
Which were the three least helpful aspects of the training to you?<br>1.<br><br>2.<br><br>3.
Which part of the training did you find most enjoyable?
Please identify at least one way that the training could be improved?
Any other comments?

Thank you for completing this evaluation sheet

Training evaluation form

My Life After Stroke - Day 3 – MLAS-specific training

Date:_________________

Thank you for attending the MLAS-specific day (Day 3) of the My Life After Stroke (MLAS) training. We would value your reflection on the training you have received today in the following areas. Please place a tick in the relevant box, indicating how much you agree or disagree with each of the following statements:

	Strongly agree	Agree	Neither agree nor disagree	Disagree	Strongly disagree
I am aware of how to use the MLAS curriculum					
I have an understanding of the content of the MLAS programme					
I have an understanding of how MLAS can be delivered					
I had the opportunity to see the resources that are used during the MLAS programme					
I found the 'having a go' session useful					
I feel able to begin to plan delivering MLAS					
I feel confident that I will be able to facilitate MLAS					
I had the opportunity to ask questions and for these to be addressed					
I have learnt new skills					
I believe I can apply the skills					

I had the opportunity to deliver a section of the curriculum:

Yes / No / Don't know (please circle)

I received a copy of the Reflection Toolkit:

Yes / No / Don't know (please circle)

Continued overleaf…

Day 3 MLAS-specific evaluation continued…

We would value your opinion on the training you attended today. Please answer the following questions:

Which were the three most helpful aspects of the training to you?<br>1.<br><br>2.<br><br>3.
Which were the three least helpful aspects of the training to you?<br>1.<br><br>2.<br><br>3.
Which part of the training did you find most enjoyable?
Please identify at least one way that the training could be improved?
Any other comments?

Thank you for completing this evaluation sheet
