# Supplementary material for: Improving Primary Care After Stroke (IPCAS) randomised controlled trial: protocol for a multidimensional process evaluation
Source: BMJ Open. 2020 Jul 8;10(7):e036879. doi: 10.1136/bmjopen-2020-036879 (PMC7348649; doi:10.1136/bmjopen-2020-036879)
Supplement: Supplementary data [file bmjopen-2020-036879supp004.pdf]

Supplementary file 4

IPCAS trial provider process evaluation/fidelity checklist							
Site ID:			Time of call:		Date: of coding:		
Spoke to (role):			Conducted by:		Duration of call:		
**Activity**			**Tick as appropriate**			**If *not done* or *attempted*, please provide reason for not delivering (e.g. ran out of time, forgot, deemed unnecessary).**	**Other comments**
			**Done**	**Partially done**	**Not done**		
**Structured review**	1. 15-item checklist of needs	a) Patient completed checklist *(Thinking back to the most recent review that you did... Did the patient bring a completed checklist of needs?)*					
		b) Discussion of up to 3 needs (as ranked by importance by patient)					
	2. Physical check *(Did you conduct any physical checks at all? If yes what were these? If no/unsure, explore reasons for this (e.g. might've already been done recently))*	a) Blood pressure					
		b) Medication review					
		c) Other (specify)					
	3. Development of action plan(s)	a) Discuss action plan *(Together with the stroke survivor, did you discuss any plans of action going forward to address their needs? If no/unsure, explore reasons why)*					
		b) Log and review of agreed actions *(Cannot be filled if answer to a) is no; however, if yes, ask: Did you make a note of these actions and review or agree it with the patient?)*					
	4. MLAS	a) Provided leaflet about MLAS					
		b) Provided instructions for accessing MLAS					
	5. Direct point of contact to stroke survivor and carer(s)	a) Explain 'direct point of contact' service *(I will now ask questions about the Direct Point of Contact service. Did you discuss/explain the service to the patient)*					
		b) Provide instructions on how to contact practice at the end of review					
	6. Service mapping tool *(When giving information and/or advice, did you refer to the searchable service mapping tool provided by us?)*						
		**TOTAL**	0	0	0		
		**PERCENTAGE**	0	0	0		
**Use of direct point of contact** *(Following on from my previous questions, since the reviews you conducted, has anyone called into the Direct Point of Contact service? If yes, think back to the most recent call. Did you signpost the patient to a specialist service? What about a community-based service?)*	1. Signposting to services	a) Signpost patient to specialist service					
		b) Signpost patient to community service					
	2. Use of service mapping for signposting (as above) *(When you offered information to the patient about either specialist/community services, did you make use of the service mapping tool (i.e. directory) at all?)*						
	3. Provide support and advice	a) Advice for stroke-specific issues					
		b) Other brief telephone support *(Beyond stroke-specific issues, did you offer any other support over the phone?)*					
	4. Arrange follow-up appointment(s)						
	5. Case management *(Finally, during the call, did you offer the patient anything else other than what we had already discussed? E.g. (comprehensive assessment of needs; development of an individualised care plan in collaboration with the service user and relevant others (including families and carers and other staff involved in the service user's care; any additional care given))*						
		**TOTAL**	0	0	0		
		**PERCENTAGE**	0	0	0		
**Enhanced communication pathways**	1. Initial meeting *(We are coming towards the end of the call now, and I wanted to ask some questions about your contact with colleagues in secondary care. Have you had your initial meeting? If no, note reasons why. If yes, ask: Did you have a discussion about your roles, etc. (Note whether meeting was via videoconference or face-to-face))*	a) Discussion of roles/services provided/contact details (video recorded)					
		b) Agreed action plan regarding communication between primary care staff and specialist staff *(In this meeting, did you agree a plan going forward about how best to communicate with each other?)*					
	2. Further contact	a) Meeting (e.g. teleconference, face-to-face, videoconference) *(Since the initial meeting, did you have any further contacts? Tell me about them (then tick off if there was a meeting, or email/phone contact))*					
		b) Other contact (e.g. telephone, email, letter)					
		**TOTAL**	0	0	0		
		**PERCENTAGE**	0	0	0		
**Duration of structured review**	On average, how long did each review run for?						
**END OF CHECKLIST**							
