# Supplementary material for: Improving Primary Care After Stroke (IPCAS) randomised controlled trial: protocol for a multidimensional process evaluation
Source: BMJ Open. 2020 Jul 8;10(7):e036879. doi: 10.1136/bmjopen-2020-036879 (PMC7348649; doi:10.1136/bmjopen-2020-036879)
Supplement: Supplementary data [file bmjopen-2020-036879supp005.pdf]

Supplementary file 5

**Assessor's name:**					
**Group/facilitator ID:**					
**Date:**					
**Week 1: Group session 1, Stroke Journey (2.5 hours, with breaks)**					
**Section A: Welcome/introduction**					
**Facilitator(s):**					
**Start time:**					
**End time:**					
	**Tick as appropriate**				
	**Present**	**Absent**	**Partially done**	**If *partially done*, please provide reasons (e.g. ran out of time, forgot, deemed unnecessary)**	**Other comments**
Content					
A1) Welcome/introductions (including housekeeping details, course handbook, course background)	☐	☐	☐		
A2) Introduction to the guidelines for working together as a group	☐	☐	☐		
A3) Opportunity for participants to ask questions	☐	☐	☐		
A4) Introduction to communication aids	☐	☐	☐		
A5) Participants' important questions	☐	☐	☐		
Materials					
A6) Flipchart – names	☐	☐	☐		
A7) Flipcharts – what would you like to get from the course?; what is your important question?	☐	☐	☐		
A8) Flipchart – working together as a group	☐	☐	☐		
Key facilitator behaviours for welcome/introduction					
A9) Uses and refers to participants' comments/quotes	☐	☐	☐		

v1.4 29/05/2019

**Section B: Stroke story**					
**Facilitator(s):**					
**Start time:**					
**End time:**					
	**Tick as appropriate**				
	**Present**	**Absent**	**Partially done**	**If *partially done*, please provide reasons (e.g. ran out of time, forgot, deemed unnecessary)**	**Other comments**
Content					
B1) Stroke story/introduction to narrative approach (including roadmap)	☐	☐	☐		
B2) Eliciting skills, strengths and strategies from participants	☐	☐	☐		
Materials					
B3) Flipchart – skills and strategies	☐	☐	☐		
Key facilitator behaviours for stroke story					
B4) Uses resources and/or participant handbooks appropriately to engage participants and enhance their understanding of the session's key outcomes	☐	☐	☐		
B5) Facilitates people to share their stories about attempts to manage their condition	☐	☐	☐		
B6) Appropriately adapts communication/environment/session delivery to meet the needs of the stroke survivors	☐	☐	☐		

**Section C: What is stroke?**					
**Facilitator(s):**					
**Start time:**					
**End time:**					
	**Tick as appropriate**				
	**Present**	**Absent**	**Partially done**	**If *partially done*, please provide reasons (e.g. ran out of time, forgot, deemed unnecessary)**	**Other comments**
Content					
C1) What is stroke (including impacts of stroke on individual – physically, emotionally, psychologically and socially)?	☐	☐	☐		
C2) Discussed neuroplasticity (the brain's ability to create new neural pathways and relearn skills	☐	☐	☐		
Materials					
C3) Flipchart – body outline	☐	☐	☐		
C4) Record of 'what is stroke?' (i.e. written on body outline/sticky notes)	☐	☐	☐		
C5) Record of 'effects of stroke on body/person' (i.e. written on body outline/sticky notes)	☐	☐	☐		
Key facilitator behaviours for what is stroke					
C6) Prompts participant to talk about their thoughts and explores misconceptions and gaps in knowledge	☐	☐	☐		
C7) Uses resources and/or participant handbooks appropriately to engage participants and enhance their understanding of the session's key outcomes	☐	☐	☐		

**Section D: Stroke passenger**					
**Facilitator(s):**					
**Start time:**					
**End time:**					
	**Tick as appropriate**				
	**Present**	**Absent**	**Partially done**	**If *partially done*, please provide reasons (e.g. ran out of time, forgot, deemed unnecessary)**	**Other comments**
Content					
D1) Discussed 'Stroke passenger' analogy	☐	☐	☐		
D2) Eliciting feelings and emotions about stroke	☐	☐	☐		
D3) Provided opportunity for separate carer/stroke survivor discussions	☐	☐	☐		
D4) Discussing skills and coping strategies for living with stroke passenger	☐	☐	☐		
Materials					
D5) Flipchart – how do you feel/think about sharing your life with stroke (carers)?	☐	☐	☐		
D6) Flipchart – how do you feel/think about sharing your life with stroke (stroke survivors)?	☐	☐	☐		
Key facilitator behaviours for stroke passenger					
D7) Prompts participants to express and explore their feelings about the condition or behaviour	☐	☐	☐		
D8) Facilitates people to share their stories about attempts to manage their condition	☐	☐	☐		
D9) Appropriately adapts communication/environment/session delivery to meet the needs of the stroke survivors	☐	☐	☐		
D10) Works as a team with co-facilitator	☐	☐	☐		

v1.4 29/05/2019

**Section E: What will I do now?**					
**Facilitator(s):**					
**Start time:**					
**End time:**					
	**Tick as appropriate**				
	**Present**	**Absent**	**Partially done**	**If *partially done*, please provide reasons (e.g. ran out of time, forgot, deemed unnecessary)**	**Other comments**
Content					
E1) Session summary/recap of session key outcomes	☐	☐	☐		
E2) 'What will I do now?' worksheet	☐	☐	☐		
Materials					
E3) 'What will I do now?' worksheet in handbook	☐	☐	☐		
Key facilitator behaviours for what will I do now					
E4) Uses resources and/or participant handbooks appropriately to engage participants and enhance their understanding of the session's key outcomes	☐	☐	☐		
E5) Prompts group to summarise their key messages from the session.	☐	☐	☐		
E6) Prompts participants to talk about what they are going to do as a result of the session.	☐	☐	☐		

Aquino MRJ(Ryc), *et al. BMJ Open* 2020; 10:e036879. doi: 10.1136/bmjopen-2020-036879

Overall facilitator behaviours					
	**Tick as appropriate**				
	**Present**	**Absent**	**Partially done**	**If *partially done*, please provide reasons (e.g. ran out of time, forgot, deemed unnecessary)**	**Other comments**
1. Uses non-judgmental statements in response to participants' verbal utterances	☐	☐	☐		
2. The facilitator seeks clarification of participants contribution	☐	☐	☐		
3. Avoids giving their own opinion	☐	☐	☐		
4. Encourages the group to discuss/answer their own question	☐	☐	☐		
5. Engages participants using rapport building skills	☐	☐	☐		
6. Notices tone/dynamics within the group, acknowledges these and uses them to manage the group	☐	☐	☐		
7. The facilitator uses strategies to manage time within session	☐	☐	☐		
8. Uses active listening skills and open body language to facilitate group/participant talk	☐	☐	☐		
9. Appropriately adapts delivery to meet the needs of the stroke survivors	☐	☐	☐		
10. Works as a team with co-facilitator	☐	☐	☐		

Aquino MRJ(Ryc), *et al. BMJ Open* 2020; 10:e036879. doi: 10.1136/bmjopen-2020-036879

Assessor comments/notes

Aquino MRJ(Ryc), *et al. BMJ Open* 2020; 10:e036879. doi: 10.1136/bmjopen-2020-036879

**Assessor's name:**					
**Group/facilitator ID:**					
**Date:**					
**Week 2/3 (circle as appropriate): Managing health and wellbeing (2.5 hours, with breaks)**					
**Section A: Welcome back [20 mins]**					
**Facilitator(s):**					
**Start time:**					
**End time:**					
	**Tick as appropriate**				
	**Present**	**Absent**	**Partially done**	**If *partially done*, please provide reasons (e.g. ran out of time, forgot, deemed unnecessary)**	**Other comments**
Content					
A1) Welcome (including housekeeping details, and ground rules)	☐	☐	☐		
A2) Session outline	☐	☐	☐		
A3) Introduction/recap of managing health and wellbeing handbook and roadmap <u>or</u> roadblocks handbook and roadmap	☐	☐	☐		
A4) Previous week's worksheets; stories and reflections from past week	☐	☐	☐		
A5) Opportunity for participants to ask questions	☐	☐	☐		
Materials					
A6) Roadmap	☐	☐	☐		
A7) Previous week's (relevant) flipcharts on view	☐	☐	☐		
Key facilitator behaviours for welcome					
A8) Uses resources and participant handbooks appropriately to engage participants and enhance understanding of the session's key outcomes	☐	☐	☐		
A9) Prompts the participants to reflect on last week's session and activities	☐	☐	☐		

Aquino MRJ(Ryc), *et al. BMJ Open* 2020; 10:e036879. doi: 10.1136/bmjopen-2020-036879

**Section B: Managing health and wellbeing [40 mins]**					
**Facilitator(s):**					
**Start time:**					
**End time:**					
	**Tick as appropriate**				
	**Present**	**Absent**	**Partially done**	**If *partially done*, please provide reasons (e.g. ran out of time, forgot, deemed unnecessary)**	**Other comments**
Content					
B1) Risk factors for stroke	☐	☐	☐		
B2) Ways to manage health and wellbeing	☐	☐	☐		
Materials					
B3) A6 headings: 'risks I can change' and 'risks I cannot change'	☐	☐	☐		
B4) A6 images: risk factors	☐	☐	☐		
B5) A4 headings: health and wellbeing	☐	☐	☐		
B6) A6 images: health and wellbeing	☐	☐	☐		
Key facilitator behaviours for managing health and wellbeing					
B7) Facilitates people to share their stories about attempts to manage their condition	☐	☐	☐		
B8) Uses resources and participant handbooks appropriately to engage participants and enhance understanding of the session's key outcomes	☐	☐	☐		
B9) Prompts participant to talk about their thoughts and explores misconceptions and gaps in knowledge	☐	☐	☐		
B10) Encourages the group to discuss/answer their own questions	☐	☐	☐		
B11) Works as a team with co-facilitator	☐	☐	☐		

**Section C: Making changes [15 mins]**					
**Facilitator(s):**					
**Start time:**					
**End time:**					
	**Tick as appropriate**				
	**Present**	**Absent**	**Partially done**	**If *partially done*, please provide reasons (e.g. ran out of time, forgot, deemed unnecessary)**	**Other comments**
Content					
C1) Elicits feelings and reactions to change	☐	☐	☐		
C2) Discussion of pros and cons of changes	☐	☐	☐		
Materials					
C3) Flipchart – pros and cons grid	☐	☐	☐		
Key facilitator behaviours for making changes					
C4) Prompts participants to discuss their thoughts about possible changes to self-management.	☐	☐	☐		
C5) Prompts participants to review the impact of possible choices on their future health.	☐	☐	☐		
C6) Prompts or allows participants to express and explore their feelings about the condition or behaviour	☐	☐	☐		

**Section D: Making a plan [35 mins]**					
**Facilitator(s):**					
**Start time:**					
**End time:**					
	**Tick as appropriate**				
	**Present**	**Absent**	**Partially done**	**If *partially done*, please provide reasons (e.g. ran out of time, forgot, deemed unnecessary)**	**Other comments**
Content					
D1) Identifying challenges to planning, problem-solving	☐	☐	☐		
D2) Making a SMART plan	☐	☐	☐		
Materials					
D3) A4 resource: ‘My action plan’	☐	☐	☐		
D4) ‘My action plan’ in handbook	☐	☐	☐		
Key facilitator behaviours for making a plan					
D5) Prompts participants to discuss their thoughts about possible changes to self-management.	☐	☐	☐		
D6) Prompts the participants to reflect on their goals/plans	☐	☐	☐		
D7) Prompts the individual or group to problem solve possible barriers to change (e.g. their desired changes or possible barriers to self-management)	☐	☐	☐		
D8) Encourages the group to discuss/answer their own questions	☐	☐	☐		
D9) Prompts group to summarise key messages from the session	☐	☐	☐		

Section E: What will I do now? [10 mins]					
**Facilitator(s):**					
**Start time:**					
**End time:**					
	**Tick as appropriate**				
	**Present**	**Absent**	**Partially done**	**If *partially done*, please provide reasons (e.g. ran out of time, forgot, deemed unnecessary)**	**Other comments**
Content					
E1) Session summary provided	☐	☐	☐		
E2) ‘What will I do now?’ worksheet	☐	☐	☐		
Materials					
E3) ‘What will I do now?’ worksheet in handbook	☐	☐	☐		
Key facilitator behaviours for what will I do now					
E4) Uses resources and participant handbooks appropriately to engage participants and enhance understanding of the session’s key outcomes	☐	☐	☐		
E5) Prompts participants to talk about what they are going to do as a result of the session	☐	☐	☐		
E6) Prompts group to summarise their key messages from the session.	☐	☐	☐		

Aquino MRJ(Ryc), *et al. BMJ Open* 2020; 10:e036879. doi: 10.1136/bmjopen-2020-036879

Overall facilitator behaviours					
	**Tick as appropriate**				
	**Present**	**Absent**	**Partially done**	**If *partially done*, please provide reasons (e.g. ran out of time, forgot, deemed unnecessary)**	**Other comments**
1. Uses non-judgmental statements in response to participants' verbal utterances	☐	☐	☐		
2. The facilitator seeks clarification of participants contribution	☐	☐	☐		
3. Avoids giving their own opinion	☐	☐	☐		
4. Encourages the group to discuss/answer their own questions and/or elicits suggestions from participants	☐	☐	☐		
5. Engages participants using rapport building skills	☐	☐	☐		
6. Notices tone/dynamics within the group, acknowledges these and uses them to manage the group	☐	☐	☐		
7. The facilitator uses strategies to manage time within session	☐	☐	☐		
8. Uses open questions, reflections, appropriate silences and open body language to facilitate group/participant talk	☐	☐	☐		
9. Adapts communication/environment/ session delivery to meet the needs of the stroke survivors	☐	☐	☐		
10. Works as a team with co-facilitator	☐	☐	☐		

Aquino MRJ(Ryc), *et al. BMJ Open* 2020; 10:e036879. doi: 10.1136/bmjopen-2020-036879

Assessor comments/notes

Aquino MRJ(Ryc), *et al. BMJ Open* 2020; 10:e036879. doi: 10.1136/bmjopen-2020-036879

**Assessor's name:**					
**Group/facilitator ID:**					
**Date:**					
Week 2/3 (circle as appropriate): Roadblocks (2.5 hrs, with breaks)					
**Section A: Welcome back**					
**Facilitator(s):**					
**Start time:**					
**End time:**					
	**Tick as appropriate**				
	**Present**	**Absent**	**Partially done**	**If *partially done*, please provide reasons (e.g. ran out of time, forgot, deemed unnecessary)**	**Other comments**
Content					
A1) Welcome (including housekeeping details, and ground rules)	☐	☐	☐		
A2) Session outline	☐	☐	☐		
A3) Introduction/recap of managing health and wellbeing handbook and roadmap <u>or</u> roadblocks handbook and roadmap	☐	☐	☐		
A4) Previous week's worksheets; stories and reflections from past week	☐	☐	☐		
A5) Opportunity for participants to ask questions					
Materials					
A6) Roadmap	☐	☐	☐		
A7) Previous week's (relevant) flipcharts on view	☐	☐	☐		
Key facilitator behaviours for welcome back					
A8) Uses resources and participant handbooks appropriately to engage participants and enhance understanding of the session's key outcomes	☐	☐	☐		
A9) Prompts the participants to reflect on last week's session and activities	☐	☐	☐		

Aquino MRJ(Ryc), *et al. BMJ Open* 2020; 10:e036879. doi: 10.1136/bmjopen-2020-036879

**Section B: Roadblocks on my journey**					
**Facilitator(s):**					
**Start time:**					
**End time:**					
	**Tick as appropriate**				
	**Present**	**Absent**	**Partially done**	**If *partially done*, please provide reasons (e.g. ran out of time, forgot, deemed unnecessary)**	**Other comments**
Content					
B1) Fictional character facing loss of confidence after stroke (using STOP sign)	☐	☐	☐		
B2) Eliciting thoughts, feelings, sensations, or behaviours as a response to loss of confidence	☐	☐	☐		
B3) Discuss what might help the fictional character, and impact of this on thoughts, feelings, sensations, or behaviours (using GO sign)	☐	☐	☐		
B4) ‘What do my roadblocks look like?’ worksheet	☐	☐	☐		
Materials					
B5) Roadmap	☐	☐	☐		
B6) Flipcharts – STOP and GO	☐	☐	☐		

Key facilitator behaviours for roadblocks					
	**Tick as appropriate**				
	**Present**	**Absent**	**Partially done**	**If *partially done*, please provide reasons (e.g. ran out of time, forgot, deemed unnecessary)**	**Other comments**
B7) Facilitates people to share their stories about attempts to manage their condition, and the challenges/roadblocks they face.	☐	☐	☐		
B8) Prompts or allows participants to express and explore their feelings about (the condition or behaviour) during the session	☐	☐	☐		
B9) Encourages the group to discuss/answer their own questions	☐	☐	☐		
B10) Uses and refers to participants' comments/quotes	☐	☐	☐		
B11) Prompts the individual or group to problem solve possible barriers to change (e.g. their desired changes or possible barriers to self-management)	☐	☐	☐		
B12) Prompts participants to review the impact of possible choices on their future health.	☐	☐	☐		

Aquino MRJ(Ryc), *et al. BMJ Open* 2020; 10:e036879. doi: 10.1136/bmjopen-2020-036879

**Section C: Working it out**					
**Facilitator(s):**					
**Start time:**					
**End time:**					
	**Tick as appropriate**				
	**Present**	**Absent**	**Partially done**	**If *partially done*, please provide reasons (e.g. ran out of time, forgot, deemed unnecessary)**	**Other comments**
Content					
C1) Problems stroke survivors could face Next steps (including session's key outcomes, 'what will I do now?' worksheet, and follow-up appointments)	☐	☐	☐		
C2) Introduce problem-solving approach	☐	☐	☐		
C3) Use fictional character to choose a problem and apply problem-solving approach	☐	☐	☐		
C4) How decisions may be made on the best course of action for solving the problem	☐	☐	☐		
C5) What to do when problems aren't solvable now	☐	☐	☐		
C6) 'Working it out' worksheet	☐	☐	☐		
Materials					
C7) Flipcharts with headings: 1) in my power to solve/change or not in my power to solve/change; 2) 'what could help'; 3) solution: immediate results/long-term results	☐	☐	☐		
C8) Previous flipcharts (e.g. what are my roadblocks; skills and strategies toolkit)	☐	☐	☐		

Key facilitator behaviours for working it out					
	**Tick as appropriate**				
	**Present**	**Absent**	**Partially done**	**If *partially done*, please provide reasons (e.g. ran out of time, forgot, deemed unnecessary)**	**Other comments**
C9) Uses resources and participant handbooks appropriately to engage participants and enhance understanding of the session's key outcomes	☐	☐	☐		
C10) Uses and refers to participants' comments/quotes	☐	☐	☐		
C11) Prompts participants to discuss their thoughts about possible changes to self-management.	☐	☐	☐		
C12) Prompts participants to review the impact of possible choices on their future health.	☐	☐	☐		
C13) Prompts the individual or group to problem solve possible barriers to change (e.g. their desired changes or possible barriers to self-management)	☐	☐	☐		

Aquino MRJ(Ryc), *et al. BMJ Open* 2020; 10:e036879. doi: 10.1136/bmjopen-2020-036879

**Section D: What will I do now**					
**Facilitator(s):**					
**Start time:**					
**End time:**					
	**Tick as appropriate**				
	**Present**	**Absent**	**Partially done**	**If *partially done*, please provide reasons (e.g. ran out of time, forgot, deemed unnecessary)**	**Other comments**
Content					
D1) Recap key outcomes	☐	☐	☐		
D2) 'What will I do now?' worksheet	☐	☐	☐		
Materials					
D3) 'What will I do now?' worksheet	☐	☐	☐		
Key facilitator behaviours for what will I do now					
D4) Uses resources and participant handbooks appropriately to engage participants and enhance understanding of the session's key outcomes	☐	☐	☐		
D5) Prompts participants to talk about what they are going to do as a result of the session.	☐	☐	☐		

Overall facilitator behaviours					
	**Tick as appropriate**				
	**Present**	**Absent**	**Partially done**	**If *partially done*, please provide reasons (e.g. ran out of time, forgot, deemed unnecessary)**	**Other comments**
1. Prompts group to summarise their key messages from the session.	☐	☐	☐		
2. The facilitator seeks clarification of participants contribution	☐	☐	☐		
3. Avoids giving their own opinion	☐	☐	☐		
4. Encourages the group to discuss/answer their own questions and/or elicits suggestions from participants	☐	☐	☐		
5. Engages participants using rapport building skills	☐	☐	☐		
6. Notices tone/dynamics within the group, acknowledges these and uses them to manage the group	☐	☐	☐		
7. The facilitator uses strategies to manage time within session	☐	☐	☐		
8. Uses open questions, reflections, appropriate silences and open body language to facilitate group/participant talk	☐	☐	☐		
9. Adapts communication/environment/ session delivery to meet the needs of the stroke survivors	☐	☐	☐		
10. Works as a team with co-facilitator	☐	☐	☐		

Aquino MRJ(Ryc), *et al. BMJ Open* 2020; 10:e036879. doi: 10.1136/bmjopen-2020-036879

Assessor comments/notes

Aquino MRJ(Ryc), *et al. BMJ Open* 2020; 10:e036879. doi: 10.1136/bmjopen-2020-036879

**Assessor's name:**					
**Group/facilitator ID:**					
**Date:**					
**Week 4: Moving forward on my journey (2.5 hours, with breaks)**					
**Section A: Welcome back**					
**Facilitator(s):**					
**Start time:**					
**End time:**					
	**Tick as appropriate**				
	**Present**	**Absent**	**Partially done**	**If *partially done*, please provide reasons (e.g. ran out of time, forgot, deemed unnecessary)**	**Other comments**
Content					
A1) Welcome (including housekeeping details, handbook and ground rules)	☐	☐	☐		
A2) Session outline	☐	☐	☐		
A3) Previous week's action plan/working it out worksheets	☐	☐	☐		
A4) Recap roadmap	☐	☐	☐		
Materials					
A5) Roadmap	☐	☐	☐		
A6) Previous week's (relevant) flipcharts on view	☐	☐	☐		
A7) Moving forward on my journey handbook	☐	☐	☐		
Key facilitator behaviours for welcome back					
A8) Re-engages participants in the journey analogy and resources	☐	☐	☐		
A9) Prompts the participants to reflect on last week's session and activities	☐	☐	☐		

**Section B: Your relationships**					
**Facilitator(s):**					
**Start time:**					
**End time:**					
	**Tick as appropriate**				
	**Present**	**Absent**	**Partially done**	**If *partially done*, please provide reasons (e.g. ran out of time, forgot, deemed unnecessary)**	**Other comments**
Content					
B1) Ways relationships can be affected by stroke	☐	☐	☐		
B2) Problem-solving exercise in relation to a relationship change	☐	☐	☐		
Materials					
B3) Flipcharts – how can relationships change (one for stroke survivors, one for carers/supporters as applicable)	☐	☐	☐		
B4) Flipcharts – ‘what is the problem exactly’; ‘what could help’; ‘solution: immediate results/long-term results’	☐	☐	☐		

Aquino MRJ(Ryc), *et al. BMJ Open* 2020; 10:e036879. doi: 10.1136/bmjopen-2020-036879

Key facilitator behaviours for your relationships					
	**Tick as appropriate**				
	**Present**	**Absent**	**Partially done**	**If *partially done*, please provide reasons (e.g. ran out of time, forgot, deemed unnecessary)**	**Other comments**
B5) Prompts or allows participants to express and explore their feelings about (the condition or behaviour) during the session	☐	☐	☐		
B6) Uses and refers to participants' comments/quotes	☐	☐	☐		
B7) Prompts the individual or group to problem solve possible barriers to change (e.g. their desired changes or possible barriers to self-management)	☐	☐	☐		
B8) Prompts participants to review the impact of possible choices on their future health.	☐	☐	☐		
B9) Adapts communication, environment, or session delivery to meet the needs of the stroke survivors	☐	☐	☐		

Aquino MRJ(Ryc), *et al. BMJ Open* 2020; 10:e036879. doi: 10.1136/bmjopen-2020-036879

**Section C: Revisiting your stroke journey**					
**Facilitator(s):**					
**Start time:**					
**End time:**					
	**Tick as appropriate**				
	**Present**	**Absent**	**Partially done**	**If *partially done*, please provide reasons (e.g. ran out of time, forgot, deemed unnecessary)**	**Other comments**
Content					
C1) Recap the roadmap/journey analogy	☐	☐	☐		
C2) Summary of key messages from each session (e.g. session outcomes/personal take-home messages/skills and strategies discussed during the course)	☐	☐	☐		
C3) Elicit feelings about moving forward from MLAS	☐	☐	☐		
Materials					
C4) Flipchart – 'skills and strategies toolkit'	☐	☐	☐		
Key facilitator behaviours for revisiting your stroke journey					
C5) Facilitates the group's recapping of the journey analogy (e.g. prompts group to summarise their key messages from the session)	☐	☐	☐		
C6) Prompts participants to reflect on where they feel they are now	☐	☐	☐		

**Section D: How will you move forward?**					
**Facilitator(s):**					
**Start time:**					
**End time:**					
	**Tick as appropriate**				
	**Present**	**Absent**	**Partially done**	**If *partially done*, please provide reasons (e.g. ran out of time, forgot, deemed unnecessary)**	**Other comments**
Content					
D1) Discussion of 'warning lights' for physical/emotional health and prevention of these	☐	☐	☐		
D2) Loneliness from the perspective of the fictional character (may also use problem-solving exercise)	☐	☐	☐		
D3) The directory	☐	☐	☐		
Materials					
D4) A4 dashboard images	☐	☐	☐		
D5) Flipchart - 'Skills and strategies toolkit'	☐	☐	☐		
D6) A4 directory	☐	☐	☐		
Key facilitator behaviours for how will you move forward					
D7) Prompts or allows participants to express and explore their feelings about the condition or behaviour	☐	☐	☐		
D8) Encourages the group to discuss/answer their own questions	☐	☐	☐		
D9) Uses and refers to participants' comments/quotes	☐	☐	☐		

**Section E: Next steps**					
**Facilitator(s):**					
**Start time:**					
**End time:**					
	**Tick as appropriate**				
	**Present**	**Absent**	**Partially done**	**If *partially done*, please provide reasons (e.g. ran out of time, forgot, deemed unnecessary)**	**Other comments**
Content					
E1) Recap session's key outcomes	☐	☐	☐		
E2) 'What will I do now?' worksheet	☐	☐	☐		
E3) Organise follow-up appointments	☐	☐	☐		
Materials					
E4) 'What will I do now?' worksheet in the handbook	☐	☐	☐		
E5) Flipchart – 'what would you like to get from the course?/What is your important question?'	☐	☐	☐		
Key facilitator behaviours for next steps					
E6) Prompts group to summarise their key messages from the session.	☐	☐	☐		
E7) Prompts participants to talk about what they are going to do as a result of the course.	☐	☐	☐		
E8) Encourages the group to discuss/answer their own questions	☐	☐	☐		

Aquino MRJ(Ryc), *et al. BMJ Open* 2020; 10:e036879. doi: 10.1136/bmjopen-2020-036879

Overall facilitator behaviours					
	**Tick as appropriate**				
	**Present**	**Absent**	**Partially done**	**If *partially done*, please provide reasons (e.g. ran out of time, forgot, deemed unnecessary)**	**Other comments**
1. Uses non-judgmental statements in response to participants' verbal utterances	☐	☐	☐		
2. The facilitator seeks clarification of participants contribution	☐	☐	☐		
3. Avoids giving their own opinion	☐	☐	☐		
4. Encourages the group to discuss/answer their own questions and/or elicits suggestions from participants	☐	☐	☐		
5. Engages participants using rapport building skills	☐	☐	☐		
6. Notices tone/dynamics within the group, acknowledges these and uses them to manage the group	☐	☐	☐		
7. The facilitator uses strategies to manage time within session	☐	☐	☐		
8. Uses open questions, reflections, appropriate silences and open body language to facilitate group/participant talk	☐	☐	☐		
9. Adapts communication/environment/ session delivery to meet the needs of the stroke survivors	☐	☐	☐		
10. Works as a team with co-facilitator	☐	☐	☐		

Aquino MRJ(Ryc), *et al. BMJ Open* 2020; 10:e036879. doi: 10.1136/bmjopen-2020-036879

Assessor comments/notes

Aquino MRJ(Ryc), *et al. BMJ Open* 2020; 10:e036879. doi: 10.1136/bmjopen-2020-036879
