# Supplementary material for: Improving Primary Care After Stroke (IPCAS) randomised controlled trial: protocol for a multidimensional process evaluation
Source: BMJ Open. 2020 Jul 8;10(7):e036879. doi: 10.1136/bmjopen-2020-036879 (PMC7348649; doi:10.1136/bmjopen-2020-036879)
Supplement: Supplementary data [file bmjopen-2020-036879supp006.pdf]

Supplementary file 6

IPCAS trial process evaluation checklist
MLAS facilitator delivery - DESMOND Observation Tool Record Sheet
Group Session 1

Instructions: DOT at 10s (i.e. at the second bleep)

Session A: Welcome/Introduction (30 min)

Facilitator's name:

Assessor's name:

Start time:

End time:

Facilitator talk	Participant talk	Other
Total A:	Total B:	Total C:
Total score= A÷(A+B+C)*100=		

Session B: Stroke story (20 min)

Facilitator's name:

Assessor's name:

Start time:

End time:

Facilitator talk	Participant talk	Other
Total A:	Total B:	Total C:
Total score= A÷(A+B+C)*100=		

Aquino MRJ(Ryc), *et al. BMJ Open* 2020; 10:e036879. doi: 10.1136/bmjopen-2020-036879

IPCAS trial process evaluation checklist

Session C: What is stroke? (25 min)

Facilitator's name:

Assessor's name:

Start time:

End time:

Facilitator talk	Participant talk	Other
Total A:	Total B:	Total C:
Total score= A÷(A+B+C)*100=		

Session D: Stroke passenger (35 min)

Facilitator's name:

Assessor's name:

Start time:

End time:

Facilitator talk	Participant talk	Other
Total A:	Total B:	Total C:
Total score= A÷(A+B+C)*100=		

Session E: What will I do now? (10 min)

Facilitator

Start time:

End time:

Facilitator talk	Participant talk	Other

Aquino MRJ(Ryc), *et al. BMJ Open* 2020; 10:e036879. doi: 10.1136/bmjopen-2020-036879

IPCAS trial process evaluation checklist

Total A:	Total B:	Total C:
Total score= A÷(A+B+C)*100=		

Aquino MRJ(Ryc), *et al. BMJ Open* 2020; 10:e036879. doi: 10.1136/bmjopen-2020-036879

IPCAS trial process evaluation checklist
MLAS facilitator delivery - DESMOND Observation Tool Record Sheet
Group Session 2/3 (circle as appropriate): Managing Health and Wellbeing

Instructions: DOT at 10s (i.e. at the second bleep)

Session A: Welcome back, recap, and introduction (20 min)

Facilitator's name:
Assessor's name:
Start time:
End time:

Facilitator talk	Participant talk	Other
Total A:	Total B:	Total C:
Total score= A÷(A+B+C)*100=		

Session B: Managing health and wellbeing (40 min)

Facilitator's name:
Assessor's name:
Start time:
End time:

Facilitator talk	Participant talk	Other
Total A:	Total B:	Total C:
Total score= A÷(A+B+C)*100=		

Aquino MRJ(Ryc), *et al. BMJ Open* 2020; 10:e036879. doi: 10.1136/bmjopen-2020-036879

IPCAS trial process evaluation checklist

Session C: Making changes (15 min)

Facilitator's name:

Assessor's name:

Start time:

End time:

Facilitator talk	Participant talk	Other
Total A:	Total B:	Total C:
Total score= A÷(A+B+C)*100=		

Session D: Making a plan (35 min)

Facilitator's name:

Assessor's name:

Start time:

End time:

Facilitator talk	Participant talk	Other
Total A:	Total B:	Total C:
Total score= A÷(A+B+C)*100=		

Session E: What will I do now? (10 min)

Facilitator

Start time:

End time:

Facilitator talk	Participant talk	Other

Aquino MRJ(Ryc), *et al. BMJ Open* 2020; 10:e036879. doi: 10.1136/bmjopen-2020-036879

IPCAS trial process evaluation checklist

Total A:	Total B:	Total C:
Total score= A÷(A+B+C)*100=		

Aquino MRJ(Ryc), *et al. BMJ Open* 2020; 10:e036879. doi: 10.1136/bmjopen-2020-036879

IPCAS trial process evaluation checklist
MLAS facilitator delivery - DESMOND Observation Tool Record Sheet
Group Session 2/3 (circle as appropriate): Roadblocks

Instructions: DOT at 10s (i.e. at the second bleep)

Session A: Welcome back and introduction (20 min)

Facilitator's name:
Assessor's name:
Start time:
End time:

Facilitator talk	Participant talk	Other
Total A:	Total B:	Total C:
Total score= A÷(A+B+C)*100=		

Session B: Roadblocks on my journey (45 min)

Facilitator's name:
Assessor's name:
Start time:
End time:

Facilitator talk	Participant talk	Other
Total A:	Total B:	Total C:
Total score= A÷(A+B+C)*100=		

Aquino MRJ(Ryc), *et al. BMJ Open* 2020; 10:e036879. doi: 10.1136/bmjopen-2020-036879

IPCAS trial process evaluation checklist

Session C: Working it out (45 min)

Facilitator's name:

Assessor's name:

Start time:

End time:

Facilitator talk	Participant talk	Other
Total A:	Total B:	Total C:
Total score= A÷(A+B+C)*100=		

Session D: What will I do now? (10 min)

Facilitator's name:

Assessor's name:

Start time:

End time:

Facilitator talk	Participant talk	Other
Total A:	Total B:	Total C:
Total score= A÷(A+B+C)*100=		

IPCAS trial process evaluation checklist
MLAS facilitator delivery - DESMOND Observation Tool Record Sheet
Group Session 4: Moving Forward on my Journey

Instructions: DOT at 10s (i.e. at the second bleep)

Session A: Welcome back, recap, and introduction (20 min)

Facilitator's name:
Assessor's name:
Start time:
End time:

Facilitator talk	Participant talk	Other
Total A:	Total B:	Total C:
Total score= A÷(A+B+C)*100=		

Session B: Your relationships (45 min)

Facilitator's name:
Assessor's name:
Start time:
End time:

Facilitator talk	Participant talk	Other
Total A:	Total B:	Total C:
Total score= A÷(A+B+C)*100=		

Aquino MRJ(Ryc), *et al. BMJ Open* 2020; 10:e036879. doi: 10.1136/bmjopen-2020-036879

IPCAS trial process evaluation checklist

Session C: Revisiting your stroke journey (10 min)

Facilitator's name:

Assessor's name:

Start time:

End time:

Facilitator talk	Participant talk	Other
Total A:	Total B:	Total C:
Total score= A÷(A+B+C)*100=		

Session D: How will you move forward? (30 min)

Facilitator's name:

Assessor's name:

Start time:

End time:

Facilitator talk	Participant talk	Other
Total A:	Total B:	Total C:
Total score= A÷(A+B+C)*100=		

Session E: What will I do now? (10 min)

Facilitator

Start time:

End time:

Facilitator talk	Participant talk	Other

Aquino MRJ(Ryc), *et al. BMJ Open* 2020; 10:e036879. doi: 10.1136/bmjopen-2020-036879

IPCAS trial process evaluation checklist

Total A:	Total B:	Total C:
Total score= A÷(A+B+C)*100=		

Aquino MRJ(Ryc), *et al. BMJ Open* 2020; 10:e036879. doi: 10.1136/bmjopen-2020-036879
