# Supplementary material for: Improving Primary Care After Stroke (IPCAS) randomised controlled trial: protocol for a multidimensional process evaluation
Source: BMJ Open. 2020 Jul 8;10(7):e036879. doi: 10.1136/bmjopen-2020-036879 (PMC7348649; doi:10.1136/bmjopen-2020-036879)
Supplement: Supplementary data [file bmjopen-2020-036879supp007.pdf]

Supplementary file 7

IPCAS trial patient process evaluation/fidelity checklist - patient							
Site ID:			Participant ID:			Date:	
Time of call:			Conducted by:			Duration of call:	
Activity			Tick as appropriate			If *no* or *unsure*, please provide reasons why/any details	Other comments
			Yes	No	Unsure		
Structured review (attending at the GP surgery)	1. Attended structured review (If no/unsure, ask for reasons why)						
	2. 15-item checklist of needs (If no/unsure, ask for reasons why)	a) Patient completed checklist					
		b) Discussion of up to 3 needs (as ranked by importance by patient) *(Can you tell me which needs you discussed?)*					
	4. Development of action plan(s) (If no/unsure, ask for reasons why)	a) Discussed action plan *(Did you talk about how to address your needs?)*					
		b) Opportunity to log and review agreed actions *(Did you agree a specific plan going forward to address these needs? E.g., book an appointment with physio on Monday)*					
	3. Physical check (If no/unsure, ask for reasons why)	a) Blood pressure					
		b) Medication review *(Did you discuss your medication(s)?)*					
		c) Other (specify) *(Was there anything else that you talked about regarding your physical health?)*					
	5. MLAS (If no/unsure, ask for reasons why)	a) Received leaflet about *MLAS* *(Would you like me to post you one if not?)*					
		b) Received instructions for accessing MLAS (they're on the bottom of the information sheet: "..call Emily…")					
	6. Direct point of contact to stroke survivor and carer(s)	a) Received information about 'direct point of contact' service *(Were you given information about a service in your GP practice where you can get in touch if you have any stroke-related needs/concerns?)*					
		b) Received instructions on how to contact practice at the end of review *(Did you receive instructions about how to access this telephone service?) IF 'NO', FINISH.*					
		TOTAL					
		PERCENTAGE					
Use of direct point of contact (i.e. calling the practice about their stroke-specific needs)	1. Called practice	a) Called practice *(Since your appointment, have you phoned the practice about your stroke? If no/unsure, ask for reasons why. After asking for reasons why, end assessment if they didn't contact the practice)*					
		b) Received signposts to specialist service *(When you called, did you receive advice about accessing any specialist healthcare professionals or services (e.g. physio, speech and language therapist)? If no/unsure, ask for reasons why)*					
		c) Received signposts to community service *(When you called, did you receive advice about accessing any services in your community (e.g. leisure services/local activity groups, GP/nurse appointment)? If no/unsure, ask*					
	2. Receive support and advice	a) Received advice for stroke-specific issues *(that you raised?)*					
		b) Other brief telephone support *(Was there anything else that you talked about that you received support for when you phoned?)*					
	3. Follow-up appointment(s) arranged for						
	4. Case management *(Finally, was there anything else that was given to you during this call? For example, any further support about how to address your needs, any agreed plans for going forward/addressing your needs?)*						
	TOTAL						
	PERCENTAGE						
END OF CHECKLIST							
