# Supplementary material for: Improving Primary Care After Stroke (IPCAS) randomised controlled trial: protocol for a multidimensional process evaluation
Source: BMJ Open. 2020 Jul 8;10(7):e036879. doi: 10.1136/bmjopen-2020-036879 (PMC7348649; doi:10.1136/bmjopen-2020-036879)
Supplement: Supplementary data [file bmjopen-2020-036879supp008.pdf]

**Supplementary file 8**

Thank you for attending **Individual appointment 1**.

Please tick (✓) **agree, disagree, don't know** or **does not apply** for each of the statements below.

| | Agree | Disagree | Don't know | Does not apply |
|---|---|---|---|---|
| 1. The facilitator explained **'My life after stroke'** to me. | | | | |
| 2. The facilitator invited me to **ask questions** about 'My life after stroke'. | | | | |
| 3. I understand what **'My life after stroke'** is. | | | | |
| 4. I felt I could talk about what life was like **before and after** my stroke. | | | | |
| 5. The facilitator invited me to share any **problems I might face** in attending the course. | | | | |
| 6. I had the chance to talk about **ways to address** the problems I might face in attending the course. | | | | |
| 7. The facilitator invited my carer to come to my appointment. | | | | |
| 8. The facilitator invited my carer to **share experiences** if they wanted to. | | | | |

**Please turn over.**

Improving primary care after stroke (IPCAS): A cluster randomised controlled trial
MLAS Patient Receipt Checklist – Individual Appointment 1
IRAS Number: 233891

v.1.0 21-06-18

Please write your **comments** below:

Improving primary care after stroke (IPCAS): A cluster randomised controlled trial
MLAS Patient Receipt Checklist – Individual Appointment 1
IRAS Number: 233891

v.1.0 21-06-18

Thank you for attending **Stroke Journey**.

Please tick (✓) **agree, disagree, don't know** or **does not apply** for each of the statements below.

| | **Agree** | **Disagree** | **Don't know** | **Does not apply** |
|---|---|---|---|---|
| 1. I felt like I could contribute to the session if I wanted to. | | | | |
| 2. I felt like I could participate in the session activities. | | | | |
| 3. The session included a discussion about my **stroke story**. | | | | |
| 4. I had a chance to think about where I am on my **journey** since my stroke. | | | | |
| 5. I know what a stroke is. | | | | |
| 6. I know how a stroke can affect me. | | | | |
| 7. I understand what **neuroplasticity** is. | | | | |
| 8. I have a **strategy** that can help me deal with the effects of my stroke. | | | | |
| 9. The session included a discussion about **feelings** after having a stroke. | | | | |

**Please turn over.**

Improving primary care after stroke (IPCAS): A cluster randomised controlled trial
MLAS Patient Receipt Checklist – Group 1
IRAS Number: 233891
v.1.0 21-06-18

Please tick ✓ **agree**, **disagree**, **don't know** or **does not apply** for each of the statements below.

| | Agree | Disagree | Don't know | Does not apply |
|---|---|---|---|---|
| 10. I am aware of the **barriers** to moving forward with my life after stroke. | | | | |
| 11. I had a chance to talk to other stroke survivors **without my carer**. | | | | |
| 12. I had a chance to complete the '**what will I do now?'** worksheet. | | | | |
| 13. I know what I am going to think about doing differently from today. | | | | |
| 14. I received my **'Stroke journey'** handbook. | | | | |

Please write your **comments** below:

| |
|---|
| |

Improving primary care after stroke (IPCAS): A cluster randomised controlled trial
MLAS Patient Receipt Checklist – Group 1
IRAS Number: 233891 v.1.0 21-06-18

Thank you for attending **Managing health and wellbeing**.

Please tick (✓) **agree, disagree, don't know** or **does not apply** for each of the statements below.

| | Agree | Disagree | Don't know | Does not apply |
|---|---|---|---|---|
| 1. I felt like I could contribute to the session if I wanted to. | | | | |
| 2. I felt like I could participate in the session activities. | | | | |
| 3. The session included an activity on **risk factors for stroke**. | | | | |
| 4. I am aware of what my risks are for having another stroke. | | | | |
| 5. I know what I could do to help **reduce** my risk of having another stroke. | | | | |
| 6. I know what I could do to **stay healthy**. | | | | |
| 7. I understand the **pros and cons** to making change. | | | | |
| 8. I made an **action plan**. | | | | |
| 9. I am confident I can achieve my plan. | | | | |

**Please turn over.**

Improving primary care after stroke (IPCAS): A cluster randomised controlled trial
MLAS Patient Receipt Checklist – Group 2
IRAS Number: 233891 v.1.0 21-06-18

Please tick ✓ **agree**, **disagree**, **don't know** or **does not apply** for each of the statements below.

| | Agree | Disagree | Don't know | Does not apply |
|---|---|---|---|---|
| 10. There were opportunities to **ask questions** at the session. | | | | |
| 11. I was able to learn from other people in the group. | | | | |
| 12. I had a chance to complete the **'what will I do now?'** worksheet. | | | | |
| 13. I know what I am going to think about doing differently from today. | | | | |
| 14. I received my **'Managing health and wellbeing'** handbook. | | | | |

Please write your **comments** below:

| |
|---|
| |

Improving primary care after stroke (IPCAS): A cluster randomised controlled trial
MLAS Patient Receipt Checklist – Group 2
IRAS Number: 233891
v.1.0 21-06-18

Thank you for attending **Roadblocks**.

Please tick (✓) **agree, disagree, don't know** or **does not apply** for each of the statements below.

| | **Agree** | **Disagree** | **Don't know** | **Does not apply** |
|---|---|---|---|---|
| 1. I felt like I could contribute to the session if I wanted to. | | | | |
| 2. I felt like I could participate in the session activities. | | | | |
| 3. The group discussed the link between **thoughts, feelings, physical sensations, and behaviour**. | | | | |
| 4. The group discussed what problems or **'roadblocks'** people might face after a stroke. | | | | |
| 5. I had a chance to complete **'what are my roadblocks?'** worksheet. | | | | |
| 6. The session included a **problem-solving** activity. | | | | |
| 7. The facilitators invited me to share my problems and feelings. | | | | |
| 8. I am able to think about my own problems or roadblocks. | | | | |
| 9. I am able to think about how to solve or manage my own problems or roadblocks. | | | | |

**Please turn over.**

Improving primary care after stroke (IPCAS): A cluster randomised controlled trial
MLAS Patient Receipt Checklist – Group 3
IRAS Number: 233891 v.1.0 21-06-18

Please tick ✓ **agree**, **disagree**, **don't know** or **does not apply** for each of the statements below.

| | Agree | Disagree | Don't know | Does not apply |
|---|---|---|---|---|
| 10. There were opportunities to **ask questions** at the session. | | | | |
| 11. I was able to learn from other people in the group. | | | | |
| 12. I had a chance to complete the '**what will I do now?'** worksheet. | | | | |
| 13. I know what I am going to think about doing differently from today. | | | | |
| 14. I received my **'Roadblocks'** handbook. | | | | |

Please write your **comments** below:

| |
|---|
| |

Improving primary care after stroke (IPCAS): A cluster randomised controlled trial
MLAS Patient Receipt Checklist – Group 3
IRAS Number: 233891
v.1.0 21-06-18

Thank you for attending **'Moving forward on my journey'**.

Please tick (✓) **agree, disagree, don't know** or **does not apply** for each of the statements below.

| | Agree | Disagree | Don't know | Does not apply |
|---|---|---|---|---|
| 1. I felt like I could contribute to the session if I wanted to. | | | | |
| 2. I felt like I could participate in the session activities. | | | | |
| 3. I was able to think about how my relationships have changed since having had my stroke. | | | | |
| 4. The facilitators invited me to share my experiences of changing relationships. | | | | |
| 5. The session included a **problem-solving** activity. | | | | |
| 6. I had a chance to talk to other stroke survivors **without my carer**. | | | | |
| 7. I had a chance to think about where I am on my **journey** since my stroke. | | | | |
| 8. I am aware of how I can keep track of my **physical and mental health**. | | | | |
| 9. I know what I could do to **stay healthy**. | | | | |

**Please turn over.**

Improving primary care after stroke (IPCAS): A cluster randomised controlled trial
MLAS Patient Receipt Checklist – Group 4
IRAS Number: 233891

v.1.0 21-06-18

Please tick ✓ **agree**, **disagree**, **don’t know** or **does not apply** for each of the statements below.

| | Agree | Disagree | Don’t know | Does not apply |
|---|---|---|---|---|
| 10. There were opportunities to **ask questions** at the session. | | | | |
| 11. The group discussed ways to get **support or information**. | | | | |
| 12. I had a chance to complete the ‘**what will I do now?’** worksheet. | | | | |
| 13. I know what I am going to think about doing differently from today. | | | | |
| 14. I received my **‘Moving forward on my journey’** handbook. | | | | |
| 15. I received my **‘Directory’**. | | | | |

Please write your **comments** below:

| |
|---|
| |

Improving primary care after stroke (IPCAS): A cluster randomised controlled trial
MLAS Patient Receipt Checklist – Group 4
IRAS Number: 233891
v.1.0 21-06-18

Thank you for attending **Individual appointment 2**.

Please tick (✓) **agree, disagree, don't know** or **does not apply** for each of the statements below.

| | **Agree** | **Disagree** | **Don't know** | **Does not apply** |
|---|---|---|---|---|
| 1. I was able to reflect on where I am on my **journey** after stroke. | | | | |
| 2. The facilitator invited me to talk about my experience of the **'My life after stroke'** course. | | | | |
| 3. The facilitator invited me to complete the **'Where am I on my journey?'** worksheet. | | | | |
| 4. I was able to **ask questions** if I wanted to. | | | | |
| 5. The facilitator and I talked about my **plans** after 'My life after stroke'. | | | | |
| 6. The facilitator checked if I had any questions. | | | | |
| 7. The appointment included **information about services** that I can access. | | | | |

**Please turn over.**

Improving primary care after stroke (IPCAS): A cluster randomised controlled trial
MLAS Patient Receipt Checklist – Individual Appointment 2
IRAS Number: 233891
v.1.0 21-06-18

Please tick ✓ **agree**, **disagree**, **don’t know** or **does not apply** for each of the statements below.

| | Agree | Disagree | Don’t know | Does not apply |
|---|---|---|---|---|
| 8. My carer was invited to come to my appointment. | | | | |
| 9. The facilitator invited my carer to **share experiences** if they wanted to. | | | | |

Please write your **comments** below:

| |
|---|
| |

Improving primary care after stroke (IPCAS): A cluster randomised controlled trial
MLAS Patient Receipt Checklist – Individual Appointment 2
IRAS Number: 233891

v.1.0 21-06-18
