# Supplementary material for: Improving Primary Care After Stroke (IPCAS) randomised controlled trial: protocol for a multidimensional process evaluation
Source: BMJ Open. 2020 Jul 8;10(7):e036879. doi: 10.1136/bmjopen-2020-036879 (PMC7348649; doi:10.1136/bmjopen-2020-036879)
Supplement: Author's manuscript [file bmjopen-2020-036879.draft_revisions.pdf]

BMJ Open is committed to open peer review. As part of this commitment we make the peer review history of every article we publish publicly available.

When an article is published we post the peer reviewers' comments and the authors' responses online. We also post the versions of the paper that were used during peer review. These are the versions that the peer review comments apply to.

The versions of the paper that follow are the versions that were submitted during the peer review process. They are not the versions of record or the final published versions. They should not be cited or distributed as the published version of this manuscript.

BMJ Open is an open access journal and the full, final, typeset and author-corrected version of record of the manuscript is available on our site with no access controls, subscription charges or pay-per-view fees (http://bmjopen.bmj.com).

If you have any questions on BMJ Open's open peer review process please email info.bmjopen@bmj.com

BMJ Open

Protocol for a multidimensional process evaluation of the Improving Primary Care After Stroke (IPCAS) randomised controlled trial

Journal:	*BMJ Open*
Manuscript ID	bmjopen-2020-036879
Article Type:	Protocol
Date Submitted by the Author:	08-Jan-2020

Complete List of Authors:	Aquino, Maria Raisa Jessica (Ryc); University of Cambridge, Department of Public Health and Primary Care<br>Mullis, Ricky; University of Cambridge, Department of Public Health and Primary Care<br>Kreit, Elizabeth; University of Cambridge, General Practice & Primary Care Research Centre<br>Johnson, Vicki; Univ Hosp Leicester NHS Trust, Leicester Diabetees Centre<br>Grant, Julie; University of Cambridge, Department of Public Health and Primary Care<br>Lim, Lisa; University of Cambridge, Department of Public Health and Primary Care<br>Sutton, Stephen; University of Cambridge,<br>Mant, Jonathan; University of Cambridge, General Practice and Primary Care Research Unit

Keywords:	Protocols & guidelines < HEALTH SERVICES ADMINISTRATION & MANAGEMENT, Clinical trials < THERAPEUTICS, PRIMARY CARE, Stroke < NEUROLOGY

Note: The following files were submitted by the author for peer review, but cannot be converted to PDF. You must view these files (e.g. movies) online.

Supplementary file 4_2020_01.xlsm
Supplementary file 7_2020_01.xlsm

SCHOLARONE™ Manuscripts

I, the Submitting Author has the right to grant and does grant on behalf of all authors of the Work (as defined in the below author licence), an exclusive licence and/or a non-exclusive licence for contributions from authors who are: i) UK Crown employees; ii) where BMJ has agreed a CC-BY licence shall apply, and/or iii) in accordance with the terms applicable for US Federal Government officers or employees acting as part of their official duties; on a worldwide, perpetual, irrevocable, royalty-free basis to BMJ Publishing Group Ltd ("BMJ") its licensees and where the relevant Journal is co-owned by BMJ to the co-owners of the Journal, to publish the Work in this journal and any other BMJ products and to exploit all rights, as set out in our licence.

The Submitting Author accepts and understands that any supply made under these terms is made by BMJ to the Submitting Author unless you are acting as an employee on behalf of your employer or a postgraduate student of an affiliated institution which is paying any applicable article publishing charge ("APC") for Open Access articles. Where the Submitting Author wishes to make the Work available on an Open Access basis (and intends to pay the relevant APC), the terms of reuse of such Open Access shall be governed by a Creative Commons licence – details of these licences and which Creative Commons licence will apply to this Work are set out in our licence referred to above.

Other than as permitted in any relevant BMJ Author's Self Archiving Policies, I confirm this Work has not been accepted for publication elsewhere, is not being considered for publication elsewhere and does not duplicate material already published. I confirm all authors consent to publication of this Work and authorise the granting of this licence.

For peer review only

Title

Protocol for a multidimensional process evaluation of the Improving Primary Care After Stroke (IPCAS) randomised controlled trial

Authors

Maria Raisa Jessica (Ryc) Aquino, PhD
Primary Care Unit, Department of Public Health and Primary Care, University of Cambridge, UK
ra532@medschl.cam.ac.uk

Mullis, Ricky, PhD
Primary Care Unit, Department of Public Health and Primary Care, University of Cambridge, UK
rm582@medschl.cam.ac.uk

Kreit, Elizabeth
Primary Care Unit, Department of Public Health and Primary Care, University of Cambridge, UK
ek445@medschl.cam.ac.uk

Johnson, Vicki
Complex Intervention Team, Leicester Diabetes Centre, University Hospitals of Leicester NHS Trust, Leicester, UK
Vicki.Johnson@uhl-tr.nhs.uk

Grant, Julie
Primary Care Unit, Department of Public Health and Primary Care, University of Cambridge, UK
julie.grant@phpc.cam.ac.uk

Lim, Lisa
Primary Care Unit, Department of Public Health and Primary Care, University of Cambridge, UK
lccl2@medschl.cam.ac.uk

Sutton, Stephen, PhD
Primary Care Unit, Department of Public Health and Primary Care, University of Cambridge, UK
srs34@medschl.cam.ac.uk

Mant, Jonathan, MD
Primary Care Unit, Department of Public Health and Primary Care, University of Cambridge, UK
jm677@medschl.cam.ac.uk

On behalf of the IPCAS investigator team: Melanie Davies, Yvonne Doherty, Kamlesh Khunti, Bundy Mackintosh, Adrian Mander, Christopher McKevitt, Martin Roland, Stephen Sutton, Marion Walker, Elizabeth Warburton.

Corresponding Author

Maria Raisa Jessica (Ryc) Aquino
Primary Care Unit, Department of Public Health and Primary Care
University of Cambridge
Worts' Causeway
Cambridge CB1 8RN

For peer review only

ra532@medschl.cam.ac.uk
+441223 762499

Word count

2988

Keywords

Process evaluation, protocol, intervention fidelity, randomised controlled trial, stroke, primary care

Abstract

Introduction

Primary care interventions are often multicomponent, with several targets (e.g. patients, healthcare professionals). Improving Primary Care After Stroke (IPCAS) is a novel primary care-based model of long-term stroke care involving a review of stroke-related needs, a self-management programme, a direct point of contact at the General Practice (GP) surgery, enhanced communication between care services, and a directory of national and local community services, currently being evaluated in a cluster randomised controlled trial. Informed by Medical Research Council guidance for complex interventions and Behaviour Change Consortium fidelity framework, this protocol outlines the process evaluation of IPCAS within this RCT, exploring how the intervention was delivered in context, and how participants engaged with the intervention.

Methods and analysis

A mixed-methods design will be used. 1) Design: Intervention content will be specified and compared to 'usual care'. 2) Training: Practice staff training sessions and My Life After Stroke (MLAS) self-management course sessions will be audio- and video-recorded 3) Delivery: Healthcare professional self-reports and audio-recordings of intervention delivery and observations of MLAS course will be compared and coded separately. 4) Receipt and 5) Enactment: Structured stroke review records will be analysed quantitatively. Practices will be compared to identify the implementation of the intervention in context. All self-reports, observations and audio/video recordings will be coded using specifically developed checklists. Semi-structured interviews will be analysed thematically. Data will be analysed iteratively, independent of primary endpoint analysis.

Ethics

Favourable ethical opinion was gained on 19/12/2017 from Yorkshire & The Humber - Bradford Leeds NHS Research Ethics Committee (17/YH/0441).

Discussion

This comprehensive process evaluation will be used to interpret trial findings. Specifically, the level of fidelity achieved can be related to the effectiveness of the intervention. The methods outlined are applicable to other settings, and could inform intervention refinement to facilitate implementation of IPCAS in routine care.

Registration details

ClinicalTrials.gov Identifier: NCT03353519

For peer review only

Strengths and limitations of this study

- A strength of this process evaluation protocol includes the use of implementation frameworks, in particular a multidimensional approach to evaluation and intervention fidelity assessment
- This study applies quantitative and qualitative methods and an iterative approach to analysis to understand how the intervention is implemented in practice. In particular it will provide insight about uptake and barriers and facilitators to implementing a new model of care in General Practice
- A limitation of our approach is that interview findings might not be generalisable to other settings. However, we have adapted our interview sampling approach to capture as broad a range of study involvement as possible, and aim to triangulate these data with other data sources, such as observations, questionnaires, and routinely collected data.

Introduction

Stroke mortality is in decline, but remains a leading cause of disability (1–3). There are rising numbers of people with stroke living in the community, and with this comes increasing demand on primary care services to address long-term needs such as cognitive, psychological and social problems (3,4). Strategies to address these long-term needs are limited, with stroke survivors and their caregivers often reporting a lack of support (3,5). Stroke prevention is a priority for the National Health Service (NHS) in England (6), as most recently outlined in the *NHS Long Term Plan*, and internationally (6–9). Primary care services have tended to focus on secondary prevention of stroke and risk factor management (10,11) rather than addressing the longer term needs (5), and no formal primary care-based model of care exists to support community-dwelling stroke survivors.

The Improving Primary Care After Stroke (IPCAS) randomised controlled trial (RCT) aims to evaluate the clinical and cost effectiveness of a new model of primary care for stroke survivors living in the community compared with standard care, and has been described previously (12). This package of care is multicomponent; comprising a structured review using a novel needs checklist (13), a self-management course (My Life After Stroke; MLAS), enhanced communication between healthcare professionals (in submission), and a direct point of contact accessible to patients. The intervention and outcomes are summarised in Figure 1. The primary endpoint will be two sub-scales (emotion and handicap) of the Stroke Impact Scale (SIS v3.0) (14) as co-primary outcomes at 12 months after entry into the trial.

[INSERT FIGURE 1 HERE]

Complex interventions are widely used to address health problems and consist of multiple interacting components (15,16), often with several targets (e.g. patients, healthcare professionals) and implemented in various settings (17,18). Consequently, variations in implementation could influence intervention effects on outcomes. Process evaluations are especially important for randomised controlled trials such as IPCAS which is being conducted in 46 General Practices in the East of England and the East Midlands. These practices have contextual differences and thus the intervention may undergo adaptations as the study is conducted (18,19). It also has intervention

For peer review only - http://bmjopen.bmj.com/site/about/guidelines.xhtml

components that are aimed at stroke survivors, as well as healthcare professionals. This warrants a multifaceted process evaluation to identify successes, failures and/or unintended consequences (15,20,21). Understanding the delivery of complex interventions is key to determining feasibility of delivering these in other settings and at scale (17).

The UK Medical Research Council (MRC) suggests that process evaluations are conducted to understand how interventions are carried out, how intervention activities produce change, and how the context in which interventions are conducted influences delivery (18,19). In line with this, the US National Institute of Health Behaviour Change Consortium's (BCC) fidelity framework and guidance specifies five dimensions of treatment fidelity: (1) Design: the degree to which the intervention is distinct (e.g. through articulated intervention components and theoretical underpinning) from usual care or other treatment conditions; (2) Training: the extent to which the training of intervention providers is adequate through monitoring training processes and activities; (3) Delivery: the extent to which intervention components and/or procedures are provided as planned; (4) Receipt: the extent to which intervention recipients understand and engage with intervention components; and (5) Enactment: the extent to which intervention recipients carry out specific/relevant behaviours learnt from the intervention in their daily lives (22,23).

This paper outlines the aims, design and methods for an embedded process evaluation within an RCT. This process evaluation is a multidimensional assessment of the IPCAS trial, specifically to explore: 1) how the intervention was delivered; 2) how participants engaged with the intervention; and 3) if/how the context influenced delivery or conduct of the intervention.

Methods and analysis

Design

A mixed-methods sequential design will be applied. The process evaluation (encompassing intervention fidelity) will be conducted in parallel to the IPCAS trial. Table 1 outlines how the five BCC fidelity dimensions will be addressed in IPCAS.

[INSERT TABLE 1 HERE]

Fidelity of design

This concerns the completeness of the intervention specification, and the degree to which the intervention reflects its theoretical foundations (20,22). All IPCAS intervention components are defined in the trial protocol (12), some of which are standardised (e.g. structured review checklist for use in a one-off, face-to-face annual stroke review), and some not, in order to allow participants and practices flexibility to adapt these components to their needs (e.g. accessing direct point of contact and MLAS, enhanced communication between healthcare professionals). The intervention development process involved a multidisciplinary team (general practitioners (GP), patient representatives, primary care experts, applied health researchers, psychologists, stroke physicians, practice nurses, therapists), with pilot work informing the feasibility and acceptability of the intervention (12,13).

To ensure that the intervention reflects its theoretical underpinnings and is distinct from usual care, the intervention components (e.g. structured reviews, MLAS etc.) and parameters (e.g., frequency or dose, mode of delivery, duration) will be coded and compared by two independent coders in

For peer review only

accordance with established methods (20,22). As such, all participating general practices will be asked to provide details of 'usual care' processes for stroke care and long-term follow-up such as the frequency and content of follow-up appointments, healthcare professionals responsible for these, investigations conducted (e.g. blood tests, blood pressure and pulse measurements) and other actions taken (e.g. referrals or further investigations). Inter-rater reliability will be assessed using Cohen's kappa (95% CI) (24). Where intervention components of similar frequency and/or mode of delivery are found in both conditions, these will be classed as *fully convergent*; where intervention components are found in both experimental conditions but are of dissimilar frequency and/or mode of delivery, these will be classed as *partially convergent;*, and where intervention components are found only in one arm of the study, these will be classed as *unique* (20). Moreover, adaptations to the intervention will be recorded and monitored by the research team throughout the intervention period through maintaining a written record of these in the fidelity of delivery calls and interviews (detailed in the following subsections).

Fidelity of training

Participating GP practices will be given a 2-hour face-to-face training session where the research team will present the intervention components and facilitate discussions on how to conduct the intervention using fictional vignettes. A sample of the training sessions (20%; up to 4 sessions) will be audio-recorded, with two raters assessing whether planned components are present using a specifically developed checklist (Supplementary file 1). Training sessions will be randomly audio-recorded (with participant consent) after the fifth training session when trainers have had an opportunity to test their curriculum and materials, and adjustments made where necessary. The percentage of present planned components will be calculated, and inter-rater agreement assessed using Cohen's kappa (95% CI) (24). MLAS facilitators will be trained via a 3-day workshop, with the research team delivering presentations on stroke and its impact, providing trainees time to practise active listening skills, explaining the philosophy and theories underpinning the self-management course, demonstrating MLAS facilitation and providing an opportunity for trainees to rehearse course delivery. MLAS training will be video-recorded and coded by an independent rater according to a priori criteria (Supplementary file 2) to assess whether planned components are present. In addition, trainees will be encouraged to complete training evaluation forms, to assess facilitator knowledge of the MLAS curriculum and expected facilitator behaviours (Supplementary file 3). The percentage of present planned components will be calculated. To ensure skills are maintained, refresher training sessions will be offered as necessary. MLAS facilitators will receive mentoring from researchers as part of their training package after having had an opportunity to deliver a programme. Qualitative interviews with healthcare providers conducting structured reviews will explore their experiences of intervention training (see Fidelity of Delivery). Finally, process variables such as the number of healthcare professionals and facilitators trained, and duration of training sessions will be recorded and summarised.

Fidelity of delivery

Monitoring intervention delivery is critical to ensuring that the intervention is provided as planned; this will be conducted using quantitative and qualitative methods. The BCC guidance suggests that evaluating intervention sessions by observation (either directly or using audio/video-recording) is the gold standard for monitoring intervention delivery (22,23). To achieve this, a sample of the structured stroke reviews will be audio-recorded and coded by two independent raters according to

For peer review only - http://bmjopen.bmj.com/site/about/guidelines.xhtml

a pre-specified checklist (Supplementary file 4), which outlines the specific intervention components which healthcare providers are expected to cover. A sample of structured reviews carried out (up to 10% or 46 30-minute reviews across all intervention sites) will be audio-recorded at each practice. With participant consent, some reviews will be recorded after the fifth review has been conducted at each practice, which allows for healthcare professionals to rehearse and become familiarised with review components. Fidelity of delivery will be assessed by calculating the percentage of the pre-specified structured review content that is present. Inter-rater reliability will be assessed using Cohen's Kappa (95% CI) (24). To enable further exploration of delivery, healthcare professionals will be contacted by members of the research team regularly during the intervention period to discuss the structured reviews and to monitor whether the intervention being delivered is as planned, with responses recorded using a pre-specified checklist (see supplementary file 4). These data will be quantified by calculating the percentage of present pre-specified structured review content. Additionally, reasons for (e.g. lack of time) and the nature of (e.g. additional support provided) deviations from the developed intervention will be explored and documented. A purposively selected sample (approximate n=15; 10 intervention and 5 control) of healthcare professionals will also be invited to participate in semi-structured interviews to explore their overall experience of delivering the intervention (intervention) or participation in the study (control) in depth. Specifically, interviews will explore healthcare professionals' reflections on their experience of conducting the structured review, the functionality of the direct point of contact and service mapping tool, and their overall experience of the intervention (e.g. what worked well vs. what did not work well). Interviews will run for approximately 30min-1hr. These interviews are planned to take place from the 6-month follow-up period.

MLAS courses will also be assessed for fidelity of delivery. Specific contents of this self-management programme are provided in the IPCAS trial protocol (12) and MLAS development paper (in prep). A sample (i.e., two full MLAS courses; approx. 20 hours of observation) of pre-selected sessions of running MLAS programmes will be directly observed independently by two observers, with disagreements resolved through consensus discussion. These observers will have been trained in course delivery and observation. A specially developed observation checklist (Supplementary file 5) will be used to code for the presence or absence of intended course contents, materials and facilitator behaviours. Fidelity will be quantified by assessing the proportion of presence of pre-specified content (i.e. % coded components/planned components). These observations are planned to take place after MLAS facilitators have received mentoring; we also aim to observe all facilitators running MLAS courses at least once. MLAS facilitator-participant interactions will also be observed, using the DESMOND observation tool (Supplementary file 6), in line with similarly designed courses (25).

Finally, process variables will be collected to assess delivery, and summarised for each participating GP surgery, such as: practice list size, role of healthcare professional conducting reviews, the number of patients attending structured reviews, duration of each structured review conducted, number and content of referrals/signposting to other services, and number of MLAS courses conducted. These fidelity of delivery assessments and process measures will offer an insight into differences in intervention delivery between practices.

Fidelity of receipt and enactment

Fidelity of receipt and enactment, which concerns understanding of intervention content and application of skills gained from the intervention, respectively (22,26) will be assessed concurrently

For peer review only - http://bmjopen.bmj.com/site/about/guidelines.xhtml

as both encompass participant engagement (26), both quantitatively and qualitatively. Similar to the process outlined for assessing fidelity of delivery, patients will be contacted by members of the research team to discuss the structured reviews they were invited to. Pre-determined questions concerning the structured review will be asked and recorded on a checklist (Supplementary file 7). This will be quantified by calculating the percentage of the present pre-specified structured review content. Concerning MLAS, a purposively selected sample of participants (i.e., 10% of participants attending MLAS, approximately 20) will be invited to complete a specially developed receipt questionnaire (Supplementary file 8) at the end of each session attended (i.e., 6 per participant in total). If necessary, participants will be offered support in completing this.

Qualitative semi-structured interviews with study participants (approximate n= 25; 15 intervention and 10 control), will be conducted to explore their understanding and experience of the new model of care (intervention arm), and their care experiences since involvement in the trial (control arm). Participants will be selected using a random purposive method, given the large pool of eligible participants (e.g. those who attended structured reviews and MLAS, and those who did not), which will allow us to gain representative insights (27). Interviews will run for approximately 30min-1hr. These interviews are expected to take place from the 6-month follow-up period. Specifically, participants will be asked about any action plans they developed with healthcare professionals during their structured review and the outcomes of these, experiences of accessing the direct point of contact for their needs, as well as participation in MLAS. Interviews with control group participants will focus on any stroke-specific appointments/contacts with the GP practice since involvement in the study and any perceived changes in their care during this period. These different areas of focus will allow for an exploration of differences within and between experimental arms.

In addition, process variables will be collected routinely, such as the number of patients invited and consented, demographic characteristics, time since stroke, number of patients attending structured reviews, number of patients completing the checklist of needs, number of participants enrolled in MLAS (defined as attending the first individual appointment and first group session), and number of participants completing MLAS (defined as attending both individual appointments and at least three of the four group sessions).

Patient and public involvement

Protocol and materials development will involve patient and public involvement. Specifically, a PPI member will provide feedback on the process evaluation protocol to ensure procedures (e.g. recruitment, data collection) in place are appropriate and relevant. They will also be involved in participant questionnaire design to ensure it is aphasia-friendly, and pilot-test the interview schedule with a member of the research team.

Analysis plan

The process evaluation analysis will be undertaken separately to the trial outcome evaluation, and will inform the interpretation of these data. Quantitative aspects of the process evaluation (e.g. observational data, checklists/questionnaires) will be assessed using descriptive statistics (i.e. proportions, frequencies and means) and percentage implementation scores on various BCC fidelity dimensions. This will enable us to examine variation in implementation between GP practices at regular intervals. It is suggested that a score of 80-100% is representative of 'high' intervention fidelity, 51-79% is representative of 'moderate' intervention fidelity, and <50% is representative of 'low' intervention fidelity (28). Qualitative aspects of the process evaluation include audio-recorded interview data and textual data (i.e. qualitative data from checklists/questionnaires). All data will be transcribed verbatim. Interview data will be synthesised using thematic analysis by members of the

For peer review only

research team using established methods (29). This will also involve an iterative comparison of participant and healthcare professional interview data to understand similarities and differences between intervention providers and recipients. Textual data from checklists and questionnaires will be analysed using content analysis. NVivo 12 will be used to support qualitative data management and analysis.

The quantitative and qualitative analyses will be conducted and integrated at different phases of the evaluation. For example, comparing findings from different data sources such as participants' self-reported receipt and enactment questionnaires and healthcare professionals' self-reported delivery questionnaires with the audio-recorded observations of the structured stroke reviews.

Ethics

Favourable ethical opinion for the research was gained on 19th December 2017 from Yorkshire & The Humber - Bradford Leeds NHS Research Ethics Committee. Approval to start was given by the Health Research Authority (HRA) on 21st December 2017, prior to the recruitment of participants commencing at any NHS site.

Discussion

This multifaceted process evaluation aims to complement the effectiveness testing of a theory-driven complex intervention. It will generate knowledge concerning how and why this novel model of primary-care based stroke care could have an impact, thereby informing ways of scaling up the intervention and implementing the model in other settings as part of usual care. Specifically, the present intervention contains adaptable components as it is implemented pragmatically; therefore, process evaluation findings can offer an insight into how this model of care contributes to the evidence base by illustrating the extent to which a high level of fidelity is useful or necessary.

It has been suggested that there are four possible scenarios as to the contribution of process evaluation findings to investigating how interventions are effective or not (20): 1) successful intervention/high fidelity, where trial outcomes demonstrate improvements in the intervention group and treatment fidelity is deemed 'high', which increases confidence in associating trial outcomes with the intervention delivered; 2) successful intervention/low fidelity, where trial outcomes demonstrate improvements in the intervention group and treatment fidelity is deemed 'low', which suggests that there may have been confounding factors, or mechanisms of action through which the intervention operated which were not identified and assessed; 3) unsuccessful intervention/high fidelity: where trial outcomes demonstrate no improvements in the intervention group and treatment fidelity is deemed 'high', which supports the conclusion that the intervention was not effective, or could have been influenced by confounding factors; and 4) unsuccessful intervention/low fidelity: where trial outcomes demonstrate no improvements in the intervention group and treatment fidelity is deemed 'low', which suggests that the intervention was not tested as it was not carried out as planned, or that poor intervention fidelity could have negatively impacted intervention outcomes.

It is recommended that process evaluation protocols are reported and published to encourage transparency (17–20). The protocol described here builds on emerging process evaluation methodology literature which can inform the development and implementation of complex interventions.

For peer review only

Trial status

At the time of submission, the IPCAS trial recruitment is ongoing, with the target sample size of 920 surpassed in March 2019. The IPCAS process evaluation started in March 2018 alongside GP practice recruitment, with the fidelity of design and training data collection and analysis commencing July 2018. Pilot observations and interviews were conducted in February-March 2019, with ongoing iterative analysis.

Author contributions

JM, RM, and SS were responsible for obtaining the funding for this study. The design of the process evaluation was led by RA, with contributions from JM, RM, EK, VJ, LL. JM, RM, EK, VJ, and LL contributed to developing intervention fidelity measures. EK, VJ, LL and JG contributed to piloting intervention fidelity materials, participant recruitment, data collection and analysis. RA wrote the first draft of the manuscript, and all authors contributed to and approved the final version.

Funding

This study is funded by the National Institute for Health Research’s Programme Grant for Applied Research titled ‘Developing primary care services for stroke survivors’ reference PTC-RP-PG-0213-20001. The views expressed are those of the authors and not necessarily those of the NIHR or the Department of Health and Social Care. Jonathan Mant is an NIHR Senior Investigator.

Competing interests

None declared.

For peer review only

References

1. Feigin VL, Norrving B, Mensah GA. Global burden of stroke. Circ Res. 2017;120(3):439–48.

2. Lee S, Shafe AC, Cowie MR. UK stroke incidence, mortality and cardiovascular risk management 1999–2008: time-trend analysis from the General Practice Research Database. BMJ Open. 2011;1(2):e000269.

3. McKevitt C, Fudge N, Redfern J, Sheldenkar A, Crichton S, Rudd AR, et al. Self-reported long-term needs after stroke. Stroke. 2011;42(5):1398–403.

4. Murray J, Young J, Forster A. Review of longer-term problems after a disabling stroke. Rev Clin Gerontol. 2007;17(4):277–92.

5. Pindus DM, Mullis R, Lim L, Wellwood I, Rundell AV, Aziz NAA, et al. Stroke survivors' and informal caregivers' experiences of primary care and community healthcare services–A systematic review and meta-ethnography. PloS One. 2018;13(2):e0192533.

6. NHS England. The NHS Long Term Plan. 2019.

7. US Department of Health and Human Services. Healthy people 2020 [Internet]. Office of Disease Prevention and Health Promotion; 2010 [cited 2019 Dec 17]. Available from: https://www.healthypeople.gov/sites/default/files/HP2020_brochure_with_LHI_508_FNL.pdf

8. The Ministry of Health and Care Services (Norway). Care plan 2020: The Norwegian Government's plan for the care services field for 2015-2020 [Internet]. The Ministry of Health and Care Services (Norway); 2015 [cited 2019 Dec 17]. Available from: https://www.regjeringen.no/contentassets/af2a24858c8340edaf78a77e2fbe9cb7/careplan2020_eng.pdf

9. Centers for Disease Control and Prevention. A study of primary stroke center policy: Recommendations for policy implementation [Internet]. US Department of Health and Human Services; 2012 [cited 2019 Dec 17]. Available from: https://www.cdc.gov/dhdsp/docs/Primary_Stroke_Center_Policies_Recommendations.pdf

10. Norrving B, Davis SM, Feigin VL, Mensah GA, Sacco RL, Varghese C. Stroke prevention worldwide-what could make it work. Neuroepidemiology. 2015;45(3):215–20.

11. Joubert J, Reid C, Joubert L, Barton D, Ruth D, Jackson D, et al. Risk factor management and depression post-stroke: the value of an integrated model of care. J Clin Neurosci. 2006;13(1):84–90.

12. Mullis R, Aquino MRJ, Dawson S, Johnson V, Jowett S, Kreit E, et al. The IPCAS Trial (Improving Primary Care After Stroke): protocol of a randomised controlled trial to evaluate a novel model of care for stroke survivors living in the community. BMJ Open. 2019;9:e030285.

13. Turner GM, Mullis R, Lim L, Kreit L, Mant J. Using a checklist to facilitate management of long-term care needs after stroke: insights from focus groups and a feasibility study. BMC Fam Pract. 2019;20(1):2.

14. Jenkinson C, Fitzpatrick R, Crocker H, Peters M. The Stroke Impact Scale: validation in a UK setting and development of a SIS short form and SIS index. Stroke. 2013;44(9):2532–5.

For peer review only

15. Craig P, Dieppe P, Macintyre S, Michie S, Nazareth I, Petticrew M, et al. Developing and evaluating complex interventions: the new Medical Research Council guidance. BMJ. 2008;337:a1655–a1655.

16. Campbell M, Fitzpatrick R, Haines A, Kinmonth AL, Sandercock P, Spiegelhalter D, et al. Framework for design and evaluation of complex interventions to improve health. Bmj. 2000;321(7262):694–6.

17. Liu H, Lindley R, Alim M, Felix C, Gandhi DB, Verma SJ, et al. Protocol for process evaluation of a randomised controlled trial of family-led rehabilitation post stroke (ATTEND) in India. BMJ Open. 2016;6(9):e012027.

18. Moore GF, Audrey S, Barker M, Bond L, Bonell C, Hardeman W, et al. Process evaluation of complex interventions: Medical Research Council guidance. bmj. 2015;350:h1258.

19. Campbell-Scherer D, Saitz R. Improving reporting and utility of evaluations of complex interventions. BMJ Evid-Based Med. 2016;21:1–3.

20. Lorencatto F, Gould NJ, McIntyre SA, During C, Bird J, Walwyn R, et al. A multidimensional approach to assessing intervention fidelity in a process evaluation of audit and feedback interventions to reduce unnecessary blood transfusions: a study protocol. Implement Sci. 2016;11(1):163.

21. Oakley A, Strange V, Bonell C, Allen E, Stephenson J. Process evaluation in randomised controlled trials of complex interventions. Bmj. 2006;332(7538):413–6.

22. Borrelli B, Sepinwall D, Ernst D, Bellg AJ, Czajkowski S, Breger R, et al. A new tool to assess treatment fidelity and evaluation of treatment fidelity across 10 years of health behavior research. J Consult Clin Psychol. 2005;73(5):852.

23. Bellg AJ, Borrelli B, Resnick B, Hecht J, Minicucci DS, Ory M, et al. Enhancing Treatment Fidelity in Health Behavior Change Studies: Best Practices and Recommendations From the NIH Behavior Change Consortium. Health Psychol. 2004;23(5):443–51.

24. Walton H, Spector A, Williamson M, Tombor I, Michie S. Developing quality fidelity and engagement measures for complex health interventions. Br J Health Psychol. 2019;

25. Skinner T, Carey M, Cradock S, Dallosso H, Daly H, Davies MJ, et al. 'Educator talk'and patient change: some insights from the DESMOND (Diabetes Education and Self Management for Ongoing and Newly Diagnosed) randomized controlled trial. Diabet Med. 2008;25(9):1117–20.

26. Walton H, Spector A, Tombor I, Michie S. Measures of fidelity of delivery of, and engagement with, complex, face-to-face health behaviour change interventions: A systematic review of measure quality. Br J Health Psychol. 2017;22(4):872–903.

27. Patton M. Qualitative research and evaluation methods. 4th ed. Saint Paul, MN: SAGE; 2015.

28. Noell GH, Gresham FM, Gansle KA. Does treatment integrity matter? A preliminary investigation of instructional implementation and mathematics performance. J Behav Educ. 2002;11(1):51–67.

29. Braun V, Clarke V. Thematic analysis. In: Cooper H, Camic PM, Long DL, Panter A, Rindskopf D, Sher KJ, editors. APA handbook of resaerch methods in psychology, Vol 2 Resaerch designs:

For peer review only

Quantitative, qualitative, neuropsychological, amd biological. American Psychological Association; 2012.

For peer review only

Strategies to assess implementation fidelity in the *Improving Primary Care After Stroke (IPCAS)* trial

Fidelity of design	Fidelity of training	Fidelity of delivery	Fidelity of receipt and enactment
Coding intervention components (i.e. IPCAS training manual, MLAS curriculum) to theoretical underpinnings<br><br>Comparison of convergence between intervention and control groups (includes recording of participating surgeries' 'usual care' practices)	Training evaluation forms (MLAS)<br><br>Video-recorded observations (MLAS)<br><br>Audio-recorded observations (IPCAS)<br><br>Post-intervention interviews (healthcare professionals, IPCAS)	Audio-recorded observations (IPCAS)<br><br>Structured telephone calls to healthcare professionals (IPCAS)<br><br>Direct observations (MLAS)<br><br>Post-intervention interviews (IPCAS)	Self-report questionnaire (MLAS)<br><br>Post-review structured telephone calls to participants (IPCAS)<br><br>Post-intervention participant interviews

For peer review only

Contextual factors within intervention environment: 'Usual care' at each practice, socioeconomic factors, stroke-related factors (e.g., time since stroke, comorbidities)

Improving Primary Care After Stroke (IPCAS): a primary care-based model of care to support community-dwelling stroke survivors

Participant-focused components
15-item checklist of common post-stroke needs sent to participant by post. Followed by:

Structured review appointment: Up to 30-min with a trained member of the practice team (e.g. nurse, GP) to discuss up to 3 needs prioritised by the patient from the checklist. The outcomes are an action plan agreed with the patient on how to address the key needs, information on the Direct Point of Contact (DPOC) service, and on the My Life After Stroke (MLAS) course.

My Life After Stroke (MLAS), an optional 6-week, group-based self-management course delivered by two trained facilitators in a local community venue. The first and final appointments are individual appointments lasting up to 45 minutes, in between which are four-weekly group sessions focused on learning about the consequences of a stroke, and managing one's health and wellbeing using goal setting and problem-solving strategies. In the final group session, participants will also be given an abbreviated print version of the service mapping tool (see below).

Healthcare professional-focused components:
Training on new model of primary care: One-off, face-to-face session with trial team lasting up to 2 hours.

DPOC: Trained practice staff will provide stroke-specific advice, information and/or support to study participants by telephone.

Optimised communication between healthcare professionals: A meeting will be arranged between primary care and specialist staff (hospital and community) to facilitate interprofessional communication. All practice staff involved with providing care to stroke survivors will be encouraged to attend any meetings/training sessions organised by specialists, and provided specialists' contact details. Video recordings of local specialist providers describing their service, commonly referred patients to the service, and means of contact/referral will be provided to practice staff.

Service mapping tool: This is a bespoke directory of national and local services provided to practices in Excel and print formats. These can be used at the structured review, or when providing support via the DPOC service.

Primary outcomes

Baseline, 6, and 12 mos.:
Emotion and handicap sub-scales of Stroke Impact Scale v3.0

Secondary outcomes

6 and 12 mos.:
EQ-5D-5L, ICEpop CAPability measure for Adults

12 mos. only:
Southampton Stroke Self-management Questionnaire, Health Literacy Questionnaire

Long-term outcomes

Cost-utility as determined in within-trial economic evaluation

**IPCAS trial provider process evaluation/fidelity checklist – training phase**							
**Site ID:**		**Duration of training:**			**Training date (Coding date):**		
**Activity**			**Tick as appropriate**			**If *present to some extent*, provide reason(s) for this rating**	**Other comments**
			**Present**	**Absent**	**Partially done**		
**Structured review**	1. 15-item checklist of needs	a) Discussion of purpose and structure of checklist					
		b) Instructions on how to use checklist					
	2. Physical check	a) Discussion of carrying out optional physical checks at review (in line with QOF)					
	3. Development of action plan(s)	a) Discussion of action plan					
		b) Instructions given: how to log and review agreed actions (using template)					
	4. MLAS	a) Discussion of purpose and structure of MLAS					
		b) Provided instructions for how patients can access MLAS					
	5. Direct point of contact to stroke survivor and carer(s)	a) Given instructions for offering 'direct point of contact' service					
		b) Discussion of how to provide instructions to patients about contacting practice at the end of review					
**Use of direct point of contact**	1. Signposting to services	a) Discussed service mapping: what it is					
		b) Discussed how to use service mapping					

For peer review only

For peer review only - http://bmjopen.bmj.com/site/about/guidelines.xhtml

**IPCAS trial provider process evaluation/fidelity checklist – training phase**							
**Site ID:**	**Duration of training:**		**Training date (Coding date):**				
**Activity**			**Tick as appropriate**			**If *present to some extent*, provide reason(s) for this rating**	**Other comments**
			**Present**	**Absent**	**Partially done**		
	2. Provide support and advice	a) Gave information on structure and scope of direct point of contact service: giving advice for stroke-specific issues					
		b) Gave information on structure and scope of direct point of contact service: arranging follow-up appointment(s)					
		c) Gave information on structure and scope of direct point of contact service: brief telephone support/case management					
**Enhanced communication pathways**	1. Discussion on communication pathways	a) Discussed broad structure of enhanced communication pathways					
		b) Initial discussion about dates for face-to-face meeting					
**END OF CHECKLIST**							

For peer review only - http://bmjopen.bmj.com/site/about/guidelines.xhtml

IPCAS trial process evaluation/fidelity checklist
MLAS facilitator training

**Date:**					
**Start time:**					
**End time:**					
**Training day 1**					
	**Please tick as appropriate**			**If *absent, attempted* or *not applicable*, please provide reasons**	**Other comments**
	**Present**	**Absent**	**Attempted**		
**Session 1: Welcome and introductions**					
**Duration of session:**					
**Materials**					
Timetable available	☐	☐	☐		
Headed flipchart paper – working together	☐	☐	☐		
Pre-training 'evaluation' form given (1 per participant)	☐	☐	☐		
**Content**					
Welcome to MLAS	☐	☐	☐		
Introduction of trainers	☐	☐	☐		

For peer review only - http://bmjopen.bmj.com/site/about/guidelines.xhtml

**Date:**					
**Start time:**					
**End time:**					
**Training day 1**					
	**Please tick as appropriate**			**If *absent, attempted* or *not applicable*, please provide reasons**	**Other comments**
	**Present**	**Absent**	**Attempted**		
Housekeeping details provided	☐	☐	☐		
Aim of training discussed	☐	☐	☐		
Training overview discussed	☐	☐	☐		
Outline of Day 1 discussed	☐	☐	☐		
Working together discussed	☐	☐	☐		
Provided time for participants to complete pre-training ‘evaluation’ form	☐	☐	☐		

For peer review only

**Session 2: Your story**					
**Duration of session:**					
**Materials**					
Headed flipchart paper (1: Name, role, place of work, 2: Experience, 3: Expectation of training, 4: Your important question)	☐	☐	☐		
**Content**					
Asked: Name, role, place of work	☐	☐	☐		
Asked: Experience (facilitating/observing groups, working with stroke)	☐	☐	☐		
Asked: Expectation(s)	☐	☐	☐		
Asked: Any question in relation to MLAS	☐	☐	☐		
Provided opportunity for each trainee to answer the above questions and record on relevant flipchart	☐	☐	☐		
**Session 3: What is stroke?**					
**Duration of session:**					
**Materials**					
Body outline used	☐	☐	☐		
Images shown: clot, bleed, brain areas	☐	☐	☐		
Images shown: risk factors, neuroplasticity	☐	☐	☐		

For peer review only - http://bmjopen.bmj.com/site/about/guidelines.xhtml

Videos shown: Act FAST, neuroplasticity	☐	☐	☐		
Images shown: health and well-being	☐	☐	☐		
Sticky notes used	☐	☐	☐		
**Content**					
Discussed 'what is stroke?'	☐	☐	☐		
Discussed signs and symptoms – ACTFAST	☐	☐	☐		
Discussed impacts of a stroke (including physical, emotional, cognitive, communication, relationships)	☐	☐	☐		
Discussed what neuroplasticity is	☐	☐	☐		
Discussed stroke risk factors	☐	☐	☐		
Discussed managing risk factors	☐	☐	☐		
Participants given an opportunity to contribute to discussion and activities	☐	☐	☐		
**Session 4: Cognitive effects of stroke**					
**Duration of session:**					
**Materials**					
PowerPoint slides used: cognitive effects of stroke	☐	☐	☐		
Video shown: Hidden effects of stroke	☐	☐	☐		

For peer review only - http://bmjopen.bmj.com/site/about/guidelines.xhtml

Booklet given: Complete guide to Cognitive problems (1 copy/facilitator)	☐	☐	☐		
**Content**					
Session introduced	☐	☐	☐		
Discussed memory problems after a stroke	☐	☐	☐		
Explored attention difficulties	☐	☐	☐		
Explored changes to perception	☐	☐	☐		
Explored effects on decision-making	☐	☐	☐		
Explored social cognition problems	☐	☐	☐		
Discussed how we can support stroke survivors experiencing these effects/top tips	☐	☐	☐		
Discussed the impact of impaired cognition on the stroke survivor and/or carer/family	☐	☐	☐		
Participants given an opportunity to contribute to discussion and activities	☐	☐	☐		
**Session 5: Emotional changes after stroke**					
**Duration of session:**					
**Materials**					
Video shown: The emotional impact of stroke	☐	☐	☐		
PowerPoint slides: Emotional problems following stroke					

For peer review only

For peer review only - http://bmjopen.bmj.com/site/about/guidelines.xhtml

Booklet given: Complete guide to Emotional changes after stroke (1 copy/facilitator)	☐	☐	☐		
**Content**					
Session introduced	☐	☐	☐		
Discussed the emotions stroke survivors highlighted in video observed	☐	☐	☐		
Discussed stages of loss	☐	☐	☐		
Discussed problems stroke survivors might face with emotions	☐	☐	☐		
Discussed difficulty controlling emotions	☐	☐	☐		
Discussed how these emotions can influence people's thoughts and behaviours	☐	☐	☐		
Discussed how facilitators can support stroke survivors experiencing these effects/top tips	☐	☐	☐		
Participants given an opportunity to contribute to discussion and activities	☐	☐	☐		
**Session 6: Relationships**					
**Duration of session:**					
**Materials**					
PowerPoint slides used: Relationships	☐	☐	☐		
**Content**					

For peer review only

For peer review only - http://bmjopen.bmj.com/site/about/guidelines.xhtml

Session introduced	☐	☐	☐		
Discussed the effect of a stroke on relationships (partners, friends, family members)	☐	☐	☐		
Discussed problems and solutions concerning physical intimacy after a stroke	☐	☐	☐		
Participants given an opportunity to contribute to discussion and activities	☐	☐	☐		
**Session 7: Communication challenges for survivors of stroke**					
**Duration of session:**					
**Materials**					
PowerPoint slides used: Communication	☐	☐	☐		
Video used: Living with aphasia; aphasia etiquette	☐	☐	☐		
Booklet given: Complete guide to Communication problems after stroke (1 copy/facilitator)	☐	☐	☐		
**Content**					
Session introduced	☐	☐	☐		
Group activity: Identifying tasks the brain needs to complete in order to be able to answer a question	☐	☐	☐		

For peer review only - http://bmjopen.bmj.com/site/about/guidelines.xhtml

Discussed communication problems after a stroke	☐	☐	☐		
Discussed how we can support stroke survivors experiencing communication problems (top tips, resources/aids)	☐	☐	☐		
Participants given an opportunity to contribute to discussion and activities	☐	☐	☐		
**Session 8: Summary and evaluation**					
**Duration of session:**					
**Materials**					
Training evaluation form given	☐	☐	☐		
Questions relating to MLAS flipchart	☐	☐	☐		
**Content**					
Summary of Day 1 Training	☐	☐	☐		
Provided time for participants to complete training evaluation form	☐	☐	☐		
Provided details of Day 2 Training	☐	☐	☐		
Participants given an opportunity to check if their questions addressed, and to ask any further questions	☐	☐	☐		
**END OF DAY 1 TRAINING**					

For peer review only - http://bmjopen.bmj.com/site/about/guidelines.xhtml

IPCAS trial process evaluation/fidelity checklist
MLAS facilitator training

**Date:**					
**Start time:**					
**End time:**					
**Training day 2**					
	**Please tick as appropriate**			**If *absent, attempted* or *not applicable*, please provide reasons**	**Other comments**
	**Present**	**Absent**	**Attempted**		
**Session 1: Welcome and introductions**					
**Duration of session:**					
**Materials**					
Timetable available	☐	☐	☐		
Headed flipchart paper from day 1 ('Your story' - name, experience, expectation, important question and working together as a group)	☐	☐	☐		
**Content**					
Welcome	☐	☐	☐		
Introduction of trainers	☐	☐	☐		

For peer review only - http://bmjopen.bmj.com/site/about/guidelines.xhtml

**Date:**					
**Start time:**					
**End time:**					
**Training day 2**					
	**Please tick as appropriate**			**If *absent, attempted* or *not applicable*, please provide reasons**	**Other comments**
	**Present**	**Absent**	**Attempted**		
Housekeeping details provided	☐	☐	☐		
Overall aim/format of training discussed	☐	☐	☐		
Outline of Day 2 discussed	☐	☐	☐		
Invited any new participants to introduce themselves to group using 'Your story' flipchart from Day 1	☐	☐	☐		
**Session 2: Background to MLAS**					
**Duration of session:**					
**Materials**					
Flipchart paper, pens	☐	☐	☐		
A4 Quadrant image	☐	☐	☐		
**Content**					
Discussed what MLAS is	☐	☐	☐		

For peer review only - http://bmjopen.bmj.com/site/about/guidelines.xhtml

**Date:**					
**Start time:**					
**End time:**					
**Training day 2**					
	**Please tick as appropriate**			**If *absent, attempted* or *not applicable*, please provide reasons**	**Other comments**
	**Present**	**Absent**	**Attempted**		
Discussed what a structured education programme is	☐	☐	☐		
Discussed rationale for MLAS	☐	☐	☐		
Discussed aims and format of MLAS course	☐	☐	☐		
Participants given an opportunity to be involved in discussion and ask questions	☐	☐	☐		
**Session 3: Philosophy**					
**Duration of session:**					
**Materials**					
How are you doing? Worksheet (1 per participant)	☐	☐	☐		
PowerPoint slides used: Philosophy	☐	☐	☐		
**Content**					

For peer review only - http://bmjopen.bmj.com/site/about/guidelines.xhtml

**Date:**					
**Start time:**					
**End time:**					
**Training day 2**					
	**Please tick as appropriate**			**If *absent, attempted* or *not applicable*, please provide reasons**	**Other comments**
	**Present**	**Absent**	**Attempted**		
Discussed what philosophy is and how it affects our behaviours	☐	☐	☐		
Explored the medical model and empowerment approach	☐	☐	☐		
Explored the philosophy and approach of the MLAS programme	☐	☐	☐		
Facilitated individual activity ('How are you doing' quiz) and gave an opportunity for each trainee to complete their own worksheet	☐	☐	☐		
Discussed the need to have importance, confidence and readiness in order to change behaviour (and not just knowledge)	☐	☐	☐		
Presented overall aim of MLAS and its core philosophy statements	☐	☐	☐		
Discussed MLAS facilitators' role					

For peer review only

For peer review only - http://bmjopen.bmj.com/site/about/guidelines.xhtml

**Date:**					
**Start time:**					
**End time:**					
**Training day 2**					
	**Please tick as appropriate**			**If *absent, attempted* or *not applicable*, please provide reasons**	**Other comments**
	**Present**	**Absent**	**Attempted**		
Group activity: Challenges experienced/perceived by facilitators when using the philosophy/approach underpinning MLAS	☐	☐	☐		
Participants given an opportunity to be involved in discussion (e.g. by giving feedback) and ask questions	☐	☐	☐		
**Session 4: Facilitator behaviours**					
**Duration of session:**					
**Materials**					
Rice pudding exercise	☐	☐	☐		
MLAS facilitator behaviours checklist given (1 per participant)	☐	☐	☐		
**Content**					

For peer review only - http://bmjopen.bmj.com/site/about/guidelines.xhtml

**Date:**					
**Start time:**					
**End time:**					
**Training day 2**					
	**Please tick as appropriate**			**If *absent, attempted* or *not applicable*, please provide reasons**	**Other comments**
	**Present**	**Absent**	**Attempted**		
Session introduced	☐	☐	☐		
Rice pudding activity (demonstrating different facilitator behaviours – 'good' and 'bad')	☐	☐	☐		
Discussed positive and negative facilitator behaviours that were observed	☐	☐	☐		
Discussed some of the behaviours on the behaviours checklist, and provided examples of what would be observed if such behaviours were being done (e.g. body language, words used etc.)	☐	☐	☐		
Encouraged participants to engage in discussion about any stroke-specific behaviours needed	☐	☐	☐		
Participants given an opportunity to be involved in discussion (e.g. by giving feedback) and ask questions	☐	☐	☐		

For peer review only

For peer review only - http://bmjopen.bmj.com/site/about/guidelines.xhtml

**Date:**					
**Start time:**					
**End time:**					
**Training day 2**					
	**Please tick as appropriate**			**If *absent, attempted* or *not applicable*, please provide reasons**	**Other comments**
	**Present**	**Absent**	**Attempted**		
**Session 5: Facilitation skills**					
**Duration of session:**					
**Materials**					
PowerPoint slides used: Facilitation skills	☐	☐	☐		
Flipchart paper and pens					
Open-ended Questions, Affirmations, Reflective listening, Summaries (OARS) sheets given (1 per participant)	☐	☐	☐		
**Content**					
Session introduced	☐	☐	☐		
Facilitated group activity on facilitator role and responsibilities	☐	☐	☐		
OARS introduced to participants	☐	☐	☐		

For peer review only - http://bmjopen.bmj.com/site/about/guidelines.xhtml

**Date:**					
**Start time:**					
**End time:**					
**Training day 2**					
	**Please tick as appropriate**			**If *absent, attempted* or *not applicable*, please provide reasons**	**Other comments**
	**Present**	**Absent**	**Attempted**		
Group activity: Challenging scenarios and resolutions	☐	☐	☐		
Encouraged participants to share personal learning	☐	☐	☐		
Participants given an opportunity to be involved in discussion (e.g. by giving feedback) and ask questions	☐	☐	☐		
**Session 6: Theories – Narrative Approach; Self-efficacy**					
**Duration of session:**					
**Materials**					
PowerPoint slides used: Theories	☐	☐	☐		
Sticky notes, flipchart paper, pens	☐	☐	☐		
MLAS Roadmap and accessories	☐	☐	☐		
**Content**					

For peer review only - http://bmjopen.bmj.com/site/about/guidelines.xhtml

**Date:**					
**Start time:**					
**End time:**					
**Training day 2**					
	**Please tick as appropriate**			**If *absent, attempted* or *not applicable*, please provide reasons**	**Other comments**
	**Present**	**Absent**	**Attempted**		
Session introduced	☐	☐	☐		
Introduced the Narrative Approach	☐	☐	☐		
Demonstrated how the narrative approach is used in MLAS (journey analogy)	☐	☐	☐		
Introduced Self-efficacy (Mastery, vicarious learning, verbal persuasion and emotions management)	☐	☐	☐		
Group activity: Real-world experiences of self-efficacy	☐	☐	☐		
Examples provided where self-efficacy is used during MLAS	☐	☐	☐		
Introduced cognitive behavioural approach and gave an example of how it is used in MLAS	☐	☐	☐		

For peer review only - http://bmjopen.bmj.com/site/about/guidelines.xhtml

**Date:**					
**Start time:**					
**End time:**					
**Training day 2**					
	**Please tick as appropriate**			**If *absent, attempted* or *not applicable*, please provide reasons**	**Other comments**
	**Present**	**Absent**	**Attempted**		
Participants given an opportunity to be involved in discussion (e.g. by giving feedback) and ask questions	☐	☐	☐		
**Session 7: Introduction to Quality Assurance**					
**Duration of session:**					
**Materials**					
Flipchart paper and pens	☐	☐	☐		
**Content**					
Session introduced	☐	☐	☐		
Discussed what quality assurance is and its important	☐	☐	☐		
Facilitated small group activity (recipe and ingredients for making an Apple Pie)	☐	☐	☐		

For peer review only - http://bmjopen.bmj.com/site/about/guidelines.xhtml

**Date:**					
**Start time:**					
**End time:**					
**Training day 2**					
	**Please tick as appropriate**			**If *absent, attempted* or *not applicable*, please provide reasons**	**Other comments**
	**Present**	**Absent**	**Attempted**		
Encouraged each group to feedback on group activity.	☐	☐	☐		
Compared similarities and differences of Apple Pie recipes and linked to quality assurance of MLAS (ingredients = curriculum, method = delivery)	☐	☐	☐		
Summarised group discussion on quality assurance processes	☐	☐	☐		
Participants given an opportunity to be involved in discussion (e.g. by giving feedback) and ask questions	☐	☐	☐		
**Session 8: A journey through the MLAS curriculum**					
**Duration of session:**					
**Materials**					

For peer review only - http://bmjopen.bmj.com/site/about/guidelines.xhtml

**Date:**					
**Start time:**					
**End time:**					
**Training day 2**					
	**Please tick as appropriate**			**If *absent, attempted* or *not applicable*, please provide reasons**	**Other comments**
	**Present**	**Absent**	**Attempted**		
MLAS curriculum given to each facilitator	☐	☐	☐		
Participant (stroke survivor) handbooks given to each facilitator (4 handbooks in total)	☐	☐	☐		
A journey through the curriculum question sheet given to each facilitator	☐	☐	☐		
**Content**					
Provided an overview of materials given	☐	☐	☐		
Explained the purpose of 'a journey through the curriculum question sheet' and instructions for completion	☐	☐	☐		
**Session 9: Close of the day reflections and evaluation**					
**Duration of session:**					
**Materials**					
Training evaluation form (day 2) given	☐	☐	☐		

For peer review only - http://bmjopen.bmj.com/site/about/guidelines.xhtml

**Date:**					
**Start time:**					
**End time:**					
**Training day 2**					
	**Please tick as appropriate**			**If *absent, attempted* or *not applicable*, please provide reasons**	**Other comments**
	**Present**	**Absent**	**Attempted**		
Questions relating to MLAS flipchart	☐	☐	☐		
**Content**					
Summary of Day 2 training by encouraging participants to share learning about what was covered	☐	☐	☐		
Provided time for participants to complete training evaluation form	☐	☐	☐		
Provided details of Day 3 Training	☐	☐	☐		
Participants given an opportunity to check if their question (from flipchart) was addressed, and to ask any further questions	☐	☐	☐		
**END OF DAY 2 TRAINING**					

For peer review only - http://bmjopen.bmj.com/site/about/guidelines.xhtml

IPCAS trial process evaluation/fidelity checklist

MLAS facilitator training

**Date:**					
**Start time:**					
**End time:**					
**Training day 3**					
	**Please tick as appropriate**			**If *absent* or *attempted* please provide reasons**	**Other comments**
	**Present**	**Absent**	**Attempted**		
**Session 1: Welcome and introductions**					
**Duration of session:**					
**Materials**					
Timetable available	☐	☐	☐		
Headed flipchart paper from days 1+2 ('Your story' - name, experience, expectation, important question and working together as a group)	☐	☐	☐		
**Content**					
Welcome	☐	☐	☐		

For peer review only - http://bmjopen.bmj.com/site/about/guidelines.xhtml

**Date:**					
**Start time:**					
**End time:**					
**Training day 3**					
	**Please tick as appropriate**			**If *absent* or *attempted* please provide reasons**	**Other comments**
	**Present**	**Absent**	**Attempted**		
Re-introduction of trainers	☐	☐	☐		
Housekeeping details provided	☐	☐	☐		
Aim of training discussed	☐	☐	☐		
Outline of Day 3 discussed	☐	☐	☐		
Invited any new participants to introduce themselves to group using 'Your story' flipchart from Day 1+2	☐	☐	☐		
**Session 2: Reflection from 'A journey through the curriculum' activity**					
**Duration of session:**					
**Materials**					
A journey through the curriculum activity sheet	☐	☐	☐		
A journey through the curriculum activity answer sheet given	☐	☐	☐		
MLAS curriculum	☐	☐	☐		
**Content**					

For peer review only - http://bmjopen.bmj.com/site/about/guidelines.xhtml

**Date:**					
**Start time:**					
**End time:**					
**Training day 3**					
	**Please tick as appropriate**			**If *absent* or *attempted* please provide reasons**	**Other comments**
	**Present**	**Absent**	**Attempted**		
Invited participants to share experiences of completing 'a journey through the curriculum' activity	☐	☐	☐		
Discussed format of the programme (1:1 and group-based sessions; interchangeable sessions)	☐	☐	☐		
Discussed format of the curriculum (e.g. key for how to use the curriculum, duration for each section, section overviews)	☐	☐	☐		
Discussed involvement of carers in MLAS and format of this	☐	☐	☐		
Participants given an opportunity to be involved in discussion and ask questions	☐	☐	☐		
**Session 3: A walk through the MLAS curriculum**					
**Duration of session:**					
**Materials**					

For peer review only - http://bmjopen.bmj.com/site/about/guidelines.xhtml

**Date:**					
**Start time:**					
**End time:**					
**Training day 3**					
	**Please tick as appropriate**			**If *absent* or *attempted* please provide reasons**	**Other comments**
	**Present**	**Absent**	**Attempted**		
MLAS curriculum	☐	☐	☐		
Participant (stroke survivor) handbooks	☐	☐	☐		
Resources/accessories for programme available	☐	☐	☐		
**Content**					
Discussed the overall aims of the study and the intervention	☐	☐	☐		
Presented each of the MLAS sessions with the resources	☐	☐	☐		
Demonstrated how to use the resources	☐	☐	☐		
Demonstrated links between the sessions and materials to the theories and philosophy applied	☐	☐	☐		
Discussed challenges to delivery and strategies for addressing these	☐	☐	☐		
Discussed tailoring of sessions	☐	☐	☐		

For peer review only - http://bmjopen.bmj.com/site/about/guidelines.xhtml

**Date:**					
**Start time:**					
**End time:**					
**Training day 3**					
	**Please tick as appropriate**			**If *absent* or *attempted* please provide reasons**	**Other comments**
	**Present**	**Absent**	**Attempted**		
Discussed some examples of what previous facilitators have observed/dealt with during previous courses	☐	☐	☐		
Participants given an opportunity to be involved in discussion and ask questions	☐	☐	☐		
**Session 4: Preparing for having a go**					
**Duration of session:**					
**Materials**					
Facilitator curricula	☐	☐	☐		
'Have a go' cards (1 per facilitator)	☐	☐	☐		
Roadmap and accessories available	☐	☐	☐		
A4 images: blood clot, bleed available	☐	☐	☐		
A6 images: risk factors, risks I can/cannot change available	☐	☐	☐		

For peer review only - http://bmjopen.bmj.com/site/about/guidelines.xhtml

**Date:**					
**Start time:**					
**End time:**					
**Training day 3**					
	**Please tick as appropriate**			**If *absent* or *attempted* please provide reasons**	**Other comments**
	**Present**	**Absent**	**Attempted**		
A4 and A6 images: health and wellbeing available	☐	☐	☐		
A4 images: dashboard available	☐	☐	☐		
Writing instruments available (sticky notes, flipchart paper, pens, Blu Tack)	☐	☐	☐		
**Content**					
Session introduced	☐	☐	☐		
Participants divided into 2 groups	☐	☐	☐		
Explained that each participant has been given details of a section of the MLAS course (to be delivered in the next session of training)	☐	☐	☐		
Given instructions for next steps (i.e. time to prepare for delivery of chosen 10-minute section, how 'have a go' session will run)	☐	☐	☐		

For peer review only

For peer review only - http://bmjopen.bmj.com/site/about/guidelines.xhtml

**Date:**					
**Start time:**					
**End time:**					
**Training day 3**					
	**Please tick as appropriate**			**If *absent* or *attempted* please provide reasons**	**Other comments**
	**Present**	**Absent**	**Attempted**		
Offered participants support for preparing for their sessions (e.g. providing relevant resources, explaining content)	☐	☐	☐		
Provided opportunity/time for facilitators to prepare for 10-minute session	☐	☐	☐		
Participants given an opportunity to be involved in discussion and ask questions	☐	☐	☐		
**Session 5: Have a go**					
**Duration of session:**					
**Materials**					
Facilitator curricula	☐	☐	☐		
'Have a go' cards (x2 sets) given out to participants	☐	☐	☐		
Roadmap and accessories displayed	☐	☐	☐		
A4 images: blood clot, bleed displayed	☐	☐	☐		

For peer review only - http://bmjopen.bmj.com/site/about/guidelines.xhtml

**Date:**					
**Start time:**					
**End time:**					
**Training day 3**					
	**Please tick as appropriate**			**If *absent* or *attempted* please provide reasons**	**Other comments**
	**Present**	**Absent**	**Attempted**		
A6 images: risk factors, risks I can/cannot change displayed	☐	☐	☐		
A4 and A6 images: health and wellbeing displayed	☐	☐	☐		
A4 images: dashboard displayed	☐	☐	☐		
Writing instruments given (sticky notes, flipchart paper)	☐	☐	☐		
**Content**					
Give instructions on delivery of 10-minute sessions	☐	☐	☐		
Inform participants of aim of practice delivery (i.e. familiarise self with MLAS, increase confidence)	☐	☐	☐		
Give instructions about giving feedback on facilitator behaviours (3 things you did well)	☐	☐	☐		

For peer review only - http://bmjopen.bmj.com/site/about/guidelines.xhtml

**Date:**					
**Start time:**					
**End time:**					
**Training day 3**					
	**Please tick as appropriate**			**If *absent* or *attempted* please provide reasons**	**Other comments**
	**Present**	**Absent**	**Attempted**		
Each facilitator given an opportunity to deliver their 10-minute session	☐	☐	☐		
Ensured everyone has written 3 positive facilitator behaviours that they saw on a sticky note and give to facilitator	☐	☐	☐		
Provided 3 positive facilitator behaviours and 1 'even better if…'	☐	☐	☐		
Encourage participants to share summaries and feedback on their experience	☐	☐	☐		
**Session 6: Reviewing confidence**					
**Duration of session:**					
**Materials**					
'How confident do I feel?' worksheet	☐	☐	☐		
**Content**					

For peer review only - http://bmjopen.bmj.com/site/about/guidelines.xhtml

**Date:**					
**Start time:**					
**End time:**					
**Training day 3**					
	**Please tick as appropriate**			**If *absent* or *attempted* please provide reasons**	**Other comments**
	**Present**	**Absent**	**Attempted**		
Explores participants' confidence levels to deliver MLAS	☐	☐	☐		
Explore what participants can do to increase their personal confidence to deliver MLAS	☐	☐	☐		
**Session 7: Quality development/Intervention fidelity**					
**Duration of session:**					
**Materials**					
Facilitator behaviours reflection toolkit	☐	☐	☐		
Quality development and intervention fidelity pathway	☐	☐	☐		
**Content**					
Recapped purpose of quality assurance	☐	☐	☐		
Highlighted the importance of self- and peer-reflection	☐	☐	☐		
Discussed facilitator behaviour reflection toolkit	☐	☐	☐		

For peer review only - http://bmjopen.bmj.com/site/about/guidelines.xhtml

**Date:**					
**Start time:**					
**End time:**					
**Training day 3**					
	**Please tick as appropriate**			**If *absent* or *attempted* please provide reasons**	**Other comments**
	**Present**	**Absent**	**Attempted**		
Discussed quality assurance pathway	☐	☐	☐		
Explored what intervention fidelity is					
Described the process for intervention fidelity assessment	☐	☐	☐		
Participants given an opportunity to be involved in discussion (e.g. by giving feedback) and ask questions	☐	☐	☐		
**Session 8: Summary, and post reflections, evaluation**					
**Duration of session:**					
**Materials**					
Training evaluation form given	☐	☐	☐		
Questions relating to MLAS flipchart	☐	☐	☐		
**Content**					

For peer review only

For peer review only - http://bmjopen.bmj.com/site/about/guidelines.xhtml

**Date:**					
**Start time:**					
**End time:**					
**Training day 3**					
	**Please tick as appropriate**			**If *absent* or *attempted* please provide reasons**	**Other comments**
	**Present**	**Absent**	**Attempted**		
Summary of Day 3 training by encouraging participants to share learning about what was covered today	☐	☐	☐		
Provided time for participants to complete training evaluation (day 3) form	☐	☐	☐		
Participants given an opportunity to check if their questions addressed, and to ask any further questions	☐	☐	☐		
Thanked everyone for attending and contributing	☐	☐	☐		
**END OF DAY 3 TRAINING**					

For peer review only - http://bmjopen.bmj.com/site/about/guidelines.xhtml

Training evaluation form

My Life After Stroke - Day 1 - Stroke

Date:__________________

Thank you for attending the Stroke day (Day 1) of the My Life After Stroke (MLAS) training. We would value your reflection on the training you have received today in the following areas. Please place a tick in the relevant box, indicating how much you agree or disagree with each of the following statements:

	Strongly agree	Agree	Neither agree nor disagree	Disagree	Strongly disagree
I am aware of the cognitive problems that can occur following stroke					
I am aware of some strategies that may help stroke survivors manage the effects of cognitive problems					
I have an understanding of the emotional problems that may follow a stroke					
I am aware of some strategies to support stroke survivors manage the effects of their emotional problems					
I have an appreciation of the effects having a stroke could put on relationships					
I am aware of some strategies that may help stroke survivors manage the effects on their relationships					
I have an awareness of communication problems following a stroke					
I have an awareness of how to communicate effectively with stroke survivors					

Continued overleaf…

For peer review only

Day 1 MLAS – Stroke evaluation continued…

	Strongly agree	Agree	Neither agree nor disagree	Disagree	Strongly disagree
I had the opportunity to ask questions and for these to be addressed					
I learnt something new today					

We would value your opinion on the training you attended today. Please answer the following questions:

Which were the three most helpful aspects of the training to you?<br>1.<br><br>2.<br><br>3.
Which were the three least helpful aspects of the training to you?<br>1.<br><br>2.<br><br>3.
Which part of the training did you find most enjoyable?
Please identify at least one way that the training could be improved?
Any other comments?

Thank you for completing this evaluation sheet

For peer review only

Training evaluation form

My Life After Stroke - Day 2 – Core training

Date:_________________

Thank you for attending the Core day (Day 2) of the My Life After Stroke (MLAS) training. We would value your reflection on the training you have received today in the following areas. Please place a tick in the relevant box, indicating how much you agree or disagree with each of the following statements:

	Strongly agree	Agree	Neither agree nor disagree	Disagree	Strongly disagree
I am aware of the aims of the MLAS programme					
I am aware of why MLAS has been developed					
My role as an MLAS facilitator is to:					
• Demonstrate a curious, non-judgemental approach towards participants					
• Support participants to explore their personal thoughts and feelings					
• Avoid giving specific advice and instead support participants to develop their own solutions and strategies to the challenges they face					
• Facilitate the groups awareness of sources of support and information					
• Promote the sharing of knowledge and ideas between participants and sign post where appropriate					
I have an understanding of the theories that underpin the MLAS programme					
I have an understanding of how these theories have been integrated practically into the MLAS programme					

Continued overleaf…

For peer review only

Day 2 MLAS Core evaluation continued…

	Strongly agree	Agree	Neither agree nor disagree	Disagree	Strongly disagree
I had the opportunity to ask questions and for these to be addressed					
I have learnt new skills					
I believe I can apply the facilitation skills					
I understand the importance of quality assurance					

I have been given an MLAS curriculum. **Yes / No** (delete as appropriate)

We would value your opinion on the training you attended today. Please answer the following questions:

Which were the three most helpful aspects of the training to you?<br>1.<br>2.<br>3.
Which were the three least helpful aspects of the training to you?<br>1.<br>2.<br>3.
Which part of the training did you find most enjoyable?
Please identify at least one way that the training could be improved?
Any other comments?

Thank you for completing this evaluation sheet

For peer review only

Training evaluation form

My Life After Stroke - Day 3 – MLAS-specific training

Date:_________________

Thank you for attending the MLAS-specific day (Day 3) of the My Life After Stroke (MLAS) training. We would value your reflection on the training you have received today in the following areas. Please place a tick in the relevant box, indicating how much you agree or disagree with each of the following statements:

	Strongly agree	Agree	Neither agree nor disagree	Disagree	Strongly disagree
I am aware of how to use the MLAS curriculum					
I have an understanding of the content of the MLAS programme					
I have an understanding of how MLAS can be delivered					
I had the opportunity to see the resources that are used during the MLAS programme					
I found the 'having a go' session useful					
I feel able to begin to plan delivering MLAS					
I feel confident that I will be able to facilitate MLAS					
I had the opportunity to ask questions and for these to be addressed					
I have learnt new skills					
I believe I can apply the skills					

I had the opportunity to deliver a section of the curriculum:

Yes / No / Don't know (please circle)

I received a copy of the Reflection Toolkit:

Yes / No / Don't know (please circle)

Continued overleaf…

For peer review only

Day 3 MLAS-specific evaluation continued…

We would value your opinion on the training you attended today. Please answer the following questions:

Which were the three most helpful aspects of the training to you?<br>1.<br><br>2.<br><br>3.
Which were the three least helpful aspects of the training to you?<br>1.<br><br>2.<br><br>3.
Which part of the training did you find most enjoyable?
Please identify at least one way that the training could be improved?
Any other comments?

Thank you for completing this evaluation sheet

For peer review only

**Assessor's name:**					
**Group/facilitator ID:**					
**Date:**					
**Week 1: Group session 1, Stroke Journey (2.5 hours, with breaks)**					
**Section A: Welcome/introduction**					
**Facilitator(s):**					
**Start time:**					
**End time:**					
	**Tick as appropriate**				
	**Present**	**Absent**	**Partially done**	**If *partially done*, please provide reasons (e.g. ran out of time, forgot, deemed unnecessary)**	**Other comments**
Content					
A1) Welcome/introductions (including housekeeping details, course handbook, course background)	☐	☐	☐		
A2) Introduction to the guidelines for working together as a group	☐	☐	☐		
A3) Opportunity for participants to ask questions	☐	☐	☐		
A4) Introduction to communication aids	☐	☐	☐		
A5) Participants' important questions	☐	☐	☐		
Materials					
A6) Flipchart – names	☐	☐	☐		
A7) Flipcharts – what would you like to get from the course?; what is your important question?	☐	☐	☐		
A8) Flipchart – working together as a group	☐	☐	☐		
Key facilitator behaviours for welcome/introduction					
A9) Uses and refers to participants' comments/quotes	☐	☐	☐		

For peer review only - http://bmjopen.bmj.com/site/about/guidelines.xhtml

**Section B: Stroke story**					
**Facilitator(s):**					
**Start time:**					
**End time:**					
	**Tick as appropriate**				
	**Present**	**Absent**	**Partially done**	**If *partially done*, please provide reasons (e.g. ran out of time, forgot, deemed unnecessary)**	**Other comments**
Content					
B1) Stroke story/introduction to narrative approach (including roadmap)	☐	☐	☐		
B2) Eliciting skills, strengths and strategies from participants	☐	☐	☐		
Materials					
B3) Flipchart – skills and strategies	☐	☐	☐		
Key facilitator behaviours for stroke story					
B4) Uses resources and/or participant handbooks appropriately to engage participants and enhance their understanding of the session's key outcomes	☐	☐	☐		
B5) Facilitates people to share their stories about attempts to manage their condition	☐	☐	☐		
B6) Appropriately adapts communication/environment/session delivery to meet the needs of the stroke survivors	☐	☐	☐		

For peer review only

For peer review only - http://bmjopen.bmj.com/site/about/guidelines.xhtml

**Section C: What is stroke?**					
**Facilitator(s):**					
**Start time:**					
**End time:**					
	**Tick as appropriate**				
	**Present**	**Absent**	**Partially done**	**If *partially done*, please provide reasons (e.g. ran out of time, forgot, deemed unnecessary)**	**Other comments**
Content					
C1) What is stroke (including impacts of stroke on individual – physically, emotionally, psychologically and socially)?	☐	☐	☐		
C2) Discussed neuroplasticity (the brain's ability to create new neural pathways and relearn skills	☐	☐	☐		
Materials					
C3) Flipchart – body outline	☐	☐	☐		
C4) Record of 'what is stroke?' (i.e. written on body outline/sticky notes)	☐	☐	☐		
C5) Record of 'effects of stroke on body/person' (i.e. written on body outline/sticky notes)	☐	☐	☐		
Key facilitator behaviours for what is stroke					
C6) Prompts participant to talk about their thoughts and explores misconceptions and gaps in knowledge	☐	☐	☐		
C7) Uses resources and/or participant handbooks appropriately to engage participants and enhance their understanding of the session's key outcomes	☐	☐	☐		

For peer review only

For peer review only - http://bmjopen.bmj.com/site/about/guidelines.xhtml

**Section D: Stroke passenger**					
**Facilitator(s):**					
**Start time:**					
**End time:**					
	**Tick as appropriate**				
	**Present**	**Absent**	**Partially done**	**If *partially done*, please provide reasons (e.g. ran out of time, forgot, deemed unnecessary)**	**Other comments**
Content					
D1) Discussed 'Stroke passenger' analogy	☐	☐	☐		
D2) Eliciting feelings and emotions about stroke	☐	☐	☐		
D3) Provided opportunity for separate carer/stroke survivor discussions	☐	☐	☐		
D4) Discussing skills and coping strategies for living with stroke passenger	☐	☐	☐		
Materials					
D5) Flipchart – how do you feel/think about sharing your life with stroke (carers)?	☐	☐	☐		
D6) Flipchart – how do you feel/think about sharing your life with stroke (stroke survivors)?	☐	☐	☐		
Key facilitator behaviours for stroke passenger					
D7) Prompts participants to express and explore their feelings about the condition or behaviour	☐	☐	☐		
D8) Facilitates people to share their stories about attempts to manage their condition	☐	☐	☐		
D9) Appropriately adapts communication/environment/session delivery to meet the needs of the stroke survivors	☐	☐	☐		
D10) Works as a team with co-facilitator	☐	☐	☐		

For peer review only - http://bmjopen.bmj.com/site/about/guidelines.xhtml

Section E: What will I do now?					
**Facilitator(s):**					
**Start time:**					
**End time:**					
	**Tick as appropriate**				
	**Present**	**Absent**	**Partially done**	**If *partially done*, please provide reasons (e.g. ran out of time, forgot, deemed unnecessary)**	**Other comments**
Content					
E1) Session summary/recap of session key outcomes	☐	☐	☐		
E2) 'What will I do now?' worksheet	☐	☐	☐		
Materials					
E3) 'What will I do now?' worksheet in handbook	☐	☐	☐		
Key facilitator behaviours for what will I do now					
E4) Uses resources and/or participant handbooks appropriately to engage participants and enhance their understanding of the session's key outcomes	☐	☐	☐		
E5) Prompts group to summarise their key messages from the session.	☐	☐	☐		
E6) Prompts participants to talk about what they are going to do as a result of the session.	☐	☐	☐		

For peer review only

Overall facilitator behaviours					
	**Tick as appropriate**				
	**Present**	**Absent**	**Partially done**	**If *partially done*, please provide reasons (e.g. ran out of time, forgot, deemed unnecessary)**	**Other comments**
1. Uses non-judgmental statements in response to participants' verbal utterances	☐	☐	☐		
2. The facilitator seeks clarification of participants contribution	☐	☐	☐		
3. Avoids giving their own opinion	☐	☐	☐		
4. Encourages the group to discuss/answer their own question	☐	☐	☐		
5. Engages participants using rapport building skills	☐	☐	☐		
6. Notices tone/dynamics within the group, acknowledges these and uses them to manage the group	☐	☐	☐		
7. The facilitator uses strategies to manage time within session	☐	☐	☐		
8. Uses active listening skills and open body language to facilitate group/participant talk	☐	☐	☐		
9. Appropriately adapts delivery to meet the needs of the stroke survivors	☐	☐	☐		
10. Works as a team with co-facilitator	☐	☐	☐		

For peer review only

For peer review only - http://bmjopen.bmj.com/site/about/guidelines.xhtml

Assessor comments/notes

For peer review only

For peer review only - http://bmjopen.bmj.com/site/about/guidelines.xhtml

**Assessor's name:**					
**Group/facilitator ID:**					
**Date:**					
**Week 2/3 (circle as appropriate): Managing health and wellbeing (2.5 hours, with breaks)**					
**Section A: Welcome back [20 mins]**					
**Facilitator(s):**					
**Start time:**					
**End time:**					
	**Tick as appropriate**				
	**Present**	**Absent**	**Partially done**	**If *partially done*, please provide reasons (e.g. ran out of time, forgot, deemed unnecessary)**	**Other comments**
Content					
A1) Welcome (including housekeeping details, and ground rules)	☐	☐	☐		
A2) Session outline	☐	☐	☐		
A3) Introduction/recap of managing health and wellbeing handbook and roadmap <u>or</u> roadblocks handbook and roadmap	☐	☐	☐		
A4) Previous week's worksheets; stories and reflections from past week	☐	☐	☐		
A5) Opportunity for participants to ask questions	☐	☐	☐		
Materials					
A6) Roadmap	☐	☐	☐		
A7) Previous week's (relevant) flipcharts on view	☐	☐	☐		
Key facilitator behaviours for welcome					
A8) Uses resources and participant handbooks appropriately to engage participants and enhance understanding of the session's key outcomes	☐	☐	☐		
A9) Prompts the participants to reflect on last week's session and activities	☐	☐	☐		

For peer review only - http://bmjopen.bmj.com/site/about/guidelines.xhtml

**Section B: Managing health and wellbeing [40 mins]**					
**Facilitator(s):**					
**Start time:**					
**End time:**					
	**Tick as appropriate**				
	**Present**	**Absent**	**Partially done**	**If *partially done*, please provide reasons (e.g. ran out of time, forgot, deemed unnecessary)**	**Other comments**
Content					
B1) Risk factors for stroke	☐	☐	☐		
B2) Ways to manage health and wellbeing	☐	☐	☐		
Materials					
B3) A6 headings: 'risks I can change' and 'risks I cannot change'	☐	☐	☐		
B4) A6 images: risk factors	☐	☐	☐		
B5) A4 headings: health and wellbeing	☐	☐	☐		
B6) A6 images: health and wellbeing	☐	☐	☐		
Key facilitator behaviours for managing health and wellbeing					
B7) Facilitates people to share their stories about attempts to manage their condition	☐	☐	☐		
B8) Uses resources and participant handbooks appropriately to engage participants and enhance understanding of the session's key outcomes	☐	☐	☐		
B9) Prompts participant to talk about their thoughts and explores misconceptions and gaps in knowledge	☐	☐	☐		
B10) Encourages the group to discuss/answer their own questions	☐	☐	☐		
B11) Works as a team with co-facilitator	☐	☐	☐		

For peer review only

**Section C: Making changes [15 mins]**					
**Facilitator(s):**					
**Start time:**					
**End time:**					
	**Tick as appropriate**				
	**Present**	**Absent**	**Partially done**	**If *partially done*, please provide reasons (e.g. ran out of time, forgot, deemed unnecessary)**	**Other comments**
Content					
C1) Elicits feelings and reactions to change	☐	☐	☐		
C2) Discussion of pros and cons of changes	☐	☐	☐		
Materials					
C3) Flipchart – pros and cons grid	☐	☐	☐		
Key facilitator behaviours for making changes					
C4) Prompts participants to discuss their thoughts about possible changes to self-management.	☐	☐	☐		
C5) Prompts participants to review the impact of possible choices on their future health.	☐	☐	☐		
C6) Prompts or allows participants to express and explore their feelings about the condition or behaviour	☐	☐	☐		

For peer review only

For peer review only - http://bmjopen.bmj.com/site/about/guidelines.xhtml

**Section D: Making a plan [35 mins]**					
**Facilitator(s):**					
**Start time:**					
**End time:**					
	**Tick as appropriate**				
	**Present**	**Absent**	**Partially done**	**If *partially done*, please provide reasons (e.g. ran out of time, forgot, deemed unnecessary)**	**Other comments**
Content					
D1) Identifying challenges to planning, problem-solving	☐	☐	☐		
D2) Making a SMART plan	☐	☐	☐		
Materials					
D3) A4 resource: 'My action plan'	☐	☐	☐		
D4) 'My action plan' in handbook	☐	☐	☐		
Key facilitator behaviours for making a plan					
D5) Prompts participants to discuss their thoughts about possible changes to self-management.	☐	☐	☐		
D6) Prompts the participants to reflect on their goals/plans	☐	☐	☐		
D7) Prompts the individual or group to problem solve possible barriers to change (e.g. their desired changes or possible barriers to self-management)	☐	☐	☐		
D8) Encourages the group to discuss/answer their own questions	☐	☐	☐		
D9) Prompts group to summarise key messages from the session	☐	☐	☐		

For peer review only

For peer review only - http://bmjopen.bmj.com/site/about/guidelines.xhtml

**Section E: What will I do now? [10 mins]**					
**Facilitator(s):**					
**Start time:**					
**End time:**					
	**Tick as appropriate**				
	**Present**	**Absent**	**Partially done**	**If *partially done*, please provide reasons (e.g. ran out of time, forgot, deemed unnecessary)**	**Other comments**
Content					
E1) Session summary provided	☐	☐	☐		
E2) 'What will I do now?' worksheet	☐	☐	☐		
Materials					
E3) 'What will I do now?' worksheet in handbook	☐	☐	☐		
Key facilitator behaviours for what will I do now					
E4) Uses resources and participant handbooks appropriately to engage participants and enhance understanding of the session's key outcomes	☐	☐	☐		
E5) Prompts participants to talk about what they are going to do as a result of the session	☐	☐	☐		
E6) Prompts group to summarise their key messages from the session.	☐	☐	☐		

For peer review only

For peer review only - http://bmjopen.bmj.com/site/about/guidelines.xhtml

Overall facilitator behaviours					
	**Tick as appropriate**				
	**Present**	**Absent**	**Partially done**	**If *partially done*, please provide reasons (e.g. ran out of time, forgot, deemed unnecessary)**	**Other comments**
1. Uses non-judgmental statements in response to participants' verbal utterances	☐	☐	☐		
2. The facilitator seeks clarification of participants contribution	☐	☐	☐		
3. Avoids giving their own opinion	☐	☐	☐		
4. Encourages the group to discuss/answer their own questions and/or elicits suggestions from participants	☐	☐	☐		
5. Engages participants using rapport building skills	☐	☐	☐		
6. Notices tone/dynamics within the group, acknowledges these and uses them to manage the group	☐	☐	☐		
7. The facilitator uses strategies to manage time within session	☐	☐	☐		
8. Uses open questions, reflections, appropriate silences and open body language to facilitate group/participant talk	☐	☐	☐		
9. Adapts communication/environment/ session delivery to meet the needs of the stroke survivors	☐	☐	☐		
10. Works as a team with co-facilitator	☐	☐	☐		

For peer review only - http://bmjopen.bmj.com/site/about/guidelines.xhtml

Assessor comments/notes

For peer review only

For peer review only - http://bmjopen.bmj.com/site/about/guidelines.xhtml

**Assessor's name:**					
**Group/facilitator ID:**					
**Date:**					
Week 2/3 (circle as appropriate): Roadblocks (2.5 hrs, with breaks)					
**Section A: Welcome back**					
**Facilitator(s):**					
**Start time:**					
**End time:**					
	**Tick as appropriate**				
	**Present**	**Absent**	**Partially done**	**If *partially done*, please provide reasons (e.g. ran out of time, forgot, deemed unnecessary)**	**Other comments**
Content					
A1) Welcome (including housekeeping details, and ground rules)	☐	☐	☐		
A2) Session outline	☐	☐	☐		
A3) Introduction/recap of managing health and wellbeing handbook and roadmap <u>or</u> roadblocks handbook and roadmap	☐	☐	☐		
A4) Previous week's worksheets; stories and reflections from past week	☐	☐	☐		
A5) Opportunity for participants to ask questions					
Materials					
A6) Roadmap	☐	☐	☐		
A7) Previous week's (relevant) flipcharts on view	☐	☐	☐		
Key facilitator behaviours for welcome back					
A8) Uses resources and participant handbooks appropriately to engage participants and enhance understanding of the session's key outcomes	☐	☐	☐		
A9) Prompts the participants to reflect on last week's session and activities	☐	☐	☐		

For peer review only - http://bmjopen.bmj.com/site/about/guidelines.xhtml

**Section B: Roadblocks on my journey**					
**Facilitator(s):**					
**Start time:**					
**End time:**					
	**Tick as appropriate**				
	**Present**	**Absent**	**Partially done**	**If *partially done*, please provide reasons (e.g. ran out of time, forgot, deemed unnecessary)**	**Other comments**
Content					
B1) Fictional character facing loss of confidence after stroke (using STOP sign)	☐	☐	☐		
B2) Eliciting thoughts, feelings, sensations, or behaviours as a response to loss of confidence	☐	☐	☐		
B3) Discuss what might help the fictional character, and impact of this on thoughts, feelings, sensations, or behaviours (using GO sign)	☐	☐	☐		
B4) 'What do my roadblocks look like?' worksheet	☐	☐	☐		
Materials					
B5) Roadmap	☐	☐	☐		
B6) Flipcharts – STOP and GO	☐	☐	☐		

For peer review only

Key facilitator behaviours for roadblocks					
	**Tick as appropriate**				
	**Present**	**Absent**	**Partially done**	**If *partially done*, please provide reasons (e.g. ran out of time, forgot, deemed unnecessary)**	**Other comments**
B7) Facilitates people to share their stories about attempts to manage their condition, and the challenges/roadblocks they face.	☐	☐	☐		
B8) Prompts or allows participants to express and explore their feelings about (the condition or behaviour) during the session	☐	☐	☐		
B9) Encourages the group to discuss/answer their own questions	☐	☐	☐		
B10) Uses and refers to participants' comments/quotes	☐	☐	☐		
B11) Prompts the individual or group to problem solve possible barriers to change (e.g. their desired changes or possible barriers to self-management)	☐	☐	☐		
B12) Prompts participants to review the impact of possible choices on their future health.	☐	☐	☐		

For peer review only

For peer review only - http://bmjopen.bmj.com/site/about/guidelines.xhtml

**Section C: Working it out**					
**Facilitator(s):**					
**Start time:**					
**End time:**					
	**Tick as appropriate**				
	**Present**	**Absent**	**Partially done**	**If *partially done*, please provide reasons (e.g. ran out of time, forgot, deemed unnecessary)**	**Other comments**
Content					
C1) Problems stroke survivors could face Next steps (including session's key outcomes, 'what will I do now?' worksheet, and follow-up appointments)	☐	☐	☐		
C2) Introduce problem-solving approach	☐	☐	☐		
C3) Use fictional character to choose a problem and apply problem-solving approach	☐	☐	☐		
C4) How decisions may be made on the best course of action for solving the problem	☐	☐	☐		
C5) What to do when problems aren't solvable now	☐	☐	☐		
C6) 'Working it out' worksheet	☐	☐	☐		
Materials					
C7) Flipcharts with headings: 1) in my power to solve/change or not in my power to solve/change; 2) 'what could help'; 3) solution: immediate results/long-term results	☐	☐	☐		
C8) Previous flipcharts (e.g. what are my roadblocks; skills and strategies toolkit)	☐	☐	☐		

For peer review only - http://bmjopen.bmj.com/site/about/guidelines.xhtml

Key facilitator behaviours for working it out					
	**Tick as appropriate**				
	**Present**	**Absent**	**Partially done**	**If *partially done*, please provide reasons (e.g. ran out of time, forgot, deemed unnecessary)**	**Other comments**
C9) Uses resources and participant handbooks appropriately to engage participants and enhance understanding of the session's key outcomes	☐	☐	☐		
C10) Uses and refers to participants' comments/quotes	☐	☐	☐		
C11) Prompts participants to discuss their thoughts about possible changes to self-management.	☐	☐	☐		
C12) Prompts participants to review the impact of possible choices on their future health.	☐	☐	☐		
C13) Prompts the individual or group to problem solve possible barriers to change (e.g. their desired changes or possible barriers to self-management)	☐	☐	☐		

For peer review only - http://bmjopen.bmj.com/site/about/guidelines.xhtml

**Section D: What will I do now**					
**Facilitator(s):**					
**Start time:**					
**End time:**					
	**Tick as appropriate**				
	**Present**	**Absent**	**Partially done**	**If *partially done*, please provide reasons (e.g. ran out of time, forgot, deemed unnecessary)**	**Other comments**
Content					
D1) Recap key outcomes	☐	☐	☐		
D2) 'What will I do now?' worksheet	☐	☐	☐		
Materials					
D3) 'What will I do now?' worksheet	☐	☐	☐		
Key facilitator behaviours for what will I do now					
D4) Uses resources and participant handbooks appropriately to engage participants and enhance understanding of the session's key outcomes	☐	☐	☐		
D5) Prompts participants to talk about what they are going to do as a result of the session.	☐	☐	☐		

For peer review only

For peer review only - http://bmjopen.bmj.com/site/about/guidelines.xhtml

Overall facilitator behaviours					
	**Tick as appropriate**				
	**Present**	**Absent**	**Partially done**	**If *partially done*, please provide reasons (e.g. ran out of time, forgot, deemed unnecessary)**	**Other comments**
1. Prompts group to summarise their key messages from the session.	☐	☐	☐		
2. The facilitator seeks clarification of participants contribution	☐	☐	☐		
3. Avoids giving their own opinion	☐	☐	☐		
4. Encourages the group to discuss/answer their own questions and/or elicits suggestions from participants	☐	☐	☐		
5. Engages participants using rapport building skills	☐	☐	☐		
6. Notices tone/dynamics within the group, acknowledges these and uses them to manage the group	☐	☐	☐		
7. The facilitator uses strategies to manage time within session	☐	☐	☐		
8. Uses open questions, reflections, appropriate silences and open body language to facilitate group/participant talk	☐	☐	☐		
9. Adapts communication/environment/ session delivery to meet the needs of the stroke survivors	☐	☐	☐		
10. Works as a team with co-facilitator	☐	☐	☐		

For peer review only

For peer review only - http://bmjopen.bmj.com/site/about/guidelines.xhtml

Assessor comments/notes

For peer review only

For peer review only - http://bmjopen.bmj.com/site/about/guidelines.xhtml

**Assessor's name:**					
**Group/facilitator ID:**					
**Date:**					
**Week 4: Moving forward on my journey (2.5 hours, with breaks)**					
**Section A: Welcome back**					
**Facilitator(s):**					
**Start time:**					
**End time:**					
	**Tick as appropriate**				
	**Present**	**Absent**	**Partially done**	**If *partially done*, please provide reasons (e.g. ran out of time, forgot, deemed unnecessary)**	**Other comments**
Content					
A1) Welcome (including housekeeping details, handbook and ground rules)	☐	☐	☐		
A2) Session outline	☐	☐	☐		
A3) Previous week's action plan/working it out worksheets	☐	☐	☐		
A4) Recap roadmap	☐	☐	☐		
Materials					
A5) Roadmap	☐	☐	☐		
A6) Previous week's (relevant) flipcharts on view	☐	☐	☐		
A7) Moving forward on my journey handbook	☐	☐	☐		
Key facilitator behaviours for welcome back					
A8) Re-engages participants in the journey analogy and resources	☐	☐	☐		
A9) Prompts the participants to reflect on last week's session and activities	☐	☐	☐		

For peer review only - http://bmjopen.bmj.com/site/about/guidelines.xhtml

**Section B: Your relationships**					
**Facilitator(s):**					
**Start time:**					
**End time:**					
	**Tick as appropriate**				
	**Present**	**Absent**	**Partially done**	**If *partially done*, please provide reasons (e.g. ran out of time, forgot, deemed unnecessary)**	**Other comments**
Content					
B1) Ways relationships can be affected by stroke	☐	☐	☐		
B2) Problem-solving exercise in relation to a relationship change	☐	☐	☐		
Materials					
B3) Flipcharts – how can relationships change (one for stroke survivors, one for carers/supporters as applicable)	☐	☐	☐		
B4) Flipcharts – 'what is the problem exactly'; 'what could help'; 'solution: immediate results/long-term results'	☐	☐	☐		

For peer review only

For peer review only - http://bmjopen.bmj.com/site/about/guidelines.xhtml

Key facilitator behaviours for your relationships					
	**Tick as appropriate**				
	**Present**	**Absent**	**Partially done**	**If *partially done*, please provide reasons (e.g. ran out of time, forgot, deemed unnecessary)**	**Other comments**
B5) Prompts or allows participants to express and explore their feelings about (the condition or behaviour) during the session	☐	☐	☐		
B6) Uses and refers to participants' comments/quotes	☐	☐	☐		
B7) Prompts the individual or group to problem solve possible barriers to change (e.g. their desired changes or possible barriers to self-management)	☐	☐	☐		
B8) Prompts participants to review the impact of possible choices on their future health.	☐	☐	☐		
B9) Adapts communication, environment, or session delivery to meet the needs of the stroke survivors	☐	☐	☐		

For peer review only

For peer review only - http://bmjopen.bmj.com/site/about/guidelines.xhtml

**Section C: Revisiting your stroke journey**					
**Facilitator(s):**					
**Start time:**					
**End time:**					
	**Tick as appropriate**				
	**Present**	**Absent**	**Partially done**	**If *partially done*, please provide reasons (e.g. ran out of time, forgot, deemed unnecessary)**	**Other comments**
Content					
C1) Recap the roadmap/journey analogy	☐	☐	☐		
C2) Summary of key messages from each session (e.g. session outcomes/personal take-home messages/skills and strategies discussed during the course)	☐	☐	☐		
C3) Elicit feelings about moving forward from MLAS	☐	☐	☐		
Materials					
C4) Flipchart – 'skills and strategies toolkit'	☐	☐	☐		
Key facilitator behaviours for revisiting your stroke journey					
C5) Facilitates the group's recapping of the journey analogy (e.g. prompts group to summarise their key messages from the session)	☐	☐	☐		
C6) Prompts participants to reflect on where they feel they are now	☐	☐	☐		

For peer review only - http://bmjopen.bmj.com/site/about/guidelines.xhtml

**Section D: How will you move forward?**					
**Facilitator(s):**					
**Start time:**					
**End time:**					
	**Tick as appropriate**				
	**Present**	**Absent**	**Partially done**	**If *partially done*, please provide reasons (e.g. ran out of time, forgot, deemed unnecessary)**	**Other comments**
Content					
D1) Discussion of 'warning lights' for physical/emotional health and prevention of these	☐	☐	☐		
D2) Loneliness from the perspective of the fictional character (may also use problem-solving exercise)	☐	☐	☐		
D3) The directory	☐	☐	☐		
Materials					
D4) A4 dashboard images	☐	☐	☐		
D5) Flipchart - 'Skills and strategies toolkit'	☐	☐	☐		
D6) A4 directory	☐	☐	☐		
Key facilitator behaviours for how will you move forward					
D7) Prompts or allows participants to express and explore their feelings about the condition or behaviour	☐	☐	☐		
D8) Encourages the group to discuss/answer their own questions	☐	☐	☐		
D9) Uses and refers to participants' comments/quotes	☐	☐	☐		

For peer review only - http://bmjopen.bmj.com/site/about/guidelines.xhtml

**Section E: Next steps**					
**Facilitator(s):**					
**Start time:**					
**End time:**					
	**Tick as appropriate**				
	**Present**	**Absent**	**Partially done**	**If *partially done*, please provide reasons (e.g. ran out of time, forgot, deemed unnecessary)**	**Other comments**
Content					
E1) Recap session's key outcomes	☐	☐	☐		
E2) 'What will I do now?' worksheet	☐	☐	☐		
E3) Organise follow-up appointments	☐	☐	☐		
Materials					
E4) 'What will I do now?' worksheet in the handbook	☐	☐	☐		
E5) Flipchart – 'what would you like to get from the course?/What is your important question?'	☐	☐	☐		
Key facilitator behaviours for next steps					
E6) Prompts group to summarise their key messages from the session.	☐	☐	☐		
E7) Prompts participants to talk about what they are going to do as a result of the course.	☐	☐	☐		
E8) Encourages the group to discuss/answer their own questions	☐	☐	☐		

For peer review only

For peer review only - http://bmjopen.bmj.com/site/about/guidelines.xhtml

Overall facilitator behaviours					
	**Tick as appropriate**				
	**Present**	**Absent**	**Partially done**	**If *partially done*, please provide reasons (e.g. ran out of time, forgot, deemed unnecessary)**	**Other comments**
1. Uses non-judgmental statements in response to participants' verbal utterances	☐	☐	☐		
2. The facilitator seeks clarification of participants contribution	☐	☐	☐		
3. Avoids giving their own opinion	☐	☐	☐		
4. Encourages the group to discuss/answer their own questions and/or elicits suggestions from participants	☐	☐	☐		
5. Engages participants using rapport building skills	☐	☐	☐		
6. Notices tone/dynamics within the group, acknowledges these and uses them to manage the group	☐	☐	☐		
7. The facilitator uses strategies to manage time within session	☐	☐	☐		
8. Uses open questions, reflections, appropriate silences and open body language to facilitate group/participant talk	☐	☐	☐		
9. Adapts communication/environment/ session delivery to meet the needs of the stroke survivors	☐	☐	☐		
10. Works as a team with co-facilitator	☐	☐	☐		

For peer review only

Assessor comments/notes

For peer review only

For peer review only - http://bmjopen.bmj.com/site/about/guidelines.xhtml

IPCAS trial process evaluation checklist
MLAS facilitator delivery - DESMOND Observation Tool Record Sheet
Group Session 1

Instructions: DOT at 10s (i.e. at the second bleep)

Session A: Welcome/Introduction (30 min)

Facilitator's name:

Assessor's name:

Start time:

End time:

Facilitator talk	Participant talk	Other
Total A:	Total B:	Total C:
Total score= A÷(A+B+C)*100=		

Session B: Stroke story (20 min)

Facilitator's name:

Assessor's name:

Start time:

End time:

Facilitator talk	Participant talk	Other
Total A:	Total B:	Total C:
Total score= A÷(A+B+C)*100=		

For peer review only

IPCAS trial process evaluation checklist

Session C: What is stroke? (25 min)

Facilitator's name:

Assessor's name:

Start time:

End time:

Facilitator talk	Participant talk	Other
Total A:	Total B:	Total C:
Total score= A÷(A+B+C)*100=		

Session D: Stroke passenger (35 min)

Facilitator's name:

Assessor's name:

Start time:

End time:

Facilitator talk	Participant talk	Other
Total A:	Total B:	Total C:
Total score= A÷(A+B+C)*100=		

Session E: What will I do now? (10 min)

Facilitator

Start time:

End time:

Facilitator talk	Participant talk	Other

For peer review only

IPCAS trial process evaluation checklist

Total A:	Total B:	Total C:
Total score= A÷(A+B+C)*100=		

For peer review only

IPCAS trial process evaluation checklist

MLAS facilitator delivery - DESMOND Observation Tool Record Sheet

Group Session 2/3 (circle as appropriate): Managing Health and Wellbeing

Instructions: DOT at 10s (i.e. at the second bleep)

Session A: Welcome back, recap, and introduction (20 min)

Facilitator's name:

Assessor's name:

Start time:

End time:

Facilitator talk	Participant talk	Other
Total A:	Total B:	Total C:
Total score= A÷(A+B+C)*100=		

Session B: Managing health and wellbeing (40 min)

Facilitator's name:

Assessor's name:

Start time:

End time:

Facilitator talk	Participant talk	Other
Total A:	Total B:	Total C:
Total score= A÷(A+B+C)*100=		

For peer review only

IPCAS trial process evaluation checklist

Session C: Making changes (15 min)

Facilitator's name:

Assessor's name:

Start time:

End time:

Facilitator talk	Participant talk	Other
Total A:	Total B:	Total C:
Total score= A÷(A+B+C)*100=		

Session D: Making a plan (35 min)

Facilitator's name:

Assessor's name:

Start time:

End time:

Facilitator talk	Participant talk	Other
Total A:	Total B:	Total C:
Total score= A÷(A+B+C)*100=		

Session E: What will I do now? (10 min)

Facilitator

Start time:

End time:

Facilitator talk	Participant talk	Other

For peer review only

IPCAS trial process evaluation checklist

Total A:	Total B:	Total C:
Total score= A÷(A+B+C)*100=		

For peer review only

IPCAS trial process evaluation checklist
MLAS facilitator delivery - DESMOND Observation Tool Record Sheet
Group Session 2/3 (circle as appropriate): Roadblocks

Instructions: DOT at 10s (i.e. at the second bleep)

Session A: Welcome back and introduction (20 min)

Facilitator's name:

Assessor's name:

Start time:

End time:

Facilitator talk	Participant talk	Other
Total A:	Total B:	Total C:
Total score= A÷(A+B+C)*100=		

Session B: Roadblocks on my journey (45 min)

Facilitator's name:

Assessor's name:

Start time:

End time:

Facilitator talk	Participant talk	Other
Total A:	Total B:	Total C:
Total score= A÷(A+B+C)*100=		

For peer review only

IPCAS trial process evaluation checklist

Session C: Working it out (45 min)

Facilitator's name:

Assessor's name:

Start time:

End time:

Facilitator talk	Participant talk	Other
Total A:	Total B:	Total C:
Total score= A÷(A+B+C)*100=		

Session D: What will I do now? (10 min)

Facilitator's name:

Assessor's name:

Start time:

End time:

Facilitator talk	Participant talk	Other
Total A:	Total B:	Total C:
Total score= A÷(A+B+C)*100=		

For peer review only

IPCAS trial process evaluation checklist
MLAS facilitator delivery - DESMOND Observation Tool Record Sheet
Group Session 4: Moving Forward on my Journey

Instructions: DOT at 10s (i.e. at the second bleep)

Session A: Welcome back, recap, and introduction (20 min)

Facilitator's name:
Assessor's name:
Start time:
End time:

Facilitator talk	Participant talk	Other
Total A:	Total B:	Total C:
Total score= A÷(A+B+C)*100=		

Session B: Your relationships (45 min)

Facilitator's name:
Assessor's name:
Start time:
End time:

Facilitator talk	Participant talk	Other
Total A:	Total B:	Total C:
Total score= A÷(A+B+C)*100=		

For peer review only

IPCAS trial process evaluation checklist

Session C: Revisiting your stroke journey (10 min)

Facilitator’s name:

Assessor’s name:

Start time:

End time:

Facilitator talk	Participant talk	Other
Total A:	Total B:	Total C:
Total score= A÷(A+B+C)*100=		

Session D: How will you move forward? (30 min)

Facilitator’s name:

Assessor’s name:

Start time:

End time:

Facilitator talk	Participant talk	Other
Total A:	Total B:	Total C:
Total score= A÷(A+B+C)*100=		

Session E: What will I do now? (10 min)

Facilitator

Start time:

End time:

Facilitator talk	Participant talk	Other

For peer review only

IPCAS trial process evaluation checklist

Total A:	Total B:	Total C:
Total score= A÷(A+B+C)*100=		

For peer review only

Thank you for attending **Individual appointment 1**.

Please tick (✓) **agree, disagree, don't know** or **does not apply** for each of the statements below.

	Agree	Disagree	Don't know	Does not apply
1. The facilitator explained **'My life after stroke'** to me.				
2. The facilitator invited me to **ask questions** about 'My life after stroke'.				
3. I understand what **'My life after stroke'** is.				
4. I felt I could talk about what life was like **before and after** my stroke.				
5. The facilitator invited me to share any **problems I might face** in attending the course.				
6. I had the chance to talk about **ways to address** the problems I might face in attending the course.				
7. The facilitator invited my carer to come to my appointment.				
8. The facilitator invited my carer to **share experiences** if they wanted to.				

Please turn over.

For peer review only - http://bmjopen.bmj.com/site/about/guidelines.xhtml

Please write your **comments** below:

For peer review only

For peer review only - http://bmjopen.bmj.com/site/about/guidelines.xhtml

Thank you for attending **Stroke Journey**.

Please tick (✓) **agree, disagree, don't know** or **does not apply** for each of the statements below.

	Agree	Disagree	Don't know	Does not apply
1. I felt like I could contribute to the session if I wanted to.				
2. I felt like I could participate in the session activities.				
3. The session included a discussion about my **stroke story**.				
4. I had a chance to think about where I am on my **journey** since my stroke.				
5. I know what a stroke is.				
6. I know how a stroke can affect me.				
7. I understand what **neuroplasticity** is.				
8. I have a **strategy** that can help me deal with the effects of my stroke.				
9. The session included a discussion about **feelings** after having a stroke.				

Please turn over.

For peer review only - http://bmjopen.bmj.com/site/about/guidelines.xhtml

Please tick ✓ **agree**, **disagree**, **don't know** or **does not apply** for each of the statements below.

	Agree	Disagree	Don't know	Does not apply
10. I am aware of the **barriers** to moving forward with my life after stroke.				
11. I had a chance to talk to other stroke survivors **without my carer**.				
12. I had a chance to complete the '**what will I do now?'** worksheet.				
13. I know what I am going to think about doing differently from today.				
14. I received my **'Stroke journey'** handbook.				

Please write your **comments** below:

For peer review only

For peer review only - http://bmjopen.bmj.com/site/about/guidelines.xhtml

Thank you for attending **Managing health and wellbeing**.

Please tick (✓) **agree, disagree, don't know** or **does not apply** for each of the statements below.

	Agree	Disagree	Don't know	Does not apply
1. I felt like I could contribute to the session if I wanted to.				
2. I felt like I could participate in the session activities.				
3. The session included an activity on **risk factors for stroke**.				
4. I am aware of what my risks are for having another stroke.				
5. I know what I could do to help **reduce** my risk of having another stroke.				
6. I know what I could do to **stay healthy**.				
7. I understand the **pros and cons** to making change.				
8. I made an **action plan**.				
9. I am confident I can achieve my plan.				

Please turn over.

For peer review only

For peer review only - http://bmjopen.bmj.com/site/about/guidelines.xhtml

Please tick ✓ **agree**, **disagree**, **don't know** or **does not apply** for each of the statements below.

	Agree	Disagree	Don't know	Does not apply
10. There were opportunities to **ask questions** at the session.				
11. I was able to learn from other people in the group.				
12. I had a chance to complete the '**what will I do now?'** worksheet.				
13. I know what I am going to think about doing differently from today.				
14. I received my **'Managing health and wellbeing'** handbook.				

Please write your **comments** below:

For peer review only - http://bmjopen.bmj.com/site/about/guidelines.xhtml

Thank you for attending **Roadblocks**.

Please tick (✓) **agree, disagree, don't know** or **does not apply** for each of the statements below.

	Agree	Disagree	Don't know	Does not apply
1. I felt like I could contribute to the session if I wanted to.				
2. I felt like I could participate in the session activities.				
3. The group discussed the link between **thoughts, feelings, physical sensations, and behaviour**.				
4. The group discussed what problems or **'roadblocks'** people might face after a stroke.				
5. I had a chance to complete **'what are my roadblocks?'** worksheet.				
6. The session included a **problem-solving** activity.				
7. The facilitators invited me to share my problems and feelings.				
8. I am able to think about my own problems or roadblocks.				
9. I am able to think about how to solve or manage my own problems or roadblocks.				

Please turn over.

For peer review only - http://bmjopen.bmj.com/site/about/guidelines.xhtml
For peer review only

Please tick ✓ **agree**, **disagree**, **don't know** or **does not apply** for each of the statements below.

	Agree	Disagree	Don't know	Does not apply
10. There were opportunities to **ask questions** at the session.				
11. I was able to learn from other people in the group.				
12. I had a chance to complete the **'what will I do now?'** worksheet.				
13. I know what I am going to think about doing differently from today.				
14. I received my **'Roadblocks'** handbook.				

Please write your **comments** below:

For peer review only - http://bmjopen.bmj.com/site/about/guidelines.xhtml

For peer review only

Thank you for attending **'Moving forward on my journey'**.

Please tick (✓) **agree, disagree, don't know** or **does not apply** for each of the statements below.

	Agree	Disagree	Don't know	Does not apply
1. I felt like I could contribute to the session if I wanted to.				
2. I felt like I could participate in the session activities.				
3. I was able to think about how my relationships have changed since having had my stroke.				
4. The facilitators invited me to share my experiences of changing relationships.				
5. The session included a **problem-solving** activity.				
6. I had a chance to talk to other stroke survivors **without my carer**.				
7. I had a chance to think about where I am on my **journey** since my stroke.				
8. I am aware of how I can keep track of my **physical and mental health**.				
9. I know what I could do to **stay healthy**.				

Please turn over.

For peer review only

For peer review only - http://bmjopen.bmj.com/site/about/guidelines.xhtml

Please tick ✓ **agree**, **disagree**, **don't know** or **does not apply** for each of the statements below.

	Agree	Disagree	Don't know	Does not apply
10. There were opportunities to **ask questions** at the session.				
11. The group discussed ways to get **support or information**.				
12. I had a chance to complete the '**what will I do now?'** worksheet.				
13. I know what I am going to think about doing differently from today.				
14. I received my **'Moving forward on my journey'** handbook.				
15. I received my **'Directory'**.				

Please write your **comments** below:

For peer review only

For peer review only - http://bmjopen.bmj.com/site/about/guidelines.xhtml

Thank you for attending **Individual appointment 2**.

Please tick (✓) **agree, disagree, don't know** or **does not apply** for each of the statements below.

	Agree	Disagree	Don't know	Does not apply
1. I was able to reflect on where I am on my **journey** after stroke.				
2. The facilitator invited me to talk about my experience of the **'My life after stroke'** course.				
3. The facilitator invited me to complete the **'Where am I on my journey?'** worksheet.				
4. I was able to **ask questions** if I wanted to.				
5. The facilitator and I talked about my **plans** after 'My life after stroke'.				
6. The facilitator checked if I had any questions.				
7. The appointment included **information about services** that I can access.				

Please turn over.

For peer review only - http://bmjopen.bmj.com/site/about/guidelines.xhtml

Please tick ✓ **agree**, **disagree**, **don't know** or **does not apply** for each of the statements below.

	**Agree**	**Disagree**	**Don't know**	**Does not apply**
8. My carer was invited to come to my appointment.				
9. The facilitator invited my carer to **share experiences** if they wanted to.				

Please write your **comments** below:

For peer review only

For peer review only - http://bmjopen.bmj.com/site/about/guidelines.xhtml

BMJ Open

Protocol for a multidimensional process evaluation of the Improving Primary Care After Stroke (IPCAS) randomised controlled trial

Journal:	*BMJ Open*
Manuscript ID	bmjopen-2020-036879.R1
Article Type:	Protocol
Date Submitted by the Author:	14-Apr-2020
Complete List of Authors:	Aquino, Maria Raisa Jessica (Ryc); University of Cambridge, Department of Public Health and Primary Care; Newcastle University<br>Mullis, Ricky; University of Cambridge, Department of Public Health and Primary Care<br>Kreit, Elizabeth; University of Cambridge, General Practice & Primary Care Research Centre<br>Johnson, Vicki; Univ Hosp Leicester NHS Trust, Leicester Diabetees Centre<br>Grant, Julie; University of Cambridge, Department of Public Health and Primary Care<br>Lim, Lisa; University of Cambridge, Department of Public Health and Primary Care<br>Sutton, Stephen; University of Cambridge,<br>Mant, Jonathan; University of Cambridge, General Practice and Primary Care Research Unit
**Primary Subject Heading**:	General practice / Family practice
Secondary Subject Heading:	Cardiovascular medicine, Research methods, Health services research
Keywords:	Protocols & guidelines < HEALTH SERVICES ADMINISTRATION & MANAGEMENT, Clinical trials < THERAPEUTICS, PRIMARY CARE, Stroke < NEUROLOGY

SCHOLARONE™
Manuscripts

I, the Submitting Author has the right to grant and does grant on behalf of all authors of the Work (as defined in the below author licence), an exclusive licence and/or a non-exclusive licence for contributions from authors who are: i) UK Crown employees; ii) where BMJ has agreed a CC-BY licence shall apply, and/or iii) in accordance with the terms applicable for US Federal Government officers or employees acting as part of their official duties; on a worldwide, perpetual, irrevocable, royalty-free basis to BMJ Publishing Group Ltd ("BMJ") its licensees and where the relevant Journal is co-owned by BMJ to the co-owners of the Journal, to publish the Work in this journal and any other BMJ products and to exploit all rights, as set out in our licence.

The Submitting Author accepts and understands that any supply made under these terms is made by BMJ to the Submitting Author unless you are acting as an employee on behalf of your employer or a postgraduate student of an affiliated institution which is paying any applicable article publishing charge ("APC") for Open Access articles. Where the Submitting Author wishes to make the Work available on an Open Access basis (and intends to pay the relevant APC), the terms of reuse of such Open Access shall be governed by a Creative Commons licence – details of these licences and which Creative Commons licence will apply to this Work are set out in our licence referred to above.

Other than as permitted in any relevant BMJ Author's Self Archiving Policies, I confirm this Work has not been accepted for publication elsewhere, is not being considered for publication elsewhere and does not duplicate material already published. I confirm all authors consent to publication of this Work and authorise the granting of this licence.

For peer review only

Title

Protocol for a multidimensional process evaluation of the Improving Primary Care After Stroke (IPCAS) randomised controlled trial

Authors

Maria Raisa Jessica (Ryc) Aquino, PhD
Primary Care Unit, Department of Public Health and Primary Care, University of Cambridge, UK
ra532@medschl.cam.ac.uk

Mullis, Ricky, PhD
Primary Care Unit, Department of Public Health and Primary Care, University of Cambridge, UK
rm582@medschl.cam.ac.uk

Kreit, Elizabeth
Primary Care Unit, Department of Public Health and Primary Care, University of Cambridge, UK
ek445@medschl.cam.ac.uk

Johnson, Vicki
Complex Intervention Team, Leicester Diabetes Centre, University Hospitals of Leicester NHS Trust, Leicester, UK
Vicki.Johnson@uhl-tr.nhs.uk

Grant, Julie
Primary Care Unit, Department of Public Health and Primary Care, University of Cambridge, UK
julie.grant@phpc.cam.ac.uk

Lim, Lisa
Primary Care Unit, Department of Public Health and Primary Care, University of Cambridge, UK
lccl2@medschl.cam.ac.uk

Sutton, Stephen, PhD
Primary Care Unit, Department of Public Health and Primary Care, University of Cambridge, UK
srs34@medschl.cam.ac.uk

Mant, Jonathan, MD
Primary Care Unit, Department of Public Health and Primary Care, University of Cambridge, UK
jm677@medschl.cam.ac.uk

On behalf of the IPCAS investigator team: Melanie Davies, Yvonne Doherty, Kamlesh Khunti, Bundy Mackintosh, Adrian Mander, Christopher McKevitt, Martin Roland, Stephen Sutton, Marion Walker, Elizabeth Warburton.

Corresponding Author

Maria Raisa Jessica (Ryc) Aquino
Primary Care Unit, Department of Public Health and Primary Care
University of Cambridge
Worts' Causeway
Cambridge CB1 8RN

For peer review only - http://bmjopen.bmj.com/site/about/guidelines.xhtml

ra532@medschl.cam.ac.uk
+441223 762499

Word count

3328

Keywords

Process evaluation, protocol, intervention fidelity, randomised controlled trial, stroke, primary care

Abstract

Introduction

Primary care interventions are often multicomponent, with several targets (e.g. patients, healthcare professionals). Improving Primary Care After Stroke (IPCAS) is a novel primary care-based model of long-term stroke care involving a review of stroke-related needs, a self-management programme, a direct point of contact in General Practice (GP), enhanced communication between care services, and a directory of national and local community services, currently being evaluated in a cluster randomised controlled trial. Informed by Medical Research Council guidance for complex interventions and Behaviour Change Consortium fidelity framework, this protocol outlines the process evaluation of IPCAS within this RCT. The process evaluation aims to: explore how the intervention was delivered in context, and how participants engaged with the intervention.

Methods and analysis

Mixed-methods will be used. 1) Design: Intervention content will be compared to 'usual care'. 2) Training: Intervention training sessions will be audio/video-recorded where feasible 3) Delivery: Healthcare professional self-reports, audio-recordings of intervention delivery and observations of MLAS course (10% of reviews and sessions) will be coded separately; semi-structured interviews will be conducted with a purposive sample of healthcare professionals. 4) Receipt and 5) Enactment: Where available, structured stroke review records will be analysed quantitatively; semi-structured interviews will be conducted with a purposive sample of study participants. Self-reports, observations and audio/video recordings will be coded and scored using specifically developed checklists. Semi-structured interviews will be analysed thematically. Data will be analysed iteratively, independent of primary endpoint analysis.

Ethics

Favourable ethical opinion was gained from Yorkshire & The Humber - Bradford Leeds NHS Research Ethics Committee (19/12/2017; 17/YH/0441).

Discussion

This process evaluation will be used to interpret trial findings: Intervention fidelity scores can be related to the effectiveness of the intervention. The methods outlined are applicable to other settings and could inform intervention refinement to facilitate implementation of IPCAS in routine care.

Registration details

ClinicalTrials.gov Identifier: NCT03353519

For peer review only - http://bmjopen.bmj.com/site/about/guidelines.xhtml

Strengths and limitations of this study

- A strength of this process evaluation protocol includes the use of implementation frameworks, in particular a multidimensional approach to evaluation and intervention fidelity assessment.
- This study applies quantitative and qualitative methods and an iterative approach to analysis to understand how the intervention is implemented in practice.
- This process evaluation will provide insight about uptake and barriers and facilitators to implementing a new model of care in General Practice.
- A limitation of our approach is that interview findings might not be generalisable to other settings.
- We have adapted our interview sampling approach to capture as broad a range of study involvement as possible and aim to triangulate these data with other data sources, such as observations, questionnaires, and routinely collected data.

Introduction

Stroke mortality is in decline, but remains a leading cause of disability (1–3). There are rising numbers of people with stroke living in the community, and with this comes increasing demand on primary care services to address long-term needs such as cognitive, psychological and social problems (3,4). Strategies to address these long-term needs are limited, with stroke survivors and their caregivers often reporting a lack of support (3,5). Stroke prevention is a priority for the National Health Service (NHS) in England (6), as most recently outlined in the *NHS Long Term Plan*, and internationally (6–9). Primary care services have tended to focus on secondary prevention of stroke and risk factor management (10,11) rather than addressing the longer term needs (5), and no formal primary care-based model of care exists to support community-dwelling stroke survivors.

The Improving Primary Care After Stroke (IPCAS) cluster randomised controlled trial (RCT) aims to evaluate the clinical and cost effectiveness of a new model of primary care for stroke survivors living in the community compared with standard care, and has been described previously (12). General practices randomised to the intervention arm will deliver a multicomponent package of care comprising: a one-off structured review with a trained primary care professional using a novel needs checklist (13), a self-management course (My Life After Stroke; MLAS) conducted by trained facilitators over a nine-week period, which aims to improve confidence, independence and hope in survivors of stroke through problem-solving and experience and knowledge exchange with course participants; enhanced communication between healthcare professionals, and a direct point of contact in primary care accessible to patients for stroke-specific support throughout the study period. General practices randomised to the control arm will provide 'usual care'; the details of which will be documented as part of this process evaluation (see Fidelity of Design). The primary endpoint will be two sub-scales (emotion and handicap) of the Stroke Impact Scale (SIS v3.0) (14) as co-primary outcomes at 12 months after entry into the trial. The intervention and outcomes are summarised in Figure 1.

[INSERT FIGURE 1 HERE]

For peer review only - http://bmjopen.bmj.com/site/about/guidelines.xhtml

Complex interventions are widely used to address health problems and consist of multiple interacting components (15,16), often with several targets (e.g. patients, healthcare professionals) and implemented in various settings (17,18). Consequently, variations in implementation could influence intervention effects on outcomes. Process evaluations are especially important for randomised controlled trials such as IPCAS which is being conducted in 46 General Practices in the East of England and the East Midlands. These practices have contextual differences and thus the intervention may undergo adaptations as the study is conducted (18,19). It also has intervention components that are aimed at stroke survivors, as well as healthcare professionals. This warrants a multifaceted process evaluation to identify successes, failures and/or unintended consequences (15,20,21). Understanding the delivery of complex interventions is key to determining feasibility of delivering these in other settings and at scale (17).

The UK Medical Research Council (MRC) suggests that process evaluations are conducted to understand how interventions are carried out, how intervention activities produce change, and how the context in which interventions are conducted influences delivery (18,19). In line with this, the US National Institute of Health Behaviour Change Consortium's (BCC) fidelity framework and guidance specifies five dimensions of treatment fidelity: (1) Design: the degree to which the intervention is distinct (e.g. through articulated intervention components and theoretical underpinning) from usual care or other treatment conditions; (2) Training: the extent to which the training of intervention providers is adequate through monitoring training processes and activities; (3) Delivery: the extent to which intervention components and/or procedures are provided as planned; (4) Receipt: the extent to which intervention recipients understand and engage with intervention components; and (5) Enactment: the extent to which intervention recipients carry out specific/relevant behaviours learnt from the intervention in their daily lives (22,23).

This paper outlines the aims, design and methods for an embedded process evaluation within an RCT. This process evaluation is a multidimensional assessment of the IPCAS trial, specifically to explore: 1) how the intervention was delivered; 2) how participants engaged with the intervention; and 3) if/how the context influenced delivery or conduct of the intervention.

Methods and analysis

Design

A mixed-methods sequential design will be applied. The process evaluation (encompassing intervention fidelity) will be conducted in parallel to the IPCAS trial. Table 1 outlines how the five BCC fidelity dimensions will be addressed in IPCAS. Each of these dimensions – design; training; delivery; engagement (enactment and receipt) – are discussed in turn, including relevant materials and data collection processes. These are followed by the patient and public involvement and analysis plans.

For peer review only - http://bmjopen.bmj.com/site/about/guidelines.xhtml

Table 1. Assessing intervention fidelity in the *Improving Primary Care After Stroke (IPCAS)* trial

**Fidelity of design**	**Fidelity of training**	**Fidelity of delivery**	**Fidelity of engagement (receipt and enactment)**
Goal:<br>• To determine the extent to which the intervention reflects its theoretical underpinnings<br>• To determine the extent to which the intervention is distinct from 'usual care'<br>***Strategies:***<br>• Coding intervention components (i.e. IPCAS training manual, MLAS curriculum) to theoretical underpinnings<br>• Comparison of convergence between intervention and control groups (includes recording of participating surgeries' 'usual care' practices)	***Goal:***<br>• To determine the extent to which the training curriculum has been provided as planned<br>***Strategies:***<br>• Training evaluation forms (MLAS)<br>• Video-recorded observations (MLAS)<br>• Audio-recorded observations (IPCAS)<br>• Post-intervention interviews (healthcare professionals, IPCAS)	***Goal:***<br>• To determine the extent to which the intervention was provided as planned<br>***Strategies:***<br>• Audio-recorded observations (IPCAS)<br>• Structured telephone calls to healthcare professionals (IPCAS)<br>• Direct observations (MLAS)<br>• Post-intervention interviews (IPCAS)	***Goal:***<br>• To determine the extent to which stroke survivors understood and applied the skills gained from the intervention<br>***Strategies:***<br>• Self-report questionnaire (MLAS)<br>• Post-review structured telephone calls to participants (IPCAS)<br>• Post-intervention participant interviews

For peer review only

Fidelity of design

This concerns the completeness of the intervention specification, and the degree to which the intervention reflects its theoretical foundations (20,22). All IPCAS intervention components are defined in the trial protocol (12), some of which are standardised (e.g. structured review checklist for use in a one-off, face-to-face annual stroke review), and some not, in order to allow participants and practices flexibility to adapt these components to their needs (e.g. accessing direct point of contact and MLAS, enhanced communication between healthcare professionals). The intervention development process involved a multidisciplinary team (general practitioners (GP), patient representatives, primary care experts, applied health researchers, psychologists, stroke physicians, practice nurses, therapists), with pilot work informing the feasibility and acceptability of the intervention (12,13).

To ensure that the intervention reflects its theoretical underpinnings and is distinct from usual care, the intervention components (e.g. structured reviews, MLAS etc.) and parameters (e.g., frequency or dose, mode of delivery, duration) will be coded and compared by two independent coders in accordance with established methods (20,22). As such, all participating general practices will be asked to provide details of 'usual care' processes for stroke care and long-term follow-up such as the frequency and content of follow-up appointments, healthcare professionals responsible for these, investigations conducted (e.g. blood tests, blood pressure and pulse measurements) and other actions taken (e.g. referrals or further investigations). Inter-rater reliability will be assessed using Cohen's kappa (95% CI) (24). Where intervention components of similar frequency and/or mode of delivery are found in both conditions, these will be classed as *fully convergent*; where intervention components are found in both experimental conditions but are of dissimilar frequency and/or mode of delivery, these will be classed as *partially convergent*;, and where intervention components are found only in one arm of the study, these will be classed as *unique* (20). Moreover, adaptations to the intervention will be recorded and monitored by the research team throughout the intervention period through maintaining a written record of these in the fidelity of delivery calls and interviews (detailed in the following subsections).

Fidelity of training

Participating GP practices will be given a 2-hour face-to-face training session where the research team will present the intervention components and facilitate discussions on how to conduct the intervention using fictional vignettes. A sample of the training sessions (20%; up to 4 sessions) will be audio-recorded, with two raters assessing whether planned components are present using a specifically developed checklist (Supplementary file 1). Training sessions will be randomly audio-recorded (with participant consent) after the fifth training session when trainers have had an opportunity to test their curriculum and materials, and adjustments made where necessary. The percentage of present planned components will be calculated, and inter-rater agreement assessed using Cohen's kappa (95% CI) (24). MLAS facilitators will be trained via a 3-day workshop, with the research team delivering presentations on stroke and its impact, providing trainees time to practise active listening skills, explaining the philosophy and theories underpinning the self-management course, demonstrating MLAS facilitation and providing an opportunity for trainees to rehearse course delivery. MLAS training will be video-recorded and coded by an independent rater according to a priori criteria (Supplementary file 2) to assess whether planned components are present. In addition, trainees will be encouraged to complete training evaluation forms, to assess facilitator

For peer review only - http://bmjopen.bmj.com/site/about/guidelines.xhtml

knowledge of the MLAS curriculum and expected facilitator behaviours (Supplementary file 3). The percentage of present planned components will be calculated. To ensure skills are maintained, refresher training sessions will be offered as necessary. MLAS facilitators will receive mentoring from researchers as part of their training package after having had an opportunity to deliver a programme. Qualitative interviews with healthcare providers conducting structured reviews will explore their experiences of intervention training (see Fidelity of Delivery). Finally, process variables such as the number of healthcare professionals and facilitators trained, and duration of training sessions will be recorded and summarised.

Fidelity of delivery

Monitoring intervention delivery is critical to ensuring that the intervention is provided as planned; this will be conducted using quantitative and qualitative methods. The BCC guidance suggests that evaluating intervention sessions by observation (either directly or using audio/video-recording) is the gold standard for monitoring intervention delivery (22,23). To achieve this, a sample of the structured stroke reviews will be audio-recorded and coded by two independent raters according to a pre-specified checklist (Supplementary file 4), which outlines the specific intervention components which healthcare providers are expected to cover. A sample of structured reviews carried out (up to 10% or 46 30-minute reviews across all intervention sites) will be audio-recorded at each practice. With participant consent, some reviews will be recorded after the fifth review has been conducted at each practice, which allows for healthcare professionals to rehearse and become familiarised with review components. Fidelity of delivery will be assessed by calculating the percentage of the pre-specified structured review content that is present. Inter-rater reliability will be assessed using Cohen's Kappa (95% CI) (24). To enable further exploration of delivery, healthcare professionals will be contacted by members of the research team regularly during the intervention period to discuss the structured reviews and to monitor whether the intervention being delivered is as planned, with responses recorded using a pre-specified checklist (see supplementary file 4). These data will be quantified by calculating the percentage of present pre-specified structured review content. Additionally, reasons for (e.g. lack of time) and the nature of (e.g. additional support provided) deviations from the developed intervention will be explored and documented. A purposively selected sample (approximate n=15; 10 intervention and 5 control) of healthcare professionals will also be invited to participate in semi-structured interviews to explore their overall experience of delivering the intervention (intervention) or participation in the study (control) in depth. Specifically, interviews will explore healthcare professionals' reflections on their experience of conducting the structured review, the functionality of the direct point of contact and service mapping tool, and their overall experience of the intervention (e.g. what worked well vs. what did not work well). Interviews will run for approximately 30min-1hr. These interviews are planned to take place from the 6-month follow-up period.

MLAS courses will also be assessed for fidelity of delivery. Specific contents of this self-management programme are provided in the IPCAS trial protocol (12) and MLAS development paper (in prep). A sample (i.e., two full MLAS courses; approx. 20 hours of observation) of pre-selected sessions of running MLAS programmes will be directly observed independently by two observers, with disagreements resolved through consensus discussion. These observers will have been trained in course delivery and observation. A specially developed observation checklist (Supplementary file 5) will be used to code for the presence or absence of intended course contents, materials and

For peer review only - http://bmjopen.bmj.com/site/about/guidelines.xhtml

facilitator behaviours. Fidelity will be quantified by assessing the proportion of presence of pre-specified content (i.e. % coded components/planned components). These observations are planned to take place after MLAS facilitators have received mentoring; we also aim to observe all facilitators running MLAS courses at least once. MLAS facilitator-participant interactions will also be observed, using the DESMOND observation tool (Supplementary file 6), in line with similarly designed courses (25).

Finally, process variables will be collected to assess delivery, and summarised for each participating GP surgery, such as: practice list size, role of healthcare professional conducting reviews, the number of patients attending structured reviews, duration of each structured review conducted, number and content of referrals/signposting to other services, and number of MLAS courses conducted. These fidelity of delivery assessments and process measures will offer an insight into differences in intervention delivery between practices.

Fidelity of receipt and enactment

Fidelity of receipt and enactment, which concerns understanding of intervention content and application of skills gained from the intervention, respectively (22,26) will be assessed concurrently as both encompass participant engagement (26), both quantitatively and qualitatively. Similar to the process outlined for assessing fidelity of delivery, patients will be contacted by members of the research team to discuss the structured reviews they were invited to. Pre-determined questions concerning the structured review will be asked and recorded on a checklist (Supplementary file 7). This will be quantified by calculating the percentage of the present pre-specified structured review content. Concerning MLAS, a purposively selected sample of participants (i.e., 10% of participants attending MLAS, approximately 20) will be invited to complete a specially developed receipt questionnaire (Supplementary file 8) at the end of each session attended (i.e., 6 per participant in total). If necessary, participants will be offered support in completing this.

Qualitative semi-structured interviews with study participants (approximate n= 25; 15 intervention and 10 control), will be conducted to explore their understanding and experience of the new model of care (intervention arm), and their care experiences since involvement in the trial (control arm). Participants will be selected using a random purposive method, given the large pool of eligible participants (e.g. those who attended structured reviews and MLAS, and those who did not), which will allow us to gain representative insights (27). Interviews will run for approximately 30min-1hr. These interviews are expected to take place from the 6-month follow-up period. Specifically, participants will be asked about any action plans they developed with healthcare professionals during their structured review and the outcomes of these, experiences of accessing the direct point of contact for their needs, as well as participation in MLAS. Interviews with control group participants will focus on any stroke-specific appointments/contacts with the GP practice since involvement in the study and any perceived changes in their care during this period. These different areas of focus will allow for an exploration of differences within and between experimental arms.

In addition, process variables will be collected routinely, such as the number of patients invited and consented, demographic characteristics, time since stroke, number of patients attending structured reviews, number of patients completing the checklist of needs, number of participants enrolled in MLAS (defined as attending the first individual appointment and first group session), and number of participants completing MLAS (defined as attending both individual appointments and at least three of the four group sessions).

For peer review only - http://bmjopen.bmj.com/site/about/guidelines.xhtml

Patient and public involvement

Protocol and materials development involved patient and public involvement. A PPI member provided feedback on the process evaluation protocol to ensure procedures (e.g. recruitment, data collection) in place are appropriate and relevant. For example, PPI consultation resulted in changes to the format and length of the questionnaires for MLAS, ensuring that the questionnaire is aphasia friendly. Together, we decided to brief facilitators about providing stroke survivors support in completing questionnaires where needed. Our PPI member was also involved in pilot-testing the interview schedule with a member of the research team, which ensured that the interview schedule was suitable and flexible (e.g. addition of closed question prompts for people with aphasia).

Analysis plan

The process evaluation analysis will be undertaken separately to the trial outcome evaluation and will inform the interpretation of these data. Quantitative aspects of the process evaluation (e.g. observational data, checklists/questionnaires) will be assessed using descriptive statistics (i.e. proportions, frequencies and means) and percentage implementation scores on various BCC fidelity dimensions. This will enable us to examine variation in implementation between GP practices at regular intervals. It is suggested that a score of 80-100% is representative of 'high' intervention fidelity, 51-79% is representative of 'moderate' intervention fidelity, and <50% is representative of 'low' intervention fidelity (28). Qualitative aspects of the process evaluation include audio-recorded interview data and textual data (i.e. qualitative data from checklists/questionnaires). All data will be transcribed verbatim. Interview data will be synthesised using thematic analysis by members of the research team using established methods (29). This will also involve an iterative comparison of participant and healthcare professional interview data to understand similarities and differences between intervention providers and recipients. Textual data from checklists and questionnaires will be analysed using content analysis. NVivo 12 will be used to support qualitative data management and analysis.

The quantitative and qualitative analyses will be conducted and integrated at different phases of the evaluation. For example, comparing findings from different data sources such as participants' self-reported receipt and enactment questionnaires and healthcare professionals' self-reported delivery questionnaires with the audio-recorded observations of the structured stroke reviews.

Ethics

Favourable ethical opinion for the research was gained on 19th December 2017 from Yorkshire & The Humber - Bradford Leeds NHS Research Ethics Committee. Approval to start was given by the Health Research Authority (HRA) on 21st December 2017, prior to the recruitment of participants commencing at any NHS site.

Discussion

This multifaceted process evaluation aims to complement the effectiveness testing of a theory-driven complex intervention. It will generate knowledge concerning how and why this novel model of primary care-based stroke care could have an impact, thereby informing ways of scaling up the intervention and implementing the model in other settings as part of usual care. Specifically, the present intervention contains adaptable components as it is implemented pragmatically; therefore, process evaluation findings can offer an insight into how this model of care contributes to the evidence base by illustrating the extent to which a high level of fidelity is useful or necessary.

For peer review only

It has been suggested that there are four possible scenarios as to the contribution of process evaluation findings to investigating how interventions are effective or not (20): 1) successful intervention/high fidelity, where trial outcomes demonstrate improvements in the intervention group and treatment fidelity is deemed 'high', which increases confidence in associating trial outcomes with the intervention delivered; 2) successful intervention/low fidelity, where trial outcomes demonstrate improvements in the intervention group and treatment fidelity is deemed 'low', which suggests that there may have been confounding factors, or mechanisms of action through which the intervention operated which were not identified and assessed; 3) unsuccessful intervention/high fidelity: where trial outcomes demonstrate no improvements in the intervention group and treatment fidelity is deemed 'high', which supports the conclusion that the intervention was not effective, or could have been influenced by confounding factors; and 4) unsuccessful intervention/low fidelity: where trial outcomes demonstrate no improvements in the intervention group and treatment fidelity is deemed 'low', which suggests that the intervention was not tested as it was not carried out as planned, or that poor intervention fidelity could have negatively impacted intervention outcomes.

It is recommended that process evaluation protocols are reported and published to encourage transparency (17–20). The protocol described here builds on emerging process evaluation methodology literature which can inform the development and implementation of complex interventions.

Trial status

At the time of submission, the IPCAS trial recruitment is ongoing, with the target sample size of 920 surpassed in March 2019. The IPCAS process evaluation started in March 2018 alongside GP practice recruitment, with the fidelity of design and training data collection and analysis commencing July 2018. Pilot observations and interviews were conducted in February-March 2019, with ongoing iterative analysis.

Author contributions

JM, RM, and SS were responsible for obtaining the funding for this study. The design of the process evaluation was led by RA, with contributions from JM, RM, EK, VJ, LL. JM, RM, EK, VJ, and LL contributed to developing intervention fidelity measures. EK, VJ, LL and JG contributed to piloting intervention fidelity materials, participant recruitment, data collection and analysis. RA wrote the first draft of the manuscript, and all authors contributed to and approved the final version.

Funding

This study is funded by the National Institute for Health Research's Programme Grant for Applied Research titled 'Developing primary care services for stroke survivors' reference PTC-RP-PG-0213-20001. The views expressed are those of the authors and not necessarily those of the NIHR or the Department of Health and Social Care. Jonathan Mant is an NIHR Senior Investigator.

Competing interests

None declared.

For peer review only - http://bmjopen.bmj.com/site/about/guidelines.xhtml

References

1. Feigin VL, Norrving B, Mensah GA. Global burden of stroke. Circ Res. 2017;120(3):439–48.

2. Lee S, Shafe AC, Cowie MR. UK stroke incidence, mortality and cardiovascular risk management 1999–2008: time-trend analysis from the General Practice Research Database. BMJ Open. 2011;1(2):e000269.

3. McKevitt C, Fudge N, Redfern J, Sheldenkar A, Crichton S, Rudd AR, et al. Self-reported long-term needs after stroke. Stroke. 2011;42(5):1398–403.

4. Murray J, Young J, Forster A. Review of longer-term problems after a disabling stroke. Rev Clin Gerontol. 2007;17(4):277–92.

5. Pindus DM, Mullis R, Lim L, Wellwood I, Rundell AV, Aziz NAA, et al. Stroke survivors' and informal caregivers' experiences of primary care and community healthcare services–A systematic review and meta-ethnography. PloS One. 2018;13(2):e0192533.

6. NHS England. The NHS Long Term Plan. 2019.

7. US Department of Health and Human Services. Healthy people 2020 [Internet]. Office of Disease Prevention and Health Promotion; 2010 [cited 2019 Dec 17]. Available from: https://www.healthypeople.gov/sites/default/files/HP2020_brochure_with_LHI_508_FNL.pdf

8. The Ministry of Health and Care Services (Norway). Care plan 2020: The Norwegian Government's plan for the care services field for 2015-2020 [Internet]. The Ministry of Health and Care Services (Norway); 2015 [cited 2019 Dec 17]. Available from: https://www.regjeringen.no/contentassets/af2a24858c8340edaf78a77e2fbe9cb7/careplan2020_eng.pdf

9. Centers for Disease Control and Prevention. A study of primary stroke center policy: Recommendations for policy implementation [Internet]. US Department of Health and Human Services; 2012 [cited 2019 Dec 17]. Available from: https://www.cdc.gov/dhdsp/docs/Primary_Stroke_Center_Policies_Recommendations.pdf

10. Norrving B, Davis SM, Feigin VL, Mensah GA, Sacco RL, Varghese C. Stroke prevention worldwide-what could make it work. Neuroepidemiology. 2015;45(3):215–20.

11. Joubert J, Reid C, Joubert L, Barton D, Ruth D, Jackson D, et al. Risk factor management and depression post-stroke: the value of an integrated model of care. J Clin Neurosci. 2006;13(1):84–90.

12. Mullis R, Aquino MRJ, Dawson S, Johnson V, Jowett S, Kreit E, et al. The IPCAS Trial (Improving Primary Care After Stroke): protocol of a randomised controlled trial to evaluate a novel model of care for stroke survivors living in the community. BMJ Open. 2019;9:e030285.

13. Turner GM, Mullis R, Lim L, Kreit L, Mant J. Using a checklist to facilitate management of long-term care needs after stroke: insights from focus groups and a feasibility study. BMC Fam Pract. 2019;20(1):2.

14. Jenkinson C, Fitzpatrick R, Crocker H, Peters M. The Stroke Impact Scale: validation in a UK setting and development of a SIS short form and SIS index. Stroke. 2013;44(9):2532–5.

For peer review only

15. Craig P, Dieppe P, Macintyre S, Michie S, Nazareth I, Petticrew M, et al. Developing and evaluating complex interventions: the new Medical Research Council guidance. BMJ. 2008;337:a1655–a1655.

16. Campbell M, Fitzpatrick R, Haines A, Kinmonth AL, Sandercock P, Spiegelhalter D, et al. Framework for design and evaluation of complex interventions to improve health. Bmj. 2000;321(7262):694–6.

17. Liu H, Lindley R, Alim M, Felix C, Gandhi DB, Verma SJ, et al. Protocol for process evaluation of a randomised controlled trial of family-led rehabilitation post stroke (ATTEND) in India. BMJ Open. 2016;6(9):e012027.

18. Moore GF, Audrey S, Barker M, Bond L, Bonell C, Hardeman W, et al. Process evaluation of complex interventions: Medical Research Council guidance. bmj. 2015;350:h1258.

19. Campbell-Scherer D, Saitz R. Improving reporting and utility of evaluations of complex interventions. BMJ Evid-Based Med. 2016;21:1–3.

20. Lorencatto F, Gould NJ, McIntyre SA, During C, Bird J, Walwyn R, et al. A multidimensional approach to assessing intervention fidelity in a process evaluation of audit and feedback interventions to reduce unnecessary blood transfusions: a study protocol. Implement Sci. 2016;11(1):163.

21. Oakley A, Strange V, Bonell C, Allen E, Stephenson J. Process evaluation in randomised controlled trials of complex interventions. Bmj. 2006;332(7538):413–6.

22. Borrelli B, Sepinwall D, Ernst D, Bellg AJ, Czajkowski S, Breger R, et al. A new tool to assess treatment fidelity and evaluation of treatment fidelity across 10 years of health behavior research. J Consult Clin Psychol. 2005;73(5):852.

23. Bellg AJ, Borrelli B, Resnick B, Hecht J, Minicucci DS, Ory M, et al. Enhancing Treatment Fidelity in Health Behavior Change Studies: Best Practices and Recommendations From the NIH Behavior Change Consortium. Health Psychol. 2004;23(5):443–51.

24. Walton H, Spector A, Williamson M, Tombor I, Michie S. Developing quality fidelity and engagement measures for complex health interventions. Br J Health Psychol. 2019;

25. Skinner T, Carey M, Cradock S, Dallosso H, Daly H, Davies MJ, et al. 'Educator talk'and patient change: some insights from the DESMOND (Diabetes Education and Self Management for Ongoing and Newly Diagnosed) randomized controlled trial. Diabet Med. 2008;25(9):1117–20.

26. Walton H, Spector A, Tombor I, Michie S. Measures of fidelity of delivery of, and engagement with, complex, face-to-face health behaviour change interventions: A systematic review of measure quality. Br J Health Psychol. 2017;22(4):872–903.

27. Patton M. Qualitative research and evaluation methods. 4th ed. Saint Paul, MN: SAGE; 2015.

28. Noell GH, Gresham FM, Gansle KA. Does treatment integrity matter? A preliminary investigation of instructional implementation and mathematics performance. J Behav Educ. 2002;11(1):51–67.

29. Braun V, Clarke V. Thematic analysis. In: Cooper H, Camic PM, Long DL, Panter A, Rindskopf D, Sher KJ, editors. APA handbook of resaerch methods in psychology, Vol 2 Resaerch designs:

For peer review only

For peer review only - http://bmjopen.bmj.com/site/about/guidelines.xhtml

Quantitative, qualitative, neuropsychological, amd biological. American Psychological Association; 2012.

For peer review only

Figure 1. Intervention and outcomes

Contextual factors within intervention environment: 'Usual care' at each practice, socioeconomic factors, stroke-related factors (e.g., time since stroke, comorbidities)

Improving Primary Care After Stroke (IPCAS): a primary care-based model of care to support community-dwelling stroke survivors

Participant-focused components

15-item checklist of common post-stroke needs sent to participant by post. Followed by:

Structured review appointment: Up to 30-min with a trained member of the practice team (e.g. nurse, GP) to discuss up to 3 needs prioritised by the patient from the checklist. The outcomes are an action plan agreed with the patient on how to address the key needs, information on the Direct Point of Contact (DPOC) service, and on the My Life After Stroke (MLAS) course.

My Life After Stroke (MLAS), an optional 6-week, group-based self-management course delivered by two trained facilitators in a local community venue. The first and final appointments are individual appointments lasting up to 45 minutes, in between which are four-weekly group sessions focused on learning about the consequences of a stroke, and managing one's health and wellbeing using goal setting and problem-solving strategies. In the final group session, participants will also be given an abbreviated print version of the service mapping tool (see below).

Healthcare professional-focused components:

Training on new model of primary care: One-off, face-to-face session with trial team lasting up to 2 hours.

DPOC: Trained practice staff will provide stroke-specific advice, information and/or support to study participants by telephone.

Optimised communication between healthcare professionals: A meeting will be arranged between primary care and specialist staff (hospital and community) to facilitate interprofessional communication. All practice staff involved with providing care to stroke survivors will be encouraged to attend any meetings/training sessions organised by specialists, and provided specialists' contact details. Video recordings of local specialist providers describing their service, commonly referred patients to the service, and means of contact/referral will be provided to practice staff.

Service mapping tool: This is a bespoke directory of national and local services provided to practices in Excel and print formats. These can be used at the structured review, or when providing support via the DPOC service.

Primary outcomes

Baseline, 6, and 12 mos.:

Emotion and handicap sub-scales of Stroke Impact Scale v3.0

Secondary outcomes

6 and 12 mos.:

EQ-5D-5L, ICEpop CAPability measure for Adults

12 mos. only:

Southampton Stroke Self-management Questionnaire, Health Literacy Questionnaire

Long-term outcomes

Cost-utility as determined in within-trial economic evaluation

Supplementary file 1

IPCAS trial provider process evaluation/fidelity checklist – training phase							
**Site ID:**		**Duration of training:**			**Training date (Coding date):**		
**Activity**			**Tick as appropriate**			**If *present to some extent*, provide reason(s) for this rating**	**Other comments**
			**Present**	**Absent**	**Partially done**		
**Structured review**	1. 15-item checklist of needs	a) Discussion of purpose and structure of checklist					
		b) Instructions on how to use checklist					
	2. Physical check	a) Discussion of carrying out optional physical checks at review (in line with QOF)					
	3. Development of action plan(s)	a) Discussion of action plan					
		b) Instructions given: how to log and review agreed actions (using template)					
	4. MLAS	a) Discussion of purpose and structure of MLAS					
		b) Provided instructions for how patients can access MLAS					
	5. Direct point of contact to stroke survivor and carer(s)	a) Given instructions for offering 'direct point of contact' service					
		b) Discussion of how to provide instructions to patients about contacting practice at the end of review					
**Use of direct point of contact**	1. Signposting to services	a) Discussed service mapping: what it is					
		b) Discussed how to use service mapping					

For peer review only - http://bmjopen.bmj.com/site/about/guidelines.xhtml

**IPCAS trial provider process evaluation/fidelity checklist – training phase**							
**Site ID:**		**Duration of training:**			**Training date (Coding date):**		
**Activity**			**Tick as appropriate**			**If *present to some extent*, provide reason(s) for this rating**	**Other comments**
			**Present**	**Absent**	**Partially done**		
	2. Provide support and advice	a) Gave information on structure and scope of direct point of contact service: giving advice for stroke-specific issues					
		b) Gave information on structure and scope of direct point of contact service: arranging follow-up appointment(s)					
		c) Gave information on structure and scope of direct point of contact service: brief telephone support/case management					
**Enhanced communication pathways**	1. Discussion on communication pathways	a) Discussed broad structure of enhanced communication pathways					
		b) Initial discussion about dates for face-to-face meeting					
**END OF CHECKLIST**							

For peer review only

For peer review only - http://bmjopen.bmj.com/site/about/guidelines.xhtml

Supplementary file 2

IPCAS trial process evaluation/fidelity checklist
MLAS facilitator training

**Date:**					
**Start time:**					
**End time:**					
**Training day 1**					
	**Please tick as appropriate**			**If *absent, attempted* or *not applicable*, please provide reasons**	**Other comments**
	**Present**	**Absent**	**Attempted**		
**Session 1: Welcome and introductions**					
**Duration of session:**					
**Materials**					
Timetable available	☐	☐	☐		
Headed flipchart paper – working together	☐	☐	☐		
Pre-training 'evaluation' form given (1 per participant)	☐	☐	☐		
**Content**					
Welcome to MLAS	☐	☐	☐		
Introduction of trainers	☐	☐	☐		

For peer review only - http://bmjopen.bmj.com/site/about/guidelines.xhtml

**Date:**					
**Start time:**					
**End time:**					
**Training day 1**					
	**Please tick as appropriate**			**If *absent, attempted* or *not applicable*, please provide reasons**	**Other comments**
	**Present**	**Absent**	**Attempted**		
Housekeeping details provided	☐	☐	☐		
Aim of training discussed	☐	☐	☐		
Training overview discussed	☐	☐	☐		
Outline of Day 1 discussed	☐	☐	☐		
Working together discussed	☐	☐	☐		
Provided time for participants to complete pre-training 'evaluation' form	☐	☐	☐		

For peer review only

For peer review only - http://bmjopen.bmj.com/site/about/guidelines.xhtml

**Session 2: Your story**					
**Duration of session:**					
**Materials**					
Headed flipchart paper (1: Name, role, place of work, 2: Experience, 3: Expectation of training, 4: Your important question)	☐	☐	☐		
**Content**					
Asked: Name, role, place of work	☐	☐	☐		
Asked: Experience (facilitating/observing groups, working with stroke)	☐	☐	☐		
Asked: Expectation(s)	☐	☐	☐		
Asked: Any question in relation to MLAS	☐	☐	☐		
Provided opportunity for each trainee to answer the above questions and record on relevant flipchart	☐	☐	☐		
**Session 3: What is stroke?**					
**Duration of session:**					
**Materials**					
Body outline used	☐	☐	☐		
Images shown: clot, bleed, brain areas	☐	☐	☐		
Images shown: risk factors, neuroplasticity	☐	☐	☐		

For peer review only

For peer review only - http://bmjopen.bmj.com/site/about/guidelines.xhtml

Videos shown: Act FAST, neuroplasticity	☐	☐	☐		
Images shown: health and well-being	☐	☐	☐		
Sticky notes used	☐	☐	☐		
**Content**					
Discussed 'what is stroke?'	☐	☐	☐		
Discussed signs and symptoms – ACTFAST	☐	☐	☐		
Discussed impacts of a stroke (including physical, emotional, cognitive, communication, relationships)	☐	☐	☐		
Discussed what neuroplasticity is	☐	☐	☐		
Discussed stroke risk factors	☐	☐	☐		
Discussed managing risk factors	☐	☐	☐		
Participants given an opportunity to contribute to discussion and activities	☐	☐	☐		
**Session 4: Cognitive effects of stroke**					
**Duration of session:**					
**Materials**					
PowerPoint slides used: cognitive effects of stroke	☐	☐	☐		
Video shown: Hidden effects of stroke	☐	☐	☐		

For peer review only - http://bmjopen.bmj.com/site/about/guidelines.xhtml

Booklet given: Complete guide to Cognitive problems (1 copy/facilitator)	☐	☐	☐		
**Content**					
Session introduced	☐	☐	☐		
Discussed memory problems after a stroke	☐	☐	☐		
Explored attention difficulties	☐	☐	☐		
Explored changes to perception	☐	☐	☐		
Explored effects on decision-making	☐	☐	☐		
Explored social cognition problems	☐	☐	☐		
Discussed how we can support stroke survivors experiencing these effects/top tips	☐	☐	☐		
Discussed the impact of impaired cognition on the stroke survivor and/or carer/family	☐	☐	☐		
Participants given an opportunity to contribute to discussion and activities	☐	☐	☐		
**Session 5: Emotional changes after stroke**					
**Duration of session:**					
**Materials**					
Video shown: The emotional impact of stroke	☐	☐	☐		
PowerPoint slides: Emotional problems following stroke					

For peer review only

Booklet given: Complete guide to Emotional changes after stroke (1 copy/facilitator)	☐	☐	☐		
**Content**					
Session introduced	☐	☐	☐		
Discussed the emotions stroke survivors highlighted in video observed	☐	☐	☐		
Discussed stages of loss	☐	☐	☐		
Discussed problems stroke survivors might face with emotions	☐	☐	☐		
Discussed difficulty controlling emotions	☐	☐	☐		
Discussed how these emotions can influence people's thoughts and behaviours	☐	☐	☐		
Discussed how facilitators can support stroke survivors experiencing these effects/top tips	☐	☐	☐		
Participants given an opportunity to contribute to discussion and activities	☐	☐	☐		
**Session 6: Relationships**					
**Duration of session:**					
**Materials**					
PowerPoint slides used: Relationships	☐	☐	☐		
**Content**					

For peer review only

For peer review only - http://bmjopen.bmj.com/site/about/guidelines.xhtml

Session introduced	☐	☐	☐		
Discussed the effect of a stroke on relationships (partners, friends, family members)	☐	☐	☐		
Discussed problems and solutions concerning physical intimacy after a stroke	☐	☐	☐		
Participants given an opportunity to contribute to discussion and activities	☐	☐	☐		
**Session 7: Communication challenges for survivors of stroke**					
**Duration of session:**					
**Materials**					
PowerPoint slides used: Communication	☐	☐	☐		
Video used: Living with aphasia; aphasia etiquette	☐	☐	☐		
Booklet given: Complete guide to Communication problems after stroke (1 copy/facilitator)	☐	☐	☐		
**Content**					
Session introduced	☐	☐	☐		
Group activity: Identifying tasks the brain needs to complete in order to be able to answer a question	☐	☐	☐		

For peer review only - http://bmjopen.bmj.com/site/about/guidelines.xhtml

Discussed communication problems after a stroke	☐	☐	☐		
Discussed how we can support stroke survivors experiencing communication problems (top tips, resources/aids)	☐	☐	☐		
Participants given an opportunity to contribute to discussion and activities	☐	☐	☐		
**Session 8: Summary and evaluation**					
**Duration of session:**					
**Materials**					
Training evaluation form given	☐	☐	☐		
Questions relating to MLAS flipchart	☐	☐	☐		
**Content**					
Summary of Day 1 Training	☐	☐	☐		
Provided time for participants to complete training evaluation form	☐	☐	☐		
Provided details of Day 2 Training	☐	☐	☐		
Participants given an opportunity to check if their questions addressed, and to ask any further questions	☐	☐	☐		
**END OF DAY 1 TRAINING**					

For peer review only - http://bmjopen.bmj.com/site/about/guidelines.xhtml

IPCAS trial process evaluation/fidelity checklist
MLAS facilitator training

**Date:**					
**Start time:**					
**End time:**					
**Training day 2**					
	**Please tick as appropriate**			**If *absent, attempted* or *not applicable*, please provide reasons**	**Other comments**
	**Present**	**Absent**	**Attempted**		
**Session 1: Welcome and introductions**					
**Duration of session:**					
**Materials**					
Timetable available	☐	☐	☐		
Headed flipchart paper from day 1 ('Your story' - name, experience, expectation, important question and working together as a group)	☐	☐	☐		
**Content**					
Welcome	☐	☐	☐		
Introduction of trainers	☐	☐	☐		

For peer review only - http://bmjopen.bmj.com/site/about/guidelines.xhtml

**Date:**					
**Start time:**					
**End time:**					
**Training day 2**					
	**Please tick as appropriate**			**If *absent, attempted* or *not applicable*, please provide reasons**	**Other comments**
	**Present**	**Absent**	**Attempted**		
Housekeeping details provided	☐	☐	☐		
Overall aim/format of training discussed	☐	☐	☐		
Outline of Day 2 discussed	☐	☐	☐		
Invited any new participants to introduce themselves to group using 'Your story' flipchart from Day 1	☐	☐	☐		
**Session 2: Background to MLAS**					
**Duration of session:**					
**Materials**					
Flipchart paper, pens	☐	☐	☐		
A4 Quadrant image	☐	☐	☐		
**Content**					
Discussed what MLAS is	☐	☐	☐		

For peer review only - http://bmjopen.bmj.com/site/about/guidelines.xhtml

**Date:**					
**Start time:**					
**End time:**					
**Training day 2**					
	**Please tick as appropriate**			**If *absent, attempted* or *not applicable*, please provide reasons**	**Other comments**
	**Present**	**Absent**	**Attempted**		
Discussed what a structured education programme is	☐	☐	☐		
Discussed rationale for MLAS	☐	☐	☐		
Discussed aims and format of MLAS course	☐	☐	☐		
Participants given an opportunity to be involved in discussion and ask questions	☐	☐	☐		
**Session 3: Philosophy**					
**Duration of session:**					
**Materials**					
How are you doing? Worksheet (1 per participant)	☐	☐	☐		
PowerPoint slides used: Philosophy	☐	☐	☐		
**Content**					

For peer review only - http://bmjopen.bmj.com/site/about/guidelines.xhtml

**Date:**					
**Start time:**					
**End time:**					
**Training day 2**					
	**Please tick as appropriate**			**If *absent, attempted* or *not applicable*, please provide reasons**	**Other comments**
	**Present**	**Absent**	**Attempted**		
Discussed what philosophy is and how it affects our behaviours	☐	☐	☐		
Explored the medical model and empowerment approach	☐	☐	☐		
Explored the philosophy and approach of the MLAS programme	☐	☐	☐		
Facilitated individual activity ('How are you doing' quiz) and gave an opportunity for each trainee to complete their own worksheet	☐	☐	☐		
Discussed the need to have importance, confidence and readiness in order to change behaviour (and not just knowledge)	☐	☐	☐		
Presented overall aim of MLAS and its core philosophy statements	☐	☐	☐		
Discussed MLAS facilitators' role					

For peer review only - http://bmjopen.bmj.com/site/about/guidelines.xhtml

**Date:**					
**Start time:**					
**End time:**					
**Training day 2**					
	**Please tick as appropriate**			**If *absent, attempted* or *not applicable*, please provide reasons**	**Other comments**
	**Present**	**Absent**	**Attempted**		
Group activity: Challenges experienced/perceived by facilitators when using the philosophy/approach underpinning MLAS	☐	☐	☐		
Participants given an opportunity to be involved in discussion (e.g. by giving feedback) and ask questions	☐	☐	☐		
**Session 4: Facilitator behaviours**					
**Duration of session:**					
**Materials**					
Rice pudding exercise	☐	☐	☐		
MLAS facilitator behaviours checklist given (1 per participant)	☐	☐	☐		
**Content**					

For peer review only - http://bmjopen.bmj.com/site/about/guidelines.xhtml

**Date:**					
**Start time:**					
**End time:**					
**Training day 2**					
	**Please tick as appropriate**			**If *absent, attempted* or *not applicable*, please provide reasons**	**Other comments**
	**Present**	**Absent**	**Attempted**		
Session introduced	☐	☐	☐		
Rice pudding activity (demonstrating different facilitator behaviours – 'good' and 'bad')	☐	☐	☐		
Discussed positive and negative facilitator behaviours that were observed	☐	☐	☐		
Discussed some of the behaviours on the behaviours checklist, and provided examples of what would be observed if such behaviours were being done (e.g. body language, words used etc.)	☐	☐	☐		
Encouraged participants to engage in discussion about any stroke-specific behaviours needed	☐	☐	☐		
Participants given an opportunity to be involved in discussion (e.g. by giving feedback) and ask questions	☐	☐	☐		

For peer review only

For peer review only - http://bmjopen.bmj.com/site/about/guidelines.xhtml

**Date:**					
**Start time:**					
**End time:**					
**Training day 2**					
	**Please tick as appropriate**			**If *absent, attempted* or *not applicable*, please provide reasons**	**Other comments**
	**Present**	**Absent**	**Attempted**		
**Session 5: Facilitation skills**					
**Duration of session:**					
**Materials**					
PowerPoint slides used: Facilitation skills	☐	☐	☐		
Flipchart paper and pens					
Open-ended Questions, Affirmations, Reflective listening, Summaries (OARS) sheets given (1 per participant)	☐	☐	☐		
**Content**					
Session introduced	☐	☐	☐		
Facilitated group activity on facilitator role and responsibilities	☐	☐	☐		
OARS introduced to participants	☐	☐	☐		

For peer review only

**Date:**					
**Start time:**					
**End time:**					
**Training day 2**					
	**Please tick as appropriate**			**If *absent, attempted* or *not applicable*, please provide reasons**	**Other comments**
	**Present**	**Absent**	**Attempted**		
Group activity: Challenging scenarios and resolutions	☐	☐	☐		
Encouraged participants to share personal learning	☐	☐	☐		
Participants given an opportunity to be involved in discussion (e.g. by giving feedback) and ask questions	☐	☐	☐		
**Session 6: Theories – Narrative Approach; Self-efficacy**					
**Duration of session:**					
**Materials**					
PowerPoint slides used: Theories	☐	☐	☐		
Sticky notes, flipchart paper, pens	☐	☐	☐		
MLAS Roadmap and accessories	☐	☐	☐		
**Content**					

For peer review only - http://bmjopen.bmj.com/site/about/guidelines.xhtml

**Date:**					
**Start time:**					
**End time:**					
**Training day 2**					
	**Please tick as appropriate**			**If *absent, attempted* or *not applicable*, please provide reasons**	**Other comments**
	**Present**	**Absent**	**Attempted**		
Session introduced	☐	☐	☐		
Introduced the Narrative Approach	☐	☐	☐		
Demonstrated how the narrative approach is used in MLAS (journey analogy)	☐	☐	☐		
Introduced Self-efficacy (Mastery, vicarious learning, verbal persuasion and emotions management)	☐	☐	☐		
Group activity: Real-world experiences of self-efficacy	☐	☐	☐		
Examples provided where self-efficacy is used during MLAS	☐	☐	☐		
Introduced cognitive behavioural approach and gave an example of how it is used in MLAS	☐	☐	☐		

For peer review only

For peer review only - http://bmjopen.bmj.com/site/about/guidelines.xhtml

**Date:**					
**Start time:**					
**End time:**					
**Training day 2**					
	**Please tick as appropriate**			**If *absent, attempted* or *not applicable*, please provide reasons**	**Other comments**
	**Present**	**Absent**	**Attempted**		
Participants given an opportunity to be involved in discussion (e.g. by giving feedback) and ask questions	☐	☐	☐		
**Session 7: Introduction to Quality Assurance**					
**Duration of session:**					
**Materials**					
Flipchart paper and pens	☐	☐	☐		
**Content**					
Session introduced	☐	☐	☐		
Discussed what quality assurance is and its important	☐	☐	☐		
Facilitated small group activity (recipe and ingredients for making an Apple Pie)	☐	☐	☐		

For peer review only - http://bmjopen.bmj.com/site/about/guidelines.xhtml

**Date:**					
**Start time:**					
**End time:**					
**Training day 2**					
	**Please tick as appropriate**			**If *absent, attempted* or *not applicable*, please provide reasons**	**Other comments**
	**Present**	**Absent**	**Attempted**		
Encouraged each group to feedback on group activity.	☐	☐	☐		
Compared similarities and differences of Apple Pie recipes and linked to quality assurance of MLAS (ingredients = curriculum, method = delivery)	☐	☐	☐		
Summarised group discussion on quality assurance processes	☐	☐	☐		
Participants given an opportunity to be involved in discussion (e.g. by giving feedback) and ask questions	☐	☐	☐		
**Session 8: A journey through the MLAS curriculum**					
**Duration of session:**					
**Materials**					

For peer review only - http://bmjopen.bmj.com/site/about/guidelines.xhtml

**Date:**					
**Start time:**					
**End time:**					
**Training day 2**					
	**Please tick as appropriate**			**If *absent, attempted* or *not applicable*, please provide reasons**	**Other comments**
	**Present**	**Absent**	**Attempted**		
MLAS curriculum given to each facilitator	☐	☐	☐		
Participant (stroke survivor) handbooks given to each facilitator (4 handbooks in total)	☐	☐	☐		
A journey through the curriculum question sheet given to each facilitator	☐	☐	☐		
**Content**					
Provided an overview of materials given	☐	☐	☐		
Explained the purpose of 'a journey through the curriculum question sheet' and instructions for completion	☐	☐	☐		
**Session 9: Close of the day reflections and evaluation**					
**Duration of session:**					
**Materials**					
Training evaluation form (day 2) given	☐	☐	☐		

For peer review only - http://bmjopen.bmj.com/site/about/guidelines.xhtml

**Date:**					
**Start time:**					
**End time:**					
**Training day 2**					
	**Please tick as appropriate**			**If *absent, attempted* or *not applicable*, please provide reasons**	**Other comments**
	**Present**	**Absent**	**Attempted**		
Questions relating to MLAS flipchart	☐	☐	☐		
**Content**					
Summary of Day 2 training by encouraging participants to share learning about what was covered	☐	☐	☐		
Provided time for participants to complete training evaluation form	☐	☐	☐		
Provided details of Day 3 Training	☐	☐	☐		
Participants given an opportunity to check if their question (from flipchart) was addressed, and to ask any further questions	☐	☐	☐		
**END OF DAY 2 TRAINING**					

For peer review only - http://bmjopen.bmj.com/site/about/guidelines.xhtml

IPCAS trial process evaluation/fidelity checklist

MLAS facilitator training

**Date:**					
**Start time:**					
**End time:**					
**Training day 3**					
	**Please tick as appropriate**			**If *absent* or *attempted* please provide reasons**	**Other comments**
	**Present**	**Absent**	**Attempted**		
**Session 1: Welcome and introductions**					
**Duration of session:**					
**Materials**					
Timetable available	☐	☐	☐		
Headed flipchart paper from days 1+2 ('Your story' - name, experience, expectation, important question and working together as a group)	☐	☐	☐		
**Content**					
Welcome	☐	☐	☐		

For peer review only - http://bmjopen.bmj.com/site/about/guidelines.xhtml

**Date:**					
**Start time:**					
**End time:**					
**Training day 3**					
	**Please tick as appropriate**			**If *absent* or *attempted* please provide reasons**	**Other comments**
	**Present**	**Absent**	**Attempted**		
Re-introduction of trainers	☐	☐	☐		
Housekeeping details provided	☐	☐	☐		
Aim of training discussed	☐	☐	☐		
Outline of Day 3 discussed	☐	☐	☐		
Invited any new participants to introduce themselves to group using 'Your story' flipchart from Day 1+2	☐	☐	☐		
**Session 2: Reflection from 'A journey through the curriculum' activity**					
**Duration of session:**					
**Materials**					
A journey through the curriculum activity sheet	☐	☐	☐		
A journey through the curriculum activity answer sheet given	☐	☐	☐		
MLAS curriculum	☐	☐	☐		
**Content**					

For peer review only - http://bmjopen.bmj.com/site/about/guidelines.xhtml

**Date:**					
**Start time:**					
**End time:**					
**Training day 3**					
	**Please tick as appropriate**			**If *absent* or *attempted* please provide reasons**	**Other comments**
	**Present**	**Absent**	**Attempted**		
Invited participants to share experiences of completing 'a journey through the curriculum' activity	☐	☐	☐		
Discussed format of the programme (1:1 and group-based sessions; interchangeable sessions)	☐	☐	☐		
Discussed format of the curriculum (e.g. key for how to use the curriculum, duration for each section, section overviews)	☐	☐	☐		
Discussed involvement of carers in MLAS and format of this	☐	☐	☐		
Participants given an opportunity to be involved in discussion and ask questions	☐	☐	☐		
**Session 3: A walk through the MLAS curriculum**					
**Duration of session:**					
**Materials**					

For peer review only

For peer review only - http://bmjopen.bmj.com/site/about/guidelines.xhtml

**Date:**					
**Start time:**					
**End time:**					
**Training day 3**					
	**Please tick as appropriate**			**If *absent* or *attempted* please provide reasons**	**Other comments**
	**Present**	**Absent**	**Attempted**		
MLAS curriculum	☐	☐	☐		
Participant (stroke survivor) handbooks	☐	☐	☐		
Resources/accessories for programme available	☐	☐	☐		
**Content**					
Discussed the overall aims of the study and the intervention	☐	☐	☐		
Presented each of the MLAS sessions with the resources	☐	☐	☐		
Demonstrated how to use the resources	☐	☐	☐		
Demonstrated links between the sessions and materials to the theories and philosophy applied	☐	☐	☐		
Discussed challenges to delivery and strategies for addressing these	☐	☐	☐		
Discussed tailoring of sessions	☐	☐	☐		

For peer review only - http://bmjopen.bmj.com/site/about/guidelines.xhtml

**Date:**					
**Start time:**					
**End time:**					
**Training day 3**					
	**Please tick as appropriate**			**If *absent* or *attempted* please provide reasons**	**Other comments**
	**Present**	**Absent**	**Attempted**		
Discussed some examples of what previous facilitators have observed/dealt with during previous courses	☐	☐	☐		
Participants given an opportunity to be involved in discussion and ask questions	☐	☐	☐		
**Session 4: Preparing for having a go**					
**Duration of session:**					
**Materials**					
Facilitator curricula	☐	☐	☐		
'Have a go' cards (1 per facilitator)	☐	☐	☐		
Roadmap and accessories available	☐	☐	☐		
A4 images: blood clot, bleed available	☐	☐	☐		
A6 images: risk factors, risks I can/cannot change available	☐	☐	☐		

For peer review only - http://bmjopen.bmj.com/site/about/guidelines.xhtml

**Date:**					
**Start time:**					
**End time:**					
**Training day 3**					
	**Please tick as appropriate**			**If *absent* or *attempted* please provide reasons**	**Other comments**
	**Present**	**Absent**	**Attempted**		
A4 and A6 images: health and wellbeing available	☐	☐	☐		
A4 images: dashboard available	☐	☐	☐		
Writing instruments available (sticky notes, flipchart paper, pens, Blu Tack)	☐	☐	☐		
**Content**					
Session introduced	☐	☐	☐		
Participants divided into 2 groups	☐	☐	☐		
Explained that each participant has been given details of a section of the MLAS course (to be delivered in the next session of training)	☐	☐	☐		
Given instructions for next steps (i.e. time to prepare for delivery of chosen 10-minute section, how 'have a go' session will run)	☐	☐	☐		

For peer review only - http://bmjopen.bmj.com/site/about/guidelines.xhtml

**Date:**					
**Start time:**					
**End time:**					
**Training day 3**					
	**Please tick as appropriate**			**If *absent* or *attempted* please provide reasons**	**Other comments**
	**Present**	**Absent**	**Attempted**		
Offered participants support for preparing for their sessions (e.g. providing relevant resources, explaining content)	☐	☐	☐		
Provided opportunity/time for facilitators to prepare for 10-minute session	☐	☐	☐		
Participants given an opportunity to be involved in discussion and ask questions	☐	☐	☐		
**Session 5: Have a go**					
**Duration of session:**					
**Materials**					
Facilitator curricula	☐	☐	☐		
'Have a go' cards (x2 sets) given out to participants	☐	☐	☐		
Roadmap and accessories displayed	☐	☐	☐		
A4 images: blood clot, bleed displayed	☐	☐	☐		

For peer review only - http://bmjopen.bmj.com/site/about/guidelines.xhtml

**Date:**					
**Start time:**					
**End time:**					
**Training day 3**					
	**Please tick as appropriate**			**If *absent* or *attempted* please provide reasons**	**Other comments**
	**Present**	**Absent**	**Attempted**		
A6 images: risk factors, risks I can/cannot change displayed	☐	☐	☐		
A4 and A6 images: health and wellbeing displayed	☐	☐	☐		
A4 images: dashboard displayed	☐	☐	☐		
Writing instruments given (sticky notes, flipchart paper)	☐	☐	☐		
**Content**					
Give instructions on delivery of 10-minute sessions	☐	☐	☐		
Inform participants of aim of practice delivery (i.e. familiarise self with MLAS, increase confidence)	☐	☐	☐		
Give instructions about giving feedback on facilitator behaviours (3 things you did well)	☐	☐	☐		

For peer review only - http://bmjopen.bmj.com/site/about/guidelines.xhtml

**Date:**					
**Start time:**					
**End time:**					
**Training day 3**					
	**Please tick as appropriate**			**If *absent* or *attempted* please provide reasons**	**Other comments**
	**Present**	**Absent**	**Attempted**		
Each facilitator given an opportunity to deliver their 10-minute session	☐	☐	☐		
Ensured everyone has written 3 positive facilitator behaviours that they saw on a sticky note and give to facilitator	☐	☐	☐		
Provided 3 positive facilitator behaviours and 1 'even better if…'	☐	☐	☐		
Encourage participants to share summaries and feedback on their experience	☐	☐	☐		
**Session 6: Reviewing confidence**					
**Duration of session:**					
**Materials**					
'How confident do I feel?' worksheet	☐	☐	☐		
**Content**					

For peer review only - http://bmjopen.bmj.com/site/about/guidelines.xhtml

**Date:**					
**Start time:**					
**End time:**					
**Training day 3**					
	**Please tick as appropriate**			**If *absent* or *attempted* please provide reasons**	**Other comments**
	**Present**	**Absent**	**Attempted**		
Explores participants' confidence levels to deliver MLAS	☐	☐	☐		
Explore what participants can do to increase their personal confidence to deliver MLAS	☐	☐	☐		
**Session 7: Quality development/Intervention fidelity**					
**Duration of session:**					
**Materials**					
Facilitator behaviours reflection toolkit	☐	☐	☐		
Quality development and intervention fidelity pathway	☐	☐	☐		
**Content**					
Recapped purpose of quality assurance	☐	☐	☐		
Highlighted the importance of self- and peer-reflection	☐	☐	☐		
Discussed facilitator behaviour reflection toolkit	☐	☐	☐		

For peer review only - http://bmjopen.bmj.com/site/about/guidelines.xhtml

**Date:**					
**Start time:**					
**End time:**					
**Training day 3**					
	**Please tick as appropriate**			**If *absent* or *attempted* please provide reasons**	**Other comments**
	**Present**	**Absent**	**Attempted**		
Discussed quality assurance pathway	☐	☐	☐		
Explored what intervention fidelity is					
Described the process for intervention fidelity assessment	☐	☐	☐		
Participants given an opportunity to be involved in discussion (e.g. by giving feedback) and ask questions	☐	☐	☐		
**Session 8: Summary, and post reflections, evaluation**					
**Duration of session:**					
**Materials**					
Training evaluation form given	☐	☐	☐		
Questions relating to MLAS flipchart	☐	☐	☐		
**Content**					

For peer review only - http://bmjopen.bmj.com/site/about/guidelines.xhtml

**Date:**					
**Start time:**					
**End time:**					
**Training day 3**					
	**Please tick as appropriate**			**If *absent* or *attempted* please provide reasons**	**Other comments**
	**Present**	**Absent**	**Attempted**		
Summary of Day 3 training by encouraging participants to share learning about what was covered today	☐	☐	☐		
Provided time for participants to complete training evaluation (day 3) form	☐	☐	☐		
Participants given an opportunity to check if their questions addressed, and to ask any further questions	☐	☐	☐		
Thanked everyone for attending and contributing	☐	☐	☐		
**END OF DAY 3 TRAINING**					

For peer review only - http://bmjopen.bmj.com/site/about/guidelines.xhtml

Supplementary file 3

Training evaluation form

My Life After Stroke - Day 1 - Stroke

Date:________________

Thank you for attending the Stroke day (Day 1) of the My Life After Stroke (MLAS) training. We would value your reflection on the training you have received today in the following areas. Please place a tick in the relevant box, indicating how much you agree or disagree with each of the following statements:

	Strongly agree	Agree	Neither agree nor disagree	Disagree	Strongly disagree
I am aware of the cognitive problems that can occur following stroke					
I am aware of some strategies that may help stroke survivors manage the effects of cognitive problems					
I have an understanding of the emotional problems that may follow a stroke					
I am aware of some strategies to support stroke survivors manage the effects of their emotional problems					
I have an appreciation of the effects having a stroke could put on relationships					
I am aware of some strategies that may help stroke survivors manage the effects on their relationships					
I have an awareness of communication problems following a stroke					
I have an awareness of how to communicate effectively with stroke survivors					

Continued overleaf…

For peer review only

Day 1 MLAS – Stroke evaluation continued…

	Strongly agree	Agree	Neither agree nor disagree	Disagree	Strongly disagree
I had the opportunity to ask questions and for these to be addressed					
I learnt something new today					

We would value your opinion on the training you attended today. Please answer the following questions:

Which were the three most helpful aspects of the training to you?<br>1.<br>2.<br>3.
Which were the three least helpful aspects of the training to you?<br>1.<br>2.<br>3.
Which part of the training did you find most enjoyable?
Please identify at least one way that the training could be improved?
Any other comments?

Thank you for completing this evaluation sheet

For peer review only

Training evaluation form

My Life After Stroke - Day 2 – Core training

Date:__________________

Thank you for attending the Core day (Day 2) of the My Life After Stroke (MLAS) training. We would value your reflection on the training you have received today in the following areas. Please place a tick in the relevant box, indicating how much you agree or disagree with each of the following statements:

	Strongly agree	Agree	Neither agree nor disagree	Disagree	Strongly disagree
I am aware of the aims of the MLAS programme					
I am aware of why MLAS has been developed					
My role as an MLAS facilitator is to:					
• Demonstrate a curious, non-judgemental approach towards participants					
• Support participants to explore their personal thoughts and feelings					
• Avoid giving specific advice and instead support participants to develop their own solutions and strategies to the challenges they face					
• Facilitate the groups awareness of sources of support and information					
• Promote the sharing of knowledge and ideas between participants and sign post where appropriate					
I have an understanding of the theories that underpin the MLAS programme					
I have an understanding of how these theories have been integrated practically into the MLAS programme					

Continued overleaf…

For peer review only

Day 2 MLAS Core evaluation continued…

	Strongly agree	Agree	Neither agree nor disagree	Disagree	Strongly disagree
I had the opportunity to ask questions and for these to be addressed					
I have learnt new skills					
I believe I can apply the facilitation skills					
I understand the importance of quality assurance					

I have been given an MLAS curriculum. **Yes / No** (delete as appropriate)

We would value your opinion on the training you attended today. Please answer the following questions:

Which were the three most helpful aspects of the training to you?<br>1.<br>2.<br>3.
Which were the three least helpful aspects of the training to you?<br>1.<br>2.<br>3.
Which part of the training did you find most enjoyable?
Please identify at least one way that the training could be improved?
Any other comments?

Thank you for completing this evaluation sheet

For peer review only

Training evaluation form

My Life After Stroke - Day 3 – MLAS-specific training

Date:_________________

Thank you for attending the MLAS-specific day (Day 3) of the My Life After Stroke (MLAS) training. We would value your reflection on the training you have received today in the following areas. Please place a tick in the relevant box, indicating how much you agree or disagree with each of the following statements:

	Strongly agree	Agree	Neither agree nor disagree	Disagree	Strongly disagree
I am aware of how to use the MLAS curriculum					
I have an understanding of the content of the MLAS programme					
I have an understanding of how MLAS can be delivered					
I had the opportunity to see the resources that are used during the MLAS programme					
I found the 'having a go' session useful					
I feel able to begin to plan delivering MLAS					
I feel confident that I will be able to facilitate MLAS					
I had the opportunity to ask questions and for these to be addressed					
I have learnt new skills					
I believe I can apply the skills					

I had the opportunity to deliver a section of the curriculum:

Yes / No / Don't know (please circle)

I received a copy of the Reflection Toolkit:

Yes / No / Don't know (please circle)

Continued overleaf…

For peer review only

Day 3 MLAS-specific evaluation continued…

We would value your opinion on the training you attended today. Please answer the following questions:

Which were the three most helpful aspects of the training to you?<br>1.<br><br>2.<br><br>3.
Which were the three least helpful aspects of the training to you?<br>1.<br><br>2.<br><br>3.
Which part of the training did you find most enjoyable?
Please identify at least one way that the training could be improved?
Any other comments?

Thank you for completing this evaluation sheet

For peer review only

IPCAS trial provider process evaluation/fidelity checklist

**Site ID:**			**Time of call:**		**Date: of coding:**		
**Spoke to (role):**			**Conducted by:**		**Duration of call:**		
**Activity**			**Tick as appropriate**			**If *not done* or *attempted*, please provide reason for not delivering (e.g. ran out of time, forgot, deemed unnecessary).**	**Other comments**
			**Done**	**Partially done**	**Not done**		
**Structured review**	1. 15-item checklist of needs	a) Patient completed checklist *(Thinking back to the most recent review that you did… Did the patient bring a completed checklist of needs?)*					
		b) Discussion of up to 3 needs (as ranked by importance by patient)					
	2. Physical check *(Did you conduct any physical checks at all? If yes what were these? If no/unsure, explore reasons for this (e.g. might've already been done recently))*	a) Blood pressure					
		b) Medication review					
		c) Other (specify)					
	3. Development of action plan(s)	a) Discuss action plan *(Together with the stroke survivor, did you discuss any plans of action going forward to address their needs? If no/unsure, explore reasons why)*					
		b) Log and review of agreed actions *(Cannot be filled if answer to a) is no; however, if yes, ask: Did you make a note of these actions and review or agree it with the patient?)*					
	4. MLAS	a) Provided leaflet about MLAS					
		b) Provided instructions for accessing MLAS					
	5. Direct point of contact to stroke survivor and carer(s)	a) Explain 'direct point of contact' service *(I will now ask questions about the Direct Point of Contact service. Did you discuss/explain the service to the patient)*					
		b) Provide instructions on how to contact practice at the end of review					
	6. Service mapping tool *(When giving information and/or advice, did you refer to the searchable service mapping tool provided by us?)*						
		**TOTAL**	0	0	0		
		**PERCENTAGE**	0	0	0		
**Use of direct point of contact** *(Following on from my previous questions, since the reviews you conducted, has anyone called into the Direct Point of Contact service? If yes, think back to the most recent call. Did you signpost the patient to a specialist service? What about a community-based service?)*	1. Signposting to services	a) Signpost patient to specialist service					
		b) Signpost patient to community service					
	2. Use of service mapping for signposting (as above) *(When you offered information to the patient about either specialist/community services, did you make use of the service mapping tool (i.e. directory) at all?)*						
	3. Provide support and advice	a) Advice for stroke-specific issues					
		b) Other brief telephone support *(Beyond stroke-specific issues, did you offer any other support over the phone?)*					
	4. Arrange follow-up appointment(s)						
	5. Case management *(Finally, during the call, did you offer the patient anything else other than what we had already discussed? E.g. (comprehensive assessment of needs; development of an individualised care plan in collaboration with the service user and relevant others (including families and carers and other staff involved in the service user's care; any additional care given))*						
		**TOTAL**	0	0	0		
		**PERCENTAGE**	0	0	0		
**Enhanced communication pathways**	1. Initial meeting *(We are coming towards the end of the call now, and I wanted to ask some questions about your contact with colleagues in secondary care. Have you had your initial meeting? If no, note reasons why. If yes, ask: Did you have a discussion about your roles, etc. (Note whether meeting was via videoconference or face-to-face))*	a) Discussion of roles/services provided/contact details (video recorded)					
		b) Agreed action plan regarding communication between primary care staff and specialist staff *(In this meeting, did you agree a plan going forward about how best to communicate with each other?)*					
	2. Further contact	a) Meeting (e.g. teleconference, face-to-face, videoconference) *(Since the initial meeting, did you have any further contacts? Tell me about them (then tick off if there was a meeting, or email/phone contact))*					
		b) Other contact (e.g. telephone, email, letter)					
		**TOTAL**	0	0	0		
		**PERCENTAGE**	0	0	0		
**Duration of structured review**	On average, how long did each review run for?						

END OF CHECKLIST

For peer review only

For peer review only - http://bmjopen.bmj.com/site/about/guidelines.xhtml

Supplementary file 5

**Assessor's name:**					
**Group/facilitator ID:**					
**Date:**					
**Week 1: Group session 1, Stroke Journey (2.5 hours, with breaks)**					
**Section A: Welcome/introduction**					
**Facilitator(s):**					
**Start time:**					
**End time:**					
	**Tick as appropriate**				
	**Present**	**Absent**	**Partially done**	**If *partially done*, please provide reasons (e.g. ran out of time, forgot, deemed unnecessary)**	**Other comments**
Content					
A1) Welcome/introductions (including housekeeping details, course handbook, course background)	☐	☐	☐		
A2) Introduction to the guidelines for working together as a group	☐	☐	☐		
A3) Opportunity for participants to ask questions	☐	☐	☐		
A4) Introduction to communication aids	☐	☐	☐		
A5) Participants' important questions	☐	☐	☐		
Materials					
A6) Flipchart – names	☐	☐	☐		
A7) Flipcharts – what would you like to get from the course?; what is your important question?	☐	☐	☐		
A8) Flipchart – working together as a group	☐	☐	☐		
Key facilitator behaviours for welcome/introduction					
A9) Uses and refers to participants' comments/quotes	☐	☐	☐		

For peer review only

**Section B: Stroke story**					
**Facilitator(s):**					
**Start time:**					
**End time:**					
	**Tick as appropriate**				
	**Present**	**Absent**	**Partially done**	**If *partially done*, please provide reasons (e.g. ran out of time, forgot, deemed unnecessary)**	**Other comments**
Content					
B1) Stroke story/introduction to narrative approach (including roadmap)	☐	☐	☐		
B2) Eliciting skills, strengths and strategies from participants	☐	☐	☐		
Materials					
B3) Flipchart – skills and strategies	☐	☐	☐		
Key facilitator behaviours for stroke story					
B4) Uses resources and/or participant handbooks appropriately to engage participants and enhance their understanding of the session's key outcomes	☐	☐	☐		
B5) Facilitates people to share their stories about attempts to manage their condition	☐	☐	☐		
B6) Appropriately adapts communication/environment/session delivery to meet the needs of the stroke survivors	☐	☐	☐		

For peer review only - http://bmjopen.bmj.com/site/about/guidelines.xhtml

**Section C: What is stroke?**					
**Facilitator(s):**					
**Start time:**					
**End time:**					
	**Tick as appropriate**				
	**Present**	**Absent**	**Partially done**	**If *partially done*, please provide reasons (e.g. ran out of time, forgot, deemed unnecessary)**	**Other comments**
Content					
C1) What is stroke (including impacts of stroke on individual – physically, emotionally, psychologically and socially)?	☐	☐	☐		
C2) Discussed neuroplasticity (the brain's ability to create new neural pathways and relearn skills	☐	☐	☐		
Materials					
C3) Flipchart – body outline	☐	☐	☐		
C4) Record of 'what is stroke?' (i.e. written on body outline/sticky notes)	☐	☐	☐		
C5) Record of 'effects of stroke on body/person' (i.e. written on body outline/sticky notes)	☐	☐	☐		
Key facilitator behaviours for what is stroke					
C6) Prompts participant to talk about their thoughts and explores misconceptions and gaps in knowledge	☐	☐	☐		
C7) Uses resources and/or participant handbooks appropriately to engage participants and enhance their understanding of the session's key outcomes	☐	☐	☐		

For peer review only - http://bmjopen.bmj.com/site/about/guidelines.xhtml

**Section D: Stroke passenger**					
**Facilitator(s):**					
**Start time:**					
**End time:**					
	**Tick as appropriate**				
	**Present**	**Absent**	**Partially done**	**If *partially done*, please provide reasons (e.g. ran out of time, forgot, deemed unnecessary)**	**Other comments**
Content					
D1) Discussed 'Stroke passenger' analogy	☐	☐	☐		
D2) Eliciting feelings and emotions about stroke	☐	☐	☐		
D3) Provided opportunity for separate carer/stroke survivor discussions	☐	☐	☐		
D4) Discussing skills and coping strategies for living with stroke passenger	☐	☐	☐		
Materials					
D5) Flipchart – how do you feel/think about sharing your life with stroke (carers)?	☐	☐	☐		
D6) Flipchart – how do you feel/think about sharing your life with stroke (stroke survivors)?	☐	☐	☐		
Key facilitator behaviours for stroke passenger					
D7) Prompts participants to express and explore their feelings about the condition or behaviour	☐	☐	☐		
D8) Facilitates people to share their stories about attempts to manage their condition	☐	☐	☐		
D9) Appropriately adapts communication/environment/session delivery to meet the needs of the stroke survivors	☐	☐	☐		
D10) Works as a team with co-facilitator	☐	☐	☐		

For peer review only - http://bmjopen.bmj.com/site/about/guidelines.xhtml

**Section E: What will I do now?**					
**Facilitator(s):**					
**Start time:**					
**End time:**					
	**Tick as appropriate**				
	**Present**	**Absent**	**Partially done**	**If *partially done*, please provide reasons (e.g. ran out of time, forgot, deemed unnecessary)**	**Other comments**
Content					
E1) Session summary/recap of session key outcomes	☐	☐	☐		
E2) 'What will I do now?' worksheet	☐	☐	☐		
Materials					
E3) 'What will I do now?' worksheet in handbook	☐	☐	☐		
Key facilitator behaviours for what will I do now					
E4) Uses resources and/or participant handbooks appropriately to engage participants and enhance their understanding of the session's key outcomes	☐	☐	☐		
E5) Prompts group to summarise their key messages from the session.	☐	☐	☐		
E6) Prompts participants to talk about what they are going to do as a result of the session.	☐	☐	☐		

For peer review only - http://bmjopen.bmj.com/site/about/guidelines.xhtml

Overall facilitator behaviours					
	**Tick as appropriate**				
	**Present**	**Absent**	**Partially done**	**If *partially done*, please provide reasons (e.g. ran out of time, forgot, deemed unnecessary)**	**Other comments**
1. Uses non-judgmental statements in response to participants' verbal utterances	☐	☐	☐		
2. The facilitator seeks clarification of participants contribution	☐	☐	☐		
3. Avoids giving their own opinion	☐	☐	☐		
4. Encourages the group to discuss/answer their own question	☐	☐	☐		
5. Engages participants using rapport building skills	☐	☐	☐		
6. Notices tone/dynamics within the group, acknowledges these and uses them to manage the group	☐	☐	☐		
7. The facilitator uses strategies to manage time within session	☐	☐	☐		
8. Uses active listening skills and open body language to facilitate group/participant talk	☐	☐	☐		
9. Appropriately adapts delivery to meet the needs of the stroke survivors	☐	☐	☐		
10. Works as a team with co-facilitator	☐	☐	☐		

For peer review only

For peer review only - http://bmjopen.bmj.com/site/about/guidelines.xhtml

Assessor comments/notes

For peer review only

For peer review only - http://bmjopen.bmj.com/site/about/guidelines.xhtml

**Assessor's name:**					
**Group/facilitator ID:**					
**Date:**					
**Week 2/3 (circle as appropriate): Managing health and wellbeing (2.5 hours, with breaks)**					
**Section A: Welcome back [20 mins]**					
**Facilitator(s):**					
**Start time:**					
**End time:**					
	**Tick as appropriate**				
	**Present**	**Absent**	**Partially done**	**If *partially done*, please provide reasons (e.g. ran out of time, forgot, deemed unnecessary)**	**Other comments**
Content					
A1) Welcome (including housekeeping details, and ground rules)	☐	☐	☐		
A2) Session outline	☐	☐	☐		
A3) Introduction/recap of managing health and wellbeing handbook and roadmap <u>or</u> roadblocks handbook and roadmap	☐	☐	☐		
A4) Previous week's worksheets; stories and reflections from past week	☐	☐	☐		
A5) Opportunity for participants to ask questions	☐	☐	☐		
Materials					
A6) Roadmap	☐	☐	☐		
A7) Previous week's (relevant) flipcharts on view	☐	☐	☐		
Key facilitator behaviours for welcome					
A8) Uses resources and participant handbooks appropriately to engage participants and enhance understanding of the session's key outcomes	☐	☐	☐		
A9) Prompts the participants to reflect on last week's session and activities	☐	☐	☐		

For peer review only - http://bmjopen.bmj.com/site/about/guidelines.xhtml

**Section B: Managing health and wellbeing [40 mins]**					
**Facilitator(s):**					
**Start time:**					
**End time:**					
	**Tick as appropriate**				
	**Present**	**Absent**	**Partially done**	**If *partially done*, please provide reasons (e.g. ran out of time, forgot, deemed unnecessary)**	**Other comments**
Content					
B1) Risk factors for stroke	☐	☐	☐		
B2) Ways to manage health and wellbeing	☐	☐	☐		
Materials					
B3) A6 headings: 'risks I can change' and 'risks I cannot change'	☐	☐	☐		
B4) A6 images: risk factors	☐	☐	☐		
B5) A4 headings: health and wellbeing	☐	☐	☐		
B6) A6 images: health and wellbeing	☐	☐	☐		
Key facilitator behaviours for managing health and wellbeing					
B7) Facilitates people to share their stories about attempts to manage their condition	☐	☐	☐		
B8) Uses resources and participant handbooks appropriately to engage participants and enhance understanding of the session's key outcomes	☐	☐	☐		
B9) Prompts participant to talk about their thoughts and explores misconceptions and gaps in knowledge	☐	☐	☐		
B10) Encourages the group to discuss/answer their own questions	☐	☐	☐		
B11) Works as a team with co-facilitator	☐	☐	☐		

For peer review only - http://bmjopen.bmj.com/site/about/guidelines.xhtml

**Section C: Making changes [15 mins]**					
**Facilitator(s):**					
**Start time:**					
**End time:**					
	**Tick as appropriate**				
	**Present**	**Absent**	**Partially done**	**If *partially done*, please provide reasons (e.g. ran out of time, forgot, deemed unnecessary)**	**Other comments**
Content					
C1) Elicits feelings and reactions to change	☐	☐	☐		
C2) Discussion of pros and cons of changes	☐	☐	☐		
Materials					
C3) Flipchart – pros and cons grid	☐	☐	☐		
Key facilitator behaviours for making changes					
C4) Prompts participants to discuss their thoughts about possible changes to self-management.	☐	☐	☐		
C5) Prompts participants to review the impact of possible choices on their future health.	☐	☐	☐		
C6) Prompts or allows participants to express and explore their feelings about the condition or behaviour	☐	☐	☐		

For peer review only

For peer review only - http://bmjopen.bmj.com/site/about/guidelines.xhtml

**Section D: Making a plan [35 mins]**					
**Facilitator(s):**					
**Start time:**					
**End time:**					
	**Tick as appropriate**				
	**Present**	**Absent**	**Partially done**	**If *partially done*, please provide reasons (e.g. ran out of time, forgot, deemed unnecessary)**	**Other comments**
Content					
D1) Identifying challenges to planning, problem-solving	☐	☐	☐		
D2) Making a SMART plan	☐	☐	☐		
Materials					
D3) A4 resource: 'My action plan'	☐	☐	☐		
D4) 'My action plan' in handbook	☐	☐	☐		
Key facilitator behaviours for making a plan					
D5) Prompts participants to discuss their thoughts about possible changes to self-management.	☐	☐	☐		
D6) Prompts the participants to reflect on their goals/plans	☐	☐	☐		
D7) Prompts the individual or group to problem solve possible barriers to change (e.g. their desired changes or possible barriers to self-management)	☐	☐	☐		
D8) Encourages the group to discuss/answer their own questions	☐	☐	☐		
D9) Prompts group to summarise key messages from the session	☐	☐	☐		

For peer review only

For peer review only - http://bmjopen.bmj.com/site/about/guidelines.xhtml

**Section E: What will I do now? [10 mins]**					
**Facilitator(s):**					
**Start time:**					
**End time:**					
	**Tick as appropriate**				
	**Present**	**Absent**	**Partially done**	**If *partially done*, please provide reasons (e.g. ran out of time, forgot, deemed unnecessary)**	**Other comments**
Content					
E1) Session summary provided	☐	☐	☐		
E2) 'What will I do now?' worksheet	☐	☐	☐		
Materials					
E3) 'What will I do now?' worksheet in handbook	☐	☐	☐		
Key facilitator behaviours for what will I do now					
E4) Uses resources and participant handbooks appropriately to engage participants and enhance understanding of the session's key outcomes	☐	☐	☐		
E5) Prompts participants to talk about what they are going to do as a result of the session	☐	☐	☐		
E6) Prompts group to summarise their key messages from the session.	☐	☐	☐		

For peer review only

Overall facilitator behaviours					
	**Tick as appropriate**				
	**Present**	**Absent**	**Partially done**	**If *partially done*, please provide reasons (e.g. ran out of time, forgot, deemed unnecessary)**	**Other comments**
1. Uses non-judgmental statements in response to participants' verbal utterances	☐	☐	☐		
2. The facilitator seeks clarification of participants contribution	☐	☐	☐		
3. Avoids giving their own opinion	☐	☐	☐		
4. Encourages the group to discuss/answer their own questions and/or elicits suggestions from participants	☐	☐	☐		
5. Engages participants using rapport building skills	☐	☐	☐		
6. Notices tone/dynamics within the group, acknowledges these and uses them to manage the group	☐	☐	☐		
7. The facilitator uses strategies to manage time within session	☐	☐	☐		
8. Uses open questions, reflections, appropriate silences and open body language to facilitate group/participant talk	☐	☐	☐		
9. Adapts communication/environment/ session delivery to meet the needs of the stroke survivors	☐	☐	☐		
10. Works as a team with co-facilitator	☐	☐	☐		

For peer review only

For peer review only - http://bmjopen.bmj.com/site/about/guidelines.xhtml

Assessor comments/notes

For peer review only

For peer review only - http://bmjopen.bmj.com/site/about/guidelines.xhtml

**Assessor's name:**					
**Group/facilitator ID:**					
**Date:**					
Week 2/3 (circle as appropriate): Roadblocks (2.5 hrs, with breaks)					
**Section A: Welcome back**					
**Facilitator(s):**					
**Start time:**					
**End time:**					
	**Tick as appropriate**				
	**Present**	**Absent**	**Partially done**	**If *partially done*, please provide reasons (e.g. ran out of time, forgot, deemed unnecessary)**	**Other comments**
Content					
A1) Welcome (including housekeeping details, and ground rules)	☐	☐	☐		
A2) Session outline	☐	☐	☐		
A3) Introduction/recap of managing health and wellbeing handbook and roadmap <u>or</u> roadblocks handbook and roadmap	☐	☐	☐		
A4) Previous week's worksheets; stories and reflections from past week	☐	☐	☐		
A5) Opportunity for participants to ask questions					
Materials					
A6) Roadmap	☐	☐	☐		
A7) Previous week's (relevant) flipcharts on view	☐	☐	☐		
Key facilitator behaviours for welcome back					
A8) Uses resources and participant handbooks appropriately to engage participants and enhance understanding of the session's key outcomes	☐	☐	☐		
A9) Prompts the participants to reflect on last week's session and activities	☐	☐	☐		

For peer review only - http://bmjopen.bmj.com/site/about/guidelines.xhtml

**Section B: Roadblocks on my journey**					
**Facilitator(s):**					
**Start time:**					
**End time:**					
	**Tick as appropriate**				
	**Present**	**Absent**	**Partially done**	**If *partially done*, please provide reasons (e.g. ran out of time, forgot, deemed unnecessary)**	**Other comments**
Content					
B1) Fictional character facing loss of confidence after stroke (using STOP sign)	☐	☐	☐		
B2) Eliciting thoughts, feelings, sensations, or behaviours as a response to loss of confidence	☐	☐	☐		
B3) Discuss what might help the fictional character, and impact of this on thoughts, feelings, sensations, or behaviours (using GO sign)	☐	☐	☐		
B4) 'What do my roadblocks look like?' worksheet	☐	☐	☐		
Materials					
B5) Roadmap	☐	☐	☐		
B6) Flipcharts – STOP and GO	☐	☐	☐		

For peer review only

For peer review only - http://bmjopen.bmj.com/site/about/guidelines.xhtml

Key facilitator behaviours for roadblocks					
	**Tick as appropriate**				
	**Present**	**Absent**	**Partially done**	**If *partially done*, please provide reasons (e.g. ran out of time, forgot, deemed unnecessary)**	**Other comments**
B7) Facilitates people to share their stories about attempts to manage their condition, and the challenges/roadblocks they face.	☐	☐	☐		
B8) Prompts or allows participants to express and explore their feelings about (the condition or behaviour) during the session	☐	☐	☐		
B9) Encourages the group to discuss/answer their own questions	☐	☐	☐		
B10) Uses and refers to participants' comments/quotes	☐	☐	☐		
B11) Prompts the individual or group to problem solve possible barriers to change (e.g. their desired changes or possible barriers to self-management)	☐	☐	☐		
B12) Prompts participants to review the impact of possible choices on their future health.	☐	☐	☐		

For peer review only

**Section C: Working it out**					
**Facilitator(s):**					
**Start time:**					
**End time:**					
	**Tick as appropriate**				
	**Present**	**Absent**	**Partially done**	**If *partially done*, please provide reasons (e.g. ran out of time, forgot, deemed unnecessary)**	**Other comments**
Content					
C1) Problems stroke survivors could face Next steps (including session's key outcomes, 'what will I do now?' worksheet, and follow-up appointments)	☐	☐	☐		
C2) Introduce problem-solving approach	☐	☐	☐		
C3) Use fictional character to choose a problem and apply problem-solving approach	☐	☐	☐		
C4) How decisions may be made on the best course of action for solving the problem	☐	☐	☐		
C5) What to do when problems aren't solvable now	☐	☐	☐		
C6) 'Working it out' worksheet	☐	☐	☐		
Materials					
C7) Flipcharts with headings: 1) in my power to solve/change or not in my power to solve/change; 2) 'what could help'; 3) solution: immediate results/long-term results	☐	☐	☐		
C8) Previous flipcharts (e.g. what are my roadblocks; skills and strategies toolkit)	☐	☐	☐		

For peer review only - http://bmjopen.bmj.com/site/about/guidelines.xhtml

Key facilitator behaviours for working it out					
	**Tick as appropriate**				
	**Present**	**Absent**	**Partially done**	**If *partially done*, please provide reasons (e.g. ran out of time, forgot, deemed unnecessary)**	**Other comments**
C9) Uses resources and participant handbooks appropriately to engage participants and enhance understanding of the session's key outcomes	☐	☐	☐		
C10) Uses and refers to participants' comments/quotes	☐	☐	☐		
C11) Prompts participants to discuss their thoughts about possible changes to self-management.	☐	☐	☐		
C12) Prompts participants to review the impact of possible choices on their future health.	☐	☐	☐		
C13) Prompts the individual or group to problem solve possible barriers to change (e.g. their desired changes or possible barriers to self-management)	☐	☐	☐		

For peer review only - http://bmjopen.bmj.com/site/about/guidelines.xhtml

**Section D: What will I do now**					
**Facilitator(s):**					
**Start time:**					
**End time:**					
	**Tick as appropriate**				
	**Present**	**Absent**	**Partially done**	**If *partially done*, please provide reasons (e.g. ran out of time, forgot, deemed unnecessary)**	**Other comments**
Content					
D1) Recap key outcomes	☐	☐	☐		
D2) 'What will I do now?' worksheet	☐	☐	☐		
Materials					
D3) 'What will I do now?' worksheet	☐	☐	☐		
Key facilitator behaviours for what will I do now					
D4) Uses resources and participant handbooks appropriately to engage participants and enhance understanding of the session's key outcomes	☐	☐	☐		
D5) Prompts participants to talk about what they are going to do as a result of the session.	☐	☐	☐		

For peer review only

For peer review only - http://bmjopen.bmj.com/site/about/guidelines.xhtml

Overall facilitator behaviours					
	**Tick as appropriate**				
	**Present**	**Absent**	**Partially done**	**If *partially done*, please provide reasons (e.g. ran out of time, forgot, deemed unnecessary)**	**Other comments**
1. Prompts group to summarise their key messages from the session.	☐	☐	☐		
2. The facilitator seeks clarification of participants contribution	☐	☐	☐		
3. Avoids giving their own opinion	☐	☐	☐		
4. Encourages the group to discuss/answer their own questions and/or elicits suggestions from participants	☐	☐	☐		
5. Engages participants using rapport building skills	☐	☐	☐		
6. Notices tone/dynamics within the group, acknowledges these and uses them to manage the group	☐	☐	☐		
7. The facilitator uses strategies to manage time within session	☐	☐	☐		
8. Uses open questions, reflections, appropriate silences and open body language to facilitate group/participant talk	☐	☐	☐		
9. Adapts communication/environment/ session delivery to meet the needs of the stroke survivors	☐	☐	☐		
10. Works as a team with co-facilitator	☐	☐	☐		

For peer review only

For peer review only - http://bmjopen.bmj.com/site/about/guidelines.xhtml

Assessor comments/notes

For peer review only

For peer review only - http://bmjopen.bmj.com/site/about/guidelines.xhtml

**Assessor's name:**					
**Group/facilitator ID:**					
**Date:**					
**Week 4: Moving forward on my journey (2.5 hours, with breaks)**					
**Section A: Welcome back**					
**Facilitator(s):**					
**Start time:**					
**End time:**					
	**Tick as appropriate**				
	**Present**	**Absent**	**Partially done**	**If *partially done*, please provide reasons (e.g. ran out of time, forgot, deemed unnecessary)**	**Other comments**
Content					
A1) Welcome (including housekeeping details, handbook and ground rules)	☐	☐	☐		
A2) Session outline	☐	☐	☐		
A3) Previous week's action plan/working it out worksheets	☐	☐	☐		
A4) Recap roadmap	☐	☐	☐		
Materials					
A5) Roadmap	☐	☐	☐		
A6) Previous week's (relevant) flipcharts on view	☐	☐	☐		
A7) Moving forward on my journey handbook	☐	☐	☐		
Key facilitator behaviours for welcome back					
A8) Re-engages participants in the journey analogy and resources	☐	☐	☐		
A9) Prompts the participants to reflect on last week's session and activities	☐	☐	☐		

For peer review only - http://bmjopen.bmj.com/site/about/guidelines.xhtml

**Section B: Your relationships**					
**Facilitator(s):**					
**Start time:**					
**End time:**					
	**Tick as appropriate**				
	**Present**	**Absent**	**Partially done**	**If *partially done*, please provide reasons (e.g. ran out of time, forgot, deemed unnecessary)**	**Other comments**
Content					
B1) Ways relationships can be affected by stroke	☐	☐	☐		
B2) Problem-solving exercise in relation to a relationship change	☐	☐	☐		
Materials					
B3) Flipcharts – how can relationships change (one for stroke survivors, one for carers/supporters as applicable)	☐	☐	☐		
B4) Flipcharts – 'what is the problem exactly'; 'what could help'; 'solution: immediate results/long-term results'	☐	☐	☐		

For peer review only - http://bmjopen.bmj.com/site/about/guidelines.xhtml

Key facilitator behaviours for your relationships					
	**Tick as appropriate**				
	**Present**	**Absent**	**Partially done**	**If *partially done*, please provide reasons (e.g. ran out of time, forgot, deemed unnecessary)**	**Other comments**
B5) Prompts or allows participants to express and explore their feelings about (the condition or behaviour) during the session	☐	☐	☐		
B6) Uses and refers to participants' comments/quotes	☐	☐	☐		
B7) Prompts the individual or group to problem solve possible barriers to change (e.g. their desired changes or possible barriers to self-management)	☐	☐	☐		
B8) Prompts participants to review the impact of possible choices on their future health.	☐	☐	☐		
B9) Adapts communication, environment, or session delivery to meet the needs of the stroke survivors	☐	☐	☐		

For peer review only

For peer review only - http://bmjopen.bmj.com/site/about/guidelines.xhtml

**Section C: Revisiting your stroke journey**					
**Facilitator(s):**					
**Start time:**					
**End time:**					
	**Tick as appropriate**				
	**Present**	**Absent**	**Partially done**	**If *partially done*, please provide reasons (e.g. ran out of time, forgot, deemed unnecessary)**	**Other comments**
Content					
C1) Recap the roadmap/journey analogy	☐	☐	☐		
C2) Summary of key messages from each session (e.g. session outcomes/personal take-home messages/skills and strategies discussed during the course)	☐	☐	☐		
C3) Elicit feelings about moving forward from MLAS	☐	☐	☐		
Materials					
C4) Flipchart – 'skills and strategies toolkit'	☐	☐	☐		
Key facilitator behaviours for revisiting your stroke journey					
C5) Facilitates the group's recapping of the journey analogy (e.g. prompts group to summarise their key messages from the session)	☐	☐	☐		
C6) Prompts participants to reflect on where they feel they are now	☐	☐	☐		

For peer review only

For peer review only - http://bmjopen.bmj.com/site/about/guidelines.xhtml

**Section D: How will you move forward?**					
**Facilitator(s):**					
**Start time:**					
**End time:**					
	**Tick as appropriate**				
	**Present**	**Absent**	**Partially done**	**If *partially done*, please provide reasons (e.g. ran out of time, forgot, deemed unnecessary)**	**Other comments**
Content					
D1) Discussion of 'warning lights' for physical/emotional health and prevention of these	☐	☐	☐		
D2) Loneliness from the perspective of the fictional character (may also use problem-solving exercise)	☐	☐	☐		
D3) The directory	☐	☐	☐		
Materials					
D4) A4 dashboard images	☐	☐	☐		
D5) Flipchart - 'Skills and strategies toolkit'	☐	☐	☐		
D6) A4 directory	☐	☐	☐		
Key facilitator behaviours for how will you move forward					
D7) Prompts or allows participants to express and explore their feelings about the condition or behaviour	☐	☐	☐		
D8) Encourages the group to discuss/answer their own questions	☐	☐	☐		
D9) Uses and refers to participants' comments/quotes	☐	☐	☐		

For peer review only - http://bmjopen.bmj.com/site/about/guidelines.xhtml

**Section E: Next steps**					
**Facilitator(s):**					
**Start time:**					
**End time:**					
	**Tick as appropriate**				
	**Present**	**Absent**	**Partially done**	**If *partially done*, please provide reasons (e.g. ran out of time, forgot, deemed unnecessary)**	**Other comments**
Content					
E1) Recap session's key outcomes	☐	☐	☐		
E2) 'What will I do now?' worksheet	☐	☐	☐		
E3) Organise follow-up appointments	☐	☐	☐		
Materials					
E4) 'What will I do now?' worksheet in the handbook	☐	☐	☐		
E5) Flipchart – 'what would you like to get from the course?/What is your important question?'	☐	☐	☐		
Key facilitator behaviours for next steps					
E6) Prompts group to summarise their key messages from the session.	☐	☐	☐		
E7) Prompts participants to talk about what they are going to do as a result of the course.	☐	☐	☐		
E8) Encourages the group to discuss/answer their own questions	☐	☐	☐		

For peer review only - http://bmjopen.bmj.com/site/about/guidelines.xhtml

Overall facilitator behaviours					
	**Tick as appropriate**				
	**Present**	**Absent**	**Partially done**	**If *partially done*, please provide reasons (e.g. ran out of time, forgot, deemed unnecessary)**	**Other comments**
1. Uses non-judgmental statements in response to participants' verbal utterances	☐	☐	☐		
2. The facilitator seeks clarification of participants contribution	☐	☐	☐		
3. Avoids giving their own opinion	☐	☐	☐		
4. Encourages the group to discuss/answer their own questions and/or elicits suggestions from participants	☐	☐	☐		
5. Engages participants using rapport building skills	☐	☐	☐		
6. Notices tone/dynamics within the group, acknowledges these and uses them to manage the group	☐	☐	☐		
7. The facilitator uses strategies to manage time within session	☐	☐	☐		
8. Uses open questions, reflections, appropriate silences and open body language to facilitate group/participant talk	☐	☐	☐		
9. Adapts communication/environment/ session delivery to meet the needs of the stroke survivors	☐	☐	☐		
10. Works as a team with co-facilitator	☐	☐	☐		

For peer review only

For peer review only - http://bmjopen.bmj.com/site/about/guidelines.xhtml

Assessor comments/notes

For peer review only

For peer review only - http://bmjopen.bmj.com/site/about/guidelines.xhtml

IPCAS trial process evaluation checklist
MLAS facilitator delivery - DESMOND Observation Tool Record Sheet
Group Session 1

Instructions: DOT at 10s (i.e. at the second bleep)

Session A: Welcome/Introduction (30 min)

Facilitator's name:

Assessor's name:

Start time:

End time:

Facilitator talk	Participant talk	Other
Total A:	Total B:	Total C:
Total score= A÷(A+B+C)*100=		

Session B: Stroke story (20 min)

Facilitator's name:

Assessor's name:

Start time:

End time:

Facilitator talk	Participant talk	Other
Total A:	Total B:	Total C:
Total score= A÷(A+B+C)*100=		

For peer review only

IPCAS trial process evaluation checklist

Session C: What is stroke? (25 min)

Facilitator's name:

Assessor's name:

Start time:

End time:

Facilitator talk	Participant talk	Other
Total A:	Total B:	Total C:
Total score= A÷(A+B+C)*100=		

Session D: Stroke passenger (35 min)

Facilitator's name:

Assessor's name:

Start time:

End time:

Facilitator talk	Participant talk	Other
Total A:	Total B:	Total C:
Total score= A÷(A+B+C)*100=		

Session E: What will I do now? (10 min)

Facilitator

Start time:

End time:

Facilitator talk	Participant talk	Other

For peer review only

IPCAS trial process evaluation checklist

Total A:	Total B:	Total C:
Total score= A÷(A+B+C)*100=		

For peer review only

IPCAS trial process evaluation checklist
MLAS facilitator delivery - DESMOND Observation Tool Record Sheet
Group Session 2/3 (circle as appropriate): Managing Health and Wellbeing

Instructions: DOT at 10s (i.e. at the second bleep)

Session A: Welcome back, recap, and introduction (20 min)

Facilitator's name:

Assessor's name:

Start time:

End time:

Facilitator talk	Participant talk	Other
Total A:	Total B:	Total C:
Total score= A÷(A+B+C)*100=		

Session B: Managing health and wellbeing (40 min)

Facilitator's name:

Assessor's name:

Start time:

End time:

Facilitator talk	Participant talk	Other
Total A:	Total B:	Total C:
Total score= A÷(A+B+C)*100=		

For peer review only

IPCAS trial process evaluation checklist

Session C: Making changes (15 min)

Facilitator's name:

Assessor's name:

Start time:

End time:

Facilitator talk	Participant talk	Other
Total A:	Total B:	Total C:
Total score= A÷(A+B+C)*100=		

Session D: Making a plan (35 min)

Facilitator's name:

Assessor's name:

Start time:

End time:

Facilitator talk	Participant talk	Other
Total A:	Total B:	Total C:
Total score= A÷(A+B+C)*100=		

Session E: What will I do now? (10 min)

Facilitator

Start time:

End time:

Facilitator talk	Participant talk	Other

For peer review only

IPCAS trial process evaluation checklist

Total A:	Total B:	Total C:
Total score= A÷(A+B+C)*100=		

For peer review only

IPCAS trial process evaluation checklist
MLAS facilitator delivery - DESMOND Observation Tool Record Sheet
Group Session 2/3 (circle as appropriate): Roadblocks

Instructions: DOT at 10s (i.e. at the second bleep)

Session A: Welcome back and introduction (20 min)

Facilitator's name:

Assessor's name:

Start time:

End time:

Facilitator talk	Participant talk	Other
Total A:	Total B:	Total C:
Total score= A÷(A+B+C)*100=		

Session B: Roadblocks on my journey (45 min)

Facilitator's name:

Assessor's name:

Start time:

End time:

Facilitator talk	Participant talk	Other
Total A:	Total B:	Total C:
Total score= A÷(A+B+C)*100=		

For peer review only

IPCAS trial process evaluation checklist

Session C: Working it out (45 min)

Facilitator's name:

Assessor's name:

Start time:

End time:

Facilitator talk	Participant talk	Other
Total A:	Total B:	Total C:
Total score= A÷(A+B+C)*100=		

Session D: What will I do now? (10 min)

Facilitator's name:

Assessor's name:

Start time:

End time:

Facilitator talk	Participant talk	Other
Total A:	Total B:	Total C:
Total score= A÷(A+B+C)*100=		

For peer review only

IPCAS trial process evaluation checklist
MLAS facilitator delivery - DESMOND Observation Tool Record Sheet
Group Session 4: Moving Forward on my Journey

Instructions: DOT at 10s (i.e. at the second bleep)

Session A: Welcome back, recap, and introduction (20 min)

Facilitator's name:
Assessor's name:
Start time:
End time:

Facilitator talk	Participant talk	Other
Total A:	Total B:	Total C:
Total score= A÷(A+B+C)*100=		

Session B: Your relationships (45 min)

Facilitator's name:
Assessor's name:
Start time:
End time:

Facilitator talk	Participant talk	Other
Total A:	Total B:	Total C:
Total score= A÷(A+B+C)*100=		

For peer review only

IPCAS trial process evaluation checklist

Session C: Revisiting your stroke journey (10 min)

Facilitator's name:

Assessor's name:

Start time:

End time:

Facilitator talk	Participant talk	Other
Total A:	Total B:	Total C:
Total score= A÷(A+B+C)*100=		

Session D: How will you move forward? (30 min)

Facilitator's name:

Assessor's name:

Start time:

End time:

Facilitator talk	Participant talk	Other
Total A:	Total B:	Total C:
Total score= A÷(A+B+C)*100=		

Session E: What will I do now? (10 min)

Facilitator

Start time:

End time:

Facilitator talk	Participant talk	Other

For peer review only - http://bmjopen.bmj.com/site/about/guidelines.xhtml

For peer review only

IPCAS trial process evaluation checklist

Total A:	Total B:	Total C:
Total score= A÷(A+B+C)*100=		

For peer review only

IPCAS trial patient process evaluation/fidelity checklist - patient							
Site ID:			Participant ID:			Date:	
Time of call:			Conducted by:			Duration of call:	
Activity			Tick as appropriate			If *no* or *unsure*, please provide reasons why/any details	Other comments
			Yes	No	Unsure		
Structured review (attending at the GP surgery)	1. Attended structured review (If no/unsure, ask for reasons why)						
	2. 15-item checklist of needs (If no/unsure, ask for reasons why)	a) Patient completed checklist					
		b) Discussion of up to 3 needs (as ranked by importance by patient) *(Can you tell me which needs you discussed?)*					
	4. Development of action plan(s) (If no/unsure, ask for reasons why)	a) Discussed action plan *(Did you talk about how to address your needs?)*					
		b) Opportunity to log and review agreed actions *(Did you agree a specific plan going forward to address these needs? E.g., book an appointment with physio on Monday)*					
	3. Physical check (If no/unsure, ask for reasons why)	a) Blood pressure					
		b) Medication review *(Did you discuss your medication(s)?)*					
		c) Other (specify) *(Was there anything else that you talked about regarding your physical health?)*					
	5. MLAS (If no/unsure, ask for reasons why)	a) Received leaflet about *MLAS* *(Would you like me to post you one if not?)*					
		b) Received instructions for accessing MLAS (they're on the bottom of the information sheet: "..call Emily...")					
	6. Direct point of contact to stroke survivor and carer(s)	a) Received information about 'direct point of contact' service *(Were you given information about a service in your GP practice where you can get in touch if you have any stroke-related needs/concerns?)*					
		b) Received instructions on how to contact practice at the end of review *(Did you receive instructions about how to access this telephone service?) IF 'NO', FINISH.*					
		TOTAL					
		PERCENTAGE					
Use of direct point of contact (i.e. calling the practice about their stroke-specific needs)	1. Called practice	a) Called practice *(Since your appointment, have you phoned the practice about your stroke? If no/unsure, ask for reasons why. After asking for reasons why, end assessment if they didn't contact the practice)*					
		b) Received signposts to specialist service *(When you called, did you receive advice about accessing any specialist healthcare professionals or services (e.g. physio, speech and language therapist)? If no/unsure, ask for reasons why)*					
		c) Received signposts to community service *(When you called, did you receive advice about accessing any services in your community (e.g. leisure services/local activity groups, GP/nurse appointment)? If no/unsure, ask*					
	2. Receive support and advice	a) Received advice for stroke-specific issues *(that you raised?)*					
		b) Other brief telephone support *(Was there anything else that you talked about that you received support for when you phoned?)*					
	3. Follow-up appointment(s) arranged for						
	4. Case management *(Finally, was there anything else that was given to you during this call? For example, any further support about how to address your needs, any agreed plans for going forward/addressing your needs?)*						
	TOTAL						
	PERCENTAGE						
END OF CHECKLIST							

For peer review only
For peer review only - http://bmjopen.bmj.com/site/about/guidelines.xhtml

Supplementary file 8

Thank you for attending **Individual appointment 1**.

Please tick (✓) **agree, disagree, don't know** or **does not apply** for each of the statements below.

	Agree	Disagree	Don't know	Does not apply
1. The facilitator explained **'My life after stroke'** to me.				
2. The facilitator invited me to **ask questions** about 'My life after stroke'.				
3. I understand what **'My life after stroke'** is.				
4. I felt I could talk about what life was like **before and after** my stroke.				
5. The facilitator invited me to share any **problems I might face** in attending the course.				
6. I had the chance to talk about **ways to address** the problems I might face in attending the course.				
7. The facilitator invited my carer to come to my appointment.				
8. The facilitator invited my carer to **share experiences** if they wanted to.				

For peer review only

Please turn over.

For peer review only - http://bmjopen.bmj.com/site/about/guidelines.xhtml

Please write your **comments** below:

For peer review only

For peer review only - http://bmjopen.bmj.com/site/about/guidelines.xhtml

Thank you for attending **Stroke Journey**.

Please tick (✓) **agree, disagree, don't know** or **does not apply** for each of the statements below.

	Agree	Disagree	Don't know	Does not apply
1. I felt like I could contribute to the session if I wanted to.				
2. I felt like I could participate in the session activities.				
3. The session included a discussion about my **stroke story**.				
4. I had a chance to think about where I am on my **journey** since my stroke.				
5. I know what a stroke is.				
6. I know how a stroke can affect me.				
7. I understand what **neuroplasticity** is.				
8. I have a **strategy** that can help me deal with the effects of my stroke.				
9. The session included a discussion about **feelings** after having a stroke.				

Please turn over.

For peer review only - http://bmjopen.bmj.com/site/about/guidelines.xhtml

Please tick ✓ **agree**, **disagree**, **don't know** or **does not apply** for each of the statements below.

	Agree	Disagree	Don't know	Does not apply
10. I am aware of the **barriers** to moving forward with my life after stroke.				
11. I had a chance to talk to other stroke survivors **without my carer**.				
12. I had a chance to complete the '**what will I do now?'** worksheet.				
13. I know what I am going to think about doing differently from today.				
14. I received my **'Stroke journey'** handbook.				

Please write your **comments** below:

For peer review only

For peer review only - http://bmjopen.bmj.com/site/about/guidelines.xhtml

Thank you for attending **Managing health and wellbeing**.

Please tick (✓) **agree, disagree, don't know** or **does not apply** for each of the statements below.

	Agree	Disagree	Don't know	Does not apply
1. I felt like I could contribute to the session if I wanted to.				
2. I felt like I could participate in the session activities.				
3. The session included an activity on **risk factors for stroke**.				
4. I am aware of what my risks are for having another stroke.				
5. I know what I could do to help **reduce** my risk of having another stroke.				
6. I know what I could do to **stay healthy**.				
7. I understand the **pros and cons** to making change.				
8. I made an **action plan**.				
9. I am confident I can achieve my plan.				

For peer review only

Please turn over.

For peer review only - http://bmjopen.bmj.com/site/about/guidelines.xhtml

Please tick ✓ **agree**, **disagree**, **don't know** or **does not apply** for each of the statements below.

	Agree	Disagree	Don't know	Does not apply
10. There were opportunities to **ask questions** at the session.				
11. I was able to learn from other people in the group.				
12. I had a chance to complete the **'what will I do now?'** worksheet.				
13. I know what I am going to think about doing differently from today.				
14. I received my **'Managing health and wellbeing'** handbook.				

Please write your **comments** below:

For peer review only - http://bmjopen.bmj.com/site/about/guidelines.xhtml

Thank you for attending **Roadblocks**.

Please tick (✓) **agree, disagree, don't know** or **does not apply** for each of the statements below.

	Agree	Disagree	Don't know	Does not apply
1. I felt like I could contribute to the session if I wanted to.				
2. I felt like I could participate in the session activities.				
3. The group discussed the link between **thoughts, feelings, physical sensations, and behaviour**.				
4. The group discussed what problems or **'roadblocks'** people might face after a stroke.				
5. I had a chance to complete **'what are my roadblocks?'** worksheet.				
6. The session included a **problem-solving** activity.				
7. The facilitators invited me to share my problems and feelings.				
8. I am able to think about my own problems or roadblocks.				
9. I am able to think about how to solve or manage my own problems or roadblocks.				

Please turn over.

For peer review only - http://bmjopen.bmj.com/site/about/guidelines.xhtml
For peer review only

Please tick ✓ **agree**, **disagree**, **don't know** or **does not apply** for each of the statements below.

	Agree	Disagree	Don't know	Does not apply
10. There were opportunities to **ask questions** at the session.				
11. I was able to learn from other people in the group.				
12. I had a chance to complete the **'what will I do now?'** worksheet.				
13. I know what I am going to think about doing differently from today.				
14. I received my **'Roadblocks'** handbook.				

Please write your **comments** below:

For peer review only

For peer review only - http://bmjopen.bmj.com/site/about/guidelines.xhtml

Thank you for attending **‘Moving forward on my journey’**.

Please tick (✓) **agree, disagree, don’t know** or **does not apply** for each of the statements below.

	Agree	Disagree	Don’t know	Does not apply
1. I felt like I could contribute to the session if I wanted to.				
2. I felt like I could participate in the session activities.				
3. I was able to think about how my relationships have changed since having had my stroke.				
4. The facilitators invited me to share my experiences of changing relationships.				
5. The session included a **problem-solving** activity.				
6. I had a chance to talk to other stroke survivors **without my carer**.				
7. I had a chance to think about where I am on my **journey** since my stroke.				
8. I am aware of how I can keep track of my **physical and mental health**.				
9. I know what I could do to **stay healthy**.				

Please turn over.

For peer review only - http://bmjopen.bmj.com/site/about/guidelines.xhtml

For peer review only

Please tick ✓ **agree**, **disagree**, **don't know** or **does not apply** for each of the statements below.

	Agree	Disagree	Don't know	Does not apply
10. There were opportunities to **ask questions** at the session.				
11. The group discussed ways to get **support or information**.				
12. I had a chance to complete the '**what will I do now?'** worksheet.				
13. I know what I am going to think about doing differently from today.				
14. I received my **'Moving forward on my journey'** handbook.				
15. I received my **'Directory'**.				

For peer review only

Please write your **comments** below:

For peer review only - http://bmjopen.bmj.com/site/about/guidelines.xhtml

Thank you for attending **Individual appointment 2**.

Please tick (✓) **agree, disagree, don't know** or **does not apply** for each of the statements below.

	Agree	Disagree	Don't know	Does not apply
1. I was able to reflect on where I am on my **journey** after stroke.				
2. The facilitator invited me to talk about my experience of the **'My life after stroke'** course.				
3. The facilitator invited me to complete the **'Where am I on my journey?'** worksheet.				
4. I was able to **ask questions** if I wanted to.				
5. The facilitator and I talked about my **plans** after 'My life after stroke'.				
6. The facilitator checked if I had any questions.				
7. The appointment included **information about services** that I can access.				

Please turn over.

For peer review only

For peer review only - http://bmjopen.bmj.com/site/about/guidelines.xhtml

Please tick ✓ **agree**, **disagree**, **don't know** or **does not apply** for each of the statements below.

	**Agree**	**Disagree**	**Don't know**	**Does not apply**
8. My carer was invited to come to my appointment.				
9. The facilitator invited my carer to **share experiences** if they wanted to.				

Please write your **comments** below:

For peer review only

For peer review only - http://bmjopen.bmj.com/site/about/guidelines.xhtml

BMJ Open

Protocol for a multidimensional process evaluation of the Improving Primary Care After Stroke (IPCAS) randomised controlled trial

Journal:	*BMJ Open*
Manuscript ID	bmjopen-2020-036879.R2
Article Type:	Protocol
Date Submitted by the Author:	05-May-2020
Complete List of Authors:	Aquino, Maria Raisa Jessica (Ryc); University of Cambridge, Department of Public Health and Primary Care; Newcastle University, Population Health Sciences Institute<br>Mullis, Ricky; University of Cambridge, Department of Public Health and Primary Care<br>Kreit, Elizabeth; University of Cambridge, General Practice & Primary Care Research Centre<br>Johnson, Vicki; Univ Hosp Leicester NHS Trust, Leicester Diabetees Centre<br>Grant, Julie; University of Cambridge, Department of Public Health and Primary Care<br>Lim, Lisa; University of Cambridge, Department of Public Health and Primary Care<br>Sutton, Stephen; University of Cambridge,<br>Mant, Jonathan; University of Cambridge, General Practice and Primary Care Research Unit
**Primary Subject Heading**:	Research methods
Secondary Subject Heading:	Cardiovascular medicine, Health services research, General practice / Family practice
Keywords:	Protocols & guidelines < HEALTH SERVICES ADMINISTRATION & MANAGEMENT, Clinical trials < THERAPEUTICS, PRIMARY CARE, Stroke < NEUROLOGY

SCHOLARONE™
Manuscripts

I, the Submitting Author has the right to grant and does grant on behalf of all authors of the Work (as defined in the below author licence), an exclusive licence and/or a non-exclusive licence for contributions from authors who are: i) UK Crown employees; ii) where BMJ has agreed a CC-BY licence shall apply, and/or iii) in accordance with the terms applicable for US Federal Government officers or employees acting as part of their official duties; on a worldwide, perpetual, irrevocable, royalty-free basis to BMJ Publishing Group Ltd ("BMJ") its licensees and where the relevant Journal is co-owned by BMJ to the co-owners of the Journal, to publish the Work in this journal and any other BMJ products and to exploit all rights, as set out in our licence.

The Submitting Author accepts and understands that any supply made under these terms is made by BMJ to the Submitting Author unless you are acting as an employee on behalf of your employer or a postgraduate student of an affiliated institution which is paying any applicable article publishing charge ("APC") for Open Access articles. Where the Submitting Author wishes to make the Work available on an Open Access basis (and intends to pay the relevant APC), the terms of reuse of such Open Access shall be governed by a Creative Commons licence – details of these licences and which Creative Commons licence will apply to this Work are set out in our licence referred to above.

Other than as permitted in any relevant BMJ Author's Self Archiving Policies, I confirm this Work has not been accepted for publication elsewhere, is not being considered for publication elsewhere and does not duplicate material already published. I confirm all authors consent to publication of this Work and authorise the granting of this licence.

For peer review only

Title

Protocol for a multidimensional process evaluation of the Improving Primary Care After Stroke (IPCAS) randomised controlled trial

Authors

Maria Raisa Jessica (Ryc) Aquino, PhD
Primary Care Unit, Department of Public Health and Primary Care, University of Cambridge, UK
ra532@medschl.cam.ac.uk

Mullis, Ricky, PhD
Primary Care Unit, Department of Public Health and Primary Care, University of Cambridge, UK
rm582@medschl.cam.ac.uk

Kreit, Elizabeth
Primary Care Unit, Department of Public Health and Primary Care, University of Cambridge, UK
ek445@medschl.cam.ac.uk

Johnson, Vicki
Complex Intervention Team, Leicester Diabetes Centre, University Hospitals of Leicester NHS Trust, Leicester, UK
Vicki.Johnson@uhl-tr.nhs.uk

Grant, Julie
Primary Care Unit, Department of Public Health and Primary Care, University of Cambridge, UK
julie.grant@phpc.cam.ac.uk

Lim, Lisa
Primary Care Unit, Department of Public Health and Primary Care, University of Cambridge, UK
lccl2@medschl.cam.ac.uk

Sutton, Stephen, PhD
Primary Care Unit, Department of Public Health and Primary Care, University of Cambridge, UK
srs34@medschl.cam.ac.uk

Mant, Jonathan, MD
Primary Care Unit, Department of Public Health and Primary Care, University of Cambridge, UK
jm677@medschl.cam.ac.uk

On behalf of the IPCAS investigator team: Melanie Davies, Yvonne Doherty, Kamlesh Khunti, Bundy Mackintosh, Adrian Mander, Christopher McKevitt, Martin Roland, Stephen Sutton, Marion Walker, Elizabeth Warburton.

Corresponding Author

Maria Raisa Jessica (Ryc) Aquino
Primary Care Unit, Department of Public Health and Primary Care
University of Cambridge
Worts' Causeway
Cambridge CB1 8RN

For peer review only - http://bmjopen.bmj.com/site/about/guidelines.xhtml

ra532@medschl.cam.ac.uk
+441223 762499

Word count

3404

Keywords

Process evaluation, protocol, intervention fidelity, randomised controlled trial, stroke, primary care

Abstract

Introduction

Primary care interventions are often multicomponent, with several targets (e.g. patients, healthcare professionals). Improving Primary Care After Stroke (IPCAS) is a novel primary care-based model of long-term stroke care involving a review of stroke-related needs, a self-management programme, a direct point of contact in General Practice (GP), enhanced communication between care services, and a directory of national and local community services, currently being evaluated in a cluster randomised controlled trial. Informed by Medical Research Council guidance for complex interventions and Behaviour Change Consortium fidelity framework, this protocol outlines the process evaluation of IPCAS within this RCT. The process evaluation aims to: explore how the intervention was delivered in context, and how participants engaged with the intervention.

Methods and analysis

Mixed-methods will be used. 1) Design: Intervention content will be compared to 'usual care'. 2) Training: Intervention training sessions will be audio/video-recorded where feasible 3) Delivery: Healthcare professional self-reports, audio-recordings of intervention delivery and observations of MLAS course (10% of reviews and sessions) will be coded separately; semi-structured interviews will be conducted with a purposive sample of healthcare professionals. 4) Receipt and 5) Enactment: Where available, structured stroke review records will be analysed quantitatively; semi-structured interviews will be conducted with a purposive sample of study participants. Self-reports, observations and audio/video recordings will be coded and scored using specifically developed checklists. Semi-structured interviews will be analysed thematically. Data will be analysed iteratively, independent of primary endpoint analysis.

Ethics and dissemination

Favourable ethical opinion was gained from Yorkshire & The Humber - Bradford Leeds NHS Research Ethics Committee (19/12/2017; 17/YH/0441). Study results will be published in a peer-reviewed journal and presented at relevant conferences.

Registration details

ClinicalTrials.gov Identifier: NCT03353519

Strengths and limitations of this study

- A strength of this process evaluation protocol includes the use of implementation frameworks, in particular a multidimensional approach to evaluation and intervention fidelity assessment.
- This study applies quantitative and qualitative methods and an iterative approach to analysis to understand how the intervention is implemented in practice.

For peer review only

- This process evaluation will provide insight about uptake and barriers and facilitators to implementing a new model of care in General Practice.
- A limitation of our approach is that interview findings might not be generalisable to other settings.
- We have adapted our interview sampling approach to capture as broad a range of study involvement as possible and aim to triangulate these data with other data sources, such as observations, questionnaires, and routinely collected data.

Introduction

Stroke mortality is in decline, but remains a leading cause of disability (1–3). There are rising numbers of people with stroke living in the community, and with this comes increasing demand on primary care services to address long-term needs such as cognitive, psychological and social problems (3,4). Strategies to address these long-term needs are limited, with stroke survivors and their caregivers often reporting a lack of support (3,5). Stroke prevention is a priority for the National Health Service (NHS) in England (6), as most recently outlined in the *NHS Long Term Plan*, and internationally (6–9). Primary care services have tended to focus on secondary prevention of stroke and risk factor management (10,11) rather than addressing the longer term needs (5), and no formal primary care-based model of care exists to support community-dwelling stroke survivors.

The Improving Primary Care After Stroke (IPCAS) cluster randomised controlled trial (RCT) aims to evaluate the clinical and cost effectiveness of a new model of primary care for stroke survivors living in the community compared with standard care, and has been described previously (12). General practices randomised to the intervention arm will deliver a multicomponent package of care comprising: a one-off structured review with a trained primary care professional using a novel needs checklist (13), a self-management course (My Life After Stroke; MLAS) conducted by trained facilitators over a nine-week period, which aims to improve confidence, independence and hope in survivors of stroke through problem-solving and experience and knowledge exchange with course participants; enhanced communication between healthcare professionals, and a direct point of contact in primary care accessible to patients for stroke-specific support throughout the study period. General practices randomised to the control arm will provide ‘usual care’; the details of which will be documented as part of this process evaluation (see Fidelity of Design). The primary endpoint will be two sub-scales (emotion and handicap) of the Stroke Impact Scale (SIS v3.0) (14) as co-primary outcomes at 12 months after entry into the trial. The intervention and outcomes are summarised in Figure 1.

[INSERT FIGURE 1 HERE]

Complex interventions are widely used to address health problems and consist of multiple interacting components (15,16), often with several targets (e.g. patients, healthcare professionals) and implemented in various settings (17,18). Consequently, variations in implementation could influence intervention effects on outcomes. Process evaluations are especially important for randomised controlled trials such as IPCAS which is being conducted in 46 General Practices in the East of England and the East Midlands. These practices have contextual differences and thus the intervention may undergo adaptations as the study is conducted (18,19). It also has intervention

For peer review only - http://bmjopen.bmj.com/site/about/guidelines.xhtml

components that are aimed at stroke survivors, as well as healthcare professionals. This warrants a multifaceted process evaluation to identify successes, failures and/or unintended consequences (15,20,21). Understanding the delivery of complex interventions is key to determining feasibility of delivering these in other settings and at scale (17). As such, the present evaluation was developed alongside IPCAS and will be conducted in parallel with it.

The UK Medical Research Council (MRC) suggests that process evaluations are conducted to understand how interventions are carried out, how intervention activities produce change, and how the context in which interventions are conducted influences delivery (18,19). In line with this, the US National Institute of Health Behaviour Change Consortium's (BCC) fidelity framework and guidance specifies five dimensions of treatment fidelity: (1) Design: the degree to which the intervention is distinct (e.g. through articulated intervention components and theoretical underpinning) from usual care or other treatment conditions; (2) Training: the extent to which the training of intervention providers is adequate through monitoring training processes and activities; (3) Delivery: the extent to which intervention components and/or procedures are provided as planned; (4) Receipt: the extent to which intervention recipients understand and engage with intervention components; and (5) Enactment: the extent to which intervention recipients carry out specific/relevant behaviours learnt from the intervention in their daily lives (22,23).

This paper outlines the aims, design and methods for an embedded process evaluation within an RCT. This process evaluation is a multidimensional assessment of the IPCAS trial, specifically to explore: 1) how the intervention was delivered; 2) how participants engaged with the intervention; and 3) if/how the context influenced delivery or conduct of the intervention.

Methods and analysis

Design

A mixed-methods sequential design will be applied. The process evaluation (encompassing intervention fidelity) will be conducted in parallel to the IPCAS trial. Table 1 outlines how the five BCC fidelity dimensions will be addressed in IPCAS. Each of these dimensions – design; training; delivery; engagement (enactment and receipt) – are discussed in turn, including relevant materials and data collection processes. These are followed by the patient and public involvement and analysis plans.

Table 1. Assessing intervention fidelity in the *Improving Primary Care After Stroke (IPCAS)* trial

**Fidelity of design**	**Fidelity of training**	**Fidelity of delivery**	**Fidelity of engagement (receipt and enactment)**
Goal:	***Goal:***	***Goal:***	***Goal:***

For peer review only

• To determine the extent to which the intervention reflects its theoretical underpinnings<br>• To determine the extent to which the intervention is distinct from 'usual care'<br>***Strategies:***<br>• Coding intervention components (i.e. IPCAS training manual, MLAS curriculum) to theoretical underpinnings<br>• Comparison of convergence between intervention and control groups (includes recording of participating surgeries' 'usual care' practices)	• To determine the extent to which the training curriculum has been provided as planned<br>***Strategies:***<br>• Training evaluation forms (MLAS)<br>• Video-recorded observations (MLAS)<br>• Audio-recorded observations (IPCAS)<br>• Post-intervention interviews (healthcare professionals, IPCAS)	• To determine the extent to which the intervention was provided as planned<br>***Strategies:***<br>• Audio-recorded observations (IPCAS)<br>• Structured telephone calls to healthcare professionals (IPCAS)<br>• Direct observations (MLAS)<br>• Post-intervention interviews (IPCAS)	• To determine the extent to which stroke survivors understood and applied the skills gained from the intervention<br>***Strategies:***<br>• Self-report questionnaire (MLAS)<br>• Post-review structured telephone calls to participants (IPCAS)<br>• Post-intervention participant interviews

Fidelity of design

This concerns the completeness of the intervention specification, and the degree to which the intervention reflects its theoretical foundations (20,22). All IPCAS intervention components are defined in the trial protocol (12), some of which are standardised (e.g. structured review checklist for use in a one-off, face-to-face annual stroke review), and some not, in order to allow participants and practices flexibility to adapt these components to their needs (e.g. accessing direct point of contact and MLAS, enhanced communication between healthcare professionals). The intervention development process involved a multidisciplinary team (general practitioners (GP), patient

For peer review only

representatives, primary care experts, applied health researchers, psychologists, stroke physicians, practice nurses, therapists), with pilot work informing the feasibility and acceptability of the intervention (12,13).

To ensure that the intervention reflects its theoretical underpinnings and is distinct from usual care, the intervention components (e.g. structured reviews, MLAS etc.) and parameters (e.g., frequency or dose, mode of delivery, duration) will be coded and compared by two independent coders in accordance with established methods (20,22). As such, all participating general practices will be asked to provide details of 'usual care' processes for stroke care and long-term follow-up such as the frequency and content of follow-up appointments, healthcare professionals responsible for these, investigations conducted (e.g. blood tests, blood pressure and pulse measurements) and other actions taken (e.g. referrals or further investigations). Inter-rater reliability will be assessed using Cohen's kappa (95% CI) (24). Where intervention components of similar frequency and/or mode of delivery are found in both conditions, these will be classed as *fully convergent*; where intervention components are found in both experimental conditions but are of dissimilar frequency and/or mode of delivery, these will be classed as *partially convergent;*, and where intervention components are found only in one arm of the study, these will be classed as *unique* (20). Moreover, adaptations to the intervention will be recorded and monitored by the research team throughout the intervention period through maintaining a written record of these in the Fidelity of Delivery assessments (detailed in the following subsections).

Fidelity of training

Participating GP practices will be given a 2-hour face-to-face training session where the research team will present the intervention components and facilitate discussions on how to conduct the intervention using fictional vignettes. A sample of the training sessions (20%; up to 4 sessions) will be audio-recorded, with two raters assessing whether planned components are present using a specifically developed checklist (Supplementary file 1). Training sessions will be randomly audio-recorded (with participant consent) after the fifth training session when trainers have had an opportunity to test their curriculum and materials, and adjustments made where necessary. The percentage of present planned components will be calculated, and inter-rater agreement assessed using Cohen's kappa (95% CI) (24). MLAS facilitators will be trained via a 3-day workshop, with the research team delivering presentations on stroke and its impact, providing trainees time to practise active listening skills, explaining the philosophy and theories underpinning the self-management course, demonstrating MLAS facilitation and providing an opportunity for trainees to rehearse course delivery. MLAS training will be video-recorded and coded by an independent rater according to a priori criteria (Supplementary file 2) to assess whether planned components are present. In addition, trainees will be encouraged to complete training evaluation forms, to assess facilitator knowledge of the MLAS curriculum and expected facilitator behaviours (Supplementary file 3). The percentage of present planned components will be calculated. To ensure skills are maintained, refresher training sessions will be offered as necessary. MLAS facilitators will receive mentoring from researchers as part of their training package after having had an opportunity to deliver a programme. Qualitative interviews with healthcare providers conducting structured reviews will explore their experiences of intervention training (see Fidelity of Delivery). Finally, process variables such as the number of healthcare professionals and facilitators trained, and duration of training sessions will be recorded and summarised.

For peer review only - http://bmjopen.bmj.com/site/about/guidelines.xhtml

Fidelity of delivery

Monitoring intervention delivery is critical to ensuring that the intervention is provided as planned; this will be conducted using quantitative and qualitative methods. The BCC guidance suggests that evaluating intervention sessions by observation (either directly or using audio/video-recording) is the gold standard for monitoring intervention delivery (22,23). To achieve this, a sample of the structured stroke reviews will be audio-recorded and coded by two independent raters according to a pre-specified checklist (Supplementary file 4), which outlines the specific intervention components which healthcare providers are expected to cover. A sample of structured reviews carried out (up to 10% or 46 30-minute reviews across all intervention sites) will be audio-recorded at each practice. With participant consent, some reviews will be recorded after the fifth review has been conducted at each practice, which allows for healthcare professionals to rehearse and become familiarised with review components. Fidelity of delivery will be assessed by calculating the percentage of the pre-specified structured review content that is present. Inter-rater reliability will be assessed using Cohen's Kappa (95% CI) (24). To enable further exploration of delivery, healthcare professionals will be contacted by members of the research team regularly during the intervention period to discuss the structured reviews and to monitor whether the intervention being delivered is as planned, with responses recorded using a pre-specified checklist (see supplementary file 4). These data will be quantified by calculating the percentage of present pre-specified structured review content. Additionally, reasons for (e.g. lack of time) and the nature of (e.g. additional support provided) deviations from the developed intervention will be explored and documented through conducting interviews. A purposively selected sample (approximate n=15; 10 intervention and 5 control) of healthcare professionals will be invited to participate in semi-structured interviews to explore their overall experience of delivering the intervention (intervention) or participation in the study (control) in depth. Specifically, interviews will explore healthcare professionals' reflections on their experience of conducting the structured review, the functionality of the direct point of contact and service mapping tool, and their overall experience of the intervention (e.g. what worked well vs. what did not work well). Interviews will run for approximately 30min-1hr. These interviews are planned to take place from the 6-month follow-up period. Recruitment and data collection will cease when adequate data addressing our evaluation aim have been acquired (25,26) (see Analysis plan).

MLAS courses will also be assessed for fidelity of delivery. Specific contents of this self-management programme are provided in the IPCAS trial protocol (12) and MLAS development paper (in prep). A sample (i.e., two full MLAS courses; approx. 20 hours of observation) of pre-selected sessions of running MLAS programmes will be directly observed independently by two observers, with disagreements resolved through consensus discussion. These observers will have been trained in course delivery and observation. A specially developed observation checklist (Supplementary file 5) will be used to code for the presence or absence of intended course contents, materials and facilitator behaviours. Fidelity will be quantified by assessing the proportion of presence of pre-specified content (i.e. % coded components/planned components). These observations are planned to take place after MLAS facilitators have received mentoring; we also aim to observe all facilitators running MLAS courses at least once. MLAS facilitator-participant interactions will also be observed, using the DESMOND observation tool (Supplementary file 6), in line with similarly designed courses (27).

For peer review only - http://bmjopen.bmj.com/site/about/guidelines.xhtml

Finally, process variables will be collected to assess delivery, and summarised for each participating GP surgery, such as: practice list size, role of healthcare professional conducting reviews, the number of patients attending structured reviews, duration of each structured review conducted, number and content of referrals/signposting to other services, and number of MLAS courses conducted. These fidelity of delivery assessments and process measures will offer an insight into differences in intervention delivery between practices.

Fidelity of receipt and enactment (engagement)

Fidelity of receipt and enactment (engagement), which concerns understanding of intervention content and application of skills gained from the intervention, respectively (22,28) will be assessed concurrently as both encompass participant engagement (28), both quantitatively and qualitatively. Similar to the process outlined for assessing fidelity of delivery, patients will be contacted by members of the research team to discuss the structured reviews they were invited to. Pre-determined questions concerning the structured review will be asked and recorded on a checklist (Supplementary file 7). This will be quantified by calculating the percentage of the present pre-specified structured review content. Concerning MLAS, a purposively selected sample of participants (i.e., 10% of participants attending MLAS, approximately 20) will be invited to complete a specially developed receipt questionnaire (Supplementary file 8) at the end of each session attended (i.e., 6 per participant in total). If necessary, participants will be offered support in completing this.

Qualitative semi-structured interviews with study participants (approximate n= 25; 15 intervention and 10 control), will be conducted to explore their understanding and experience of the new model of care (intervention arm), and their care experiences since involvement in the trial (control arm). Participants will be selected using a random purposive method, given the large pool of eligible participants (e.g. those who attended structured reviews and MLAS, and those who did not), which will allow us to gain representative insights (29). Interviews will run for approximately 30min-1hr. These interviews are expected to take place from the 6-month follow-up period. Specifically, participants will be asked about any action plans they developed with healthcare professionals during their structured review and the outcomes of these, experiences of accessing the direct point of contact for their needs, as well as participation in MLAS. Interviews with control group participants will focus on any stroke-specific appointments/contacts with the GP practice since involvement in the study and any perceived changes in their care during this period. These different areas of focus will allow for an exploration of differences within and between experimental arms. Recruitment and data collection will cease when adequate data addressing our evaluation aim have been acquired (25,26) (see Analysis plan).

In addition, process variables will be collected routinely, such as the number of patients invited and consented, demographic characteristics, time since stroke, number of patients attending structured reviews, number of patients completing the checklist of needs, number of participants enrolled in MLAS (defined as attending the first individual appointment and first group session), and number of participants completing MLAS (defined as attending both individual appointments and at least three of the four group sessions).

Patient and public involvement

Protocol and materials development involved patient and public involvement. A PPI member provided feedback on the process evaluation protocol to ensure procedures (e.g. recruitment, data collection) in place are appropriate and relevant. For example, PPI consultation resulted in changes to the format and length of the questionnaires for MLAS, ensuring that the questionnaire is aphasia

For peer review only - http://bmjopen.bmj.com/site/about/guidelines.xhtml

friendly. Together, we decided to brief facilitators about providing stroke survivors support in completing questionnaires where needed. Our PPI member was also involved in pilot-testing the interview schedule with a member of the research team, which ensured that the interview schedule was suitable and flexible (e.g. addition of closed question prompts for people with aphasia).

Analysis plan

The process evaluation analysis will be undertaken separately to the trial outcome evaluation and will inform the interpretation of these data. Quantitative aspects of the process evaluation (e.g. observational data, checklists/questionnaires) will be assessed using descriptive statistics (i.e. proportions, frequencies and means) and percentage implementation scores on various BCC fidelity dimensions. This will enable us to examine variation in implementation between GP practices at regular intervals. It is suggested that a score of 80-100% is representative of 'high' intervention fidelity, 51-79% is representative of 'moderate' intervention fidelity, and <50% is representative of 'low' intervention fidelity (30). Qualitative aspects of the process evaluation include audio-recorded interview data and textual data (i.e. qualitative data from checklists/questionnaires). All data will be transcribed verbatim. Interview data will be synthesised using thematic analysis by members of the research team using established methods (25). This will also involve an iterative comparison of participant and healthcare professional interview data to understand similarities and differences between intervention providers and recipients. Textual data from checklists and questionnaires will be analysed using content analysis. NVivo 12 will be used to support qualitative data management and analysis.

The quantitative and qualitative analyses will be conducted and integrated at different phases of the evaluation. For example, comparing findings from different data sources such as participants' self-reported receipt and enactment questionnaires and healthcare professionals' self-reported delivery questionnaires with the audio-recorded observations of the structured stroke reviews.

Ethics and dissemination

Favourable ethical opinion for the research was gained on 19th December 2017 from Yorkshire & The Humber - Bradford Leeds NHS Research Ethics Committee. Approval to start was given by the Health Research Authority (HRA) on 21st December 2017, prior to the recruitment of participants commencing at any NHS site. Process evaluation findings will be published in peer-reviewed journals and presented at relevant academic conferences.

Discussion

This multifaceted process evaluation aims to complement the effectiveness testing of a theory-driven complex intervention. It will generate knowledge concerning how and why this novel model of primary care-based stroke care could have an impact, thereby informing ways of scaling up the intervention and implementing the model in other settings as part of usual care. Specifically, the present intervention contains adaptable components as it is implemented pragmatically; therefore, process evaluation findings can offer an insight into how this model of care contributes to the evidence base by illustrating the extent to which a high level of fidelity is useful or necessary.

It has been suggested that there are four possible scenarios as to the contribution of process evaluation findings to investigating how interventions are effective or not (20): 1) successful intervention/high fidelity, where trial outcomes demonstrate improvements in the intervention group and treatment fidelity is deemed 'high', which increases confidence in associating trial

For peer review only - http://bmjopen.bmj.com/site/about/guidelines.xhtml

outcomes with the intervention delivered; 2) successful intervention/low fidelity, where trial outcomes demonstrate improvements in the intervention group and treatment fidelity is deemed ‘low’, which suggests that there may have been confounding factors, or mechanisms of action through which the intervention operated which were not identified and assessed; 3) unsuccessful intervention/high fidelity: where trial outcomes demonstrate no improvements in the intervention group and treatment fidelity is deemed ‘high’, which supports the conclusion that the intervention was not effective, or could have been influenced by confounding factors; and 4) unsuccessful intervention/low fidelity: where trial outcomes demonstrate no improvements in the intervention group and treatment fidelity is deemed ‘low’, which suggests that the intervention was not tested as it was not carried out as planned, or that poor intervention fidelity could have negatively impacted intervention outcomes.

It is recommended that process evaluation protocols are reported and published to encourage transparency (17–20). The protocol described here builds on emerging process evaluation methodology literature which can inform the development and implementation of complex interventions.

Trial status

At the time of submission, the IPCAS trial recruitment is ongoing, with the target sample size of 920 surpassed in March 2019. The IPCAS process evaluation started in March 2018 alongside GP practice recruitment, with the fidelity of design and training data collection and analysis commencing July 2018. Pilot observations and interviews were conducted in February-March 2019, with ongoing iterative analysis.

Author contributions

JM, RM, and SS were responsible for obtaining the funding for this study. The design of the process evaluation was led by RA, with contributions from JM, RM, EK, VJ, LL. JM, RM, EK, VJ, and LL contributed to developing intervention fidelity measures. EK, VJ, LL and JG contributed to piloting intervention fidelity materials, participant recruitment, data collection and analysis. RA wrote the first draft of the manuscript, and all authors contributed to and approved the final version.

Funding

This study is funded by the National Institute for Health Research’s Programme Grant for Applied Research titled ‘Developing primary care services for stroke survivors’ reference PTC-RP-PG-0213-20001. The views expressed are those of the authors and not necessarily those of the NIHR or the Department of Health and Social Care. Jonathan Mant is an NIHR Senior Investigator.

Competing interests

None declared.

For peer review only - http://bmjopen.bmj.com/site/about/guidelines.xhtml

References

1. Feigin VL, Norrving B, Mensah GA. Global burden of stroke. Circ Res. 2017;120(3):439–48.

2. Lee S, Shafe AC, Cowie MR. UK stroke incidence, mortality and cardiovascular risk management 1999–2008: time-trend analysis from the General Practice Research Database. BMJ Open. 2011;1(2):e000269.

3. McKevitt C, Fudge N, Redfern J, Sheldenkar A, Crichton S, Rudd AR, et al. Self-reported long-term needs after stroke. Stroke. 2011;42(5):1398–403.

4. Murray J, Young J, Forster A. Review of longer-term problems after a disabling stroke. Rev Clin Gerontol. 2007;17(4):277–92.

5. Pindus DM, Mullis R, Lim L, Wellwood I, Rundell AV, Aziz NAA, et al. Stroke survivors' and informal caregivers' experiences of primary care and community healthcare services–A systematic review and meta-ethnography. PloS One. 2018;13(2):e0192533.

6. NHS England. The NHS Long Term Plan [Internet]. 2019 [cited 2020 Apr 16]. Available from: https://www.longtermplan.nhs.uk

7. US Department of Health and Human Services. Healthy people 2020 [Internet]. Office of Disease Prevention and Health Promotion; 2010 [cited 2019 Dec 17]. Available from: https://www.healthypeople.gov/sites/default/files/HP2020_brochure_with_LHI_508_FNL.pdf

8. The Ministry of Health and Care Services (Norway). Care plan 2020: The Norwegian Government's plan for the care services field for 2015-2020 [Internet]. The Ministry of Health and Care Services (Norway); 2015 [cited 2019 Dec 17]. Available from: https://www.regjeringen.no/contentassets/af2a24858c8340edaf78a77e2fbe9cb7/careplan2020_eng.pdf

9. Centers for Disease Control and Prevention. A study of primary stroke center policy: Recommendations for policy implementation [Internet]. US Department of Health and Human Services; 2012 [cited 2019 Dec 17]. Available from: https://www.cdc.gov/dhdsp/docs/Primary_Stroke_Center_Policies_Recommendations.pdf

10. Norrving B, Davis SM, Feigin VL, Mensah GA, Sacco RL, Varghese C. Stroke prevention worldwide-what could make it work. Neuroepidemiology. 2015;45(3):215–20.

11. Joubert J, Reid C, Joubert L, Barton D, Ruth D, Jackson D, et al. Risk factor management and depression post-stroke: the value of an integrated model of care. J Clin Neurosci. 2006;13(1):84–90.

12. Mullis R, Aquino MRJ, Dawson S, Johnson V, Jowett S, Kreit E, et al. The IPCAS Trial (Improving Primary Care After Stroke): protocol of a randomised controlled trial to evaluate a novel model of care for stroke survivors living in the community. BMJ Open. 2019;9:e030285.

13. Turner GM, Mullis R, Lim L, Kreit L, Mant J. Using a checklist to facilitate management of long-term care needs after stroke: insights from focus groups and a feasibility study. BMC Fam Pract. 2019;20(1):2.

14. Jenkinson C, Fitzpatrick R, Crocker H, Peters M. The Stroke Impact Scale: validation in a UK setting and development of a SIS short form and SIS index. Stroke. 2013;44(9):2532–5.

For peer review only

15. Craig P, Dieppe P, Macintyre S, Michie S, Nazareth I, Petticrew M, et al. Developing and evaluating complex interventions: the new Medical Research Council guidance. BMJ. 2008;337:a1655–a1655.

16. Campbell M, Fitzpatrick R, Haines A, Kinmonth AL, Sandercock P, Spiegelhalter D, et al. Framework for design and evaluation of complex interventions to improve health. Bmj. 2000;321(7262):694–6.

17. Liu H, Lindley R, Alim M, Felix C, Gandhi DB, Verma SJ, et al. Protocol for process evaluation of a randomised controlled trial of family-led rehabilitation post stroke (ATTEND) in India. BMJ Open. 2016;6(9):e012027.

18. Moore GF, Audrey S, Barker M, Bond L, Bonell C, Hardeman W, et al. Process evaluation of complex interventions: Medical Research Council guidance. bmj. 2015;350:h1258.

19. Campbell-Scherer D, Saitz R. Improving reporting and utility of evaluations of complex interventions. BMJ Evid-Based Med. 2016;21:1–3.

20. Lorencatto F, Gould NJ, McIntyre SA, During C, Bird J, Walwyn R, et al. A multidimensional approach to assessing intervention fidelity in a process evaluation of audit and feedback interventions to reduce unnecessary blood transfusions: a study protocol. Implement Sci. 2016;11(1):163.

21. Oakley A, Strange V, Bonell C, Allen E, Stephenson J. Process evaluation in randomised controlled trials of complex interventions. Bmj. 2006;332(7538):413–6.

22. Borrelli B, Sepinwall D, Ernst D, Bellg AJ, Czajkowski S, Breger R, et al. A new tool to assess treatment fidelity and evaluation of treatment fidelity across 10 years of health behavior research. J Consult Clin Psychol. 2005;73(5):852.

23. Bellg AJ, Borrelli B, Resnick B, Hecht J, Minicucci DS, Ory M, et al. Enhancing Treatment Fidelity in Health Behavior Change Studies: Best Practices and Recommendations From the NIH Behavior Change Consortium. Health Psychol. 2004;23(5):443–51.

24. Walton H, Spector A, Williamson M, Tombor I, Michie S. Developing quality fidelity and engagement measures for complex health interventions. Br J Health Psychol. 2019;

25. Braun V, Clarke V. Thematic analysis. In: Cooper H, Camic PM, Long DL, Panter A, Rindskopf D, Sher KJ, editors. APA handbook of resaerch methods in psychology, Vol 2 Resaerch designs: Quantitative, qualitative, neuropsychological, amd biological. American Psychological Association; 2012.

26. Braun V, Clarke V. To saturate or not to saturate? Questioning data saturation as a useful concept for thematic analysis and sample-size rationales. Qual Res Sport Exerc Health. 2019;1–16.

27. Skinner T, Carey M, Cradock S, Dallosso H, Daly H, Davies MJ, et al. 'Educator talk'and patient change: some insights from the DESMOND (Diabetes Education and Self Management for Ongoing and Newly Diagnosed) randomized controlled trial. Diabet Med. 2008;25(9):1117–20.

28. Walton H, Spector A, Tombor I, Michie S. Measures of fidelity of delivery of, and engagement with, complex, face-to-face health behaviour change interventions: A systematic review of measure quality. Br J Health Psychol. 2017;22(4):872–903.

29. Patton M. Qualitative research and evaluation methods. 4th ed. Saint Paul, MN: SAGE; 2015.

For peer review only

30. Noell GH, Gresham FM, Gansle KA. Does treatment integrity matter? A preliminary investigation of instructional implementation and mathematics performance. J Behav Educ. 2002;11(1):51–67.

For peer review only

Figure 1. Intervention and outcomes

Contextual factors within intervention environment: 'Usual care' at each practice, socioeconomic factors, stroke-related factors (e.g., time since stroke, comorbidities)

Improving Primary Care After Stroke (IPCAS): a primary care-based model of care to support community-dwelling stroke survivors	Primary outcomes	Secondary outcomes	Long-term outcomes
<u>Participant-focused components</u><br>15-item checklist of common post-stroke needs sent to participant by post. Followed by:<br><br>Structured review appointment: Up to 30-min with a trained member of the practice team (e.g. nurse, GP) to discuss up to 3 needs prioritised by the patient from the checklist. The outcomes are an action plan agreed with the patient on how to address the key needs, information on the Direct Point of Contact (DPOC) service, and on the My Life After Stroke (MLAS) course.<br><br>My Life After Stroke (MLAS), an optional 6-week, group-based self-management course delivered by two trained facilitators in a local community venue. The first and final appointments are individual appointments lasting up to 45 minutes, in between which are four-weekly group sessions focused on learning about the consequences of a stroke, and managing one's health and wellbeing using goal setting and problem-solving strategies. In the final group session, participants will also be given an abbreviated print version of the service mapping tool (see below).<br><br><u>Healthcare professional-focused components:</u><br>Training on new model of primary care: One-off, face-to-face session with trial team lasting up to 2 hours.<br><br>DPOC: Trained practice staff will provide stroke-specific advice, information and/or support to study participants by telephone.<br><br>Optimised communication between healthcare professionals: A meeting will be arranged between primary care and specialist staff (hospital and community) to facilitate interprofessional communication. All practice staff involved with providing care to stroke survivors will be encouraged to attend any meetings/training sessions organised by specialists, and provided specialists' contact details. Video recordings of local specialist providers describing their service, commonly referred patients to the service, and means of contact/referral will be provided to practice staff.<br><br>Service mapping tool: This is a bespoke directory of national and local services provided to practices in Excel and print formats. These can be used at the structured review, or when providing support via the DPOC service.	<u>Baseline, 6, and 12 mos.:</u><br>Emotion and handicap sub-scales of Stroke Impact Scale v3.0	<u>6 and 12 mos.:</u><br>EQ-5D-5L, ICEpop CAPability measure for Adults<br><br><u>12 mos. only:</u><br>Southampton Stroke Self-management Questionnaire, Health Literacy Questionnaire	Cost-utility as determined in within-trial economic evaluation

For peer review only - http://bmjopen.bmj.com/site/about/guidelines.xhtml

Supplementary file 1

IPCAS trial provider process evaluation/fidelity checklist – training phase							
**Site ID:**		**Duration of training:**			**Training date (Coding date):**		
**Activity**			**Tick as appropriate**			**If *present to some extent*, provide reason(s) for this rating**	**Other comments**
			**Present**	**Absent**	**Partially done**		
**Structured review**	1. 15-item checklist of needs	a) Discussion of purpose and structure of checklist					
		b) Instructions on how to use checklist					
	2. Physical check	a) Discussion of carrying out optional physical checks at review (in line with QOF)					
	3. Development of action plan(s)	a) Discussion of action plan					
		b) Instructions given: how to log and review agreed actions (using template)					
	4. MLAS	a) Discussion of purpose and structure of MLAS					
		b) Provided instructions for how patients can access MLAS					
	5. Direct point of contact to stroke survivor and carer(s)	a) Given instructions for offering 'direct point of contact' service					
		b) Discussion of how to provide instructions to patients about contacting practice at the end of review					
**Use of direct point of contact**	1. Signposting to services	a) Discussed service mapping: what it is					
		b) Discussed how to use service mapping					

For peer review only - http://bmjopen.bmj.com/site/about/guidelines.xhtml

IPCAS trial provider process evaluation/fidelity checklist – training phase							
**Site ID:**		**Duration of training:**			**Training date (Coding date):**		
**Activity**			**Tick as appropriate**			**If *present to some extent*, provide reason(s) for this rating**	**Other comments**
			**Present**	**Absent**	**Partially done**		
	2. Provide support and advice	a) Gave information on structure and scope of direct point of contact service: giving advice for stroke-specific issues					
		b) Gave information on structure and scope of direct point of contact service: arranging follow-up appointment(s)					
		c) Gave information on structure and scope of direct point of contact service: brief telephone support/case management					
**Enhanced communication pathways**	1. Discussion on communication pathways	a) Discussed broad structure of enhanced communication pathways					
		b) Initial discussion about dates for face-to-face meeting					
**END OF CHECKLIST**							

For peer review only

For peer review only - http://bmjopen.bmj.com/site/about/guidelines.xhtml

IPCAS trial process evaluation/fidelity checklist
MLAS facilitator training

**Date:**					
**Start time:**					
**End time:**					
**Training day 1**					
	**Please tick as appropriate**			**If *absent, attempted* or *not applicable*, please provide reasons**	**Other comments**
	**Present**	**Absent**	**Attempted**		
**Session 1: Welcome and introductions**					
**Duration of session:**					
**Materials**					
Timetable available	☐	☐	☐		
Headed flipchart paper – working together	☐	☐	☐		
Pre-training 'evaluation' form given (1 per participant)	☐	☐	☐		
**Content**					
Welcome to MLAS	☐	☐	☐		
Introduction of trainers	☐	☐	☐		

For peer review only - http://bmjopen.bmj.com/site/about/guidelines.xhtml

**Date:**					
**Start time:**					
**End time:**					
**Training day 1**					
	**Please tick as appropriate**			**If *absent, attempted* or *not applicable*, please provide reasons**	**Other comments**
	**Present**	**Absent**	**Attempted**		
Housekeeping details provided	☐	☐	☐		
Aim of training discussed	☐	☐	☐		
Training overview discussed	☐	☐	☐		
Outline of Day 1 discussed	☐	☐	☐		
Working together discussed	☐	☐	☐		
Provided time for participants to complete pre-training 'evaluation' form	☐	☐	☐		

For peer review only - http://bmjopen.bmj.com/site/about/guidelines.xhtml

**Session 2: Your story**					
**Duration of session:**					
**Materials**					
Headed flipchart paper (1: Name, role, place of work, 2: Experience, 3: Expectation of training, 4: Your important question)	☐	☐	☐		
**Content**					
Asked: Name, role, place of work	☐	☐	☐		
Asked: Experience (facilitating/observing groups, working with stroke)	☐	☐	☐		
Asked: Expectation(s)	☐	☐	☐		
Asked: Any question in relation to MLAS	☐	☐	☐		
Provided opportunity for each trainee to answer the above questions and record on relevant flipchart	☐	☐	☐		
**Session 3: What is stroke?**					
**Duration of session:**					
**Materials**					
Body outline used	☐	☐	☐		
Images shown: clot, bleed, brain areas	☐	☐	☐		
Images shown: risk factors, neuroplasticity	☐	☐	☐		

For peer review only - http://bmjopen.bmj.com/site/about/guidelines.xhtml

Videos shown: Act FAST, neuroplasticity	☐	☐	☐		
Images shown: health and well-being	☐	☐	☐		
Sticky notes used	☐	☐	☐		
**Content**					
Discussed 'what is stroke?'	☐	☐	☐		
Discussed signs and symptoms – ACTFAST	☐	☐	☐		
Discussed impacts of a stroke (including physical, emotional, cognitive, communication, relationships)	☐	☐	☐		
Discussed what neuroplasticity is	☐	☐	☐		
Discussed stroke risk factors	☐	☐	☐		
Discussed managing risk factors	☐	☐	☐		
Participants given an opportunity to contribute to discussion and activities	☐	☐	☐		
**Session 4: Cognitive effects of stroke**					
**Duration of session:**					
**Materials**					
PowerPoint slides used: cognitive effects of stroke	☐	☐	☐		
Video shown: Hidden effects of stroke	☐	☐	☐		

For peer review only - http://bmjopen.bmj.com/site/about/guidelines.xhtml

Booklet given: Complete guide to Cognitive problems (1 copy/facilitator)	☐	☐	☐		
**Content**					
Session introduced	☐	☐	☐		
Discussed memory problems after a stroke	☐	☐	☐		
Explored attention difficulties	☐	☐	☐		
Explored changes to perception	☐	☐	☐		
Explored effects on decision-making	☐	☐	☐		
Explored social cognition problems	☐	☐	☐		
Discussed how we can support stroke survivors experiencing these effects/top tips	☐	☐	☐		
Discussed the impact of impaired cognition on the stroke survivor and/or carer/family	☐	☐	☐		
Participants given an opportunity to contribute to discussion and activities	☐	☐	☐		
**Session 5: Emotional changes after stroke**					
**Duration of session:**					
**Materials**					
Video shown: The emotional impact of stroke	☐	☐	☐		
PowerPoint slides: Emotional problems following stroke					

For peer review only

For peer review only - http://bmjopen.bmj.com/site/about/guidelines.xhtml

Booklet given: Complete guide to Emotional changes after stroke (1 copy/facilitator)	☐	☐	☐		
**Content**					
Session introduced	☐	☐	☐		
Discussed the emotions stroke survivors highlighted in video observed	☐	☐	☐		
Discussed stages of loss	☐	☐	☐		
Discussed problems stroke survivors might face with emotions	☐	☐	☐		
Discussed difficulty controlling emotions	☐	☐	☐		
Discussed how these emotions can influence people's thoughts and behaviours	☐	☐	☐		
Discussed how facilitators can support stroke survivors experiencing these effects/top tips	☐	☐	☐		
Participants given an opportunity to contribute to discussion and activities	☐	☐	☐		
**Session 6: Relationships**					
**Duration of session:**					
**Materials**					
PowerPoint slides used: Relationships	☐	☐	☐		
**Content**					

For peer review only

For peer review only - http://bmjopen.bmj.com/site/about/guidelines.xhtml

Session introduced	☐	☐	☐		
Discussed the effect of a stroke on relationships (partners, friends, family members)	☐	☐	☐		
Discussed problems and solutions concerning physical intimacy after a stroke	☐	☐	☐		
Participants given an opportunity to contribute to discussion and activities	☐	☐	☐		
**Session 7: Communication challenges for survivors of stroke**					
**Duration of session:**					
**Materials**					
PowerPoint slides used: Communication	☐	☐	☐		
Video used: Living with aphasia; aphasia etiquette	☐	☐	☐		
Booklet given: Complete guide to Communication problems after stroke (1 copy/facilitator)	☐	☐	☐		
**Content**					
Session introduced	☐	☐	☐		
Group activity: Identifying tasks the brain needs to complete in order to be able to answer a question	☐	☐	☐		

For peer review only - http://bmjopen.bmj.com/site/about/guidelines.xhtml

Discussed communication problems after a stroke	☐	☐	☐		
Discussed how we can support stroke survivors experiencing communication problems (top tips, resources/aids)	☐	☐	☐		
Participants given an opportunity to contribute to discussion and activities	☐	☐	☐		
**Session 8: Summary and evaluation**					
**Duration of session:**					
**Materials**					
Training evaluation form given	☐	☐	☐		
Questions relating to MLAS flipchart	☐	☐	☐		
**Content**					
Summary of Day 1 Training	☐	☐	☐		
Provided time for participants to complete training evaluation form	☐	☐	☐		
Provided details of Day 2 Training	☐	☐	☐		
Participants given an opportunity to check if their questions addressed, and to ask any further questions	☐	☐	☐		
**END OF DAY 1 TRAINING**					

For peer review only - http://bmjopen.bmj.com/site/about/guidelines.xhtml

IPCAS trial process evaluation/fidelity checklist
MLAS facilitator training

**Date:**					
**Start time:**					
**End time:**					
**Training day 2**					
	**Please tick as appropriate**			**If *absent, attempted* or *not applicable*, please provide reasons**	**Other comments**
	**Present**	**Absent**	**Attempted**		
**Session 1: Welcome and introductions**					
**Duration of session:**					
**Materials**					
Timetable available	☐	☐	☐		
Headed flipchart paper from day 1 (‘Your story’ - name, experience, expectation, important question and working together as a group)	☐	☐	☐		
**Content**					
Welcome	☐	☐	☐		
Introduction of trainers	☐	☐	☐		

For peer review only - http://bmjopen.bmj.com/site/about/guidelines.xhtml

**Date:**					
**Start time:**					
**End time:**					
**Training day 2**					
	**Please tick as appropriate**			**If *absent, attempted* or *not applicable*, please provide reasons**	**Other comments**
	**Present**	**Absent**	**Attempted**		
Housekeeping details provided	☐	☐	☐		
Overall aim/format of training discussed	☐	☐	☐		
Outline of Day 2 discussed	☐	☐	☐		
Invited any new participants to introduce themselves to group using 'Your story' flipchart from Day 1	☐	☐	☐		
**Session 2: Background to MLAS**					
**Duration of session:**					
**Materials**					
Flipchart paper, pens	☐	☐	☐		
A4 Quadrant image	☐	☐	☐		
**Content**					
Discussed what MLAS is	☐	☐	☐		

For peer review only

**Date:**					
**Start time:**					
**End time:**					
**Training day 2**					
	**Please tick as appropriate**			**If *absent, attempted* or *not applicable*, please provide reasons**	**Other comments**
	**Present**	**Absent**	**Attempted**		
Discussed what a structured education programme is	☐	☐	☐		
Discussed rationale for MLAS	☐	☐	☐		
Discussed aims and format of MLAS course	☐	☐	☐		
Participants given an opportunity to be involved in discussion and ask questions	☐	☐	☐		
**Session 3: Philosophy**					
**Duration of session:**					
**Materials**					
How are you doing? Worksheet (1 per participant)	☐	☐	☐		
PowerPoint slides used: Philosophy	☐	☐	☐		
**Content**					

For peer review only - http://bmjopen.bmj.com/site/about/guidelines.xhtml

**Date:**					
**Start time:**					
**End time:**					
**Training day 2**					
	**Please tick as appropriate**			**If *absent, attempted* or *not applicable*, please provide reasons**	**Other comments**
	**Present**	**Absent**	**Attempted**		
Discussed what philosophy is and how it affects our behaviours	☐	☐	☐		
Explored the medical model and empowerment approach	☐	☐	☐		
Explored the philosophy and approach of the MLAS programme	☐	☐	☐		
Facilitated individual activity ('How are you doing' quiz) and gave an opportunity for each trainee to complete their own worksheet	☐	☐	☐		
Discussed the need to have importance, confidence and readiness in order to change behaviour (and not just knowledge)	☐	☐	☐		
Presented overall aim of MLAS and its core philosophy statements	☐	☐	☐		
Discussed MLAS facilitators' role					

For peer review only

For peer review only - http://bmjopen.bmj.com/site/about/guidelines.xhtml

**Date:**					
**Start time:**					
**End time:**					
**Training day 2**					
	**Please tick as appropriate**			**If *absent, attempted* or *not applicable*, please provide reasons**	**Other comments**
	**Present**	**Absent**	**Attempted**		
Group activity: Challenges experienced/perceived by facilitators when using the philosophy/approach underpinning MLAS	☐	☐	☐		
Participants given an opportunity to be involved in discussion (e.g. by giving feedback) and ask questions	☐	☐	☐		
**Session 4: Facilitator behaviours**					
**Duration of session:**					
**Materials**					
Rice pudding exercise	☐	☐	☐		
MLAS facilitator behaviours checklist given (1 per participant)	☐	☐	☐		
**Content**					

For peer review only - http://bmjopen.bmj.com/site/about/guidelines.xhtml

**Date:**					
**Start time:**					
**End time:**					
**Training day 2**					
	**Please tick as appropriate**			**If *absent, attempted* or *not applicable*, please provide reasons**	**Other comments**
	**Present**	**Absent**	**Attempted**		
Session introduced	☐	☐	☐		
Rice pudding activity (demonstrating different facilitator behaviours – 'good' and 'bad')	☐	☐	☐		
Discussed positive and negative facilitator behaviours that were observed	☐	☐	☐		
Discussed some of the behaviours on the behaviours checklist, and provided examples of what would be observed if such behaviours were being done (e.g. body language, words used etc.)	☐	☐	☐		
Encouraged participants to engage in discussion about any stroke-specific behaviours needed	☐	☐	☐		
Participants given an opportunity to be involved in discussion (e.g. by giving feedback) and ask questions	☐	☐	☐		

For peer review only

For peer review only - http://bmjopen.bmj.com/site/about/guidelines.xhtml

**Date:**					
**Start time:**					
**End time:**					
**Training day 2**					
	**Please tick as appropriate**			**If *absent, attempted* or *not applicable*, please provide reasons**	**Other comments**
	**Present**	**Absent**	**Attempted**		
**Session 5: Facilitation skills**					
**Duration of session:**					
**Materials**					
PowerPoint slides used: Facilitation skills	☐	☐	☐		
Flipchart paper and pens					
Open-ended Questions, Affirmations, Reflective listening, Summaries (OARS) sheets given (1 per participant)	☐	☐	☐		
**Content**					
Session introduced	☐	☐	☐		
Facilitated group activity on facilitator role and responsibilities	☐	☐	☐		
OARS introduced to participants	☐	☐	☐		

For peer review only - http://bmjopen.bmj.com/site/about/guidelines.xhtml

**Date:**					
**Start time:**					
**End time:**					
**Training day 2**					
	**Please tick as appropriate**			**If *absent, attempted* or *not applicable*, please provide reasons**	**Other comments**
	**Present**	**Absent**	**Attempted**		
Group activity: Challenging scenarios and resolutions	☐	☐	☐		
Encouraged participants to share personal learning	☐	☐	☐		
Participants given an opportunity to be involved in discussion (e.g. by giving feedback) and ask questions	☐	☐	☐		
**Session 6: Theories – Narrative Approach; Self-efficacy**					
**Duration of session:**					
**Materials**					
PowerPoint slides used: Theories	☐	☐	☐		
Sticky notes, flipchart paper, pens	☐	☐	☐		
MLAS Roadmap and accessories	☐	☐	☐		
**Content**					

For peer review only - http://bmjopen.bmj.com/site/about/guidelines.xhtml

**Date:**					
**Start time:**					
**End time:**					
**Training day 2**					
	**Please tick as appropriate**			**If *absent, attempted* or *not applicable*, please provide reasons**	**Other comments**
	**Present**	**Absent**	**Attempted**		
Session introduced	☐	☐	☐		
Introduced the Narrative Approach	☐	☐	☐		
Demonstrated how the narrative approach is used in MLAS (journey analogy)	☐	☐	☐		
Introduced Self-efficacy (Mastery, vicarious learning, verbal persuasion and emotions management)	☐	☐	☐		
Group activity: Real-world experiences of self-efficacy	☐	☐	☐		
Examples provided where self-efficacy is used during MLAS	☐	☐	☐		
Introduced cognitive behavioural approach and gave an example of how it is used in MLAS	☐	☐	☐		

For peer review only - http://bmjopen.bmj.com/site/about/guidelines.xhtml

**Date:**					
**Start time:**					
**End time:**					
**Training day 2**					
	**Please tick as appropriate**			**If *absent, attempted* or *not applicable*, please provide reasons**	**Other comments**
	**Present**	**Absent**	**Attempted**		
Participants given an opportunity to be involved in discussion (e.g. by giving feedback) and ask questions	☐	☐	☐		
**Session 7: Introduction to Quality Assurance**					
**Duration of session:**					
**Materials**					
Flipchart paper and pens	☐	☐	☐		
**Content**					
Session introduced	☐	☐	☐		
Discussed what quality assurance is and its important	☐	☐	☐		
Facilitated small group activity (recipe and ingredients for making an Apple Pie)	☐	☐	☐		

For peer review only - http://bmjopen.bmj.com/site/about/guidelines.xhtml

**Date:**					
**Start time:**					
**End time:**					
**Training day 2**					
	**Please tick as appropriate**			**If *absent, attempted* or *not applicable*, please provide reasons**	**Other comments**
	**Present**	**Absent**	**Attempted**		
Encouraged each group to feedback on group activity.	☐	☐	☐		
Compared similarities and differences of Apple Pie recipes and linked to quality assurance of MLAS (ingredients = curriculum, method = delivery)	☐	☐	☐		
Summarised group discussion on quality assurance processes	☐	☐	☐		
Participants given an opportunity to be involved in discussion (e.g. by giving feedback) and ask questions	☐	☐	☐		
**Session 8: A journey through the MLAS curriculum**					
**Duration of session:**					
**Materials**					

For peer review only - http://bmjopen.bmj.com/site/about/guidelines.xhtml

**Date:**					
**Start time:**					
**End time:**					
**Training day 2**					
	**Please tick as appropriate**			**If *absent, attempted* or *not applicable*, please provide reasons**	**Other comments**
	**Present**	**Absent**	**Attempted**		
MLAS curriculum given to each facilitator	☐	☐	☐		
Participant (stroke survivor) handbooks given to each facilitator (4 handbooks in total)	☐	☐	☐		
A journey through the curriculum question sheet given to each facilitator	☐	☐	☐		
**Content**					
Provided an overview of materials given	☐	☐	☐		
Explained the purpose of 'a journey through the curriculum question sheet' and instructions for completion	☐	☐	☐		
**Session 9: Close of the day reflections and evaluation**					
**Duration of session:**					
**Materials**					
Training evaluation form (day 2) given	☐	☐	☐		

For peer review only - http://bmjopen.bmj.com/site/about/guidelines.xhtml

**Date:**					
**Start time:**					
**End time:**					
**Training day 2**					
	**Please tick as appropriate**			**If *absent, attempted* or *not applicable*, please provide reasons**	**Other comments**
	**Present**	**Absent**	**Attempted**		
Questions relating to MLAS flipchart	☐	☐	☐		
**Content**					
Summary of Day 2 training by encouraging participants to share learning about what was covered	☐	☐	☐		
Provided time for participants to complete training evaluation form	☐	☐	☐		
Provided details of Day 3 Training	☐	☐	☐		
Participants given an opportunity to check if their question (from flipchart) was addressed, and to ask any further questions	☐	☐	☐		
**END OF DAY 2 TRAINING**					

For peer review only

IPCAS trial process evaluation/fidelity checklist

MLAS facilitator training

**Date:**					
**Start time:**					
**End time:**					
**Training day 3**					
	**Please tick as appropriate**			**If *absent* or *attempted* please provide reasons**	**Other comments**
	**Present**	**Absent**	**Attempted**		
**Session 1: Welcome and introductions**					
**Duration of session:**					
**Materials**					
Timetable available	☐	☐	☐		
Headed flipchart paper from days 1+2 ('Your story' - name, experience, expectation, important question and working together as a group)	☐	☐	☐		
**Content**					
Welcome	☐	☐	☐		

For peer review only - http://bmjopen.bmj.com/site/about/guidelines.xhtml

**Date:**					
**Start time:**					
**End time:**					
**Training day 3**					
	**Please tick as appropriate**			**If *absent* or *attempted* please provide reasons**	**Other comments**
	**Present**	**Absent**	**Attempted**		
Re-introduction of trainers	☐	☐	☐		
Housekeeping details provided	☐	☐	☐		
Aim of training discussed	☐	☐	☐		
Outline of Day 3 discussed	☐	☐	☐		
Invited any new participants to introduce themselves to group using 'Your story' flipchart from Day 1+2	☐	☐	☐		
**Session 2: Reflection from 'A journey through the curriculum' activity**					
**Duration of session:**					
**Materials**					
A journey through the curriculum activity sheet	☐	☐	☐		
A journey through the curriculum activity answer sheet given	☐	☐	☐		
MLAS curriculum	☐	☐	☐		
**Content**					

For peer review

For peer review only - http://bmjopen.bmj.com/site/about/guidelines.xhtml

**Date:**					
**Start time:**					
**End time:**					
**Training day 3**					
	**Please tick as appropriate**			**If *absent* or *attempted* please provide reasons**	**Other comments**
	**Present**	**Absent**	**Attempted**		
Invited participants to share experiences of completing 'a journey through the curriculum' activity	☐	☐	☐		
Discussed format of the programme (1:1 and group-based sessions; interchangeable sessions)	☐	☐	☐		
Discussed format of the curriculum (e.g. key for how to use the curriculum, duration for each section, section overviews)	☐	☐	☐		
Discussed involvement of carers in MLAS and format of this	☐	☐	☐		
Participants given an opportunity to be involved in discussion and ask questions	☐	☐	☐		
**Session 3: A walk through the MLAS curriculum**					
**Duration of session:**					
**Materials**					

For peer review only - http://bmjopen.bmj.com/site/about/guidelines.xhtml

**Date:**					
**Start time:**					
**End time:**					
**Training day 3**					
	**Please tick as appropriate**			**If *absent* or *attempted* please provide reasons**	**Other comments**
	**Present**	**Absent**	**Attempted**		
MLAS curriculum	☐	☐	☐		
Participant (stroke survivor) handbooks	☐	☐	☐		
Resources/accessories for programme available	☐	☐	☐		
**Content**					
Discussed the overall aims of the study and the intervention	☐	☐	☐		
Presented each of the MLAS sessions with the resources	☐	☐	☐		
Demonstrated how to use the resources	☐	☐	☐		
Demonstrated links between the sessions and materials to the theories and philosophy applied	☐	☐	☐		
Discussed challenges to delivery and strategies for addressing these	☐	☐	☐		
Discussed tailoring of sessions	☐	☐	☐		

For peer review only - http://bmjopen.bmj.com/site/about/guidelines.xhtml

**Date:**					
**Start time:**					
**End time:**					
**Training day 3**					
	**Please tick as appropriate**			**If *absent* or *attempted* please provide reasons**	**Other comments**
	**Present**	**Absent**	**Attempted**		
Discussed some examples of what previous facilitators have observed/dealt with during previous courses	☐	☐	☐		
Participants given an opportunity to be involved in discussion and ask questions	☐	☐	☐		
**Session 4: Preparing for having a go**					
**Duration of session:**					
**Materials**					
Facilitator curricula	☐	☐	☐		
'Have a go' cards (1 per facilitator)	☐	☐	☐		
Roadmap and accessories available	☐	☐	☐		
A4 images: blood clot, bleed available	☐	☐	☐		
A6 images: risk factors, risks I can/cannot change available	☐	☐	☐		

For peer review only

For peer review only - http://bmjopen.bmj.com/site/about/guidelines.xhtml

**Date:**					
**Start time:**					
**End time:**					
**Training day 3**					
	**Please tick as appropriate**			**If *absent* or *attempted* please provide reasons**	**Other comments**
	**Present**	**Absent**	**Attempted**		
A4 and A6 images: health and wellbeing available	☐	☐	☐		
A4 images: dashboard available	☐	☐	☐		
Writing instruments available (sticky notes, flipchart paper, pens, Blu Tack)	☐	☐	☐		
**Content**					
Session introduced	☐	☐	☐		
Participants divided into 2 groups	☐	☐	☐		
Explained that each participant has been given details of a section of the MLAS course (to be delivered in the next session of training)	☐	☐	☐		
Given instructions for next steps (i.e. time to prepare for delivery of chosen 10-minute section, how 'have a go' session will run)	☐	☐	☐		

For peer review only - http://bmjopen.bmj.com/site/about/guidelines.xhtml

For peer review only

**Date:**					
**Start time:**					
**End time:**					
**Training day 3**					
	**Please tick as appropriate**			**If *absent* or *attempted* please provide reasons**	**Other comments**
	**Present**	**Absent**	**Attempted**		
Offered participants support for preparing for their sessions (e.g. providing relevant resources, explaining content)	☐	☐	☐		
Provided opportunity/time for facilitators to prepare for 10-minute session	☐	☐	☐		
Participants given an opportunity to be involved in discussion and ask questions	☐	☐	☐		
**Session 5: Have a go**					
**Duration of session:**					
**Materials**					
Facilitator curricula	☐	☐	☐		
‘Have a go’ cards (x2 sets) given out to participants	☐	☐	☐		
Roadmap and accessories displayed	☐	☐	☐		
A4 images: blood clot, bleed displayed	☐	☐	☐		

For peer review only - http://bmjopen.bmj.com/site/about/guidelines.xhtml

**Date:**					
**Start time:**					
**End time:**					
**Training day 3**					
	**Please tick as appropriate**			**If *absent* or *attempted* please provide reasons**	**Other comments**
	**Present**	**Absent**	**Attempted**		
A6 images: risk factors, risks I can/cannot change displayed	☐	☐	☐		
A4 and A6 images: health and wellbeing displayed	☐	☐	☐		
A4 images: dashboard displayed	☐	☐	☐		
Writing instruments given (sticky notes, flipchart paper)	☐	☐	☐		
**Content**					
Give instructions on delivery of 10-minute sessions	☐	☐	☐		
Inform participants of aim of practice delivery (i.e. familiarise self with MLAS, increase confidence)	☐	☐	☐		
Give instructions about giving feedback on facilitator behaviours (3 things you did well)	☐	☐	☐		

For peer review only

For peer review only - http://bmjopen.bmj.com/site/about/guidelines.xhtml

**Date:**					
**Start time:**					
**End time:**					
**Training day 3**					
	**Please tick as appropriate**			**If *absent* or *attempted* please provide reasons**	**Other comments**
	**Present**	**Absent**	**Attempted**		
Each facilitator given an opportunity to deliver their 10-minute session	☐	☐	☐		
Ensured everyone has written 3 positive facilitator behaviours that they saw on a sticky note and give to facilitator	☐	☐	☐		
Provided 3 positive facilitator behaviours and 1 'even better if…'	☐	☐	☐		
Encourage participants to share summaries and feedback on their experience	☐	☐	☐		
**Session 6: Reviewing confidence**					
**Duration of session:**					
**Materials**					
'How confident do I feel?' worksheet	☐	☐	☐		
**Content**					

For peer review only - http://bmjopen.bmj.com/site/about/guidelines.xhtml

**Date:**					
**Start time:**					
**End time:**					
**Training day 3**					
	**Please tick as appropriate**			**If *absent* or *attempted* please provide reasons**	**Other comments**
	**Present**	**Absent**	**Attempted**		
Explores participants' confidence levels to deliver MLAS	☐	☐	☐		
Explore what participants can do to increase their personal confidence to deliver MLAS	☐	☐	☐		
**Session 7: Quality development/Intervention fidelity**					
**Duration of session:**					
**Materials**					
Facilitator behaviours reflection toolkit	☐	☐	☐		
Quality development and intervention fidelity pathway	☐	☐	☐		
**Content**					
Recapped purpose of quality assurance	☐	☐	☐		
Highlighted the importance of self- and peer-reflection	☐	☐	☐		
Discussed facilitator behaviour reflection toolkit	☐	☐	☐		

For peer review only - http://bmjopen.bmj.com/site/about/guidelines.xhtml

**Date:**					
**Start time:**					
**End time:**					
**Training day 3**					
	**Please tick as appropriate**			**If *absent* or *attempted* please provide reasons**	**Other comments**
	**Present**	**Absent**	**Attempted**		
Discussed quality assurance pathway	☐	☐	☐		
Explored what intervention fidelity is					
Described the process for intervention fidelity assessment	☐	☐	☐		
Participants given an opportunity to be involved in discussion (e.g. by giving feedback) and ask questions	☐	☐	☐		
**Session 8: Summary, and post reflections, evaluation**					
**Duration of session:**					
**Materials**					
Training evaluation form given	☐	☐	☐		
Questions relating to MLAS flipchart	☐	☐	☐		
**Content**					

For peer review only - http://bmjopen.bmj.com/site/about/guidelines.xhtml

**Date:**					
**Start time:**					
**End time:**					
**Training day 3**					
	**Please tick as appropriate**			**If *absent* or *attempted* please provide reasons**	**Other comments**
	**Present**	**Absent**	**Attempted**		
Summary of Day 3 training by encouraging participants to share learning about what was covered today	☐	☐	☐		
Provided time for participants to complete training evaluation (day 3) form	☐	☐	☐		
Participants given an opportunity to check if their questions addressed, and to ask any further questions	☐	☐	☐		
Thanked everyone for attending and contributing	☐	☐	☐		
**END OF DAY 3 TRAINING**					

For peer review only

For peer review only - http://bmjopen.bmj.com/site/about/guidelines.xhtml

Supplementary file 3

Training evaluation form

My Life After Stroke - Day 1 - Stroke

Date:_________________

Thank you for attending the Stroke day (Day 1) of the My Life After Stroke (MLAS) training. We would value your reflection on the training you have received today in the following areas. Please place a tick in the relevant box, indicating how much you agree or disagree with each of the following statements:

	Strongly agree	Agree	Neither agree nor disagree	Disagree	Strongly disagree
I am aware of the cognitive problems that can occur following stroke					
I am aware of some strategies that may help stroke survivors manage the effects of cognitive problems					
I have an understanding of the emotional problems that may follow a stroke					
I am aware of some strategies to support stroke survivors manage the effects of their emotional problems					
I have an appreciation of the effects having a stroke could put on relationships					
I am aware of some strategies that may help stroke survivors manage the effects on their relationships					
I have an awareness of communication problems following a stroke					
I have an awareness of how to communicate effectively with stroke survivors					

Continued overleaf…

For peer review only

Day 1 MLAS – Stroke evaluation continued…

	Strongly agree	Agree	Neither agree nor disagree	Disagree	Strongly disagree
I had the opportunity to ask questions and for these to be addressed					
I learnt something new today					

We would value your opinion on the training you attended today. Please answer the following questions:

Which were the three most helpful aspects of the training to you?<br>1.<br>2.<br>3.
Which were the three least helpful aspects of the training to you?<br>1.<br>2.<br>3.
Which part of the training did you find most enjoyable?
Please identify at least one way that the training could be improved?
Any other comments?

Thank you for completing this evaluation sheet

For peer review only

Training evaluation form

My Life After Stroke - Day 2 – Core training

Date:_________________

Thank you for attending the Core day (Day 2) of the My Life After Stroke (MLAS) training. We would value your reflection on the training you have received today in the following areas. Please place a tick in the relevant box, indicating how much you agree or disagree with each of the following statements:

	Strongly agree	Agree	Neither agree nor disagree	Disagree	Strongly disagree
I am aware of the aims of the MLAS programme					
I am aware of why MLAS has been developed					
My role as an MLAS facilitator is to:					
• Demonstrate a curious, non-judgemental approach towards participants					
• Support participants to explore their personal thoughts and feelings					
• Avoid giving specific advice and instead support participants to develop their own solutions and strategies to the challenges they face					
• Facilitate the groups awareness of sources of support and information					
• Promote the sharing of knowledge and ideas between participants and sign post where appropriate					
I have an understanding of the theories that underpin the MLAS programme					
I have an understanding of how these theories have been integrated practically into the MLAS programme					

Continued overleaf…

For peer review only

Day 2 MLAS Core evaluation continued…

	Strongly agree	Agree	Neither agree nor disagree	Disagree	Strongly disagree
I had the opportunity to ask questions and for these to be addressed					
I have learnt new skills					
I believe I can apply the facilitation skills					
I understand the importance of quality assurance					

I have been given an MLAS curriculum. **Yes / No** (delete as appropriate)

We would value your opinion on the training you attended today. Please answer the following questions:

Which were the three most helpful aspects of the training to you?<br>1.<br>2.<br>3.
Which were the three least helpful aspects of the training to you?<br>1.<br>2.<br>3.
Which part of the training did you find most enjoyable?
Please identify at least one way that the training could be improved?
Any other comments?

Thank you for completing this evaluation sheet

For peer review only

Training evaluation form

My Life After Stroke - Day 3 – MLAS-specific training

Date:_________________

Thank you for attending the MLAS-specific day (Day 3) of the My Life After Stroke (MLAS) training. We would value your reflection on the training you have received today in the following areas. Please place a tick in the relevant box, indicating how much you agree or disagree with each of the following statements:

	Strongly agree	Agree	Neither agree nor disagree	Disagree	Strongly disagree
I am aware of how to use the MLAS curriculum					
I have an understanding of the content of the MLAS programme					
I have an understanding of how MLAS can be delivered					
I had the opportunity to see the resources that are used during the MLAS programme					
I found the 'having a go' session useful					
I feel able to begin to plan delivering MLAS					
I feel confident that I will be able to facilitate MLAS					
I had the opportunity to ask questions and for these to be addressed					
I have learnt new skills					
I believe I can apply the skills					

I had the opportunity to deliver a section of the curriculum:

Yes / No / Don't know (please circle)

I received a copy of the Reflection Toolkit:

Yes / No / Don't know (please circle)

Continued overleaf…

For peer review only

Day 3 MLAS-specific evaluation continued…

We would value your opinion on the training you attended today. Please answer the following questions:

Which were the three most helpful aspects of the training to you?<br>1.<br><br>2.<br><br>3.
Which were the three least helpful aspects of the training to you?<br>1.<br><br>2.<br><br>3.
Which part of the training did you find most enjoyable?
Please identify at least one way that the training could be improved?
Any other comments?

Thank you for completing this evaluation sheet

For peer review only

IPCAS trial provider process evaluation/fidelity checklist

Site ID:	Time of call:	Date: of coding:
Spoke to (role):	Conducted by:	Duration of call:

Activity			Tick as appropriate			If *not done* or *attempted*, please provide reason for not delivering (e.g. ran out of time, forgot, deemed unnecessary).	Other comments
			Done	Partially done	Not done		
Structured review	1. 15-item checklist of needs	a) Patient completed checklist *(Thinking back to the most recent review that you did... Did the patient bring a completed checklist of needs?)*					
		b) Discussion of up to 3 needs (as ranked by importance by patient)					
	2. Physical check *(Did you conduct any physical checks at all? If yes what were these? If no/unsure, explore reasons for this (e.g. might've already been done recently))*	a) Blood pressure					
		b) Medication review					
		c) Other (specify)					
	3. Development of action plan(s)	a) Discuss action plan *(Together with the stroke survivor, did you discuss any plans of action going forward to address their needs? If no/unsure, explore reasons why)*					
		b) Log and review of agreed actions *(Cannot be filled if answer to a) is no; however, if yes, ask: Did you make a note of these actions and review or agree it with the patient?)*					
	4. MLAS	a) Provided leaflet about MLAS					
		b) Provided instructions for accessing MLAS					
	5. Direct point of contact to stroke survivor and carer(s)	a) Explain 'direct point of contact' service *(I will now ask questions about the Direct Point of Contact service. Did you discuss/explain the service to the patient)*					
		b) Provide instructions on how to contact practice at the end of review					
	6. Service mapping tool *(When giving information and/or advice, did you refer to the searchable service mapping tool provided by us?)*						
		TOTAL	0	0	0		
		PERCENTAGE	0	0	0		
Use of direct point of contact *(Following on from my previous questions, since the reviews you conducted, has anyone called into the Direct Point of Contact service? If yes, think back to the most recent call. Did you signpost the patient to a specialist service? What about a community-based service?)*	1. Signposting to services	a) Signpost patient to specialist service					
		b) Signpost patient to community service					
	2. Use of service mapping for signposting (as above) *(When you offered information to the patient about either specialist/community services, did you make use of the service mapping tool (i.e. directory) at all?)*						
	3. Provide support and advice	a) Advice for stroke-specific issues					
		b) Other brief telephone support *(Beyond stroke-specific issues, did you offer any other support over the phone?)*					
	4. Arrange follow-up appointment(s)						
	5. Case management *(Finally, during the call, did you offer the patient anything else other than what we had already discussed? E.g. (comprehensive assessment of needs; development of an individualised care plan in collaboration with the service user and relevant others (including families and carers and other staff involved in the service user's care; any additional care given))*						
		TOTAL	0	0	0		
		PERCENTAGE	0	0	0		
Enhanced communication pathways	1. Initial meeting *(We are coming towards the end of the call now, and I wanted to ask some questions about your contact with colleagues in secondary care. Have you had your initial meeting? If no, note reasons why. If yes, ask: Did you have a discussion about your roles, etc. (Note whether meeting was via videoconference or face-to-face))*	a) Discussion of roles/services provided/contact details (video recorded)					
		b) Agreed action plan regarding communication between primary care staff and specialist staff *(In this meeting, did you agree a plan going forward about how best to communicate with each other?)*					
	2. Further contact	a) Meeting (e.g. teleconference, face-to-face, videoconference) *(Since the initial meeting, did you have any further contacts? Tell me about them (then tick off if there was a meeting, or email/phone contact))*					
		b) Other contact (e.g. telephone, email, letter)					
		TOTAL	0	0	0		
		PERCENTAGE	0	0	0		
Duration of structured review	On average, how long did each review run for?						

END OF CHECKLIST

For peer review only

Supplementary file 5

**Assessor's name:**					
**Group/facilitator ID:**					
**Date:**					
**Week 1: Group session 1, Stroke Journey (2.5 hours, with breaks)**					
**Section A: Welcome/introduction**					
**Facilitator(s):**					
**Start time:**					
**End time:**					
	**Tick as appropriate**				
	**Present**	**Absent**	**Partially done**	**If *partially done*, please provide reasons (e.g. ran out of time, forgot, deemed unnecessary)**	**Other comments**
Content					
A1) Welcome/introductions (including housekeeping details, course handbook, course background)	☐	☐	☐		
A2) Introduction to the guidelines for working together as a group	☐	☐	☐		
A3) Opportunity for participants to ask questions	☐	☐	☐		
A4) Introduction to communication aids	☐	☐	☐		
A5) Participants' important questions	☐	☐	☐		
Materials					
A6) Flipchart – names	☐	☐	☐		
A7) Flipcharts – what would you like to get from the course?; what is your important question?	☐	☐	☐		
A8) Flipchart – working together as a group	☐	☐	☐		
Key facilitator behaviours for welcome/introduction					
A9) Uses and refers to participants' comments/quotes	☐	☐	☐		

For peer review only

**Section B: Stroke story**					
**Facilitator(s):**					
**Start time:**					
**End time:**					
	**Tick as appropriate**				
	**Present**	**Absent**	**Partially done**	**If *partially done*, please provide reasons (e.g. ran out of time, forgot, deemed unnecessary)**	**Other comments**
Content					
B1) Stroke story/introduction to narrative approach (including roadmap)	☐	☐	☐		
B2) Eliciting skills, strengths and strategies from participants	☐	☐	☐		
Materials					
B3) Flipchart – skills and strategies	☐	☐	☐		
Key facilitator behaviours for stroke story					
B4) Uses resources and/or participant handbooks appropriately to engage participants and enhance their understanding of the session's key outcomes	☐	☐	☐		
B5) Facilitates people to share their stories about attempts to manage their condition	☐	☐	☐		
B6) Appropriately adapts communication/environment/session delivery to meet the needs of the stroke survivors	☐	☐	☐		

For peer review only - http://bmjopen.bmj.com/site/about/guidelines.xhtml

**Section C: What is stroke?**					
**Facilitator(s):**					
**Start time:**					
**End time:**					
	**Tick as appropriate**				
	**Present**	**Absent**	**Partially done**	**If *partially done*, please provide reasons (e.g. ran out of time, forgot, deemed unnecessary)**	**Other comments**
Content					
C1) What is stroke (including impacts of stroke on individual – physically, emotionally, psychologically and socially)?	☐	☐	☐		
C2) Discussed neuroplasticity (the brain's ability to create new neural pathways and relearn skills	☐	☐	☐		
Materials					
C3) Flipchart – body outline	☐	☐	☐		
C4) Record of 'what is stroke?' (i.e. written on body outline/sticky notes)	☐	☐	☐		
C5) Record of 'effects of stroke on body/person' (i.e. written on body outline/sticky notes)	☐	☐	☐		
Key facilitator behaviours for what is stroke					
C6) Prompts participant to talk about their thoughts and explores misconceptions and gaps in knowledge	☐	☐	☐		
C7) Uses resources and/or participant handbooks appropriately to engage participants and enhance their understanding of the session's key outcomes	☐	☐	☐		

For peer review only

For peer review only - http://bmjopen.bmj.com/site/about/guidelines.xhtml

**Section D: Stroke passenger**					
**Facilitator(s):**					
**Start time:**					
**End time:**					
	**Tick as appropriate**				
	**Present**	**Absent**	**Partially done**	**If *partially done*, please provide reasons (e.g. ran out of time, forgot, deemed unnecessary)**	**Other comments**
Content					
D1) Discussed 'Stroke passenger' analogy	☐	☐	☐		
D2) Eliciting feelings and emotions about stroke	☐	☐	☐		
D3) Provided opportunity for separate carer/stroke survivor discussions	☐	☐	☐		
D4) Discussing skills and coping strategies for living with stroke passenger	☐	☐	☐		
Materials					
D5) Flipchart – how do you feel/think about sharing your life with stroke (carers)?	☐	☐	☐		
D6) Flipchart – how do you feel/think about sharing your life with stroke (stroke survivors)?	☐	☐	☐		
Key facilitator behaviours for stroke passenger					
D7) Prompts participants to express and explore their feelings about the condition or behaviour	☐	☐	☐		
D8) Facilitates people to share their stories about attempts to manage their condition	☐	☐	☐		
D9) Appropriately adapts communication/environment/session delivery to meet the needs of the stroke survivors	☐	☐	☐		
D10) Works as a team with co-facilitator	☐	☐	☐		

For peer review only

For peer review only - http://bmjopen.bmj.com/site/about/guidelines.xhtml

**Section E: What will I do now?**					
**Facilitator(s):**					
**Start time:**					
**End time:**					
	**Tick as appropriate**				
	**Present**	**Absent**	**Partially done**	**If *partially done*, please provide reasons (e.g. ran out of time, forgot, deemed unnecessary)**	**Other comments**
Content					
E1) Session summary/recap of session key outcomes	☐	☐	☐		
E2) 'What will I do now?' worksheet	☐	☐	☐		
Materials					
E3) 'What will I do now?' worksheet in handbook	☐	☐	☐		
Key facilitator behaviours for what will I do now					
E4) Uses resources and/or participant handbooks appropriately to engage participants and enhance their understanding of the session's key outcomes	☐	☐	☐		
E5) Prompts group to summarise their key messages from the session.	☐	☐	☐		
E6) Prompts participants to talk about what they are going to do as a result of the session.	☐	☐	☐		

For peer review only

For peer review only - http://bmjopen.bmj.com/site/about/guidelines.xhtml

Overall facilitator behaviours					
	**Tick as appropriate**				
	**Present**	**Absent**	**Partially done**	**If *partially done*, please provide reasons (e.g. ran out of time, forgot, deemed unnecessary)**	**Other comments**
1. Uses non-judgmental statements in response to participants' verbal utterances	☐	☐	☐		
2. The facilitator seeks clarification of participants contribution	☐	☐	☐		
3. Avoids giving their own opinion	☐	☐	☐		
4. Encourages the group to discuss/answer their own question	☐	☐	☐		
5. Engages participants using rapport building skills	☐	☐	☐		
6. Notices tone/dynamics within the group, acknowledges these and uses them to manage the group	☐	☐	☐		
7. The facilitator uses strategies to manage time within session	☐	☐	☐		
8. Uses active listening skills and open body language to facilitate group/participant talk	☐	☐	☐		
9. Appropriately adapts delivery to meet the needs of the stroke survivors	☐	☐	☐		
10. Works as a team with co-facilitator	☐	☐	☐		

For peer review only

For peer review only - http://bmjopen.bmj.com/site/about/guidelines.xhtml

Assessor comments/notes

For peer review only

For peer review only - http://bmjopen.bmj.com/site/about/guidelines.xhtml

**Assessor's name:**					
**Group/facilitator ID:**					
**Date:**					
**Week 2/3 (circle as appropriate): Managing health and wellbeing (2.5 hours, with breaks)**					
**Section A: Welcome back [20 mins]**					
**Facilitator(s):**					
**Start time:**					
**End time:**					
	**Tick as appropriate**				
	**Present**	**Absent**	**Partially done**	**If *partially done*, please provide reasons (e.g. ran out of time, forgot, deemed unnecessary)**	**Other comments**
Content					
A1) Welcome (including housekeeping details, and ground rules)	☐	☐	☐		
A2) Session outline	☐	☐	☐		
A3) Introduction/recap of managing health and wellbeing handbook and roadmap <u>or</u> roadblocks handbook and roadmap	☐	☐	☐		
A4) Previous week's worksheets; stories and reflections from past week	☐	☐	☐		
A5) Opportunity for participants to ask questions	☐	☐	☐		
Materials					
A6) Roadmap	☐	☐	☐		
A7) Previous week's (relevant) flipcharts on view	☐	☐	☐		
Key facilitator behaviours for welcome					
A8) Uses resources and participant handbooks appropriately to engage participants and enhance understanding of the session's key outcomes	☐	☐	☐		
A9) Prompts the participants to reflect on last week's session and activities	☐	☐	☐		

For peer review only

For peer review only - http://bmjopen.bmj.com/site/about/guidelines.xhtml

**Section B: Managing health and wellbeing [40 mins]**					
**Facilitator(s):**					
**Start time:**					
**End time:**					
	**Tick as appropriate**				
	**Present**	**Absent**	**Partially done**	**If *partially done*, please provide reasons (e.g. ran out of time, forgot, deemed unnecessary)**	**Other comments**
Content					
B1) Risk factors for stroke	☐	☐	☐		
B2) Ways to manage health and wellbeing	☐	☐	☐		
Materials					
B3) A6 headings: 'risks I can change' and 'risks I cannot change'	☐	☐	☐		
B4) A6 images: risk factors	☐	☐	☐		
B5) A4 headings: health and wellbeing	☐	☐	☐		
B6) A6 images: health and wellbeing	☐	☐	☐		
Key facilitator behaviours for managing health and wellbeing					
B7) Facilitates people to share their stories about attempts to manage their condition	☐	☐	☐		
B8) Uses resources and participant handbooks appropriately to engage participants and enhance understanding of the session's key outcomes	☐	☐	☐		
B9) Prompts participant to talk about their thoughts and explores misconceptions and gaps in knowledge	☐	☐	☐		
B10) Encourages the group to discuss/answer their own questions	☐	☐	☐		
B11) Works as a team with co-facilitator	☐	☐	☐		

For peer review only

For peer review only - http://bmjopen.bmj.com/site/about/guidelines.xhtml

**Section C: Making changes [15 mins]**					
**Facilitator(s):**					
**Start time:**					
**End time:**					
	**Tick as appropriate**				
	**Present**	**Absent**	**Partially done**	**If *partially done*, please provide reasons (e.g. ran out of time, forgot, deemed unnecessary)**	**Other comments**
Content					
C1) Elicits feelings and reactions to change	☐	☐	☐		
C2) Discussion of pros and cons of changes	☐	☐	☐		
Materials					
C3) Flipchart – pros and cons grid	☐	☐	☐		
Key facilitator behaviours for making changes					
C4) Prompts participants to discuss their thoughts about possible changes to self-management.	☐	☐	☐		
C5) Prompts participants to review the impact of possible choices on their future health.	☐	☐	☐		
C6) Prompts or allows participants to express and explore their feelings about the condition or behaviour	☐	☐	☐		

For peer review only - http://bmjopen.bmj.com/site/about/guidelines.xhtml
For peer review only

**Section D: Making a plan [35 mins]**					
**Facilitator(s):**					
**Start time:**					
**End time:**					
	**Tick as appropriate**				
	**Present**	**Absent**	**Partially done**	**If *partially done*, please provide reasons (e.g. ran out of time, forgot, deemed unnecessary)**	**Other comments**
Content					
D1) Identifying challenges to planning, problem-solving	☐	☐	☐		
D2) Making a SMART plan	☐	☐	☐		
Materials					
D3) A4 resource: 'My action plan'	☐	☐	☐		
D4) 'My action plan' in handbook	☐	☐	☐		
Key facilitator behaviours for making a plan					
D5) Prompts participants to discuss their thoughts about possible changes to self-management.	☐	☐	☐		
D6) Prompts the participants to reflect on their goals/plans	☐	☐	☐		
D7) Prompts the individual or group to problem solve possible barriers to change (e.g. their desired changes or possible barriers to self-management)	☐	☐	☐		
D8) Encourages the group to discuss/answer their own questions	☐	☐	☐		
D9) Prompts group to summarise key messages from the session	☐	☐	☐		

For peer review only

For peer review only - http://bmjopen.bmj.com/site/about/guidelines.xhtml

**Section E: What will I do now? [10 mins]**					
**Facilitator(s):**					
**Start time:**					
**End time:**					
	**Tick as appropriate**				
	**Present**	**Absent**	**Partially done**	**If *partially done*, please provide reasons (e.g. ran out of time, forgot, deemed unnecessary)**	**Other comments**
Content					
E1) Session summary provided	☐	☐	☐		
E2) 'What will I do now?' worksheet	☐	☐	☐		
Materials					
E3) 'What will I do now?' worksheet in handbook	☐	☐	☐		
Key facilitator behaviours for what will I do now					
E4) Uses resources and participant handbooks appropriately to engage participants and enhance understanding of the session's key outcomes	☐	☐	☐		
E5) Prompts participants to talk about what they are going to do as a result of the session	☐	☐	☐		
E6) Prompts group to summarise their key messages from the session.	☐	☐	☐		

For peer review only

For peer review only - http://bmjopen.bmj.com/site/about/guidelines.xhtml

Overall facilitator behaviours					
	**Tick as appropriate**				
	**Present**	**Absent**	**Partially done**	**If *partially done*, please provide reasons (e.g. ran out of time, forgot, deemed unnecessary)**	**Other comments**
1. Uses non-judgmental statements in response to participants' verbal utterances	☐	☐	☐		
2. The facilitator seeks clarification of participants contribution	☐	☐	☐		
3. Avoids giving their own opinion	☐	☐	☐		
4. Encourages the group to discuss/answer their own questions and/or elicits suggestions from participants	☐	☐	☐		
5. Engages participants using rapport building skills	☐	☐	☐		
6. Notices tone/dynamics within the group, acknowledges these and uses them to manage the group	☐	☐	☐		
7. The facilitator uses strategies to manage time within session	☐	☐	☐		
8. Uses open questions, reflections, appropriate silences and open body language to facilitate group/participant talk	☐	☐	☐		
9. Adapts communication/environment/ session delivery to meet the needs of the stroke survivors	☐	☐	☐		
10. Works as a team with co-facilitator	☐	☐	☐		

For peer review only

For peer review only - http://bmjopen.bmj.com/site/about/guidelines.xhtml

Assessor comments/notes

For peer review only - http://bmjopen.bmj.com/site/about/guidelines.xhtml

**Assessor's name:**					
**Group/facilitator ID:**					
**Date:**					
Week 2/3 (circle as appropriate): Roadblocks (2.5 hrs, with breaks)					
**Section A: Welcome back**					
**Facilitator(s):**					
**Start time:**					
**End time:**					
	**Tick as appropriate**				
	**Present**	**Absent**	**Partially done**	**If *partially done*, please provide reasons (e.g. ran out of time, forgot, deemed unnecessary)**	**Other comments**
Content					
A1) Welcome (including housekeeping details, and ground rules)	☐	☐	☐		
A2) Session outline	☐	☐	☐		
A3) Introduction/recap of managing health and wellbeing handbook and roadmap <u>or</u> roadblocks handbook and roadmap	☐	☐	☐		
A4) Previous week's worksheets; stories and reflections from past week	☐	☐	☐		
A5) Opportunity for participants to ask questions					
Materials					
A6) Roadmap	☐	☐	☐		
A7) Previous week's (relevant) flipcharts on view	☐	☐	☐		
Key facilitator behaviours for welcome back					
A8) Uses resources and participant handbooks appropriately to engage participants and enhance understanding of the session's key outcomes	☐	☐	☐		
A9) Prompts the participants to reflect on last week's session and activities	☐	☐	☐		

For peer review only - http://bmjopen.bmj.com/site/about/guidelines.xhtml

Section B: Roadblocks on my journey					
**Facilitator(s):**					
**Start time:**					
**End time:**					
	**Tick as appropriate**				
	**Present**	**Absent**	**Partially done**	**If *partially done*, please provide reasons (e.g. ran out of time, forgot, deemed unnecessary)**	**Other comments**
Content					
B1) Fictional character facing loss of confidence after stroke (using STOP sign)	☐	☐	☐		
B2) Eliciting thoughts, feelings, sensations, or behaviours as a response to loss of confidence	☐	☐	☐		
B3) Discuss what might help the fictional character, and impact of this on thoughts, feelings, sensations, or behaviours (using GO sign)	☐	☐	☐		
B4) 'What do my roadblocks look like?' worksheet	☐	☐	☐		
Materials					
B5) Roadmap	☐	☐	☐		
B6) Flipcharts – STOP and GO	☐	☐	☐		

For peer review only

For peer review only - http://bmjopen.bmj.com/site/about/guidelines.xhtml

Key facilitator behaviours for roadblocks					
	**Tick as appropriate**				
	**Present**	**Absent**	**Partially done**	**If *partially done*, please provide reasons (e.g. ran out of time, forgot, deemed unnecessary)**	**Other comments**
B7) Facilitates people to share their stories about attempts to manage their condition, and the challenges/roadblocks they face.	☐	☐	☐		
B8) Prompts or allows participants to express and explore their feelings about (the condition or behaviour) during the session	☐	☐	☐		
B9) Encourages the group to discuss/answer their own questions	☐	☐	☐		
B10) Uses and refers to participants' comments/quotes	☐	☐	☐		
B11) Prompts the individual or group to problem solve possible barriers to change (e.g. their desired changes or possible barriers to self-management)	☐	☐	☐		
B12) Prompts participants to review the impact of possible choices on their future health.	☐	☐	☐		

For peer review only

For peer review only - http://bmjopen.bmj.com/site/about/guidelines.xhtml

**Section C: Working it out**					
**Facilitator(s):**					
**Start time:**					
**End time:**					
	**Tick as appropriate**				
	**Present**	**Absent**	**Partially done**	**If *partially done*, please provide reasons (e.g. ran out of time, forgot, deemed unnecessary)**	**Other comments**
Content					
C1) Problems stroke survivors could face Next steps (including session's key outcomes, 'what will I do now?' worksheet, and follow-up appointments)	☐	☐	☐		
C2) Introduce problem-solving approach	☐	☐	☐		
C3) Use fictional character to choose a problem and apply problem-solving approach	☐	☐	☐		
C4) How decisions may be made on the best course of action for solving the problem	☐	☐	☐		
C5) What to do when problems aren't solvable now	☐	☐	☐		
C6) 'Working it out' worksheet	☐	☐	☐		
Materials					
C7) Flipcharts with headings: 1) in my power to solve/change or not in my power to solve/change; 2) 'what could help'; 3) solution: immediate results/long-term results	☐	☐	☐		
C8) Previous flipcharts (e.g. what are my roadblocks; skills and strategies toolkit)	☐	☐	☐		

For peer review only - http://bmjopen.bmj.com/site/about/guidelines.xhtml

Key facilitator behaviours for working it out					
	**Tick as appropriate**				
	**Present**	**Absent**	**Partially done**	**If *partially done*, please provide reasons (e.g. ran out of time, forgot, deemed unnecessary)**	**Other comments**
C9) Uses resources and participant handbooks appropriately to engage participants and enhance understanding of the session's key outcomes	☐	☐	☐		
C10) Uses and refers to participants' comments/quotes	☐	☐	☐		
C11) Prompts participants to discuss their thoughts about possible changes to self-management.	☐	☐	☐		
C12) Prompts participants to review the impact of possible choices on their future health.	☐	☐	☐		
C13) Prompts the individual or group to problem solve possible barriers to change (e.g. their desired changes or possible barriers to self-management)	☐	☐	☐		

For peer review only - http://bmjopen.bmj.com/site/about/guidelines.xhtml

**Section D: What will I do now**					
**Facilitator(s):**					
**Start time:**					
**End time:**					
	**Tick as appropriate**				
	**Present**	**Absent**	**Partially done**	**If *partially done*, please provide reasons (e.g. ran out of time, forgot, deemed unnecessary)**	**Other comments**
Content					
D1) Recap key outcomes	☐	☐	☐		
D2) 'What will I do now?' worksheet	☐	☐	☐		
Materials					
D3) 'What will I do now?' worksheet	☐	☐	☐		
Key facilitator behaviours for what will I do now					
D4) Uses resources and participant handbooks appropriately to engage participants and enhance understanding of the session's key outcomes	☐	☐	☐		
D5) Prompts participants to talk about what they are going to do as a result of the session.	☐	☐	☐		

For peer review only

For peer review only - http://bmjopen.bmj.com/site/about/guidelines.xhtml

Overall facilitator behaviours					
	**Tick as appropriate**				
	**Present**	**Absent**	**Partially done**	**If *partially done*, please provide reasons (e.g. ran out of time, forgot, deemed unnecessary)**	**Other comments**
1. Prompts group to summarise their key messages from the session.	☐	☐	☐		
2. The facilitator seeks clarification of participants contribution	☐	☐	☐		
3. Avoids giving their own opinion	☐	☐	☐		
4. Encourages the group to discuss/answer their own questions and/or elicits suggestions from participants	☐	☐	☐		
5. Engages participants using rapport building skills	☐	☐	☐		
6. Notices tone/dynamics within the group, acknowledges these and uses them to manage the group	☐	☐	☐		
7. The facilitator uses strategies to manage time within session	☐	☐	☐		
8. Uses open questions, reflections, appropriate silences and open body language to facilitate group/participant talk	☐	☐	☐		
9. Adapts communication/environment/ session delivery to meet the needs of the stroke survivors	☐	☐	☐		
10. Works as a team with co-facilitator	☐	☐	☐		

For peer review only

For peer review only - http://bmjopen.bmj.com/site/about/guidelines.xhtml

Assessor comments/notes

For peer review only

For peer review only - http://bmjopen.bmj.com/site/about/guidelines.xhtml

**Assessor's name:**					
**Group/facilitator ID:**					
**Date:**					
**Week 4: Moving forward on my journey (2.5 hours, with breaks)**					
**Section A: Welcome back**					
**Facilitator(s):**					
**Start time:**					
**End time:**					
	**Tick as appropriate**				
	**Present**	**Absent**	**Partially done**	**If *partially done*, please provide reasons (e.g. ran out of time, forgot, deemed unnecessary)**	**Other comments**
Content					
A1) Welcome (including housekeeping details, handbook and ground rules)	☐	☐	☐		
A2) Session outline	☐	☐	☐		
A3) Previous week's action plan/working it out worksheets	☐	☐	☐		
A4) Recap roadmap	☐	☐	☐		
Materials					
A5) Roadmap	☐	☐	☐		
A6) Previous week's (relevant) flipcharts on view	☐	☐	☐		
A7) Moving forward on my journey handbook	☐	☐	☐		
Key facilitator behaviours for welcome back					
A8) Re-engages participants in the journey analogy and resources	☐	☐	☐		
A9) Prompts the participants to reflect on last week's session and activities	☐	☐	☐		

For peer review only - http://bmjopen.bmj.com/site/about/guidelines.xhtml

**Section B: Your relationships**					
**Facilitator(s):**					
**Start time:**					
**End time:**					
	**Tick as appropriate**				
	**Present**	**Absent**	**Partially done**	**If *partially done*, please provide reasons (e.g. ran out of time, forgot, deemed unnecessary)**	**Other comments**
Content					
B1) Ways relationships can be affected by stroke	☐	☐	☐		
B2) Problem-solving exercise in relation to a relationship change	☐	☐	☐		
Materials					
B3) Flipcharts – how can relationships change (one for stroke survivors, one for carers/supporters as applicable)	☐	☐	☐		
B4) Flipcharts – 'what is the problem exactly'; 'what could help'; 'solution: immediate results/long-term results'	☐	☐	☐		

For peer review only

For peer review only - http://bmjopen.bmj.com/site/about/guidelines.xhtml

Key facilitator behaviours for your relationships					
	**Tick as appropriate**				
	**Present**	**Absent**	**Partially done**	**If *partially done*, please provide reasons (e.g. ran out of time, forgot, deemed unnecessary)**	**Other comments**
B5) Prompts or allows participants to express and explore their feelings about (the condition or behaviour) during the session	☐	☐	☐		
B6) Uses and refers to participants' comments/quotes	☐	☐	☐		
B7) Prompts the individual or group to problem solve possible barriers to change (e.g. their desired changes or possible barriers to self-management)	☐	☐	☐		
B8) Prompts participants to review the impact of possible choices on their future health.	☐	☐	☐		
B9) Adapts communication, environment, or session delivery to meet the needs of the stroke survivors	☐	☐	☐		

For peer review only

For peer review only - http://bmjopen.bmj.com/site/about/guidelines.xhtml

**Section C: Revisiting your stroke journey**					
**Facilitator(s):**					
**Start time:**					
**End time:**					
	**Tick as appropriate**				
	**Present**	**Absent**	**Partially done**	**If *partially done*, please provide reasons (e.g. ran out of time, forgot, deemed unnecessary)**	**Other comments**
Content					
C1) Recap the roadmap/journey analogy	☐	☐	☐		
C2) Summary of key messages from each session (e.g. session outcomes/personal take-home messages/skills and strategies discussed during the course)	☐	☐	☐		
C3) Elicit feelings about moving forward from MLAS	☐	☐	☐		
Materials					
C4) Flipchart – ‘skills and strategies toolkit’	☐	☐	☐		
Key facilitator behaviours for revisiting your stroke journey					
C5) Facilitates the group’s recapping of the journey analogy (e.g. prompts group to summarise their key messages from the session)	☐	☐	☐		
C6) Prompts participants to reflect on where they feel they are now	☐	☐	☐		

For peer review only

For peer review only - http://bmjopen.bmj.com/site/about/guidelines.xhtml

**Section D: How will you move forward?**					
**Facilitator(s):**					
**Start time:**					
**End time:**					
	**Tick as appropriate**				
	**Present**	**Absent**	**Partially done**	**If *partially done*, please provide reasons (e.g. ran out of time, forgot, deemed unnecessary)**	**Other comments**
Content					
D1) Discussion of 'warning lights' for physical/emotional health and prevention of these	☐	☐	☐		
D2) Loneliness from the perspective of the fictional character (may also use problem-solving exercise)	☐	☐	☐		
D3) The directory	☐	☐	☐		
Materials					
D4) A4 dashboard images	☐	☐	☐		
D5) Flipchart - 'Skills and strategies toolkit'	☐	☐	☐		
D6) A4 directory	☐	☐	☐		
Key facilitator behaviours for how will you move forward					
D7) Prompts or allows participants to express and explore their feelings about the condition or behaviour	☐	☐	☐		
D8) Encourages the group to discuss/answer their own questions	☐	☐	☐		
D9) Uses and refers to participants' comments/quotes	☐	☐	☐		

For peer review only - http://bmjopen.bmj.com/site/about/guidelines.xhtml
For peer review only

**Section E: Next steps**					
**Facilitator(s):**					
**Start time:**					
**End time:**					
	**Tick as appropriate**				
	**Present**	**Absent**	**Partially done**	**If *partially done*, please provide reasons (e.g. ran out of time, forgot, deemed unnecessary)**	**Other comments**
Content					
E1) Recap session's key outcomes	☐	☐	☐		
E2) 'What will I do now?' worksheet	☐	☐	☐		
E3) Organise follow-up appointments	☐	☐	☐		
Materials					
E4) 'What will I do now?' worksheet in the handbook	☐	☐	☐		
E5) Flipchart – 'what would you like to get from the course?/What is your important question?'	☐	☐	☐		
Key facilitator behaviours for next steps					
E6) Prompts group to summarise their key messages from the session.	☐	☐	☐		
E7) Prompts participants to talk about what they are going to do as a result of the course.	☐	☐	☐		
E8) Encourages the group to discuss/answer their own questions	☐	☐	☐		

For peer review only

Overall facilitator behaviours					
	**Tick as appropriate**				
	**Present**	**Absent**	**Partially done**	**If *partially done*, please provide reasons (e.g. ran out of time, forgot, deemed unnecessary)**	**Other comments**
1. Uses non-judgmental statements in response to participants' verbal utterances	☐	☐	☐		
2. The facilitator seeks clarification of participants contribution	☐	☐	☐		
3. Avoids giving their own opinion	☐	☐	☐		
4. Encourages the group to discuss/answer their own questions and/or elicits suggestions from participants	☐	☐	☐		
5. Engages participants using rapport building skills	☐	☐	☐		
6. Notices tone/dynamics within the group, acknowledges these and uses them to manage the group	☐	☐	☐		
7. The facilitator uses strategies to manage time within session	☐	☐	☐		
8. Uses open questions, reflections, appropriate silences and open body language to facilitate group/participant talk	☐	☐	☐		
9. Adapts communication/environment/ session delivery to meet the needs of the stroke survivors	☐	☐	☐		
10. Works as a team with co-facilitator	☐	☐	☐		

For peer review only

For peer review only - http://bmjopen.bmj.com/site/about/guidelines.xhtml

Assessor comments/notes

For peer review only

For peer review only - http://bmjopen.bmj.com/site/about/guidelines.xhtml

IPCAS trial process evaluation checklist
MLAS facilitator delivery - DESMOND Observation Tool Record Sheet
Group Session 1

Instructions: DOT at 10s (i.e. at the second bleep)

Session A: Welcome/Introduction (30 min)

Facilitator's name:

Assessor's name:

Start time:

End time:

Facilitator talk	Participant talk	Other
Total A:	Total B:	Total C:
Total score= A÷(A+B+C)*100=		

Session B: Stroke story (20 min)

Facilitator's name:

Assessor's name:

Start time:

End time:

Facilitator talk	Participant talk	Other
Total A:	Total B:	Total C:
Total score= A÷(A+B+C)*100=		

For peer review only

IPCAS trial process evaluation checklist

Session C: What is stroke? (25 min)

Facilitator's name:

Assessor's name:

Start time:

End time:

Facilitator talk	Participant talk	Other
Total A:	Total B:	Total C:
Total score= A÷(A+B+C)*100=		

Session D: Stroke passenger (35 min)

Facilitator's name:

Assessor's name:

Start time:

End time:

Facilitator talk	Participant talk	Other
Total A:	Total B:	Total C:
Total score= A÷(A+B+C)*100=		

Session E: What will I do now? (10 min)

Facilitator

Start time:

End time:

Facilitator talk	Participant talk	Other

For peer review only

IPCAS trial process evaluation checklist

Total A:	Total B:	Total C:
Total score= A÷(A+B+C)*100=		

For peer review only

IPCAS trial process evaluation checklist
MLAS facilitator delivery - DESMOND Observation Tool Record Sheet
Group Session 2/3 (circle as appropriate): Managing Health and Wellbeing

Instructions: DOT at 10s (i.e. at the second bleep)

Session A: Welcome back, recap, and introduction (20 min)

Facilitator's name:

Assessor's name:

Start time:

End time:

Facilitator talk	Participant talk	Other
Total A:	Total B:	Total C:
Total score= A÷(A+B+C)*100=		

Session B: Managing health and wellbeing (40 min)

Facilitator's name:

Assessor's name:

Start time:

End time:

Facilitator talk	Participant talk	Other
Total A:	Total B:	Total C:
Total score= A÷(A+B+C)*100=		

For peer review only

IPCAS trial process evaluation checklist

Session C: Making changes (15 min)

Facilitator's name:

Assessor's name:

Start time:

End time:

Facilitator talk	Participant talk	Other
Total A:	Total B:	Total C:
Total score= A÷(A+B+C)*100=		

Session D: Making a plan (35 min)

Facilitator's name:

Assessor's name:

Start time:

End time:

Facilitator talk	Participant talk	Other
Total A:	Total B:	Total C:
Total score= A÷(A+B+C)*100=		

Session E: What will I do now? (10 min)

Facilitator

Start time:

End time:

Facilitator talk	Participant talk	Other

For peer review only

IPCAS trial process evaluation checklist

Total A:	Total B:	Total C:
Total score= A÷(A+B+C)*100=		

For peer review only

IPCAS trial process evaluation checklist
MLAS facilitator delivery - DESMOND Observation Tool Record Sheet
Group Session 2/3 (circle as appropriate): Roadblocks

Instructions: DOT at 10s (i.e. at the second bleep)

Session A: Welcome back and introduction (20 min)

Facilitator's name:

Assessor's name:

Start time:

End time:

Facilitator talk	Participant talk	Other
Total A:	Total B:	Total C:
Total score= A÷(A+B+C)*100=		

Session B: Roadblocks on my journey (45 min)

Facilitator's name:

Assessor's name:

Start time:

End time:

Facilitator talk	Participant talk	Other
Total A:	Total B:	Total C:
Total score= A÷(A+B+C)*100=		

For peer review only

IPCAS trial process evaluation checklist

Session C: Working it out (45 min)

Facilitator's name:

Assessor's name:

Start time:

End time:

Facilitator talk	Participant talk	Other
Total A:	Total B:	Total C:
Total score= A÷(A+B+C)*100=		

Session D: What will I do now? (10 min)

Facilitator's name:

Assessor's name:

Start time:

End time:

Facilitator talk	Participant talk	Other
Total A:	Total B:	Total C:
Total score= A÷(A+B+C)*100=		

For peer review only

IPCAS trial process evaluation checklist
MLAS facilitator delivery - DESMOND Observation Tool Record Sheet
Group Session 4: Moving Forward on my Journey

Instructions: DOT at 10s (i.e. at the second bleep)

Session A: Welcome back, recap, and introduction (20 min)

Facilitator's name:

Assessor's name:

Start time:

End time:

Facilitator talk	Participant talk	Other
Total A:	Total B:	Total C:
Total score= A÷(A+B+C)*100=		

Session B: Your relationships (45 min)

Facilitator's name:

Assessor's name:

Start time:

End time:

Facilitator talk	Participant talk	Other
Total A:	Total B:	Total C:
Total score= A÷(A+B+C)*100=		

For peer review only

IPCAS trial process evaluation checklist

Session C: Revisiting your stroke journey (10 min)

Facilitator's name:

Assessor's name:

Start time:

End time:

Facilitator talk	Participant talk	Other
Total A:	Total B:	Total C:
Total score= A÷(A+B+C)*100=		

Session D: How will you move forward? (30 min)

Facilitator's name:

Assessor's name:

Start time:

End time:

Facilitator talk	Participant talk	Other
Total A:	Total B:	Total C:
Total score= A÷(A+B+C)*100=		

Session E: What will I do now? (10 min)

Facilitator

Start time:

End time:

Facilitator talk	Participant talk	Other

For peer review only - http://bmjopen.bmj.com/site/about/guidelines.xhtml

For peer review only

IPCAS trial process evaluation checklist

Total A:	Total B:	Total C:
Total score= A÷(A+B+C)*100=		

For peer review only

IPCAS trial patient process evaluation/fidelity checklist - patient							
Site ID:			Participant ID:			Date:	
Time of call:			Conducted by:			Duration of call:	
Activity			Tick as appropriate			If *no* or *unsure*, please provide reasons why/any details	Other comments
			Yes	No	Unsure		
Structured review (attending at the GP surgery)	1. Attended structured review (If no/unsure, ask for reasons why)						
	2. 15-item checklist of needs (If no/unsure, ask for reasons why)	a) Patient completed checklist					
		b) Discussion of up to 3 needs (as ranked by importance by patient) *(Can you tell me which needs you discussed?)*					
	4. Development of action plan(s) (If no/unsure, ask for reasons why)	a) Discussed action plan *(Did you talk about how to address your needs?)*					
		b) Opportunity to log and review agreed actions *(Did you agree a specific plan going forward to address these needs? E.g., book an appointment with physio on Monday)*					
	3. Physical check (If no/unsure, ask for reasons why)	a) Blood pressure					
		b) Medication review *(Did you discuss your medication(s)?)*					
		c) Other (specify) *(Was there anything else that you talked about regarding your physical health?)*					
	5. MLAS (If no/unsure, ask for reasons why)	a) Received leaflet about *MLAS* *(Would you like me to post you one if not?)*					
		b) Received instructions for accessing MLAS (they're on the bottom of the information sheet: "..call Emily...")					
	6. Direct point of contact to stroke survivor and carer(s)	a) Received information about 'direct point of contact' service *(Were you given information about a service in your GP practice where you can get in touch if you have any stroke-related needs/concerns?)*					
		b) Received instructions on how to contact practice at the end of review *(Did you receive instructions about how to access this telephone service?) IF 'NO', FINISH.*					
		TOTAL					
		PERCENTAGE					
Use of direct point of contact (i.e. calling the practice about their stroke-specific needs)	1. Called practice	a) Called practice *(Since your appointment, have you phoned the practice about your stroke? If no/unsure, ask for reasons why. After asking for reasons why, end assessment if they didn't contact the practice)*					
		b) Received signposts to specialist service *(When you called, did you receive advice about accessing any specialist healthcare professionals or services (e.g. physio, speech and language therapist)? If no/unsure, ask for reasons why)*					
		c) Received signposts to community service *(When you called, did you receive advice about accessing any services in your community (e.g. leisure services/local activity groups, GP/nurse appointment)? If no/unsure, ask*					
	2. Receive support and advice	a) Received advice for stroke-specific issues *(that you raised?)*					
		b) Other brief telephone support *(Was there anything else that you talked about that you received support for when you phoned?)*					
	3. Follow-up appointment(s) arranged for						
	4. Case management *(Finally, was there anything else that was given to you during this call? For example, any further support about how to address your needs, any agreed plans for going forward/addressing your needs?)*						
	TOTAL						
	PERCENTAGE						
END OF CHECKLIST							

For peer review only
For peer review only - http://bmjopen.bmj.com/site/about/guidelines.xhtml

Supplementary file 8

Thank you for attending **Individual appointment 1**.

Please tick (✓) **agree, disagree, don't know** or **does not apply** for each of the statements below.

	Agree	Disagree	Don't know	Does not apply
1. The facilitator explained **'My life after stroke'** to me.				
2. The facilitator invited me to **ask questions** about 'My life after stroke'.				
3. I understand what **'My life after stroke'** is.				
4. I felt I could talk about what life was like **before and after** my stroke.				
5. The facilitator invited me to share any **problems I might face** in attending the course.				
6. I had the chance to talk about **ways to address** the problems I might face in attending the course.				
7. The facilitator invited my carer to come to my appointment.				
8. The facilitator invited my carer to **share experiences** if they wanted to.				

Please turn over.

For peer review only - http://bmjopen.bmj.com/site/about/guidelines.xhtml

For peer review only

Please write your **comments** below:

For peer review only

For peer review only - http://bmjopen.bmj.com/site/about/guidelines.xhtml

Thank you for attending **Stroke Journey**.

Please tick (✓) **agree, disagree, don't know** or **does not apply** for each of the statements below.

	Agree	Disagree	Don't know	Does not apply
1. I felt like I could contribute to the session if I wanted to.				
2. I felt like I could participate in the session activities.				
3. The session included a discussion about my **stroke story**.				
4. I had a chance to think about where I am on my **journey** since my stroke.				
5. I know what a stroke is.				
6. I know how a stroke can affect me.				
7. I understand what **neuroplasticity** is.				
8. I have a **strategy** that can help me deal with the effects of my stroke.				
9. The session included a discussion about **feelings** after having a stroke.				

Please turn over.

For peer review only - http://bmjopen.bmj.com/site/about/guidelines.xhtml

Please tick ✓ **agree**, **disagree**, **don't know** or **does not apply** for each of the statements below.

	Agree	Disagree	Don't know	Does not apply
10. I am aware of the **barriers** to moving forward with my life after stroke.				
11. I had a chance to talk to other stroke survivors **without my carer**.				
12. I had a chance to complete the '**what will I do now?'** worksheet.				
13. I know what I am going to think about doing differently from today.				
14. I received my **'Stroke journey'** handbook.				

Please write your **comments** below:

For peer review only

Thank you for attending **Managing health and wellbeing**.

Please tick (✓) **agree, disagree, don't know** or **does not apply** for each of the statements below.

	Agree	Disagree	Don't know	Does not apply
1. I felt like I could contribute to the session if I wanted to.				
2. I felt like I could participate in the session activities.				
3. The session included an activity on **risk factors for stroke**.				
4. I am aware of what my risks are for having another stroke.				
5. I know what I could do to help **reduce** my risk of having another stroke.				
6. I know what I could do to **stay healthy**.				
7. I understand the **pros and cons** to making change.				
8. I made an **action plan**.				
9. I am confident I can achieve my plan.				

Please turn over.

For peer review only

For peer review only - http://bmjopen.bmj.com/site/about/guidelines.xhtml

Please tick ✓ **agree**, **disagree**, **don't know** or **does not apply** for each of the statements below.

	Agree	Disagree	Don't know	Does not apply
10. There were opportunities to **ask questions** at the session.				
11. I was able to learn from other people in the group.				
12. I had a chance to complete the '**what will I do now?'** worksheet.				
13. I know what I am going to think about doing differently from today.				
14. I received my **'Managing health and wellbeing'** handbook.				

Please write your **comments** below:

For peer review only

For peer review only - http://bmjopen.bmj.com/site/about/guidelines.xhtml

Thank you for attending **Roadblocks**.

Please tick (✓) **agree, disagree, don't know** or **does not apply** for each of the statements below.

	Agree	Disagree	Don't know	Does not apply
1. I felt like I could contribute to the session if I wanted to.				
2. I felt like I could participate in the session activities.				
3. The group discussed the link between **thoughts, feelings, physical sensations, and behaviour**.				
4. The group discussed what problems or **'roadblocks'** people might face after a stroke.				
5. I had a chance to complete **'what are my roadblocks?'** worksheet.				
6. The session included a **problem-solving** activity.				
7. The facilitators invited me to share my problems and feelings.				
8. I am able to think about my own problems or roadblocks.				
9. I am able to think about how to solve or manage my own problems or roadblocks.				

Please turn over.

For peer review only - http://bmjopen.bmj.com/site/about/guidelines.xhtml
For peer review only

Please tick ✓ **agree**, **disagree**, **don't know** or **does not apply** for each of the statements below.

	Agree	Disagree	Don't know	Does not apply
10. There were opportunities to **ask questions** at the session.				
11. I was able to learn from other people in the group.				
12. I had a chance to complete the '**what will I do now?'** worksheet.				
13. I know what I am going to think about doing differently from today.				
14. I received my **'Roadblocks'** handbook.				

Please write your **comments** below:

For peer review only

For peer review only - http://bmjopen.bmj.com/site/about/guidelines.xhtml

Thank you for attending **‘Moving forward on my journey’**.

Please tick (✓) **agree, disagree, don’t know** or **does not apply** for each of the statements below.

	Agree	Disagree	Don’t know	Does not apply
1. I felt like I could contribute to the session if I wanted to.				
2. I felt like I could participate in the session activities.				
3. I was able to think about how my relationships have changed since having had my stroke.				
4. The facilitators invited me to share my experiences of changing relationships.				
5. The session included a **problem-solving** activity.				
6. I had a chance to talk to other stroke survivors **without my carer**.				
7. I had a chance to think about where I am on my **journey** since my stroke.				
8. I am aware of how I can keep track of my **physical and mental health**.				
9. I know what I could do to **stay healthy**.				

Please turn over.

For peer review only - http://bmjopen.bmj.com/site/about/guidelines.xhtml

For peer review only

Please tick ✓ **agree**, **disagree**, **don't know** or **does not apply** for each of the statements below.

	Agree	Disagree	Don't know	Does not apply
10. There were opportunities to **ask questions** at the session.				
11. The group discussed ways to get **support or information**.				
12. I had a chance to complete the '**what will I do now?'** worksheet.				
13. I know what I am going to think about doing differently from today.				
14. I received my **'Moving forward on my journey'** handbook.				
15. I received my **'Directory'**.				

Please write your **comments** below:

For peer review only

For peer review only - http://bmjopen.bmj.com/site/about/guidelines.xhtml

Thank you for attending **Individual appointment 2**.

Please tick (✓) **agree, disagree, don't know** or **does not apply** for each of the statements below.

	Agree	Disagree	Don't know	Does not apply
1. I was able to reflect on where I am on my **journey** after stroke.				
2. The facilitator invited me to talk about my experience of the **'My life after stroke'** course.				
3. The facilitator invited me to complete the **'Where am I on my journey?'** worksheet.				
4. I was able to **ask questions** if I wanted to.				
5. The facilitator and I talked about my **plans** after 'My life after stroke'.				
6. The facilitator checked if I had any questions.				
7. The appointment included **information about services** that I can access.				

Please turn over.

For peer review only - http://bmjopen.bmj.com/site/about/guidelines.xhtml

For peer review only

Please tick ✓ **agree**, **disagree**, **don't know** or **does not apply** for each of the statements below.

	**Agree**	**Disagree**	**Don't know**	**Does not apply**
8. My carer was invited to come to my appointment.				
9. The facilitator invited my carer to **share experiences** if they wanted to.				

Please write your **comments** below:

For peer review only

For peer review only - http://bmjopen.bmj.com/site/about/guidelines.xhtml
